# Photoluminescent, dielectric, and magnetic responsivity to the humidity variation in SHG-active pyroelectric manganese(ii)-based molecular material†

**DOI:** 10.1039/d5sc00404g

**Published:** 2025-04-15

**Authors:** Aleksander Hoffman, Mikolaj Zychowicz, Junhao Wang, Keisuke Matsuura, Fumitaka Kagawa, Jan Rzepiela, Michal Heczko, Sebastian Baś, Hiroko Tokoro, Shin-ichi Ohkoshi, Szymon Chorazy

**Affiliations:** a Faculty of Chemistry, Jagiellonian University Gronostajowa 2 30-387 Krakow Poland simon.chorazy@uj.edu.pl; b Doctoral School of Exact and Natural Sciences, Jagiellonian University Lojasiewicza 11 30-348 Krakow Poland; c Department of Materials Science, Faculty of Pure and Applied Science, University of Tsukuba 1-1-1 Tennodai Tsukuba Ibaraki 305-8573 Japan; d Department of Physics, Tokyo Institute of Technology 2-12-1 O-Okayama, Meguro Tokyo 152-8551 Japan; e RIKEN Center for Emergent Matter Science (CEMS) 2-1 Hirosawa Wako 351-0198 Japan; f Department of Chemistry, School of Science, The University of Tokyo 7-3-1 Hongo, Bunkyo-ku Tokyo 113-0033 Japan

## Abstract

Multifunctional response to external stimuli which engages various properties, including optical, dielectric, magnetic, or mechanical, can be the source of new generations of highly sensitive sensors and advanced switches. Such responsivity is expected for molecular materials based on metal complexes whose properties are often sensitive to even subtle changes in a particular stimulus. We present a novel hybrid organic–inorganic salt based on earth-abundant divalent manganese ions forming two types of complexes, octahedral [Mn^II^(Me-dppmO_2_)_3_]^2+^ cations with methyl-functionalized bis(diphenylphosphino)methane dioxide ligands and tetrahedral [Mn^II^Cl_4_]^2−^ anions. These ions crystallize with water molecules leading to the molecular material [Mn^II^(Me-dppmO_2_)_3_][Mn^II^Cl_4_]·H_2_O (1). We show that, due to the simple methyl substituent on the diphosphine-type ligand, 1 reveals a polar crystal structure of the *Cc* space group as confirmed by the single-crystal X-ray diffraction, second-harmonic generation (SHG) effect, piezoelectric response, and pyroelectricity. Besides these non-centrosymmetricity-related non-linear optical and electrical features, this material combines three other physical properties, *i.e.*, visible room-temperature (RT) photoluminescence (PL) originating from d–d electronic transitions of octahedral Mn(ii) complexes, dielectric relaxation in *ca.* 170–300 K range related to Bjerrum-type orientation defects of water molecules, and slow magnetic relaxation below 3 K related to spin–phonon interactions involving paramagnetic Mn(ii) centers. We demonstrate that these three physical effects detected in 1 are sensitive to humidity variation that governs the RT–PL intensity, leads to the ON/OFF switching of dielectric relaxation around RT, and non-trivially modulates the magnetic relaxation at cryogenic temperatures. Thus, we report a unique molecular material revealing broadened multifunctionality and triple physical responsivity to the humidity change exploring luminescent, dielectric, and magnetic properties.

## Abstract 2

Molecular material based on Mn(ii) centers covered by CH_3_-functionalized diphosphine dioxides exhibits SHG activity, pyroelectricity, and rich responsivity to the humidity change.

## Graphical abstract

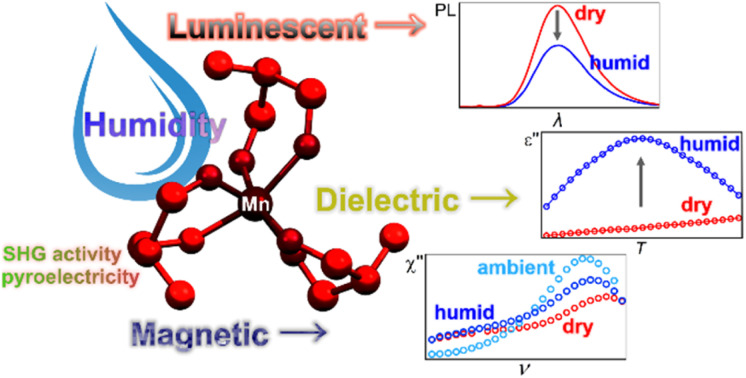

## Introduction

To ensure the continuous development of new technologies based on miniaturized, more efficient, and more economical devices, researchers need to search for new generations of materials with improved optical, electrical, magnetic, and mechanical characteristics.^1–6^ Among them, a promising route to challenge the issue of high-performance future devices is provided by multifunctional materials that combine a few different physical functionalities, *e.g.*, some from the set of magnetic, piezo-/pyro-/ferroelectric, luminescent, and non-linear optical activity, within a single phase.^7–14^ This approach was realized using carefully designed classical materials, such as oxides or fluorides, but it was shown that multifunctionality can be more effectively achieved by playing with molecular materials based on organic species or metal complexes.^10–14^

Besides being efficient prerequisites for multifunctionality, another advantage of molecular materials is related to the sensitivity of their physical properties to various chemical and physical stimuli, such as solvent vapors, other guest molecules, temperature, pressure, or light.^15–23^ This great responsivity to external stimuli opens wide applications in display and memory devices, more importantly, sensors, as well as molecular switches and machines.^24–28^ One of the crucial stimuli that was demonstrated to affect the physical properties of molecular systems is relative humidity which governs the number of water molecules adsorbed by the material leading to the modulation of its magnetic,^29–32^ optical (including photoluminescent),^33–36^ electrical,^37–41^ or mechanical properties.^42–44^

In this context, our goal was to construct multifunctional material that can combine numerous physical functionalities, including magnetic, electrical, and optical ones, as well as whose multiple physical properties can be diversely influenced by relative humidity (RH). Such a material can be then considered as a promising response to the challenge of miniaturized and more efficient devices. Moreover, it is expected to become a good candidate for an effective humidity sensor taking advantage of various possible responses to the RH changes, including ON/OFF switching of the specific property, its continuous change, or the sensitivity only to the particular RH range.^45–48^ To address these challenges, we decided to focus on coordination compounds incorporating manganese(ii) centers which combine a few promising features in the context of multimodal humidity sensors, including their relatively low cost related to good earth abundance,^49,50^ low toxicity,^51^ and high ground state spin of 5/2 under the intermediate-to-weak ligand field, the latter providing families of Mn(ii)-based molecular magnets.^52–56^ Moreover, it was demonstrated that Mn(ii) complexes, placed in either octahedral or tetrahedral coordination geometries, can be strongly photoluminescent (PL) due to the effective d–d emissive electronic transitions. This was found to be the basis of related molecular solid luminophores, revealing sometimes also the optical sensitivity to external stimuli, *e.g.*, temperature.^57–62^ The materials based on Mn(ii) complexes were also shown to exhibit various electrical effects, including switchable dielectric constant and ferroelectricity, which were even combined with magnetic and PL effects in unique multifunctional materials.^63–66^ All these reports suggest that materials based on Mn(ii) complexes are the rational choice for the design of humidity-responsive systems exploring a series of physical properties, including luminescent, dielectric, and magnetic ones. Our direct inspiration was the work of A. S. Berezin *et al.* who reported a molecular hybrid salt of the formula of [Mn^II^(dppmO_2_)_3_] [Mn^II^Cl_4_] (dppmO_2_ = bis(diphenylphosphino)methane dioxide), exhibiting double thermosensitive PL signal originating from two incorporated Mn(ii) complexes.^67^ We decided to modify the dppmO_2_ ligand functionalizing it by a methyl group to lower the crystal's symmetry. This strategy was expected to be supportive in achieving a non-centrosymmetric space group opening a pathway for non-linear optical effects, such as second-harmonic generation (SHG), and electrical properties, such as piezo- and pyroelectricity.^68–71^ Moreover, the methylation was expected to decrease the C–H bond acidity in the dppmO_2_ entity which disturbs the hydrogen bonding. This, together with the enlarged size of the ligand after methylation, was the prerequisite to better water sorption property of the material that was expected to contribute to the switching of both luminescent properties due to the modulation of the number of emission-quenching O–H oscillators as well as dielectric properties due to the strong variation of the number of incorporated water molecules of high polarity. The increased size of the ligand was also expected to contribute to the induction and switching of a slow magnetic relaxation effect due to the impact on the geometry of Mn(ii) centers and the increase of their magnetic isolation in the crystal lattice. The latter was also expected to depend on the number of incorporated water molecules contributing to spin–phonon relaxation routes.^72^ Realizing all these suggestions and concepts, here, we report a novel hybrid organic-inorganic salt, [Mn^II^(Me-dppmO_2_)_3_][Mn^II^Cl_4_]·H_2_O (1), which crystallizes in a polar *Cc* space group resulting in the SHG activity, piezoelectric response and pyroelectricity, and reveals the combination of photoluminescence, as well as dielectric and magnetic relaxation effects, all responsive to the humidity variation. The origins of observed physical phenomena and the modulation of the mentioned set of luminescent, dielectric, and magnetic effects upon the RH change in the surroundings of the obtained material, as well as the resulting potential for humidity sensing, are thoroughly discussed based on extensive experimental structural and physicochemical studies, supported by the results of *ab initio* calculations.

## Results and discussion

### Structural studies

**Fig. 1:**
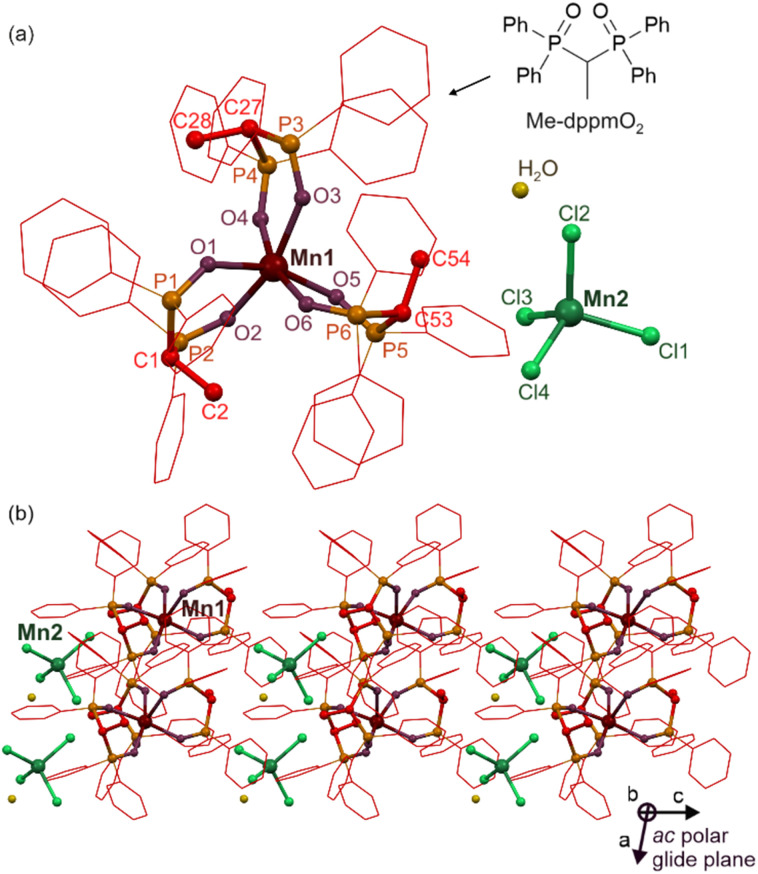
The crystal structure of 1, determined at 100(2) K (1^100K^ phase) including the insight into the molecular building unit consisting of two Mn(ii) complexes and a water molecule of crystallization, shown with the structure of a Me-dppmO_2_ ligand, (a) and the view of the polar arrangement of the molecular components within the supramolecular network with the indication of ac polar glide plane (b).

The colorless block crystals of [Mn^II^(Me-dppmO_2_)_3_][Mn^II^Cl_4_]·H_2_O (1) were synthesized by mixing Mn^II^Cl_2_·4H_2_O with Me-dppmO_2_ (bis(diphenylphosphino)-1,1-ethane dioxide) ligand in the 2 : 3 ratio in MeCN and then recrystallizing the primarily formed precipitate from the MeCN/MeOH solution layered with Et_2_O (see Experimental section and Fig. S1–S3 and Table S1 in the ESI†). The obtained compound was preliminarily characterized by IR spectroscopy, TGA, and CHN elemental analyses (Fig. S1 and S2†), while the crystal structure of 1 was determined using the single-crystal X-ray diffraction (SC-XRD) method (Table S1†) performed on air-dried crystals containing Mn(ii) complexes and water molecules of crystallization (Fig. 1).

The crystal structure of the obtained material was determined first at 100(2) K (1^100K^ phase), then at 270(2) K (1^270K^), at 300(2) K (1^deh,300K^), for which the dehydration of the material occurs, and, finally, at 330(2) K (1^deh,330K^, see Fig. S4–S7 and Tables S1–S5†). At all indicated temperatures, the structural analyses showed that 1 crystallizes in a non-centrosymmetric polar *Cc* space group of a monoclinic crystal system (Table S1†). It is built of octahedral [Mn^II^(Me-dppmO_2_)_3_]^2+^ molecular cations and tetrahedral [Mn^II^Cl_4_]^2−^ anions. These metal complexes are accompanied by water molecules of crystallization, *i.e.*, one such molecule per the pair of octa- and tetrahedral Mn(ii) centers, but this happens only for the crystalline phases measured at 100(2) and 270(2) K, as, at 300(2) K, these solvent molecules are removed. The cationic Mn(ii) complex contains three *O*,*O*-bidentate Me-dppmO_2_ ligands, thus the {Mn^II^O_6_} coordination sphere is observed. This metal complex reveals the geometry of a significantly distorted octahedron as depicted by Continuous Shape Measure (CShM) parameters (Table S8†). For 1^100K^, the Mn–O distances vary from 2.159(4) Å to 2.222(4) Å. The O–Mn–O angles formed by oxygen atoms within each of Me-dppmO_2_ ligands vary from 81.84(15)° to 85.00(15)°, while the analogous angles for oppositely aligned O-atoms vary from 159.98(15)° to 163.45(15)°, visualizing the large distortion from an ideal octahedral geometry (Tables S2–S6†). The anionic Mn(ii) complex contains four chlorido ligands, exhibiting a geometry close to an ideal tetrahedron (Table S8†). For 1^100K^, the Mn–Cl distances vary from 2.3574(18) Å to 2.3689(17) Å with the Cl–Mn–Cl angles lie in the range from 105.85(7)° to 114.20(7)°. Due to the expanded organic Me-dppmO_2_ ligands, the closest distance between neighboring octahedral Mn1 complexes, *i.e.*, Mn1⋯Mn1, is 14.05 Å, the analogous closest distance between tetrahedral Mn2 sites is 14.50 Å, while between Mn1 and Mn2 centers is 8.15 Å. Besides Mn(ii) complexes, water molecules of crystallization are present in the crystal structure of 1^100K^. They are placed in the cavities formed by chlorido ligands of [Mn^II^Cl_4_]^2−^ anions and phenyl rings of [Mn^II^(Me-dppmO_2_)_3_]^2+^ cations (Fig. 1b, S4, and S5†). These cavities are aligned along the a crystallographic direction with the windows between them, thus the formation of a kind of channels with water molecules can be postulated. This is probably the main direction used for the removal of water molecules upon the formation of 1^deh,300K^ (Fig. S6†), especially since these water molecules do not form distinct supramolecular interactions with the mentioned aromatic rings or chlorido ligands. Only for the latter some weak hydrogen bonds can be noticed with the oxygen–chlorine distances of 3.3–3.5 Å. As a result, upon heating to 300 K in the flow of inert gas, the easy removal of water molecules is observed. This does not lead to drastic structural changes, thus the crystal structure of a dehydrated form, 1^deh,300K^, could be determined using the SC-XRD method (Fig. S6 and Table S4†). We checked that this dehydration process occurs around room temperature and above as the crystal of 1 at 270(2) K (1^270K^ phase) was found to still contain water molecules of crystallization (Fig. S5 and Table S3, the related comment in the Experimental section in the ESI†). They are efficiently removed at 300 K as proven by the crystal structure of 1^deh,300K^. However, except for the lack of solvent molecules of crystallization, the metric parameters of the dehydrated phase are very similar to the hydrated one (Fig. S1–S4 and Tables S2–S5†). It indicates that the supramolecular framework of 1 is rather rigid and weakly bonded water molecules are simply removed leaving the empty structural pores. The additional SC-XRD measurement at 330(2) K (1^deh,330K^) shows that the resulting dehydrated phase becomes stable at further heating without any structural reorganization in comparison to the 1^deh,300K^ phase (Fig. S7 and Table S5†).

Besides the above-presented structural studies, to get further proof for the dehydration process, we performed the additional SC-XRD experiment in which the crystal of 1 taken from the mother solution was heated to 300(2) K and left for 2 hours in the flow of dry inert gas, then cooled down to 100(2) K and measured. The crystal structure of the resulting phase of 1^deh,100K^ is almost identical to those found for the dehydrated phase of 1^deh,300K^ measured at a higher temperature, except for the expected cooling-induced shortening of all bond lengths. Moreover, similar to 1^deh,300K^, the structure of 1^deh,100K^ does not contain any solvent molecules revealing a very small residual electron density of 0.329 e Å^−3^ (Fig. S8 and Tables S1, S6, S8†). This data, especially confronted with the structure of the hydrated form of 1^100K^ measured for the single crystal taken directly from the mother solution, air-dried and cooled to 100(2) K (Fig. 1), confirms the complete dehydration process of 1 that occurs under the flow of inert gas at 300(2) K and above (Fig. S6–S8†). Without these conditions, the air-dried crystals of 1 remain at room temperature in the hydrated form as indicated by SC-XRD studies for the 1^100K^ phase and the CHN elemental analysis confirming the presence of water molecules for the air-dried polycrystalline sample of 1 (see Experimental section in the ESI†). To get additional insight into the conditions for the dehydration process of 1, the DSC measurement, for the powder sample placed in the Al pan with a pierced lid enabling the removal of solvent molecules upon heating, was conducted (see Fig. S59 with the comment, as well as the technical details in the Experimental section in the ESI†). The obtained DSC curves, gathered upon heating in the dry inert gas atmosphere, reveal the positive peak which can be assigned to the dehydration as it disappears for the subsequent cooling and within the next cycle of heating and cooling. The related temperature range varies on the sweeping rate of the DSC measurement. However, for the relatively slow heating of 2 °C min^−1^, the DSC curve indicates that the dehydration happens in the 10–60 °C range and is complete at *ca.* 60 °C under such continuous heating procedure. Further studies on the dehydration at room temperature using the long exposition to the dry inert gas are discussed below in the Water vapor sorption section.

As mentioned above, there are rather small changes in the metric parameters upon the dehydration of 1. This is well visible when comparing the structures of hydrated and dehydrated phases determined at the same temperature of 100(2) K, *i.e.*, 1^100K^ and 1^deh,100K^ (Tables S1, S2 and S6†). As a result, the powder X-ray diffraction (P-XRD) patterns simulated from the respective structural models are almost identical while the analogous P-XRD pattern simulated for 1^deh,300K^ differs also very subtly only due to the thermal expansion effect (Fig. S10†). The P-XRD patterns were measured for the powder sample of 1 stabilized at four different conditions, including ambient relative humidity of *ca.* 40% (corresponding to the hydrated form, 1), high-humidity conditions of *ca.* 90% RH (named the high-humidity phase, 1^HH^), the dry atmosphere of 0% RH (vacuum conditions followed by inert gas, corresponding to the dehydrated phase, 1^deh^), and the wet sample (measured under the water solution, abbreviated as 1^wet^). All the resulting P-XRD patterns suit well the calculated ones for 1^100K^ as well as 1^deh,300K^ (Fig. S10†). First, this proves the phase purity of the obtained material and the structural identity of the bulk sample with the single crystal selected for the SC-XRD experiment. Moreover, the negligibly small differences between the P-XRD patterns of 1^HH^, 1, 1^deh^, and 1^wet^ phases confirm that the supramolecular framework of this material can be considered rigid and not affected by the removal of water molecules of crystallization that simply leave and further enter the existing structural pores. Thus, their impact on the structural features of the framework (depicted by the P-XRD peaks) is negligible. In addition, these studies indicate the perfect stability of the material under high humidity (90% RH) or even in the water solution which is crucial for the reliability of the further investigation of various physical responses to the humidity variation (see below).

For the comparison, using analogous synthetic conditions as presented for 1, we prepared the crystals of [Mn^II^(dppmO_2_)_3_] [Mn^II^Cl_4_]·2MeCN (2), containing a previously reported six-coordinated Mn(ii) complexes with unmethylated bis(di-phenylphosphino)methane dioxide ligands (Fig. S9 and Tables S1, S7, S8†).^67^ On the contrary to 1, they crystallize in the centrosymmetric *P*2_1_/*c* space group. Moreover, they contain six-coordinated Mn1 complexes with three dppmO_2_ ligands which are of a perfect octahedral geometry being very different than the analogous Mn1 centers in 1 (Table S8†). The crystal structure of 2 also differs from 1 in the solvent content as MeCN molecules instead of water ones were detected in the structural pores (Fig. S9†). However, upon air-drying, they are easily replaced with water from the air. This solvent exchange was found not to affect significantly the whole structure as suggested by the P-XRD data (Fig. S10†).

### Second-harmonic generation activity

**Fig. 2:**
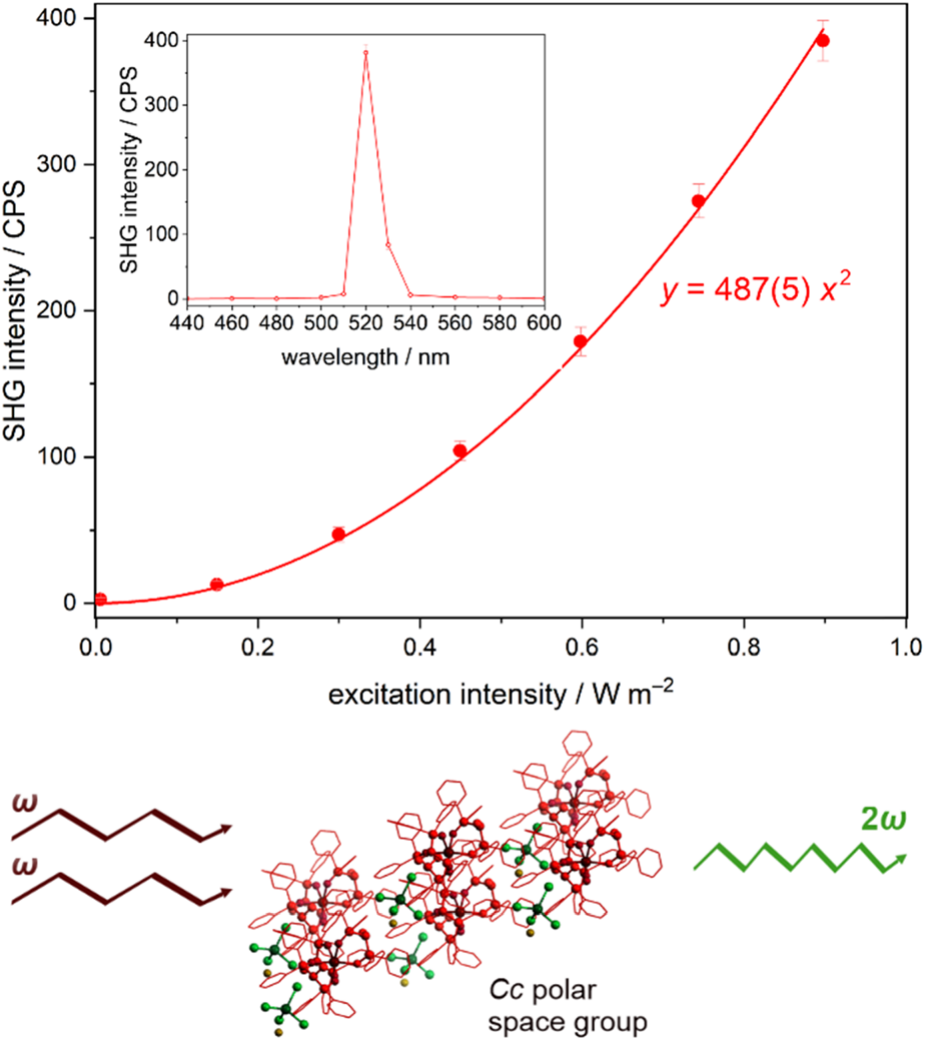
The room-temperature SHG intensity of the powder sample of 1 in the function of excitation intensity (top), the wavelength dependence of the SH signal for the incident power of 0.897 W m^−2^ for the 1040 nm laser (top, the inset), shown together with the schematic illustration of the SHG phenomenon generated in 1 (bottom). The colored circular points represent the experimental data. The solid line in the main part shows the best fit following the quadratic function with the indicated parameter (*R*^2^ = 0.99864) while the solid line in the inset is only to guide the eye.

Taking into account the non-centrosymmetric *Cc* space group, compound 1 was tested for the expected non-linear optical (NLO) activity employing the experiment of second harmonic generation (SHG). The SHG is related to the interactions of photons of the *ω* frequency with the crystalline material lacking an inversion center, which results in the frequency doubled (2*ω*) output light. Thus, the polycrystalline sample of 1 was irradiated by the 1040 nm pulse laser as incident fundamental light, and the second harmonic green light with the maximum at *ca.* 520 nm was found (Fig. 2, S11, and S12†). The intensity of SHG light was found to follow the quadratic dependence in the function of excitation light intensity which indicates a two-photon process characteristic of the SHG phenomenon. The SHG nature of the observed optical effect is also proven by the wavelength dependence of the output light showing the maximum at the double frequency to incident light. To evaluate quantitatively the SHG response of 1, the well-known SHG referential material of potassium dihydrogen phosphate (KDP) was measured using the analogous experimental conditions (Fig. S11 and S12†). From this experiment, the SHG light intensity of 1 can be estimated to reach *ca.* 0.8% of KDP, which is a rather small value, however, at the level observed for other families of non-centrosymmetric molecular materials based on metal complexes and organic species.^73–76^ Moreover, for comparison purposes, we also performed the SHG experiment for the reference compound 2 crystallizing in a centrosymmetric *P*2_1_/*c* space group. As expected, this material shows a negligibly weak SHG signal (Fig. S11†) which supports the conclusion that the relatively weak but non-negligible signal for 1 represents its polarity suggested by the SC-XRD data. Even with this rather weak SHG activity of 1, it was possible to examine the influence of relative humidity, *i.e.*, the dehydration process (see Water vapor sorption section below), on the efficiency of this non-linear optical process. To achieve this, we performed the *in situ* studies of the SHG effect upon the changes in relative humidity between 97% RH, through 50% RH, and 0% RH (realized by using the vacuum conditions, Fig. S13 and Table S9, with the detailed comment in the ESI†). It was found that the RH decrease from 97% to 50% leads to the enhancement of the SHG intensity by *ca.* 2.6% while the decrease of RH to 0% results in the total SHG intensity increase by *ca.* 7%. Thus, it can be stated that the related dehydration of the molecular material of 1 produces a subtle increase in the SHG activity by *ca.* 7%. However, this effect should be considered as a rather minor one as the relative experimental error was estimated to be of *ca.* 3.4–3.7%, thus the observed SHG enhancement by dehydration is only weakly stronger than the natural variation of the SHG intensity in the applied experimental setup.

### Piezoelectric and pyroelectric properties

**Fig. 3:**
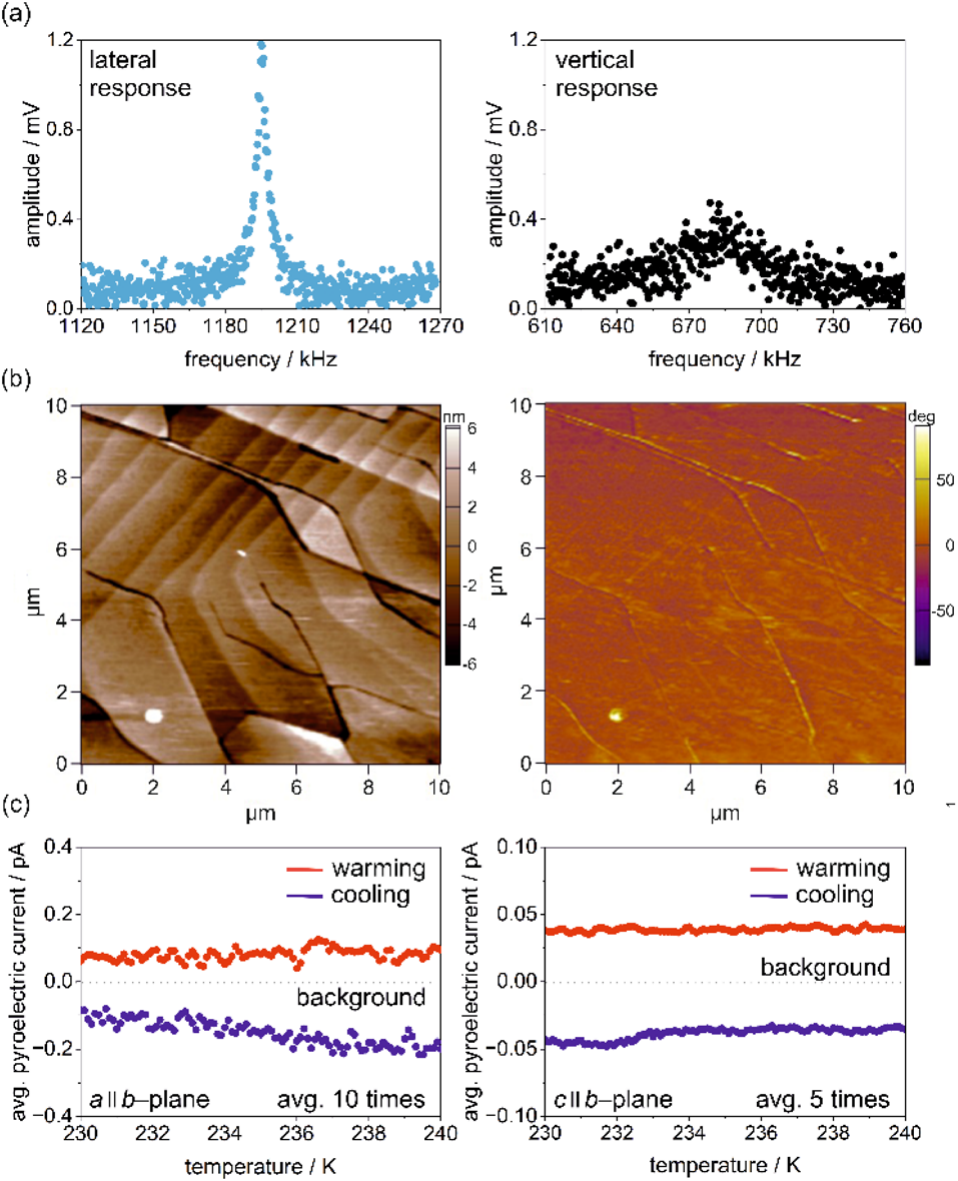
Representative results of piezoelectric force microscopy (PFM) and pyroelectric measurements for 1: lateral and vertical piezoelectric contact response curves (a), crystal topography and phase image of the (010) plane of a crystal (b), and average pyroelectric current, on the *a*-axis along the *b*-plane (c, left), and on the *c*-axis along the *b*-plane of the single crystal of 1 (c, right).

As further consequences of the polar *Cc* space group of 1 (*i.e.*, a polar point group *m*), one can expect the appearance of piezo-, pyro-, and even ferroelectric characteristics.^10,14,77–81^ To examine these possibilities, first, vertical and lateral piezoelectric force microscopy (PFM) measurements were performed on the *b*-plane of a selected single crystal (Fig. 3, see also Experimental section in the ESI† for more details). In general, PFM signals are associated with the inverse piezoelectric response, which is defined using the eqn (1):

1 *ε*_*jk*_ = *d*_*ijk*_*E*_*i*_ (*i*, *j*, *k* = 1, 2, 3)

where *E*_*i*_ is the electric field, *d*_*ijk*_ is the tensor coefficient, *ε*_*jk*_ is the strain tensor, and (*i*, *j*, *k*) represents three orthogonal axes, *x*_1_, *x*_2_, *x*_3_.^78,79^ For the point group *m*, the *x*_2_-axis is set to the *b*-axis. The vertical and lateral PFM measurements on the *b*-plane (*i.e.*, the electric field is applied to the *x*_2_- or *b*-axis *via* the cantilever) can detect a signal when *d*_222_ and *d*_2*jk*_ are finite, respectively. Before the scanning measurements, we checked the piezoelectric-response-induced resonance profile of the cantilever in contact with the *b*-plane (*i.e.*, the so-called contact resonance). We observed no clear contact resonance in the vertical PFM setup, while a sharp resonance is observed in the lateral PFM setup (Fig. 3a). These observations are consistent with what is expected for crystals belonging to the point group *m*, in which *d*_222_ is forbidden by symmetry (Neumann's principle) while *d*_223_ and *d*_212_ may be finite. The small and broad peak of the vertical PFM is attributed to the background signal of the cantilever response due to stray capacitance. Having observed finite response for the lateral PFM, we next performed the scanning measurements of the lateral PFM on the *b*-plane. The topography and PFM-phase images are shown in the left and right panels in Fig. 3b, respectively. We performed the lateral PFM scanning for several different positions but found no phase contrast (Fig. 3a, right), implying that the single crystal of 1 possesses nearly a single domain. This feature does not result in the conclusion on the ferroelectricity but implies that finite pyroelectric current may be observed upon temperature variations. Note that if the sample consists of many domains with opposite polarization, the macroscopic pyroelectric current would be close to zero as a result of the cancellation of pyroelectric currents with opposite signs. Pyroelectric currents were measured in two orthogonal directions (labeled as *a* and *c* directions) within the *b*-plane (Fig. 3c, see also Experimental section in the ESI, as well as Fig. S14 and S15†). The pyroelectric current, *I*_p_ is represented by the eqn (2):2
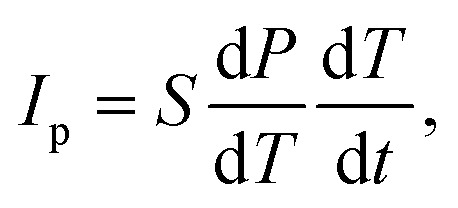
where *S* is the surface area of the electrode, *P* is the electric polarization, *T* represents the temperature, and *t* denotes time, respectively.^80,81^ The sign of the pyroelectric current *I*_P_ in eqn (2) depends on the direction of the temperature sweep, *i.e.*, d*T*/d*t*. Fig. 3c shows that the pyroelectric current is positive for warming runs and negative for cooling runs. The sign reversal depending on warming or cooling is a hallmark of pyroelectric currents, thus the molecular material of 1 can be undoubtedly assigned to the group of molecular pyroelectrics.

### Water vapor sorption

**Fig. 4:**
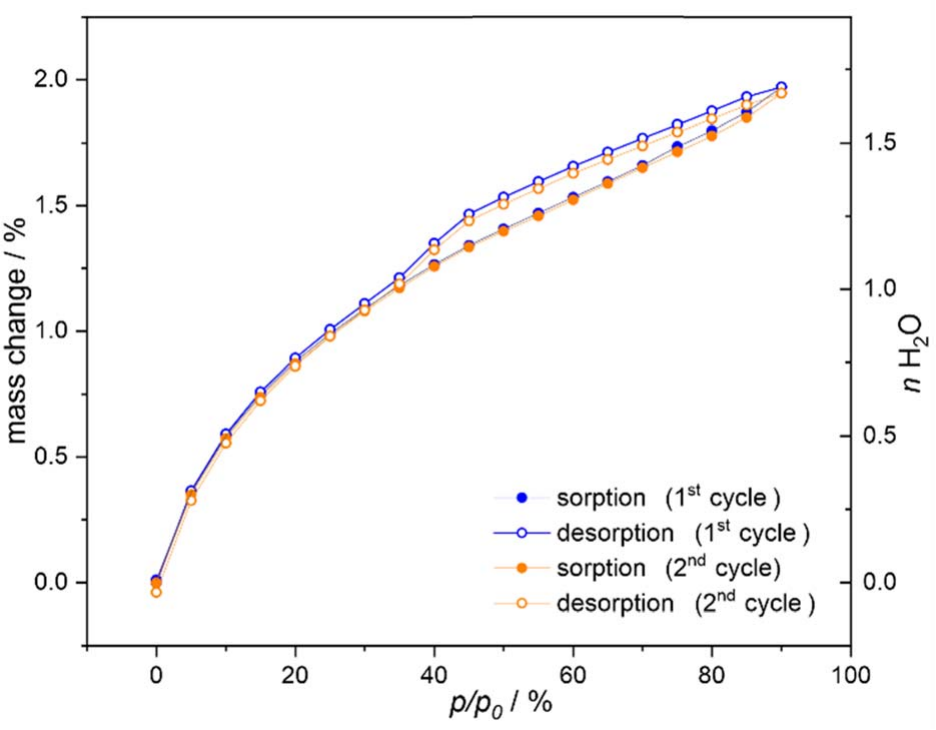
Water vapor sorption isotherms at 25 °C for compound 1, presented for two consecutive cycles of sorption and desorption. The scale on the right side indicates the approximate number of water molecules exchanged upon sorption/desorption.

As 1^100K^ was found to contain weakly bonded water molecules of crystallization that can be easily removed within the SC-XRD experiment at 300(2) K (see above), it was deduced that the reversible water vapor sorption property can be observed for this material. Thus, dehydration and rehydration processes for 1 were studied by the dynamic vapor sorption (DVS) gravimetric method at 25 °C with the relative humidity changing stepwise in the range between 0 and 90% with a 5% step. The resulting isotherms for two consecutive cycles of sorption and desorption are presented in Fig. 4. At these conditions, 1 does not form distinct stable phases with a plateau on the sorption isotherms. We can observe fast sorption of water vapors between 0% and *ca.* 35% of relative humidity (RH) which corresponds to nearly one water molecule per the formula unit, agreeing with the number of water molecules of crystallization found for the 1^100K^ phase within the SC-XRD experiment (Fig. 1). At higher RH, the sorption slows down and the next *ca.* 0.6 water molecules per the formula unit are adsorbed linearly up to the used limit of 90% of RH. For the desorption, we repeatedly observe the narrow hysteresis within the high-humidity region of 40–90% of RH. The width of this hysteresis in the context of the number of adsorbed is very narrow, *ca.* 0.1 water molecules per the formula unit. For lower RH, the sorption and desorption curves perfectly overlap. This behavior was found repeatable as the second cycle of sorption–desorption is identical to the first one.

Based on these water vapor sorption studies we can distinguish three phases of material 1, including (a) the dehydrated phase, 1^deh^, achievable for the sorption experiment at 0% of RH, of the composition of [Mn^II^(Me-dppmO_2_)_3_][Mn^II^Cl_4_] (named 1^deh^), which corresponds to the 1^deh,300K^ phase generated *in situ* within the SC-XRD experiment, (b) the hydrated phase, 1, achieved at *ca.* 35% of RH in the sorption experiment, of the composition of [Mn^II^(Me-dppmO_2_)_3_][Mn^II^Cl_4_]·H_2_O (1), which corresponds to the crystal structure of 1^100K^ obtained by taking the fresh crystal from the ambient laboratory conditions, and (c) the high-humidity phase (named 1^HH^) with the maximal achievable number of *ca.* 1.7 molecules per formula unit, obtained by conditioning the sample of 1 at 90% of RH. It is important to note here that the dehydrated and high-humidity phases of 1 are well-defined to be related to the 0% of RH and 90% of RH, respectively. On the contrary, the hydrated phase of 1 was for the sorption properties defined to be related to *ca.* 35% of RH; however, we generally assign the hydrated phase to those corresponding to the fresh crystalline material of 1, air-dried under laboratory conditions, which are 20–40% of RH. To be precise in the interpretation of experimental data, if the RH conditions were different from the 35% of RH, this was stated in the discussion (*e.g.*, in the magnetic studies, see below).

### Solid-state photoluminescence

**Fig. 5:**
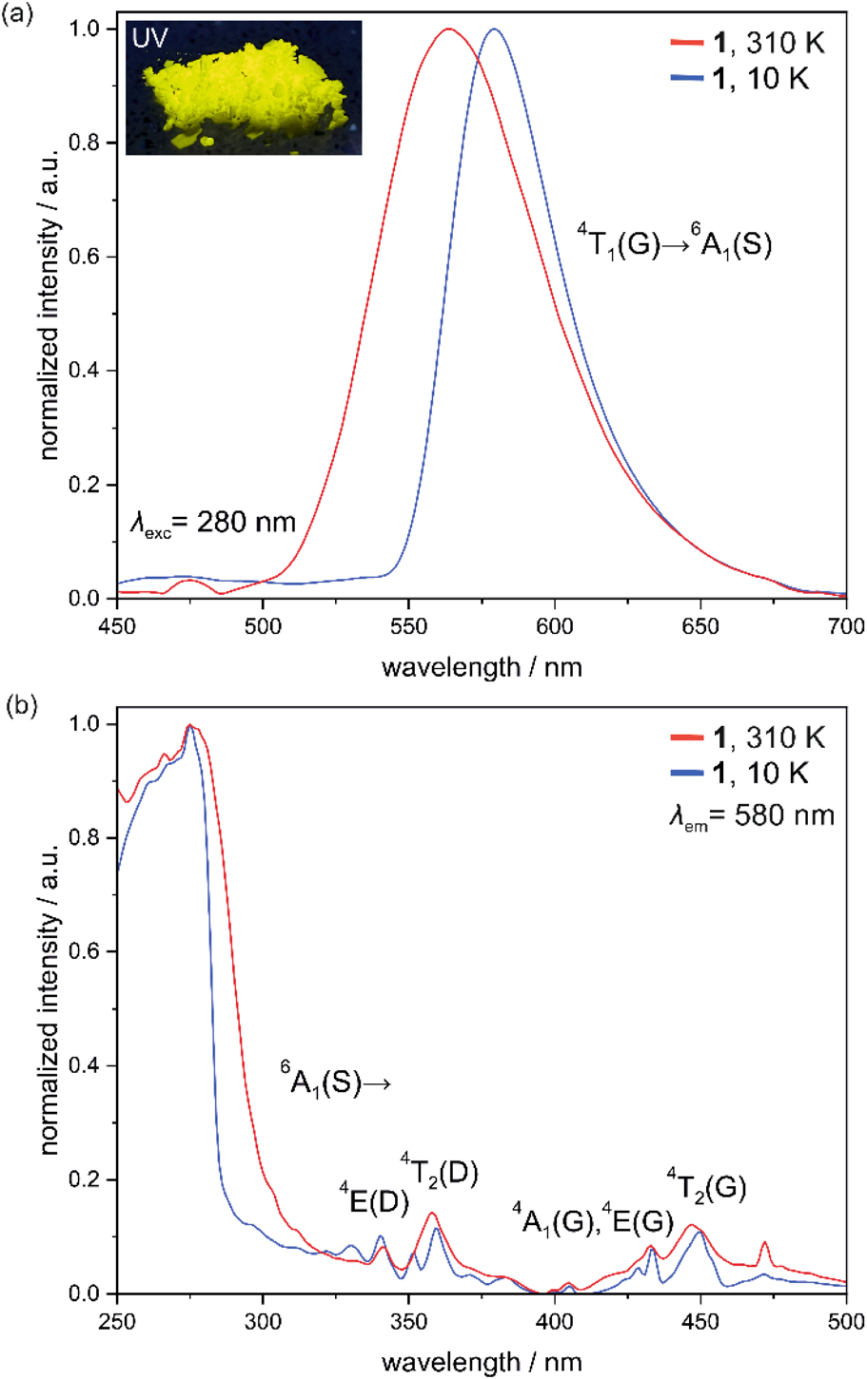
Solid-state photoluminescent properties of 1 at 310 K and 10 K, including the emission spectra for the most efficient 280 nm excitation (a) and the excitation spectra for the monitored emission at 580 nm (b). The selected d–d electronic transitions corresponding to the properties of six-coordinated Mn1 centers of 1 were indicated in both parts of the figure. In the inset of (a), the photo of the polycrystalline sample of 1 under UV light (365 nm) irradiation was presented.

As Mn(ii) complexes placed in a weak ligand-field environment can reveal rich visible photoluminescence,^57–62^ the optical properties of 1, as well as the related organic precursors, were thoroughly examined (Fig. 5, S16–S31 and Tables S10–S13†). Here, the results for the hydrated form of 1 are discussed while the next section shows the effect of humidity on the photo-luminescence of this material. Solid-state UV-vis absorption spectra show that 1 exhibits strong UV absorption in the range from 200 to 300 nm, assignable to spin-allowed n → π* and π → π* organic-ligand-based electronic transitions as they are also detectable in the similar range for free Me-dppmO_2_ molecules (Fig. S16†).^67^ On the contrary, the sharper weaker absorption peaks related to the Mn(ii)-centered d–d electronic transitions, which are expected to cover the broad UV-to-vis range, are hardly visible in our experimental setup. This is not surprising as they are strongly forbidden by selection rules.^57–62^

Therefore, searching for luminescent properties, first, we focused on the strong UV absorption below 300 nm. Under the excitation of 1 by such the deep UV light at room temperature, yellow photoluminescence was detected (Fig. 5a). At 310 K the emission spectra, under the UV excitation of 280 nm, contain a single broad band with a maximum positioned at 560 nm. It can be assigned to the d–d ^4^T_1_(G) → ^6^A_1_(S) electronic transition of Mn1 complexes of a distorted octahedral geometry rather than the tetrahedral Mn2 sites which are expected to show the emission at higher energies, as shown, *e.g.*, in the mentioned work of A. S. Berezin *et al.* on the analogous salt of [Mn^II^(dppmO_2_)_3_][Mn^II^Cl_4_].^57–62,67^ The other related materials incorporating [Mn^II^Cl_4_]^2−^ ions also indicate that these molecular luminophores are expected to reveal the higher-energy PL, usually centered around 520–530 nm.^82,83^ On the other hand, the observed emission maximum is of noticeably higher energy than typically detected for octahedral Mn(ii) complexes in the O-based coordination environment, including previously reported [Mn^II^(dppmO_2_)_3_]^2+^ complexes revealing PL with the room-temperature maximum at *ca.* 610 nm.^57–62,67,84–88^ This can be related to the high degree of distortion from an octahedral geometry, which was confirmed by the CShM analysis (Table S8†). Such deformation can weaken the ligand field provided by the Me-dppmO_2_ ligands. This correlates well with the elongation of Mn–O distances when compared 1 with the reference compound 2 (Tables S2 and S7†). Under the unchanged excitation conditions, upon cooling to 10 K, the emission band in 1 becomes narrower and red-shifted to the maximum at 580 nm which is a typical trend for PL of octahedral Mn(ii) complexes (Fig. 5a).^57–62,67,84–88^ The thermal changes in the emission spectrum of 1 are gradual as depicted by the set of *T*-dependent spectra gathered in the 10–310 K (Fig. S19†). This results in the gradual cooling-induced color change of PL from yellow to orange, which corresponds to the changes in the *xy* CIE 1931 parameters from 0.445, 0.538 at 310 K to 0.528, 0.457 at 10 K (Fig. S22 and Table S11†).

It is worth discussing here more about the lack of PL from tetrahedral [Mn^II^Cl_4_]^2−^ complexes in the room-temperature emission spectrum of 1 (Fig. 5a). As mentioned above, it was expected to reveal the maximum around 520–530 nm but, using the 280 nm excitation (corresponding mainly to the organic-ligand-centered excited states, see below), it is not detected even at very low temperatures (Fig. 5a and S19†). There is a tiny additional emission peak with a maximum at *ca.* 475 nm which can be noticed in the series of high-temperature (in the region of *ca.* 200–310 K) emission spectra of 1. However, it is positioned at too high energy and was attributed to the artifact occurring often in the emission spectra using the applied experimental setup. Upon cooling, it becomes negligibly small when compared with the main emission band due to the large cooling-induced enhancement of the latter (Fig. S19†). The above-discussed emission features were gathered for the 280 nm excitation which was selected as an optimal one (see the discussion on excitation spectra below, Fig. 5b). Thus, to search for additional emission bands, we also examined other excitation wavelengths. Among them, we precisely examine PL of 1 upon the 360 nm excitation assignable to the direct excitation of d–d Mn(ii) electronic transitions, namely ^6^A_1_(S) → ^4^T_2_ (D) (Fig. S20†).^57–62,67,84–88^ At 310 K, the related emission spectrum is almost identical to those found for the 280 nm excitation. After collecting the emission spectra for this excitation at variable temperatures (Fig. S19†), we also noticed the identical thermal changes as found for the 280 nm excitation (Fig. S22 and Table S11†). The small difference appears only below 50 K (especially for the spectrum at the lowest accessible temperature of 10 K, Fig. S20†) when the tiny emission peak appears at *ca.* 535 nm. It can be ascribed to the residual PL from [Mn^II^Cl_4_]^2−^ ions as postulated also for the analogous [Mn^II^(dppmO_2_)_3_][Mn^II^Cl_4_] salt.^67^ This suggests that the most energy absorbed by these Mn(ii) (Mn2) centers is rather transferred to the octahedral Mn(ii) (Mn1) complexes showing the emissive state of lower energy. This is expected to be achievable in 1 due to the alignment of Mn(ii) complexes with the shortest Mn1–Mn2 distances of *ca.* 8.5 Å.^67,89^

The excitation spectra of 1 for the monitored emission at 580 nm contain, both at 310 K and 10 K, the main broad high-energy maximum centered at *ca.* 280 nm (Fig. 5b). This excitation, used for the main PL characteristics, can be ascribed to Me-dppmO_2_-ligand-based excited states with the eventual admixture of ligand-to-metal charge transfer (LMCT) states (see below the further comment on the role of an organic ligand in the PL properties of 1).^57–62,67,82,83^ Moreover, weaker yet distinct and sharp bands at 340 nm and 357 nm are observed. They can be ascribed to ^6^A_1_(S) → ^4^E(D) and ^6^A_1_(S) → ^4^T_2_(D) d–d electronic transitions of Mn1 complexes, respectively. Similarly, further relatively sharp emission peaks at 433 nm and 447 nm are related to the analogous ^6^A_1_(S) → [^4^A_1_(G), ^4^E(G)] and ^6^A_1_(S) → ^4^T_2_(G) transitions.^84–86^ These findings agree with the interpretation that the observed PL in 1 originates from Mn(ii) complexes. Except for the intensity increase, the excitation spectra do not reveal significant changes in their temperature dependencies gathered in the 10–310 K range (Fig. S18†).

The emission decay profiles of 1 were found to be mono-exponential, providing the best-fit emission lifetime at 310 K of 4.27(3) ms (*λ*_exc_ = 280 nm, *λ*_em_ = 580 nm, Fig. S27 and Table S12†). Upon cooling, this lifetime gradually increases up to 7.51(1) ms at 10 K (Fig. S24–S27 and Table S12†). These values lie within the range expected for phosphorescence originating from spin-forbidden d–d electronic transitions of Mn(ii) complexes of deformed octahedral geometry.^57–62,67,82–88^ With the change of the excitation to 360 nm, the emission lifetimes are very similar changing from 4.39(2) ms at 310 K to 7.56(2) ms at 10 K (Fig. S28–S31 and Table S12†). Note that in all the *T*-variable experiments presented in this section, the powder sample of 1 was protected from dehydration, thus the effect of dehydration and the influence of relative humidity are excluded from consideration. The absolute emission quantum yield exceeds 33% at room temperature (Table S13†).

**Fig. 6:**
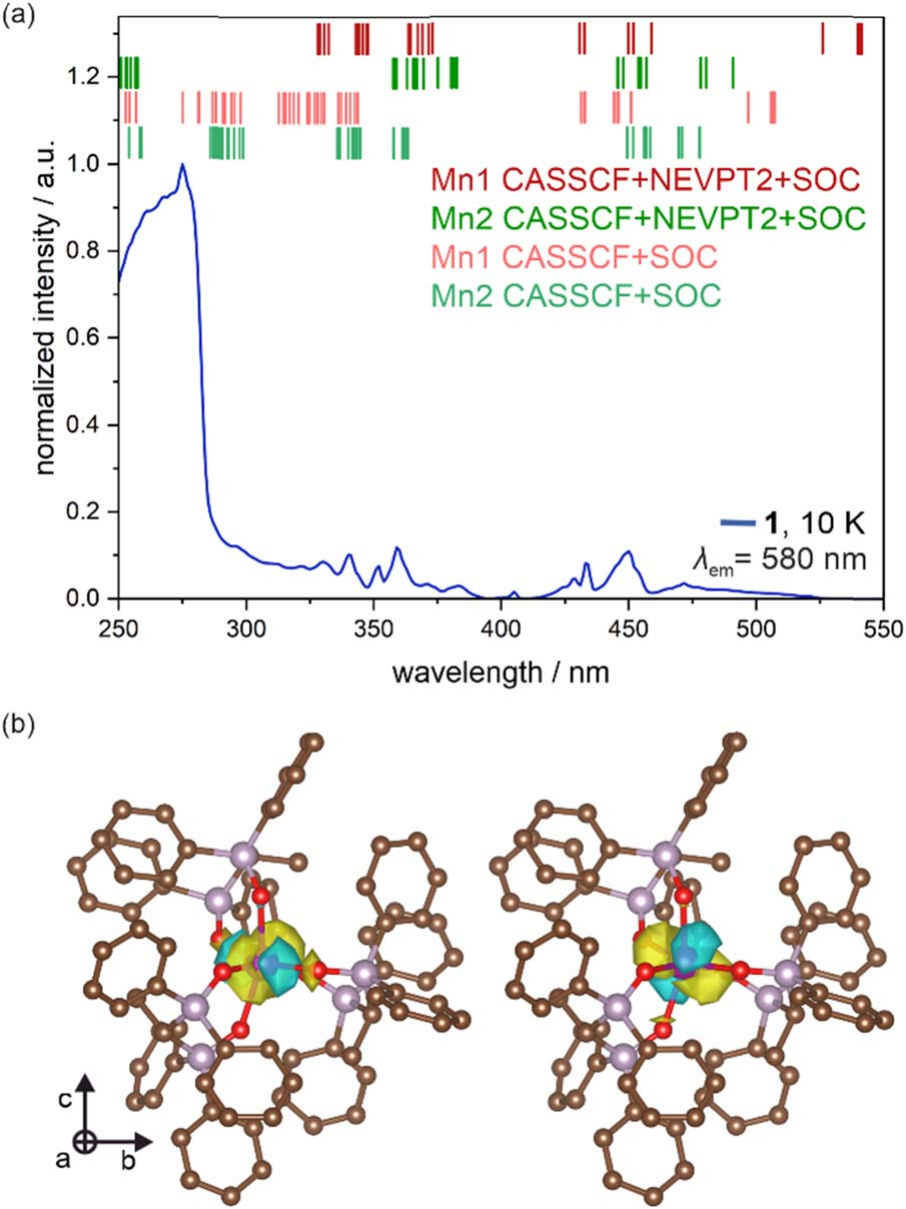
Representative results of the *ab initio* calculations performed for Mn(ii) complexes of 1 (*i.e.*, octahedral Mn1 and tetrahedral Mn2), including the energy splitting schemes calculated by the indicated methods and presented on the experimental excitation spectrum of 1 for the monitored emission of 580 nm corresponding to the ^4^T_1_(G) → ^6^A_1_(S) electronic transition (a), and the computed largest contribution (>30%) to the lowest energy electronic transition in 1 (found for Mn1), visualized by the 3d orbitals of used active space for the ground level (left) and the first excited level (right). The visualization is provided on the ground state geometry with the isosurface level of 0.022 for the 3d orbitals (see Fig. S32† for the whole active space for Mn1 centers, and the text for details). In (a), vertical bars represent the calculated energies of d–d excited states. For the CASSCF + SOC theory level, besides the ground sextet state, states of both quartet and doublet multiplicities were considered and the scaling factor was 8000 cm^−1^ (Table S17, and the details in the main and the ESI†). For the CASSCF + NEVPT2 + SOC theory level, besides the ground sextet state, only the states of quartet multiplicity were considered and the scaling factor was 5000 cm^−1^ (Table S20, and the details in the main text and ESI†). In (b), the visualized optical transition was calculated using the CASSCF + NEVPT2 theory level with only the states of sextet and quartet multiplicities considered (Table S18†).

The electronic transitions responsible for the PL properties of 1 were examined by the *ab initio* calculations, employing the ORCA 5.0.4 quantum chemistry package.^90,91^ We used the *ab initio* approach of an SA-CASSCF (*i.e.*, state-averaged complete active space self-consistent field) type as it is known to be the most reliable tool for calculations of energy splitting schemes related to metal-centered excited states.^92,93^ They are crucial for 1 as, except for the broad deep UV absorption and further excitation bands assignable to the organic ligand, the critical optical characteristics, including the observed visible-light emission and the set of excitation peaks above 300 nm, are of purely metal-centered origin (Fig. 5). In this methodology (for its technical details, see the comment in the ESI†), we considered two metal complexes of 1, [Mn^II^(Me-dppmO_2_)_3_]^2+^ (Mn1) and [Mn^II^Cl_4_]^2−^ (Mn2), separately, and their models for calculations were taken directly from the crystal structure obtained by the SC-XRD experiment (Fig. 1), without geometry optimization as typical for *ab initio* methods. For both complexes, the applied active space was composed of 5 electrons and five 3d orbitals of Mn(ii) (Fig. S32 and S33†). We optimized the combination of 1 sextet (the ground state of high-spin Mn(ii)), 24 quartet, and 75 doublet states, originating from various possible configurations within the d-shell. In some cases, we limited the calculations to only sextet and quartet states due to the computational limitations (for large Mn1 complexes) or to show useful comparisons. We started with the SA-CASSCF approach only with sextet and quartet states (Fig. S35a and Table S14†). The resulting energy-splitting schemes indicate that the lowest energy d–d transitions are of lower energy for Mn1 (*ca.* 20 032 cm^−1^, *i.e.*, *ca.* 499 nm) than for Mn2 (*ca.* 21 369 cm^−1^, *i.e.*, *ca.* 468 nm) which agrees with the expected lower energy absorption and emission transitions for octahedral Mn2 complexes and the observed lower energy PL for Mn1 sites (Fig. 5 and S20†).^57–62,67^ Using this first method, the matching of the calculated energy splitting scheme to the excitation spectrum of 1 appears to be relatively good as the series of peaks in the 320–360 nm range as well as those in the 420–470 nm region find the reflection in the calculated ones. However, from this view, the peaks were hard to assign to Mn1 or Mn2 centers giving the possible optical transitions in similar wavelength ranges, except for the well-distinguished lowest-energy ones for the Mn1 site (Fig. S35a†). It is worth noting that the calculated d–d electronic transitions are of the absorption character but they cannot be compared with the absorption spectrum (Fig. S16†) which was not sensitive to weak peaks of spin-forbidden d–d transitions. Thus, they are compared with the excitation spectrum of 1 where the sharp peaks of d–d transitions are detectable (Fig. 5 and 6a). The second notice should be given to the scaling procedure which was performed for the calculated energy splitting schemes based on the well-investigated absorption/excitation peak of the ^6^A_1_(S) → ^4^E(D) electronic transition for [Mn^II^Cl_4_]^2−^ (in 1, Mn2) complexes at 360 nm.^67,82,83^ In the next step, we performed the SA-CASSCF calculations with also doublet states involved which resulted in the richer energy splitting scheme in the UV-vis range (Fig. S35b and Table S15†). However, the previously computed peaks were only slightly shifted while the new ones cover only the higher-energy UV range of 250–320 nm. This agrees with the typical trend for high-spin Mn(ii) revealing the dominance of the sextet-to-quartet transitions above 320 nm (Fig. 5).^57–62,67^ Next, the calculated states were mixed with spin–orbit coupling (SOC). The resulting energy schemes of transitions between SO states are shown in Fig. S35c and Table S16 (only quartets), Fig. 6a, S35d, and Table S17† (quartets and doublets). The SOC effect results in the additional splitting for all previously computed peaks without significant energy shifts. This result correlates well with the excitation spectrum of 1 which consists of several closely positioned peaks assignable now to the SOC-induced energy splitting. Therefore, from the CASSCF + SOC method with quartets and doublets involved, the conclusion on the lower energy of lowest-lying optical transitions for Mn1 than for Mn2 centers can be undoubtedly drawn.

To further correct the computed energy schemes, we added the NEVPT2 correction,^94^ using the CASSCF + NEVPT2 and CASSCF + NEVPT2 + SOC methods with versions of only quartets as well as quartets + doublets (the latter achievable only for the smaller Mn2 complex). The related results are provided in Fig. 6a, S35e–h, and Tables S18–S21.† The NEVPT2 correction results in noticeable energy shifts for optical transitions. It can be again concluded that the doublets' inclusion gives additional peaks only below 330 nm where they are experimentally represented by weak peaks visible onto the broad component assignable to the organic ligand which cannot be taken into account in the *ab initio* approach. The crucial quartet-related excited states (Fig. 6a, top part) are distinctly responsible for the series of excitation peaks above 330 nm. The peaks computed for Mn1 complexes are sufficient to describe all the most pronounced excitation peaks confirming their assignment to selected d–d transitions of these metal centers (Fig. 5b). The overlap with peaks ascribable to Mn2 sites can exist as they are partially situated within similar wavelength ranges. However, the peaks computed for Mn2 centers provide much worse overlap with the experiment suggesting that at least the most intense excitation bands are assignable to Mn1 which agrees with the discussion of experimental results presented above. Moreover, this most precise level of calculations confirms the presence of lowest-energy optical transitions for Mn1 centers. They are present in the 530–550 nm range (Tables S20 and S21†), thus they are not visible in the excitation spectrum as it is too close in comparison to the monitored broad emission band. However, taking into account the expected Stokes shift of *ca.* 50 nm,^57–62,67^ the computed lowest-energy excited state of Mn1 is well placed to be assigned as the presumable source of the observed main emission of 1. We found that the largest (*i.e.*, >30%) contribution to this lowest-energy electronic transition in 1 is related to the transition between two d-orbitals visualized in Fig. 6b. Thus, such a metal-centered d–d transition provides the absorption at *ca.* 540 nm and can have the crucial role in the detected emission at *ca.* 580 nm (Fig. 5a). We were not able to optimize the geometry of Mn1 complexes in this excited state due to the computational limitations; thus, the precise analysis of the emissive transition could not be performed but its d–d character is indicated by the above discussion. Moreover, by all types of *ab initio* methods, Mn2 centers reveal the lowest-lying excited states of higher energies than Mn1 (Tables S14–S21†). Thus, the related emission is also postulated to be of higher energy for Mn1 than for Mn2. This agrees well with the previous reports as well as the residual PL in 1 at *ca.* 535 nm at 10 K which was assigned to Mn2 (Fig. S20b†). For smaller Mn2 complexes, we were able to optimize the geometry of the first lowest-lying excited state at the CASSCF-NEVPT2 level of theory in the gas phase utilizing numerical gradients (Fig. S34†). The subsequent calculations involving the mixing of the obtained states with SOC yield the final energies gathered in Table S22.† The resulting emissive transition was found at *ca.* 413 nm which is much lower than the detected maximum at *ca.* 535 nm. However, this value was given without the scaling procedure that should be done as was the case of absorption transitions. Taking the correction of *ca.* 5000 cm^−1^ as established for NEVPT2-involved methods, the scaled emission wavelength is *ca.* 520 nm which is close to the observed one confirming the assignment of this emission to Mn2. Thus, the set of used *ab initio* methods well describes the lowest-energy d–d electronic transitions of Mn1 and Mn2 complexes responsible for the main yellow PL of 1 and the residual PL at *ca.* 535 nm, respectively. The *ab initio*-calculated d–d excited states well describe also the excitation pattern above 330 nm. They cannot explain the origin of the broad excitation band below 300 nm as it is ascribable to the Me-dppmO_2_ ligand whose orbitals could not be taken into account in the *ab initio* method. The elucidation of the ligand's contribution to these absorption/excitation might be done using the DFT approach but our related attempts were not successful due to too large size of Mn1 complexes. However, these absorption/excitation routes are proven experimentally, *e.g.*, by the comparison with the free Me-dppmO_2_ molecules revealing the strong light absorption bands below 300 nm of almost identical shape as the absorption bands in 1 (Fig. S16†).

One additional notice regarding the performed *ab initio* calculations should be devoted to the hydrogen atoms and their eventual role in the accuracy of the obtained results. The hydrogen atoms are present in the structure of Mn1 complexes and they were found within the SC-XRD analysis where a riding model for their refinement was used (see Experimental section for details). Thus, for comparison, we also optimized their positions separately using a ZORA-B3LYP level of theory using “Tight” convergence criteria for wavefunction and gradients. The resulting structural model of Mn1 sites, named Mn1^opt^, was found to reveal noticeably longer C–H bond lengths when compared with those found in the SC-XRD experiment (Fig. S66 and Table S34†). Then, we performed the analogous *ab initio* calculations for the Mn1^opt^ sites as was done for Mn1 models from the SC-XRD analysis. The resulting energy differences between the analogous computed states were found to be very small, up to *ca.* 10 cm^−1^ (below 0.04%) and up to *ca.* 25 cm^−1^ (below 0.11%) for the CASSCF-/CASSCF-SOC-type calculations and their further modifications with the NEVPT-2 correction, respectively (Tables S35–S38†). This proves that the uncertainty existing on the positions of H-atoms cannot affect the accuracy of presented computational results.

In this context, the role of the Me-dppmO_2_ ligand in the optical properties of 1 should be discussed. As mentioned above, the main and strong light absorption of the ligand is positioned below 300 nm and can be assigned to the spin-allowed electronic transitions. Almost the identical shape of the absorption band in this region was found for 1, thus it can be assigned to Me-dppmO_2_-centered electronic transitions (Fig. S16†). The LMCT transitions can be observed for Mn(ii)-based luminophores; however, they do not contribute significantly to the detected light absorption in 1 or they are situated at higher energies.^57–62,67,82,83^ We measured the PL properties of free Me-dppmO_2_ molecules together with the reference dppmO_2_ (Fig. S17, S21 and Table S10†). At room temperature, under the deep UV excitation of 280 nm, corresponding to the mentioned strong absorption detectable also for dppmO_2_ molecules (Fig. S16†), both investigated organic species reveal the UV emission centered at 330 and 340 nm, for Me-dppmO_2_ and dppmO_2_, respectively, with only the weak component in the vis-range up to *ca.* 400 nm (Fig. S17†). This PL can be assigned to a fluorescent character as the typical Stokes shift of *ca.* 50 nm in relation to the strong absorption band is observed. Under the same excitation conditions but at 77 K, only such fluorescence is still detectable for dppmO_2_. On the other hand, Me-dppmO_2_ reveals then the second emission band which is the broadband blue PL centered at 475 nm. It can be assigned to the phosphorescence induced thanks to the decreased temperature promoting the intersystem crossing (ISC) (Fig. S17c and d†). This emission can be observed at room temperature but using higher excitation wavelengths above 310 nm producing the easily observable PL of a blue color (Fig. S17d, S21 and Table S10†). The related emission band is broad and its exact pattern depends on temperature and excitation conditions leading to the subtle PL color variation oscillating around the blue color towards white or green (Fig. S21†). Such emission can be also induced for dppmO_2_ using the excitation above 290 nm; however, it is of noticeably higher energy (maximum at 430 nm) and much weaker. Thus, only very weak blue PL is visible for dppmO_2_ (Fig. S17b, S21 and Table S10†). The observed phosphorescence of Me-dppmO_2_ covers the relatively broad range of the visible spectrum but is of distinctly higher energy than the emission of 1 (Fig. S23†), *i.e.*, the yellow-to-orange PL of 1 while blue-to-greenish-blue phosphorescence for Me-dppmO_2_ with the UV-positioned fluorescence. Thus, it can be easily noticed that the PL bands of the ligand disappear upon its coordination by Mn(ii) centers in 1. It can be postulated that the deep UV excitation of, *e.g.*, 280 nm (Fig. 5), leads mainly to the population of the Me-dppmO_2_ singlet excited state, which is followed by the ISC facilitated by the heavy atom effect, and then the energy transfer to the excited states of Mn1 sites, resulting in their PL, is observed. Such a scenario agrees with the broad ligand-based excitation band of 1 at 280 nm. On the contrary, the direct excitation of the Me-dppmO_2_ triplet state is not effective as depicted by the presence of only the sharp excitation peaks assignable to d–d excited states of Mn(ii) centers in 1 above 300 nm. This is not surprising as the absorption to the ligand's triplet state is expected to be very weak. The indicated sensitization of Mn(ii)-centered PL by Me-dppmO_2_ is mainly realized on Mn1 centers that coordinate this organic ligand. It is much less possible for Mn2 sites which are surrounded only by chlorido ligands and placed at much longer distances to Me-dppmO_2_ molecules. Even if the organic-ligand-to-Mn2 energy transfer at least partially occurs, the further Mn2-to-Mn1 energy transfer can also operate. Therefore, the PL of Mn2 complexes is not promoted in 1 as depicted by its lack under the ligand-directed excitation of 280 nm, and their residual observation only at very low temperatures under direct d–d excitation (Fig. S20†).

### Humidity-dependent solid-state photoluminescence

**Fig. 7:**
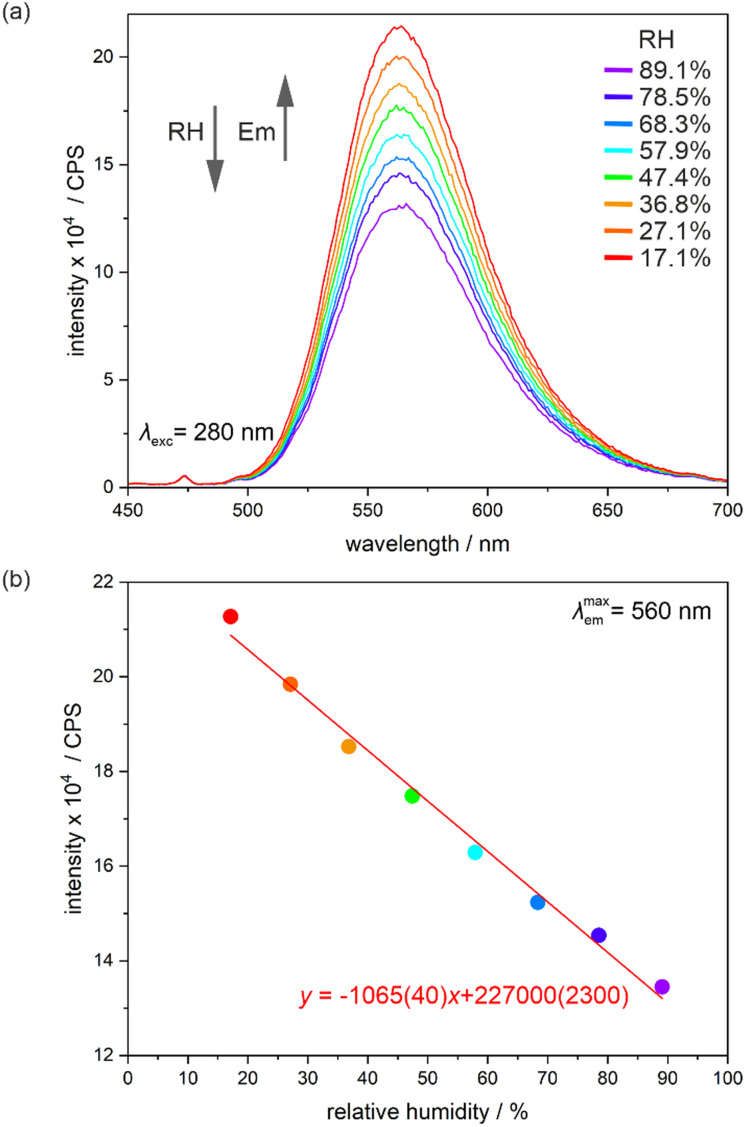
Relative-humidity-dependent emission spectra of 1 under the 280 nm excitation, gathered in the 17–90% relative humidity (RH) range at the indicated RH values at room temperature (a) and the resulting RH-dependence of the emission intensity at the peak maximum (b), shown with the linear fit of the presented parameters (*R*^2^ = 0.9914).

As 1 combines water vapor sorption property and photo-luminescence, the humidity dependence of light emission features was investigated. The related experiments were performed using the homemade experimental setup at room temperature in the accessible *ca.* 17–90% range of relative humidity (RH) for two consecutive cycles of water vapor sorption/desorption using the powder sample of 1 (see Experimental section in the ESI and Scheme S1† for technical details, while Fig. 7, S36–S40 and Tables S23–S27† for the resulting optical data). Upon increasing the RH, the emission signal remains in a similar band shape but its intensity gradually decreases (Fig. 7a). This effect can be ascribed to the increasing number of O–H vibrations which quenches the Mn(ii)-based emission through the energy overlap between their overtones and the emissive electronic transitions. On the other hand, water molecules of crystallization, detected in the crystal structure of 1 (Fig. 1), were found not to be involved in significant hydrogen bonds, thus, their presence does not contribute to the increase of the stiffness of a supramolecular framework which often leads to the increase of the emission signal upon the increased RH. This is related to the applied Me-dppmO_2_ ligands which possess the methyl groups that block the acidic C–H donor sites present between the phosphine groups in an original dppmO_2_ ligand.^67^

The humidity sensing ability of 1 was tested by plotting emission intensity *versus* RH (Fig. 7b and S36†). This dependence was found to be repeatedly, *i.e.*, upon sorption/desorption cycles, represented by the linear equation as depicted by the plot for the first desorption (Fig. 7b) which gives the best-fit equation of Emission intensity (CPS) = −1065(40)·RH + 227 000(2300), *R*^2^ = 0.9914. The analogous dependences were found for the other sorption processes (Fig. S36 and Table S23†). Such the linear dependence between the emission intensity and humidity agrees generally with the water vapor sorption curve (Fig. 4) which shows rather smooth changes of the mass upon the increased RH. The detected narrow hysteresis for the high RH values does not affect the sensing abilities of the material.

Besides the emission intensity, we also found that the emission lifetime significantly depends on the RH changes (Fig. S37–S40 and Tables S24–S27†). The emission decays for *λ*_exc_ = 280 nm and *λ*_em_ = 560 nm were gathered in the range of 20–87% RH for one cycle of water vapor sorption/desorption. They were found to be mono-exponential in the whole RH range. The best-fit curves reveal the RH-dependent changes between 4.193(12) ms at 20.6% RH to 3.148(9) ms at 87.0% RH for sorption and 3.148(9) ms at 87.0% RH to 4.249(11) ms at 20.7% RH for desorption. This indicates that the RH increases lead to a decrease in the emission lifetime which agrees well with the interpretation including the critical role of the RH-induced increased number of emission-quenching O–H vibrations. Plotting emission lifetime against RH provides the dependencies that can be satisfactorily described by the linear fitting with the equations of Emission lifetime (ms) = −0.0173(13)·RH + 4.53(7), *R*^2^ = 0.9842 for sorption and Emission lifetime (ms) = −0.0162(13)·RH + 4.45(8), *R*^2^ = 0.9813 for desorption (Fig. S39†). However, due to the slight deviation from the linear fit in the range of 30–70% RH, we decided to perform an alternative allometric fitting producing the equations of Emission lifetime (ms) = 7.83(19)·RH^−0.202(7)^, *R*^2^ = 0.9926 for sorption and Emission lifetime (ms) = 8.31(28)·RH^−0.217(9)^, *R*^2^ = 0.9883 for desorption (Fig. S40†). Thus, the obtained material is applicable for the RH optical sensing using both the emission intensity as well as the emission lifetime, and the sensing response is provided for the broad RH ranges of *ca.* 17–90%.

### Humidity-dependent dielectric response

**Fig. 8:**
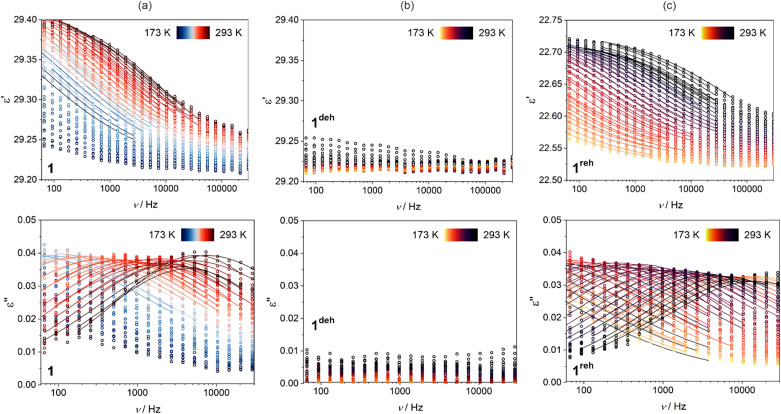
Frequency dependences of the in-phase (real, *ε*′, top parts) and out-of-phase (imaginary, *ε*′′, bottom parts) components of electrical permittivity at various temperatures from the indicated ranges for the as-synthesized phase of 1 (a), the dehydrated phase of 1^deh^ (b), and the subsequently re-hydrated phase of 1^reh^ (c). The presented dielectric characteristics were gathered for the cooling mode with the 5 K step (the 1st cycle of cooling–heating, see Fig. S41–S44†). The colored circle points represent the experimental data while the solid lines show the best-fit curves to the generalized Debye model for a single relaxation process (eqn (3)).

**Fig. 9:**
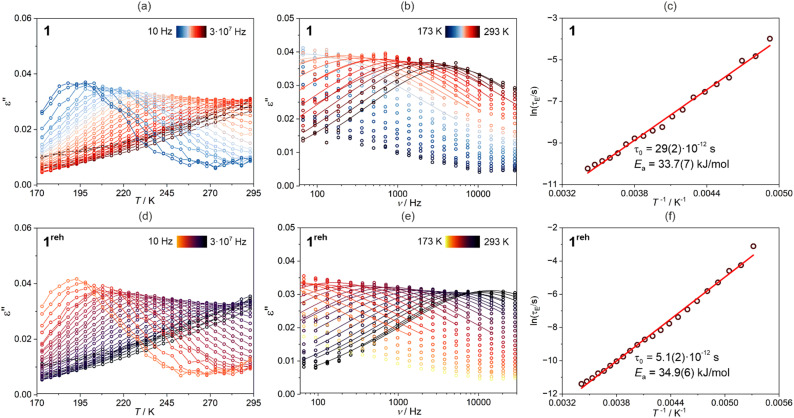
Temperature-variable dielectric characteristics of 1 (a–c) and 1^reh^ (d–f), gathered for the cooling mode with the 5 K step (the 2nd cycle of cooling–heating, see Fig. S41–S44†), including the temperature dependences of the imaginary part of electrical permittivity (*ε*′′) at various field frequencies from the indicated range (a and d), the related frequency dependences of *ε*′′ at various temperatures from the indicated range (b and e), shown together with the best-fit curves to the generalized Debye model for a single relaxation process (eqn (3)), and the resulting dielectric relaxation times in the function of temperature (c and f), shown together with the best-fit curve to the Arrhenius-type process (eqn (4)). The related best-fit parameters are given on the graphs (c and f).

As 1 contains weakly bonded water molecules of crystallization of high polarity, we decided to examine its dielectric properties and their correlation with the dehydration of the material. Therefore, the variable-temperature frequency-dependent dielectric response, represented by the complex electrical permittivity (*ε*), was investigated for the pressed disc of the polycrystalline sample of 1 in the broad frequency range from 50 to 3 × 10^7^ Hz (see Experimental section for all technical details, Fig. 8, 9, and S41–S48†). First, to avoid dehydration, the dielectric studies were performed in the limited 173–293 K range (sample 1, Fig. 8a, 9, S41, and S42†). The detected values of the real part of electrical permittivity, *ε*′, lie in the range of 29.2–29.4 while the related imaginary part, *ε*′′, is placed in the range of 0.007–0.045 for the investigated frequency range (Fig. 8a). More importantly, the relaxor-type behavior was found,^95^ as the *ε*′(*ν*) dependences reveal the gradual decrease with increasing frequency while *ε*′′(*ν*) curves contain the maxima. Upon cooling, these maxima are gradually shifted to lower frequencies which leads to their transition outside the accessible frequency window below *ca.* 215 K. Such the temperature variation of dielectric response was found well repeatable as was tested by three consecutive cycles of cooling and heating in the 293–173 K range (Fig. S41 and S42†).

After examining the hydrated phase, the sample of 1 was heated to 353 K to achieve complete dehydration, and then the dielectric characteristics of the sample of the dehydrated phase, 1^deh^, both upon cooling and heating, were examined (Fig. 8b and S42†). Within the broad investigated temperature range of 173–353 K, the *ε*′ values remain at a similar level of 29.20–29.25 as was found in 1 but there is no distinct temperature variation on the *ε*′(*ν*) dependences. As a result, there are no maxima on the *ε*′′(*ν*) curves, and the related signal of *ε*′′ is very small, below 0.01 for all frequencies and temperatures. This indicates that the dielectric relaxation processes disappear upon the removal of water molecules from the structure of 1.

To further prove the direct correlation between the hydration state of 1 and its dielectric properties, we re-hydrate the sample by its exposition to the ambient laboratory humidity (see Experimental section for details). The resulting re-hydrated sample, 1^reh^, was then investigated (Fig. 8c, 9, S43, and S44†). It exhibits dielectric relaxation processes, almost identical to those found for the as-synthesized hydrated sample of 1 indicating that these electrical characteristics are fully related to the presence of water molecules in the structure. We even further dehydrate the sample of 1^reh^ inducing again the disappearance of the dielectric relaxation effect (Fig. S44†). Thus, the hydration–dehydration process in 1 can be used for ON/OFF switching of dielectric relaxation easily detectable in the 215–293 K range. To quantitatively discuss the dielectric relaxation in 1, and further in 1^reh^, the *ε*′(*ν*) and *ε*′′(*ν*) dependences were fitted using the generalized Debye model for a single dielectric relaxation, expressed by the eqn (3):^96^3
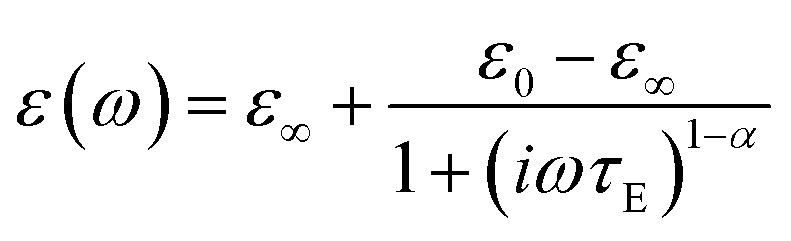
where *ε*(*ω*) is a complex electrical permittivity containing both the real and imaginary parts, *ε* = *ε*′+*iε*′′, and *ω* = 2π*ν*. The resulting best-fit curves, which are shown in Fig. 8, 9, and S41–S44,† well reproduce the experimental data in the broad temperature ranges. The critical parameter, determined from each of the fits, is the dielectric relaxation time, *τ*_E_, which was further plotted in the form of ln(*τ*_E_) against the inverse temperature (Fig. 9 and S41–S44†). The resulting temperature dependences of dielectric relaxation time were found to follow the linear trend for all the investigated samples, 1 and 1^reh^, and heating–cooling cycles, which indicates the Arrhenius-type relaxation process given by the eqn (4):4
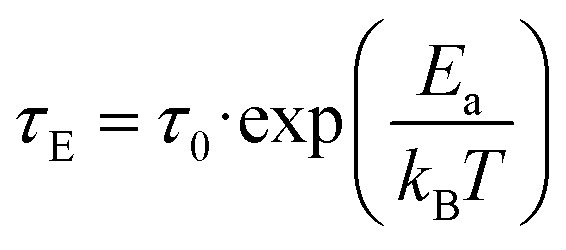
where *E*_a_ stands for the activation energy for macroscopic dielectric relaxation.^97–99^ The best-fit values of *E*_a_ in 1 oscillate within the narrow range of 33.5–37.1 kJ mol^−1^ for all heating–cooling cycles, while 1^reh^ shows a similar range of 34.9–39.9 kJ mol^−1^ taking into account all analogously performed cycles of heating and cooling (Table S28†). The pre-exponential factors, *τ*_0_, are also similar for 1 and 1^reh^, and within the cycles of heating–cooling, being in the range of 10^−11^–10^−12^ s. The obtained activation energy is typical for the dielectric relaxation process associated with the Bjerrum-type orientation defects that can appear in crystalline solids filled with water molecules.^100–105^ On the other hand, the order-disorder phase transitions, which can be a source of thermal dielectric relaxations and could have been observed due to the presence of tetrahedral Mn(ii) complexes,^106,107^ are rather excluded as they are not visible in the variable-temperature structural studies (see above) and the DSC peaks do not appear in the related temperature range (see Fig. S59 with the comment in the ESI†). This further supports the direct correlation between the generation of dielectric relaxation in 1 and the presence of weakly bonded water molecules in its crystal structure. The less obvious question remains if the observed dielectric relaxations are somehow affected by the polar *Cc* space group of 1. To examine this aspect, we investigated the dielectric characteristics of the reference material built of dppmO_2_ ligand, namely [Mn^II^(dppmO_2_)_3_][Mn^II^Cl_4_]·2H_2_O (2), which crystallizes in the centrosymmetric *P*2_1_/*c* group (see structural studies above and Experimental section in the ESI for details†). We found that similarly to 1 the hydrated form of 2 exhibits relaxor-type dielectric relaxations below room temperature (Fig. S45 and S46†). These relaxation processes disappear upon dehydration and could be recovered by the rehydration process (Fig. S47 and S48†). This indicates that a polarity of 1 does not have a direct influence on the dielectric characteristics of the material, supporting the interpretation of the key role of incorporated water molecules. However, there are distinct differences in dielectric properties of 1 and 2 as the latter reveals much higher values of activation energies determined for the observed Arrhenius-type relaxation effect (Fig. S41–S48 and Table S28†). For 2, the *E*_a_ value is placed within the range of 58.2–64.6 kJ mol^−1^. This can be correlated with the stronger binding of water molecules in 2 by the hydrogen bonds involving acidic protons of dppmO_2_ ligands which do not appear in 1 due to the presence of methyl groups replacing these acidic protons. This indicates the surprisingly crucial role of this small organic substituent in the dielectric properties of the investigated hybrid material.

### Magnetic properties and their modulation with humidity changes

**Fig. 10:**
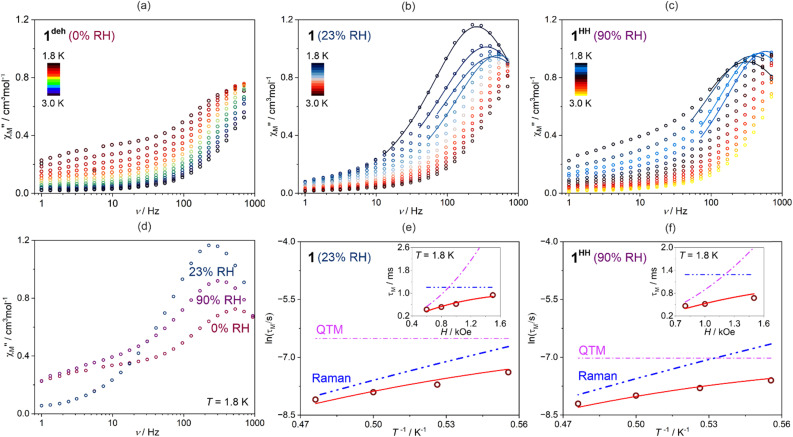
Representative alternate-current (ac) magnetic characteristics of 1^deh^ (1 at RH = 0%), 1 (RH = 23%), and 1^HH^ (1 at RH = 90%), including the frequency dependences of the out-of-phase magnetic susceptibility, 
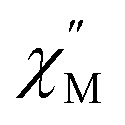
, of 1^deh^ (a), 1 (b), and 1^HH^ (c) at indicated temperatures under the dc field of 1 kOe, comparison of the selected 
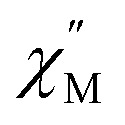
(*ν*) curves at *T* = 1.8 K for three indicated RH conditions (d), and the temperature- and field-dependences of magnetic relaxation time, *τ*_M_ for 1 (e) and 1^HH^ (f). Colored points in (a–d) parts represent the experimental data while the solid lines (b and c) show the best-fit curves to the generalized Debye model for a single relaxation process (eqn (5)). The empty circle points in (e) and (f) represent the resulting experimental relaxation times, while the red solid lines show the best-fit curves taking into account two indicated relaxation processes (eqn (6), the simultaneous fit of the *τ*_M_(*H*, *T*) dependences). The contributions of the indicated relaxation processes to the overall relaxation are depicted by dashed colored lines. For the whole set of ac magnetic characteristics, please see Fig. S50–S58.†

As 1 contains high-spin Mn(ii) centers embedded in a humidity-sensitive supramolecular framework, it is expected that the RH changes might affect its magnetism, including presumable spin–lattice relaxation.^54,55^ To examine this, we performed a series of both direct-current (dc) and alternate-current (ac) magnetic measurements for the as-synthesized sample of 1 (prepared under the ambient laboratory conditions), the dehydrated phase of 1^deh^, and the sample of 1 stabilized at high humidity (RH = 90%, 1^HH^, see Experimental section in the ESI for technical details, Fig. 10, S49–S58 and Tables S29, S30†). The room-temperature *χ*_M_*T* values are 8.75, 8.80, and 8.69 cm^3^ mol^−1^ K, for 1, 1^deh^, and 1^HH^, respectively, which are close to the theoretical limit of 8.75 cm^3^ mol^−1^ K for two free Mn(ii) centers of *S* = 5/2, *g* = 2.0. The *T*-dependence of *χ*_M_*T* measured at *H*_dc_ = 1 kOe shows almost no changes in the whole 1.8–300 K range (Fig. S49†), indicating that Mn(ii) centers are well magnetically isolated in all investigated phases of 1 as well as their magnetic anisotropy, correlated with spin–orbit coupling and crystal-field effects, is very weak. Field dependences of molar magnetization (*M*) at *T* = 1.8 K show the signal's increase with increasing field to 10*μ*_B_ at 70 kOe for all samples, which lies in the typical range for two uncoupled paramagnetic Mn(ii) centers (Fig. S49,† the insets).

The ac magnetic characteristics for 1, 1^deh^, and 1^HH^ do not demonstrate the noticeable signal of the out-of-phase magnetic susceptibility, 
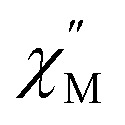
, under zero dc field, indicating the lack of field-free slow relaxation of magnetization which agrees well with the expected very weak anisotropy of Mn(ii) complexes. However, under the applied dc magnetic field, the distinct signal on the 
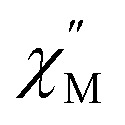
(*ν*) dependences appears below *ca.* 3 K. The optimal dc field was estimated, based on the dc-field-variable 
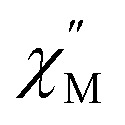
(*ν*) plots, to be 1 kOe for all phases (Fig. S50, S53, and S56†). For this dc field, the hydrated phases, both 1 and 1^HH^, exhibit distinct maxima on the 
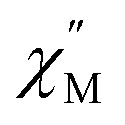
(*ν*) dependences indicating the appearance of field-induced slow magnetic relaxation, while 1^deh^ reveals only the onset of the related 
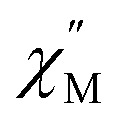
(*ν*) peaks (except the lowest accessible temperature, Fig. 10a–c). This indicates that the dehydrated phase shows the fastest magnetic relaxation but, interestingly, the slowest relaxation rate, represented by the shift of the 
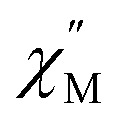
(*ν*) peak to the lowest frequency range is demonstrated by the sample of 1 conditioned at 23% RH (ambient laboratory conditions), thus the trend of the influence of RH on the magnetic relaxation is not-trivial (Fig. 10d). To discuss this quantitatively, both the field- and temperature-variable ac magnetic characteristics for phases showing the distinct maxima on the 
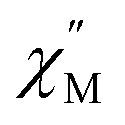
(*ν*) plots (*i.e.*, 1 and 1^HH^) were fitted using a generalized Debye model for a single magnetic relaxation process, expressed by the eqn (5),^96^ using the relACs software:^108^5
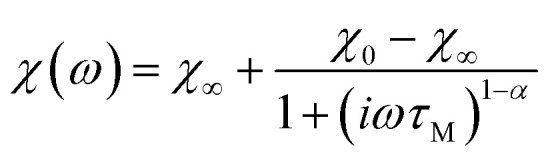
where *χ*(*ω*) is a complex magnetic susceptibility containing both the real and imaginary parts, *χ* = *χ*′+*iχ*′′, and *ω* = 2π*ν*. The best-fit curves (Fig. 10b, c, S51, S52, S57, and S58) enabled the extraction of magnetic relaxation times, *τ*_M_, only for the lowest temperatures below 2.1 K and limited ranges of dc field below 1.6 kOe. Despite this, it was found achievable, both for 1 and 1^HH^, to analyze the *τ*_M_(*T*, *H*) using the 3-D simultaneous fitting procedure implemented in relACs,^108^ taking into account two relaxation processes represented by the eqn (6):6
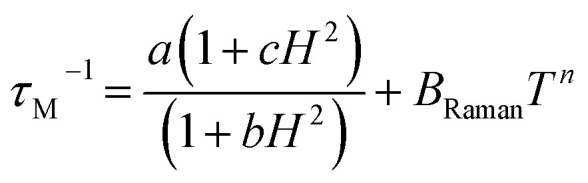
where the first term represents the quantum tunneling of magnetization (QTM) depicted by three parameters (*a*, *b*, and *c*), while the second term corresponds to the phonon-assisted Raman relaxation with the parameters of *B*_Raman_ and *n*.

The best-fit curves (Fig. 10e and f) provide the following set of parameters: for 1, *a* = 4.27 × 10^11^ s^−1^, *b* = 636.42 Oe^−2^, *c* = 0, *B*_Raman_ = 6.29 s^−1^ K^−*n*^, *n* = 8.30, for 1^HH^, *a* = 3.16 × 10^11^ s^−1^, *b* = 281.84 Oe^−2^, *c* = 0, *B*_Raman_ = 5.11 s^−1^ K^−*n*^, *n* = 8.55. In general, within our multiple attempts at fitting the *τ*_M_(*T*, *H*), we tested not only the above-mentioned Raman and QTM contributions to the observed slow magnetic relaxation but also other typically considered for molecular nanomagnets, including an Orbach process with the Arrhenius-type thermal activation providing the parameter of the energy barrier between two energy states responsible for this relaxation, a Direct process with the *A*_direct_*H*^*m*^*T* (see eqn (7) below) term, as well as a phonon-bottleneck effect which shows a similar dependence on temperature to the Raman relaxation but with the *n* parameter close to 2.^109–113^ The first from this set, *i.e.*, the Orbach relaxation, could be excluded due to the very weak intrinsic magnetic anisotropy of Mn(ii) complexes in 1 which was depicted by the dc magnetic data (see above) and the performed *ab initio* calculations showing negligibly small zero-field splitting effect for the ground sextet states of both Mn1 and Mn2 complexes (Tables S16, S17, S20, and S21†). The other two processes, *i.e.*, a direct process and a phonon-bottleneck effect, were considered. However, their usage instead of the Raman process did not lead to the effective fitting of the obtained ac magnetic data while their addition as the co-existing process led to the over-parameterization issue. Thus, the slow magnetic relaxation in both 1 and 1^HH^ appears to be well-described by the QTM and Raman relaxation processes with the dominant role of the latter as depicted by their visualized contributions on the ln(*τ*_M_) *versus T*^−1^ dependencies (Fig. 10e and f). In fact, the above-mentioned set of QTM, Raman, direct, and phonon-bottleneck processes was found to govern the dc-field-induced slow magnetic relaxation reported for a few examples of Mn(ii) complexes,^55,114–117^ or other similar very weakly anisotropic metal centers, such as Gd(iii).^118^ Such slow magnetic relaxation phenomena under the applied dc field were shown to occur for basically isotropic Mn(ii) centers embedded in five-, six-, and seven-coordinated complexes of distorted geometries, including the deformed octahedral ones.^55,114,115^ It was found that the distortion can lead to non-negligible energy splitting of the ground state under the dc field contributing to the rise of the slow magnetic relaxation. Taking these findings into account, it can be postulated that the observed magnetic relaxation effect is attributable to the Mn1 complexes as they are distinctly distorted from an ideal octahedral geometry (Table S8†). This statement is further confirmed by the lack of distinct slow magnetic relaxation characteristics in the accessible frequency range for compound 2, which contains six-coordinated Mn(ii) complexes of the geometry close to a perfect octahedron. Moreover, it was found that the variable distances between Mn(ii)-based molecular species in a crystal lattice as well as the variable amount of solvent molecules of crystallization can affect their magnetic relaxation by influencing the dipolar interactions between spin centers and/or amending the phonon modes which are crucial in mediating the spin–lattice relaxation processes (Raman, direct, and phonon-bottleneck effects).^54,55,114–117^

In this context, the experimental data for both 1 and 1^HH^ indicate the dominant role of Raman relaxation, which is directly related to the spin–phonon interaction.^109–112^ Therefore, the differences in magnetic relaxation effects within the investigated phases of 1 have to be discussed in terms of the changes in energies and accessibility of the phonon modes that are crucial in magnetic relaxation. Going from 1 to 1^HH^, the power *n* of Raman relaxation slightly increases from 8.30 to 8.55. This can be associated with lowering the energies of crucial phonon modes due to the higher content of water molecules leading to the stronger hydrogen bonding network and stiffening of the supramolecular framework. However, on the other hand, the *B*_Raman_ parameter decreases indicating that the high humidity phase has a lower number of accessible phonon modes. The first effect is probably the dominant one as visualized by the distinctly stronger temperature dependence of relaxation times for the high humidity phase (Fig. 10e and f). These trends would have suggested that 1^deh^ should have demonstrated the slowest relaxation; however, for this phase, the relaxation is the fastest one. This might be attributed to the increasing role of magnetic dipole-magnetic dipole interactions that occur more easily after dehydration and facilitate the overall relaxation rate. This explanation can be supported by the second, low-frequency relaxation process, related to such dipolar interactions, which appears at the lowest dc fields only for the dehydrated phase. A more precise discussion is here difficult due to the impossibility of determining the relaxation rates for 1^deh^. Nevertheless, it can be clearly stated that the humidity level governs the magnetic relaxation in 1 with the non-trivial trend including the primary slowdown and further acceleration of magnetic relaxation upon the increased relative humidity (Fig. 10d).

In addition, it should be noted that the high dc magnetic field of, *e.g.*, 5 kOe, induces another relaxation process, especially in 1 and 1^deh^ (Fig. S52, S55 and S58†). This relaxation pathway is different as it can be analyzed using a single type of relaxation, *i.e.*, the Direct process depicted by the eqn (7):

7 *τ*_M_^−1^ = *A*_direct_*H*^*m*^*T*

The best-fit parameters are *A*_direct_ = 5.57 s^−1^ K^−1^, *m* = 0 for 1, and *A*_direct_ = 6.33 s^−1^ K^−1^, *m* = 0.65 for 1^deh^ which demonstrates also the sensitivity to the relative humidity at this level of magnetic relaxation characteristics.

For additional confirmation of the origin of the magnetic relaxation of 1, the isostructural analog [Mn^II^(Me-dppmO_2_)_3_] [Zn^II^Cl_4_] (4), along with the only-Zn(ii)-containing compound 3 of the different composition of [Zn^II^(Me-dppmO_2_)Cl_2_]·0.5H_2_O (3), were synthesized and characterized (Fig. S60–S65 and Tables S31–S33, with related comments in the ESI†). As strongly suggested by the structural and dc magnetic data, the mentioned Mn(ii)–Zn(ii) mixed-metal compound 4 was found to contain the octahedral [Mn^II^(Me-dppmO_2_)_3_]^2+^ complexes, identical to those present in 1 with the tetrahedral sites occupied by diamagnetic [Zn^II^Cl_4_]^2−^ complexes. This reference compound, for the sample conditioned at the selected RH of 28%, reveals the distinct slow magnetic relaxation effect under the external dc magnetic field (Fig. S63 and S64†). Moreover, similarly to 1, this magnetic relaxation is driven by QTM and Raman processes (Table S33†). In the case of 4, due to the lack of tetrahedral [Mn^II^Cl_4_]^2−^ complexes, these magnetic features can be undoubtedly assigned to the octahedral [Mn^II^(Me-dppmO_2_)_3_]^2+^ complexes. Therefore, these additional results for compound 4 strongly support the assignment of the humidity-dependent slow magnetic relaxation effects in 1 to the octahedral Mn1 sites as was proposed in the discussion above.

## Conclusions

We report a simple synthetic route to a unique polar molecular material demonstrating broad multifunctionality, including non-linear optical activity and pyroelectricity, as well as a series of other physical properties, including luminescent, dielectric, and magnetic ones, that are responsive to humidity variation. This molecular material explores the well-known magnetic and luminescent functionalities of weak-ligand-field manganese(ii) complexes which originate from their high-spin d^5^ configuration ensuring the high spin on the ground state and efficient emission from d–d electronic transitions. We demonstrate that this intrinsic functional potential of Mn(ii) complexes can be greatly enriched by a simple methyl-functionalization of the attached bis(diphenylphosphino)methane dioxide ligand which plays a crucial role in a series of events, including (a) generation of crystal's polarity as proven by the non-linear optical (NLO) activity and pyroelectricity, (b) generation of efficient room-temperature water vapor sorption property, which further results in (c) humidity control over the photoluminescence intensity and lifetime. Moreover, we found the easily detectable dielectric relaxations occurring around room temperature and below due to the presence of weakly bonded water molecules which results in the dehydration–hydration-induced ON/OFF switching of such the dielectric response. The role of the functionalized ligand appeared also in this context as the activation energy for dielectric relaxation was found to be greatly decreased when compared with the reference material with the unsubstituted ligand providing too strong bonding of water molecules. Last but not least, the humidity level was found to affect the slow magnetic relaxation effect occurring mainly through the spin–phonon interactions influenced by the water content in the non-trivial way as both too high and too low humidity facilitates the faster magnetic relaxation. Therefore, our approach proves that earth-abundant Mn metal can be used for the preparation of stimuli-responsive molecular material exhibiting an extensive set of physical functionalities as well as the remarkable triple luminescent–dielectric–magnetic response to relative humidity. This work provides a proof-of-concept example that does not finalize the further enrichment and improvement of the proposed research line. First, the observed SHG activity was found to be very weak but even with the obtained related signal, it was possible to detect a weak influence of relative humidity also on this physical parameter. Thus, it is worth searching for the relative materials with enhanced SHG activity that might also reveal a more pronounced humidity switching of non-linear optical effects.^119^ Moreover, the chirality might be conveniently induced by the proper functionalization of the used diphosphine-dioxide-type ligand, and then chiroptical properties are expected, such as circularly polarized luminescence (CPL) that was suggested to be a promising tool for the next generation of highly stimuli-responsive molecular materials.^120^ We are currently working on the realization of these concepts.

## Data availability

The data supporting this article have been included as part of the ESI.† Crystallographic data for MedppmO_2_, 1^100K^, 1^270K^, 1^deh,300K^, 1^deh,330K^, 1^deh,100K^, 2, 3, and 4 has been deposited at the CCDC database under 2328045, 2328046, 2328047, 2328049, 2328048, 2411622, 2328050, 2431595, and 2431594 numbers. The other data can be obtained from the corresponding author.

## Author contributions

A. H.: conceptualization (equal), investigation: (lead; syntheses, structural studies, basic physicochemical characterization, luminescence, RH-dependent optical, dielectric, and magnetic studies; contribution also to water vapor sorption), data curation (lead), formal analysis, methodology, validation (equal; contributions to all parts of the work), visualization, writing – original draft (lead), writing – review & editing (supporting). M. Z.: conceptualization, investigation, data curation, formal analysis, methodology, software, resources, validation (supporting; the main contribution to the part related to theoretical calculations), writing – review & editing (supporting). J. W.: conceptualization, investigation, data curation, formal analysis, methodology, validation (supporting; the main contribution to the part related to SHG activity), writing – review & editing (supporting). K. M.: investigation, data curation, formal analysis, methodology, validation, visualization (supporting; the main contribution to the part related to piezo- and pyroelectricity), writing – review & editing (supporting). F. K.: conceptualization, investigation, data curation, formal analysis, methodology, validation, software, resources, visualization (supporting; the main contribution to the part related to piezo- and pyroelectricity), supervision (supporting; coordination of international collaboration), writing – original draft and writing – review & editing (supporting). J. R.: investigation, data curation, formal analysis, methodology, validation, supervision (supporting; contribution to the part related to structural studies, basic physicochemical characterization, and luminescence), writing – review & editing (supporting). M. H.: conceptualization, investigation, data curation, formal analysis, methodology, validation (supporting; contribution to the part related to water vapor sorption), writing – review & editing (supporting). S. B.: conceptualization, investigation, data curation, formal analysis, methodology, validation, supervision, resources (supporting; contribution to the part related to the preparation and investigation of organic precursors), writing – review & editing (supporting). H. T.: conceptualization, formal analysis, methodology, software, resources, supervision, validation (supporting; contribution to the part related to SHG activity), supervision: supporting (coordination of international collaboration), writing – review & editing (supporting). S. O.: conceptualization, formal analysis, methodology, software, resources, validation (supporting; contribution to the part related to SHG activity), supervision: supporting (coordination of international collaboration), writing – review & editing (supporting). S. C.: conceptualization (equal), investigation, data curation, methodology (supporting; syntheses, structural studies, basic physicochemical characterization, photoluminescence, RH-dependent optical, dielectric, and magnetic studies, water vapor sorption), formal analysis, validation, visualization (supporting; contributions to all parts of the manuscript), funding acquisition, project administration, software, resources, supervision (lead), writing – original draft (supporting), writing – review & editing (lead).

## Conflicts of interest

There are no conflicts of interest to declare.

## Supplementary Material

SC-016-D5SC00404G-s001

SC-016-D5SC00404G-s002

## References

[cit1] Quan L. N., Rand B. P., Friend R. H., Mhaisalkar S. G., Lee T.-W., Sargent E. H. (2019). Chem. Rev..

[cit2] Bellani S., Bartolotta A., Agresti A., Calogero G., Grancini G., Di Carlo A., Kymakis E., Bonaccorso F. (2021). Chem. Soc. Rev..

[cit3] Allendorf M. D., Dong R., Feng X., Kaskel S., Matoga D., Stavila V. (2020). Chem. Rev..

[cit4] Du K., Feng J., Gao X., Zhang H. (2022). Light Sci. Appl..

[cit5] Fop S. (2021). J. Mater. Chem. A.

[cit6] Liu J., Hesjedal T. (2023). Adv. Mater..

[cit7] Spaldin N. A., Ramesh R. (2019). Nat. Mater..

[cit8] Yee D. W., Lifson M. L., Edwards B. W., Greer J. R. (2019). Adv. Mater..

[cit9] Lee G.-H., Moon H., Kim H., Lee G. H., Kwon W., Yoo S., Myung D., Yun S. H., Bao Z., Hahn S. K. (2020). Nat. Rev. Mater..

[cit10] Guo P.-H., Liu J.-L., Jia J.-H., Wang J., Guo F.-S., Chen Y.-C., Lin W.-Q., Leng J.-D., Bao D.-H., Zhang X.-D., Luo J.-H., Tong M.-L. (2013). Chem.–Eur. J..

[cit11] Estrader M., Salinas Uber J., Barrios L. A., Garcia J., Lloyd-Williams P., Roubeau O., Teat S. J., Aromí G. (2017). Angew. Chem., Int. Ed..

[cit12] Solomos M. A., Claire F. J., Kempa T. J. (2019). J. Mater. Chem. A.

[cit13] Marin R., Brunet G., Murugesu M. (2021). Angew. Chem., Int. Ed..

[cit14] Jankowski R., Wyczesany M., Chorazy S. (2023). Chem. Commun..

[cit15] Miller R. G., Brooker S. (2016). Chem. Sci..

[cit16] Kobayashi A., Imada S., Shigeta Y., Nagao Y., Yoshida M., Kato M. (2019). J. Mater. Chem. C.

[cit17] Ni Z.-P., Liu J.-L., Hogue M. N., Liu W., Li J.-Y., Chen Y.-C., Tong M.-L. (2017). Coord. Chem. Rev..

[cit18] Wang J., Zakrzewski J. J., Zychowicz M., Vieru V., Chibotaru L. F., Nakabayashi K., Chorazy S., Ohkoshi S. (2021). Chem. Sci..

[cit19] Resines-Urien E., Garcia-Tunin M. A. G., Garcia-Hernandez M., Rodriguez-Velamazan J. A., Espinoza A., Sanchez Costa J. (2022). Adv. Sci..

[cit20] Boukheddaden K., Ritti M. H., Bouchez G., Sy M., Dirtu M. M., Parlier M., Linares J., Garcia Y. (2018). J. Phys. Chem. C.

[cit21] Huang Y., Pathak A. K., Tsai J.-Y., Rumsey C., Ivill M., Kramer N., Hu Y., Trebbin M., Yan Q., Ren S. (2023). Nat. Commun..

[cit22] Huang X.-D., Wen G.-H., Bao S.-S., Jia J.-G., Zheng L.-M. (2021). Chem. Sci..

[cit23] Ohkoshi S., Nakagawa K., Imoto K., Tokoro H., Shibata Y., Okamoto K., Miyamoto Y., Komine M., Yoshikiyo M., Namai A. (2020). Nat. Chem..

[cit24] Sang Y., Han J., Zhao T., Duan P., Liu M. (2020). Adv. Mater..

[cit25] Kulachenkov N., Haar Q., Shipilovskikh S., Yankin A., Pierson J.-F., Nomine A., Milichko V. A. (2022). Adv. Funct. Mater..

[cit26] Zhao Y., Li D. (2020). J. Mater. Chem. C.

[cit27] Rabelo R., Toma L., Moliner N., Julve M., Lloret F., Inclan M., Garcia-Espana E., Pasan J., Ruiz-Garcia R., Cano J. (2023). Chem. Sci..

[cit28] Erbas-Cakmak S., Leigh D. A., McTernan C. T., Nussbaumer A. L. (2015). Chem. Rev..

[cit29] Xin Y., Wang J., Zychowicz M., Zakrzewski J. J., Nakabayashi K., Sieklucka B., Chorazy S., Ohkoshi S. (2019). J. Am. Chem. Soc..

[cit30] Ohkoshi S., Namai A., Tokoro H. (2019). Coord. Chem. Rev..

[cit31] Chorazy S., Zakrzewski J. J., Reczyński M., Nakabayashi K., Ohkoshi S., Sieklucka B. (2019). J. Mater. Chem. C.

[cit32] Luo Y.-H., Dong H., Ma S.-H., Zeng F.-L., Jin X.-T., Liu M. (2023). J. Mater. Chem. A.

[cit33] Chen L., Ye J.-W., Wang H.-P., Pan M., Yin S.-Y., Wei Z.-W., Zhang L.-Y., Wu K., Fan Y.-N., Su C.-Y. (2017). Nat. Commun..

[cit34] Lan R., Gao Y., Shen C., Huang R., Bao J., Zhang Z., Wang Q., Zhang L., Yang H. (2021). Adv. Funct. Mater..

[cit35] Gao L., Baryshnikov G. V., Ali A., Kuklin A., Qian C., Zhang X., Chen F., Yi T., Wu H. (2024). Angew. Chem., Int. Ed..

[cit36] Nandi S., Singh H. D., Shekhar P., Chakraborty D., Kushwaha R., Vaidhyananthan R. (2024). J. Mater. Chem. A.

[cit37] Liu Y.-L., Zhang W. (2017). Chem. Commun..

[cit38] Singh H., Tomer V. K., Jena N., Bala I., Sharma N., Nepak D., Sarkar A. D., Kailasam K., Pal S. K. (2017). J. Mater. Chem. A.

[cit39] Ye W., Cao Q., Cheng X.-F., Yu C., He J.-H., Lu J.-M. (2020). J. Mater. Chem. A.

[cit40] Ogawa T., Anilkumar G. M., Tamaki T., Ohashi H., Yamaguchi T. (2022). Mater. Chem. Front..

[cit41] Zhang L., Yang L., He Y., Han J.-M. (2022). J. Mater. Chem. A.

[cit42] Duyker S. G., Halder G. J., Southon P. D., Price D. J., Edwards A. J., Peterson V. K., Kepert C. J. (2014). Chem. Sci..

[cit43] Aggarwal H., Das R. K., Engel E. R., Barbour L. J. (2017). Chem. Commun..

[cit44] Reczyński M., Chorazy S., Nowicka B., Sieklucka B., Ohkoshi S. (2017). Inorg. Chem..

[cit45] Duan Z., Jiang Y., Yan M., Wang S., Yuan Z., Zhao Q., Sun P., Xie G., Du X., Tai H. (2019). ACS Appl. Mater. Interfaces.

[cit46] Lee H., Lee D., Jim H., Baek D., Kim M. K., Cha J., Kim S.-K., Kim M. (2022). Nanoscale Adv..

[cit47] Zhan L., Tang Y., Ning W., Xie G., Zhong C., Gong S., Yang C. (2023). Chem. Eng. J..

[cit48] Bagghi A., Deniard P., Cartigny Y., Dessapt R. (2024). J. Mater. Chem. C.

[cit49] Wenger O. S. (2018). J. Am. Chem. Soc..

[cit50] Das K., Waiba S., Jana A., Maji B. (2022). Chem. Soc. Rev..

[cit51] Zheng R., Guo J., Cai X., Bin L., Lu C., Singh A., Trivedi M., Kumar A., Liu J. (2022). Colloids Surf., B.

[cit52] Berlinguette C. P., Vaughn D., Canada-Vilalta C., Galan-Mascaros J. R., Dunbar K. R. (2003). Angew. Chem., Int. Ed..

[cit53] Guo F.-S., Chen Y.-C., Liu J.-L., Leng J.-D., Meng Z.-S., Vrabel P., Orendac M., Tong M.-L. (2012). Chem. Commun..

[cit54] Konieczny P., Chorazy S., Pełka R., Bednarek K., Wasiutyński T., Baran S., Sieklucka B., Podgajny R. (2017). Inorg. Chem..

[cit55] Rajnak C., Titis J., Moncol J., Micova R., Boca R. (2019). Inorg. Chem..

[cit56] Hu J.-X., Li Q., Zhu H.-L., Gao Z.-N., Zhang Q., Liu T., Wang G.-M. (2022). Nat. Commun..

[cit57] Qin Y., She P., Huang X., Huang W., Zhao Q. (2020). Coord. Chem. Rev..

[cit58] Morad V., Cherniukh I., Pöttschacher L., Shynkarenko Y., Yakunin S., Kovalenko M. V. (2019). Chem. Mater..

[cit59] Wang S., Han X., Kou T., Zhou Y., Liang Y., Wu Z., Huang J., Chang T., Peng C., Wei Q., Zou B. (2021). J. Mater. Chem. C.

[cit60] Meng H., Zhu W., Zhou Z., Zhou R., Yan D., Zhao Q., Liu S. (2022). J. Mater. Chem. C.

[cit61] Davydova M. P., Meng L., Rakhmanova M. I., Bagryanskaya I. Y., Sulyaeva C. S., Meng H., Artem’ev A. V. (2023). Adv. Opt. Mater..

[cit62] Adranno B., Paterlini V., Smetana V., Bousrez G., Ovchinnikov A., Mudring A.-V. (2023). Dalton Trans..

[cit63] Zhang Y., Liao W.-Q., Fu D.-W., Ye H.-Y., Chen Z.-N., Xiong R.-G. (2015). J. Am. Chem. Soc..

[cit64] Zhang Y., Liao W.-Q., Fu D.-W., Ye H.-Y., Liu C.-M., Chen Z.-N., Xiong R.-G. (2015). Adv. Mater..

[cit65] Zhang H., Tan Y.-H., Tang Y.-Z., Fan X.-W., Peng X.-L., Han R.-R., Li Y.-K., Wang F.-X. (2022). Inorg. Chem..

[cit66] Wu L.-K., Zou Q.-H., Yao H.-Q., Ye H.-Y., Li J.-R. (2023). Dalton Trans..

[cit67] Berezin A. S., Samsonenko D. G., Brel V. K., Artem’ev A. V. (2018). Dalton Trans..

[cit68] Schmitt T., Bourelle S., Tye N., Soavi G., Bond A. D., Feldmann S., Traore B., Katan C., Even J., Dutton S. E., Deschler F. (2020). J. Am. Chem. Soc..

[cit69] Park H., Ha C., Lee J.-H. (2020). J. Mater. Chem. A.

[cit70] Zhang T., Li J.-Y., Du G.-W., Ding K., Chen X.-G., Zhang Y., Fu D.-W. (2022). Inorg. Chem. Front..

[cit71] Qiu Z.-X., Zheng Z.-X., Jiang X.-M., Liu B.-W., Guo G.-C. (2023). Chem. Sci..

[cit72] Wang L.-X., Wu X.-F., Jin X.-X., Li J.-Y., Wang B.-W., Liu J.-Y., Xiang J., Gao S. (2023). Dalton Trans..

[cit73] Jankowski R., Zakrzewski J. J., Zychowicz M., Wang J., Oki Y., Ohkoshi S., Chorazy S., Sieklucka B. (2021). J. Mater. Chem. C.

[cit74] Zaręba J. K., J Białek M., Janczak J., Nyk M., Zoń J., Samoć M. (2015). Inorg. Chem..

[cit75] Wu C., Li L., Song J., Yang G., Humprey M. G., Zhang C. (2017). Inorg. Chem..

[cit76] Wang S., Zhang Y., Halasyamani P. S., Mitzi D. B. (2024). Inorg. Chem..

[cit77] Shi P.-P., Tang Y.-Y., Li P.-F., Liao W.-Q., Wang Z.-X., Ye Q., Xiong R.-G. (2016). Chem. Soc. Rev..

[cit78] Kholkin A. L. , Pertsev N. A. and Goltsev A. V., Piezoelectricity and Crystal Symmetry, in Piezoelectric and Acoustic Materials for Transducer Applications, ed. A. Safari and E. K. Akdogan, Springer, 2008, pp. 17–38

[cit79] Sezer N., Koc M. (2021). Nano Energy.

[cit80] Lubomirsky I., Stafsudd O. (2012). Rev. Sci. Instrum..

[cit81] Zhang D., Wu H., Bowen C. R., Yang Y. (2021). Small.

[cit82] Nikolic K., Lignou F., de la Garanderie H. P. (1973). J. Lumin..

[cit83] Morad V., Cherniukh I., Pötschacher L., Shynkarenko Y., Yahunin S., Kovalenko M. V. (2019). Chem. Mater..

[cit84] Lawson K. E. (1967). J. Chem. Phys..

[cit85] Pitula S., Mudring A.-V. (2010). Chem.–Eur. J..

[cit86] Barreda-Argüeso J. A., Nataf L., Rodríguez-Lazcano Y., Aguado F., González J., Valiente R., Rodríguez F., Wilhelm H., Jephcoat A. P. (2014). Inorg. Chem..

[cit87] Orive J., Mesa J. L., Balda R., Fernández J., Rodríguez Fernández J., Rojo T., Arriortua M. I. (2011). Inorg. Chem..

[cit88] Song E., Ye S., Liu T., Du P., Si R., Jing X., Ding S., Peng M., Zhang Q., Wondraczek L. (2015). Adv. Sci..

[cit89] Valeur B. and Berberan-Santos M. N., Molecular Fluorescence, Wiley, 2012, pp. 213–261

[cit90] Neese F. (2012). Wiley Interdiscip. Rev. Comput. Mol. Sci..

[cit91] Neese F. (2022). Wiley Interdiscip. Rev. Comput. Mol. Sci..

[cit92] Kollmar C., Sivalingam K., Helmich-Paris B., Angeli C., Neese F. (2019). J. Comput. Chem..

[cit93] Lang L., Atanasov M., Neese F. (2020). J. Phys. Chem. A.

[cit94] Angeli C., Cimiraglia R., Malrieu J. P. (2002). J. Chem. Phys..

[cit95] Krohns S., Lunkenheimer P. (2019). Phys. Sci. Rev..

[cit96] V Topping C., Blundell S. J. (2019). J. Phys.: Condens. Matter.

[cit97] Kutnjak Z., Filipič C., Pirc R., Levstik A., Farhi R., El Marssi M. (1999). Phys. Rev. B: Condens. Matter Mater. Phys..

[cit98] Bobnar V., Kutnjak Z., Pirc R., Blinc R., Levstik A. (2000). Phys. Rev. Lett..

[cit99] Hemberger J., Lunkenheimer P., Fichtl R., Krug von Nidda H.-A., Tsurkan V., Loidl A. (2005). Nature.

[cit100] Bjerrum N. (1952). Science.

[cit101] Sugimoto Y. (2022). Science.

[cit102] von Hippel A. R. (1988). IEEE Trans. Electr. Insul..

[cit103] Kaatze U. , in Dielectric Relaxation in Biological Systems, Oxford University Press, 2015, pp. 189–227

[cit104] Popov I., Puzenko A., Khamzin A., Feldman Y. (2015). Phys. Chem. Chem. Phys..

[cit105] Popov I., Ben Ishai P., Khamzin A., Feldman Y. (2016). Phys. Chem. Chem. Phys..

[cit106] Asghar M. A., Zhang S., Khan T., Sun Z., Zeb A., Ji C., Li L., Zhao S., Luo J. (2016). J. Mater. Chem. C.

[cit107] Yang W.-L., Yan X., Wang M., Yuan H., Tang Y.-Y., Qin Y., Song X.-J. (2024). Inorg. Chem. Front..

[cit108] Liberka M., Zychowicz M., Zychowicz W., Chorazy S. (2022). Chem. Commun..

[cit109] Van Vleck J. H. (1941). Phys. Rev..

[cit110] Briganti M., Santanni F., Tesi L., Totti F., Sessoli R., Lunghi A. (2021). J. Am. Chem. Soc..

[cit111] Zakrzewski J. J., Kumar K., Zychowicz M., Jankowski R., Wyczesany M., Sieklucka B., Ohkoshi S., Chorazy S. (2021). J. Phys. Chem. Lett..

[cit112] Santana F. S., Perfetti M., Briganti M., Sacco F., Poneti G., Ravera E., Soares J. F., Sessoli R. (2022). Chem. Sci..

[cit113] Aravena D., Ruiz E. (2020). Dalton Trans..

[cit114] Benniston A. C., Melnic S., Turta C., Arauzo A. B., Bartolome J., Bartolome E., Harrington R. W., Probert M. R. (2014). Dalton Trans..

[cit115] de Cunha T. R., Barbosa V. M. M., Oliveira W. X. C., Pedroso E. F., Garcia D. M. A., Nunes W. C., Pereira C. L. M. (2020). Inorg. Chem..

[cit116] Uchida K., Cosquer G., Sugisaki K., Matsuoka H., Saot K., Breedlove B. K., Yamashita M. (2019). Dalton Trans..

[cit117] Nakajima H., Uchida K., Yoshida T., Horii Y., Sato T., Luming Z., Yamashita S., Nakazawa Y., Agulto V. C., Nakajima M., Breedlove B. K., Yamashita M., Iguchi H., Takaishi S. (2023). ChemPhysChem.

[cit118] Dunstan M. A., Brown D. S., Sorace L., Mole R. A., Boskovic C. (2022). Chem.–Asian J..

[cit119] Wang Q., Jin J., Wang Z., Ren S., Ye W., Dou Y., Liu S., Morris A., Slebodnick C., Quan L. (2024). J. Am. Chem. Soc..

[cit120] Deng Y., Wang M., Zhuang Y., Liu S., Huang W., Zhao Q. (2021). Light Sci. Appl..

