# Supplementary material for: Photoluminescent, dielectric, and magnetic responsivity to the humidity variation in SHG-active pyroelectric manganese(ii)-based molecular material
Source: Chem Sci. 2025 Apr 15;16(20):8979–97. doi: 10.1039/d5sc00404g (PMC12013632; doi:10.1039/d5sc00404g)
Supplement: SC-016-D5SC00404G-s001 [file SC-016-D5SC00404G-s001.pdf]

Supplementary Information (SI) for Chemical Science.
This journal is © The Royal Society of Chemistry 2025

Supporting information

Photoluminescent, dielectric, and magnetic responsivity to the humidity variation in SHG-active pyroelectric manganese(II)-based molecular material

Aleksander Hoffman,[ab] Mikolaj Zychowicz,[ab] Junhao Wang,[c] Keisuke Matsuura,[de] Fumitaka Kagawa,[e] Jan Rzepiela,[ab] Michal Heczko,[a] Sebastian Baś,[a] Hiroko Tokoro,[c] Shin-ichi Ohkoshi,[f] and Szymon Chorazy*[a]

[a]Faculty of Chemistry, Jagiellonian University, Gronostajowa 2, 30-387 Kraków, Poland; [b]Doctoral School of Exact and Natural Sciences, Jagiellonian University, Łojasiewicza 11, 30-348 Kraków, Poland; [c]Department of Materials Science, Faculty of Pure and Applied Science, University of Tsukuba, 1-1-1 Tennodai, Tsukuba, Ibaraki 305-8573, Japan; [d]Department of Physics, Tokyo Institute of Technology, 2-12-1 O-Okayama, Meguro, Tokyo 152-8551, Japan; [e]RIKEN Center for Emergent Matter Science (CEMS), 2-1 Hirosawa, Wako, 351-0198, Japan; [f]Department of Chemistry, School of Science, The University of Tokyo, 7-3-1 Hongo, Bunkyo-ku, Tokyo 113-0033, Japan.
*Corresponding author: simon.chorazy@uj.edu.pl

Experimental section, including the schematic visualization of the homemade experimental setup used for humidity-variable photoluminescence studies performed for the polycrystalline sample of **1**. (Scheme S1)	**S6**
Infrared (IR) absorption spectra of **1** and **2**, compared with the analogous spectra for **dppmO$_2$** and **Me-dppmO$_2$** ligands. (Fig. S1)	**S14**
Thermogravimetric curves (TG) for compounds **1** and **2**. (Fig. S2)	**S14**
Details of crystal data and structure refinement for **Me-dppmO$_2$**, **1** (at various temperatures), and **2**. (Table S1)	**S15**
Representative views of the crystal structure of **Me-dppmO$_2$**. (Fig. S3)	**S16**
Representative views of the crystal structure of **1^{100K}** (compound **1** at 100(2) K). (Fig. S4)	**S17**
Representative views of the crystal structure of **1^{270K}** (compound **1** at 270(2) K). (Fig. S5)	**S18**
Selected detailed structure parameters of **1^{100K}**. (Table S2)	**S19**
Selected detailed structure parameters of **1^{270K}**. (Table S3)	**S20**
Representative views of the crystal structure of **1^{deh,300K}** (the dehydrated phase of **1** at 300(2) K). (Fig. S6)	**S21**
Representative views of the crystal structure of **1^{deh,330K}** (the dehydrated phase of **1** at 330(2) K). (Fig. S7)	**S22**
Selected detailed structure parameters of **1^{deh,300K}**. (Table S4)	**S23**
Selected detailed structure parameters of **1^{deh,330K}**. (Table S5)	**S24**
Representative views of the crystal structure of **1^{deh,100K}** (the dehydrated phase of **1** at 100(2) K). (Fig. S8)	**S25**
Selected detailed structure parameters of **1^{deh,100K}**. (Table S6)	**S26**
Representative views of the crystal structure of **2**. (Fig. S9)	**S27**
Selected detailed structure parameters of **2**. (Table S7)	**S28**
Results of Continuous Shape Measure analysis for six- and four-coordinated Mn(II) centers in **1** and **2**. (Table S8)	**S29**
Powder X-ray diffraction (P-XRD) patterns of the powder samples of **1** (conditioned at various humidity levels and placed in the water solution) and **2**, compared with the respective P-XRD patterns from the structural models obtained by the SC-XRD experiments. (Fig. S10)	**S30**
Wavelength dependences of the SHG signal for the powder samples of **1**, **2**, and the reference of **KDP**. (Fig. S11)	**S31**
Dependences of the SHG effect on the excitation intensity for the powder samples of **1** and **KDP**. (Fig. S12)	**S32**
The results of the *in situ* studies of the SHG effect intensity of the pellet sample of **1** under variable indicated relative humidity (RH) and temperature conditions. (Fig. S13)	**S33**

Summary of the average SHG signals achieved in the *in situ* studies of the dependence of the SHG effect of **1** on the variable relative humidity (RH) and temperature. (Table S9)	**S33**
Comment to Fig. S13 and Table S9.	**S34**
Photos of the selected indexed single crystal of **1** for pyroelectric measurement and photos of wiring of the pyroelectric measurement setup. (Fig. S14)	**S35**
Detailed curves for pyroelectric measurements performed for **1** including temperature sweeping rate profiles for warming and cooling runs, average pyroelectric coefficient measured on the *a*-axis along the *b*-plane, and on the *c*-axis along the *b*-plane. (Fig. S15)	**S36**
Solid-state UV-vis absorption spectra for **1**, **2**, **Me-dppmO$_2$**, and **dppmO$_2$**. (Fig. S16)	**S37**
Solid-state photoluminescent characteristics of free organic molecules of **dppmO$_2$** and **Me-dppmO$_2$**, gathered at room temperature and 77 K, shown together with their photos under UV light irradiation. (Fig. S17)	**S38**
Temperature-variable solid-state excitation spectra of **1** for the monitored emission at 580 nm, gathered in the 10−310 K range. (Fig. S18)	**S39**
Temperature-variable solid-state emission spectra of **1** under the 280 and 360 nm excitation, gathered in the 10−310 K range. (Fig. S19)	**S40**
Comparison of the solid-state emission spectra of **1** at 310 K and 10K under the excitation by the wavelengths of λ_{exc} = 280 nm and λ_{exc} = 360 nm. (Fig. S20)	**S40**
Emission colors for free **Me-dppmO$_2$** and **dppmO$_2$** molecules under variable temperature and excitation conditions, presented on the CIE 1931 chromaticity diagram. (Fig. S21)	**S41**
The summary of the CIE 1931 chromaticity parameters for emission signals of free **Me-dppmO$_2$** and **dppmO$_2$** molecules under variable temperature and excitation conditions. (Table S10)	**S41**
Emission colors for the powder sample of **1** under the excitation of λ_{exc} = 280 nm and λ_{exc} = 360 nm, for the 10−310 K temperature range, presented on the CIE 1931 chromaticity diagrams. (Fig. S22)	**S42**
Chromaticity parameters of the CIE 1931 scale of the solid-state emission of **1** at variable temperatures from the 10–310 K range. (Table S11)	**S43**
Comparison of emission spectra and emission colors presented on the CIE 1931 chromaticity diagram for the powder samples of **1** and free **Me-dppmO$_2$** molecules. (Fig. S23)	**S44**
Temperature-variable emission decay profiles for **1** under the excitation of λ_{exc} = 280 nm for the emission of λ_{em} = 580 nm, gathered in the 10−80 K temperature range. (Fig. S24)	**S45**
Temperature-variable emission decay profiles for **1** under the excitation of λ_{exc} = 280 nm for the emission of λ_{em} = 580 nm, gathered in the 90−160 K temperature range. (Fig. S25)	**S46**
Temperature-variable emission decay profiles for **1** under the excitation of λ_{exc} = 280 nm for the emission of λ_{em} = 580 nm, gathered in the 170−240 K temperature range. (Fig. S26)	**S47**
Temperature-variable emission decay profiles for **1** under the excitation of λ_{exc} = 280 nm for the emission of λ_{em} = 580 nm, gathered in the 250−310 K temperature range (Fig. S27)	**S48**
Temperature-variable emission decay profiles for **1** under the excitation of λ_{exc} = 360 nm for the emission of λ_{em} = 580 nm, gathered in the 10−80 K temperature range. (Fig. S28)	**S49**
Temperature-variable emission decay profiles for **1** under the excitation of λ_{exc} = 360 nm for the emission of λ_{em} = 580 nm, gathered in the 90−160 K temperature range. (Fig. S29)	**S50**
Temperature-variable emission decay profiles for **1** under the excitation of λ_{exc} = 360 nm for the emission of λ_{em} = 580 nm, gathered in the 170−240 K temperature range. (Fig. S30)	**S51**
Temperature-variable emission decay profiles for **1** under the excitation of λ_{exc} = 360 nm for the emission of λ_{em} = 580 nm, gathered in the 250−310 K temperature range (Fig. S31)	**S52**
Best-fit parameters to the mono-exponential decay function for the temperature-variable emission decay profiles of **1** (λ_{exc} = 280 nm and λ_{em} = 580 nm, as well as λ_{exc} = 360 nm and λ_{em} = 580 nm) which were gathered in the 10−310 K temperature. (Table S12)	**S53**
The absolute quantum yield of **1** at room temperature in the solid state. (Table S13)	**S54**
Comment on theoretical calculations of optical transitions in Mn(II) complexes in **1**. (Fig. S32–S35 and S66, as well as Tables S14–S22 and S35–S38)	**S54**

Visualization of active space, composed of five 3d orbitals, applied for the computational procedures for Mn1 centers (ground state geometry). (Fig. S32) **S55**
Visualization of active space, composed of five 3d orbitals, applied for the computational procedures for Mn2 centers (ground state geometry). (Fig. S33) **S55**
Visualization of active space, composed of five 3d orbitals, applied for the computational procedures for Mn2 centers (optimized excited state geometry). (Fig. S34) **S55**
The *ab initio*-calculated energy splitting schemes for Mn1 and Mn2 centers of **1** using various theory levels, shown on the experimental excitation spectrum of **1**. (Fig. S35) **S56**
Energies of excited states obtained from CASSCF-type *ab initio* calculations for Mn1 and Mn2 centers of **1** when taking into account only the quartet excited states, shown together with the resulting wavelengths of optical transitions and the values of energies and wavelengths after the scaling procedure. (Table S14) **S57**
Energies of excited states obtained from CASSCF-type *ab initio* calculations for Mn1 and Mn2 centers of **1** when taking into account the quartet and doublet excited states, shown together with the resulting wavelengths of optical transitions and the values of energies and wavelengths after the scaling procedure. (Table S15) **S58**
Energies of ground and excited states obtained from CASSCF-SOC-type *ab initio* calculations for Mn1 and Mn2 centers of **1** when taking into account only the quartet excited states, shown together with the resulting wavelengths of optical transitions and the values of energies and wavelengths after the scaling procedure. (Table S16) **S61**
Energies of ground and excited states obtained from CASSCF-SOC-type *ab initio* calculations for Mn1 and Mn2 centers of **1** when taking into account the quartet and doublet excited states, shown together with the resulting wavelengths of optical transitions and the values of energies and wavelengths after the scaling procedure. (Table S17) **S64**
Energies of excited states obtained from CASSCF-NEVPT2-type *ab initio* calculations for Mn1 and Mn2 centers of **1** when taking into account only the quartet excited states, shown together with the resulting wavelengths of optical transitions and the values of energies and wavelengths after the scaling procedure. (Table S18) **S71**
Energies of excited states obtained from CASSCF-NEVPT2-type *ab initio* calculations for Mn1 and Mn2 centers of **1** when taking into account the quartet and doublet excited states, shown together with the resulting wavelengths of optical transitions and the values of energies and wavelengths after the scaling procedure. (Table S19) **S72**
Energies of ground and excited states obtained from CASSCF-NEVPT2-SOC-type *ab initio* calculations for Mn1 and Mn2 centers of **1** when taking into account only the quartet excited states, shown together with the resulting wavelengths of optical transitions and the values of energies and wavelengths after the scaling procedure. (Table S20) **S75**
Energies of ground and excited states obtained from CASSCF-NEVPT2-SOC-type *ab initio* calculations for Mn1 and Mn2 centers of **1** when taking into account the quartet and doublet excited states, shown together with the resulting wavelengths of optical transitions and the values of energies and wavelengths after the scaling procedure. (Table S21) **S78**
Energies of ground and excited states obtained from CASSCF-NEVPT2-SOC-type *ab initio* calculations for Mn2 centers of **1** in the optimized geometry of the lowest-lying excited quartet state, taking into account only the quartet excited states, shown together with the resulting wavelengths of optical transitions representing the hypothetical luminescent transitions for these complexes. (Table S22) **S85**
Humidity-dependent emission spectra of **1** in the 17–90% relative humidity (RH) range at room temperature and the related RH-dependences of the intensity of an emission maximum with the linear fits. (Fig. S36) **S87**
Best-fit parameters for the relative humidity-dependent intensity of the emission maximum of **1** (fitting to the linear function) in the 10-90% RH range at RT for two cycles of desorption and sorption. (Table S23) **S88**
Humidity-dependent emission decay profiles of **1** in the 20–87% relative humidity range at room temperature for the sorption cycle. (Fig. S37) **S89**
Humidity-dependent emission decay profiles of **1** in the 20–87% relative humidity range at room temperature for the desorption cycle. (Fig. S38) **S90**
Best-fit parameters to the mono-exponential decay function for the humidity-dependent emission decay profiles of **1**, which were gathered in the 20–87% relative humidity range at room temperature for the sorption cycle (Table S24) **S91**

Best-fit parameters to the mono-exponential decay function for the humidity-dependent emission decay profiles of **1**, which were gathered in the 20–87% relative humidity range at room temperature for the desorption cycle (Table S25) **S92**

Humidity dependencies of emission lifetime of **1** at room temperature, for the sorption cycle and the desorption cycle, shown with the best linear fits. (Fig. S39) **S93**

Best-fit parameters (fitting to the linear function) for the humidity-dependences of emission lifetime of **1** for the sorption and desorption cycles in the 20–87% relative humidity range at room temperature. (Table S26) **S93**

Humidity dependencies of emission lifetime of **1** at room temperature, for the sorption cycle and the desorption cycle, shown with the best fits to the allometric function. (Fig. S40) **S94**

Best-fit parameters (fitting to the allometric function) for the humidity-dependences of emission lifetime of **1** for the sorption and desorption cycles in the 20–87% relative humidity range at room temperature. (Table S27) **S94**

Temperature-variable dielectric characteristics of **1** upon two cycles of cooling-heating (the 1st cycle and the 2nd cycle) within the 173–293 K range, including frequency dependences of the real part, ε', and the imaginary part, ε'', of the electrical permittivity, with the best-fit curves to the generalized Debye model, and the temperature dependencies of dielectric relaxation times, τ_E, with the best fit to the Arrhenius equation. (Fig. S41) **S95**

T-variable dielectric characteristics of **1** upon two cycles of cooling-heating (the 3rd cycle and the 4th cycle, after the cycles shown in Fig. S22) within the 173–353 K range, including frequency dependences of the real part, ε', and the imaginary part, ε'', of the electrical permittivity, with the best-fit curves to the generalized Debye model, and the *T*-dependencies of dielectric relaxation times, τ_E, with the best fit to the Arrhenius equation. (Fig. S42) **S96**

T-variable dielectric characteristics of $\mathbf{1^{reh}}$ (the re-hydrated sample of **1**) upon two cycles of cooling-heating (the 1st cycle and the 2nd cycle) within the 173–293 K range, including frequency dependences of the real part, ε', and the imaginary part, ε'', of the electrical permittivity, with the best-fit curves to the generalized Debye model, and the *T*-dependencies of dielectric relaxation times, τ_E, with the best fit to the Arrhenius equation. (Fig. S43) **S97**

T-variable dielectric characteristics of $\mathbf{1^{reh}}$ upon two cycles of cooling-heating (the 3rd cycle and the 4th cycle, after the cycles shown in Fig. S24) within the 173–353 K range, including frequency dependences of the real part, ε', and the imaginary part, ε'', of the electrical permittivity, with the best-fit curves to the generalized Debye model, and the *T*-dependencies of dielectric relaxation times, τ_E, with the best fit to the Arrhenius equation. (Fig. S44) **S98**

T-variable dielectric characteristics of **2** upon two cycles of cooling-heating (the 1st cycle and the 2nd cycle) within the 173–293 K range, including frequency dependences of the real part, ε', and the imaginary part, ε'', of the electrical permittivity, with the best-fit curves to the generalized Debye model, and the temperature dependencies of dielectric relaxation times, τ_E, with the best fit to the Arrhenius equation. (Fig. S45) **S99**

Temperature-variable dielectric characteristics of **2** upon two cycles of cooling-heating (the 3rd cycle and the 4th cycle, after the cycles shown in Fig. S26) within the 173–353 K range, including frequency dependences of the real part, ε', and the imaginary part, ε'', of the electrical permittivity, with the best-fit curves to the generalized Debye model, and the *T*-dependencies of dielectric relaxation times, τ_E, with the best fit to the Arrhenius equation. (Fig. S46) **S100**

T-variable dielectric characteristics of $\mathbf{2^{reh}}$ (the re-hydrated sample of **2**) upon two cycles of cooling-heating (the 1st cycle and the 2nd cycle) within the 173–293 K range, including frequency dependences of the real part, ε', and the imaginary part, ε'', of the electrical permittivity, with the best-fit curves to the generalized Debye model, and the *T*-dependencies of dielectric relaxation times, τ_E, with the best fit to the Arrhenius equation. (Fig. S47) **S101**

T-variable dielectric characteristics of $\mathbf{2^{reh}}$ upon two cycles of cooling-heating (the 3rd cycle and the 4th cycle, after the cycles shown in Fig. S28) within the 173–353 K range, including frequency dependences of the real part, ε', and the imaginary part, ε'', of the electrical permittivity, with the best-fit curves to the generalized Debye model, and the *T*-dependencies of dielectric relaxation times, τ_E, with the best fit to the Arrhenius equation. (Fig. S48) **S102**

Summary of best-fit parameters to the Arrhenius equation used for the *T*-dependencies of dielectric relaxation times for compounds **1**, $\mathbf{1^{reh}}$, **2**, and $\mathbf{2^{reh}}$, determined separately for the cycles of cooling and heating. (Table S28) **S103**

Direct-current (*dc*) magnetic characteristics of the powder samples of **1** (23% RH), $\mathbf{1^{deh}}$ (0% RH), and $\mathbf{1^{HH}}$ (the sample of **1** at the high relative humidity, 90% RH). (Fig. S49) **S104**

Magnetic-field-variable alternate-current (*ac*) magnetic characteristics of **1** (23% RH) at *T* = 1.8 K. (Fig. S50) **S105**

Temperature-variable alternate-current (*ac*) magnetic characteristics of **1** (23% RH) under H_{dc} of 1 kOe. (Fig. S51) **S106**

Temperature-variable alternate-current (*ac*) magnetic characteristics of **1** (23% RH) under H_{dc} of 5 kOe. (Fig. S52) **S107**

Magnetic-field-variable alternate-current (*ac*) magnetic characteristics of **1**^{deh} (0% RH) at T = 1.8 K. (Fig. S53) **S108**
Temperature-variable alternate-current magnetic characteristics of **1**^{deh} (0% RH) under H_{dc} of 1 kOe. (Fig. S54) **S109**
Temperature-variable alternate-current magnetic characteristics of **1**^{deh} (0% RH) under H_{dc} of 5 kOe. (Fig. S55) **S110**
Magnetic-field-variable alternate-current (*ac*) magnetic characteristics of **1**^{HH} (90% RH) at T = 1.8 K. (Fig. S56) **S111**
T-variable alternate-current (*ac*) magnetic characteristics of **1**^{HH} (90% RH) under H_{dc} of 1 kOe. (Fig. S57) **S112**
T-variable alternate-current (*ac*) magnetic characteristics of **1**^{HH} (90% RH) under H_{dc} of 5 kOe. (Fig. S58) **S113**
Summary of the best-fit slow magnetic relaxation parameters for **1** and **1**^{HH} obtained within the three-dimensional simultaneous fitting of the field dependence at T = 1.8 K and the temperature dependence at H_{dc} =1000 Oe of magnetic relaxation times. (Table S29) **S114**
Summary of the best-fit slow magnetic relaxation parameters for **1** and **1**^{deh} obtained within the fitting of the temperature dependences at H_{dc} = 5000 Oe of magnetic relaxation times. (Table S30) **S114**
DSC curves of **1** gathered in the variable temperature ranges and sweeping rates, with the comment. (Fig. S59) **S115**
Crystal data and structure refinement parameters for reference compounds **3** and **4**. (Table S31) **S117**
Representative views of the crystal structure of Zn(II)-containing reference compound **3**. (Fig. S60) **S118**
Representative views of the crystal structure of Zn(II)-containing reference compound **4**. (Fig. S61) **S119**
Selected detailed structure parameters of Zn(II)-containing reference compound **4**. (Table S32) **S120**
Comment to Fig. S60–S62, as well as Tables S30 and S31. **S121**
Powder X-ray diffraction (P-XRD) patterns of **3** and **4**, compared with the respective P-XRD patterns from the structural models obtained by the SC-XRD experiments, and with the P-XRD pattern of **1**^{100K}. (Fig. S62) **S122**
Additional comment to Fig. S62. **S122**
Direct-current (*dc*) magnetic characteristics of Zn(II)-containing reference compound **4.** (Fig. S63) **S123**
Magnetic-field-variable alternate-current (*ac*) magnetic characteristics of **4** at T = 1.8 K. (Fig. S64) **S124**
Temperature-variable alternate-current (*ac*) magnetic characteristics of **4** under H_{dc} of 1 kOe. (Fig. S65) **S125**
Summary of the best-fit slow magnetic relaxation parameters for Zn(II)-containing reference compound **4** obtained within the three-dimensional simultaneous fitting of the field dependence at T = 1.8 K and the temperature dependence at H_{dc} =1000 Oe of magnetic relaxation times. (Table S33) **S126**
Comment to Fig. S63–S64 and Table S33. **S126**
Comparison of the structural model of Mn1 complexes from the experimental SC–XRD analysis (**1**^{100K}) and the structural model of the same complexes, named Mn1^{opt}, with the optimized hydrogen atoms at ZORA-B3LYP level of theory using "Tight" convergence criteria for wavefunction and gradients. (Fig. S66) **S127**
Comparison between carbon-hydrogen bond lengths of the Mn1 complexes obtained from the SC-XRD experiment and the Mn1^{opt} model of the same complex with the hydrogen atoms optimized with the ZORA-B3LYP level of theory using "Tight" convergence criteria for wavefunction and gradients. (Table S34) **S128**
Comparison of the energies of excited states obtained from CASSCF-type *ab initio* calculations for Mn1^{opt} and Mn1 models of octahedral Mn(II) complexes of **1** when taking into account the quartet and doublet excited states, shown together with the resulting wavelengths of optical transitions and the values of energies and wavelengths after the scaling procedure. (Table S35) **S129**
Comparison of the energies of ground and excited states obtained from CASSCF-SOC-type *ab initio* calculations for Mn1^{opt} and Mn1 models of octahedral Mn(II) complexes of **1** when taking into account the quartet and doublet excited states, shown together with the resulting wavelengths of optical transitions and the values of energies and wavelengths after the scaling procedure. (Table S36) **S132**
Comparison of the energies of excited states obtained from CASSCF-NEVPT2-type *ab initio* calculations for Mn1^{opt} and Mn1 models of octahedral Mn(II) complexes of **1** when taking into account the quartet and doublet excited states, shown together with the resulting wavelengths of optical transitions and the values of energies and wavelengths after the scaling procedure. (Table S37) **S139**
Comparison of the energies (E) of ground and excited states obtained from CASSCF-NEVPT2-SOC-type *ab initio* calculations for Mn1^{opt} and Mn1 models of octahedral Mn(II) complexes of **1** when taking into account the quartet and doublet excited states, shown together with the resulting wavelengths of optical transitions and the values of energies and wavelengths after the scaling procedure. (Table S38) **S140**
References to the Supporting Information. **S142**

Experimental section

Materials

Manganese(II) chloride tetrahydrate was purchased from Sigma-Aldrich, bis(diphenylphosphino)methane (**dppm**) was purchased from TCI Chemicals. All other reagents and solvents were purchased from Sigma-Aldrich or TCI Chemicals and used as received unless otherwise written.

Synthesis of bis(diphenylphosphino)methane dioxide (dppmO$_2$)

To a two-neck flask, an organic **dppm** precursor was added (10.00 g, 26.01 mmol) and dissolved in the mixture of DCM (280 mL) and MeOH (120 mL). The reaction mixture was cooled to –78 °C (dry ice/acetone bath) and the ozone and oxygen gas mixture, generated by the Prozonex ozone generator system, was passed through it (oxygen flow of 4 mL/min, power 70%). The reaction was run until the solution turned color to light blue. Full conversion of the substrate was confirmed by TLC (hexane/ethyl acetate, 1:4, v/v). After completing the reaction, the mixture was warmed to room temperature and purged with argon before further handling, then the solvent was removed on a rotary evaporator, and the resulting precipitate was dried using a vacuum pump. It gave the pure, white crystalline product of **dppmO$_2$**. Yield: 10.72 g (99%). The structure and purity of the obtained product were checked by NMR, IR, and ESI-MS studies, compared with the related published data for **dppmO$_2$** obtained using a different method.[S1]

^{1}H NMR (600 MHz, chloroform-d) δ 7.81–7.66 (m, 8H), 7.43–7.37 (m, 4H), 7.34–7.27 (m, 8H), 3.59 (t, *J* = 14.7 Hz, 2H).

^{13}C NMR (151 MHz, chloroform-d) δ 132.9 (s), 132.2 (s), 131.8 (s), 131.4–130.7 (m), 128.4 (p, *J* = 7,1 Hz).

^{31}P NMR (121 MHz, chloroform-d) δ 25.08 (s).

IR: 3318, 3053, 2925, 1589, 1437, 1205, 1189, 780, 741, 699 cm^{-1}.

LC-MS (ESI) [M+1] = 417 m/z .

Synthesis of bis(diphenylphosphino)-1,1-ethane dioxide (Me-dppmO$_2$)

To a flame-dried round-bottom flask in Ar atmosphere, a **dppmO$_2$** precursor was added (5.0 g, 1.0 eq., 12.01 mmol) and dispersed in THF (120 mL), then cooled to –78 °C (dry ice/acetone bath). After that, *n*BuLi (5.28 mL, 1.1 eq., 13.21 mmol, 2.5 M solution in hexane) was dropwise added and stirred. After 1 h, MeI (0.90 mL, 1.2 eq. 14.41 mmol) was dropwise added and the reaction mixture was brought to room temperature, then heated to 64 °C for 24 h. After checking the full conversion of the substrate (TLC, 5% MeOH in DCM), the reaction was quenched with water and concentrated on a rotary evaporator, then extracted with DCM (4 x 40 mL). Combined organic phases were dried over anhydrous sodium sulfate and evaporated to dryness obtaining the light-yellow crystalline product of **Me-dppmO$_2$**. Yield: 71% (3.68 g). The structure and purity of the obtained product were checked by NMR, IR, and ESI-MS studies, compared with the related published data for **Me-dppmO$_2$** obtained using a different procedure.[S2] The structure was also confirmed by the single-crystal X-ray diffraction (SC-XRD) method (Fig. S3 and Table S1). The single crystals of **Me-dppmO$_2$**, suitable for the SC-XRD experiment, were prepared by crystallization from DCM layered with *n*-pentane.

^{1}H NMR (600 MHz, chloroform-d) δ 7.97–7.89 (m, 4H), 7.73 (dt, *J* = 10.4, 8.4 Hz, 4H), 7.39 (dd, *J* = 10.5, 4.2 Hz, 2H), 7.37–7.30 (m, 6H), 7.26 (dt, *J* = 7.4, 3.1 Hz, 4H), 3.50 (tq, *J* = 15.2, 7.4 Hz, 1H), 1.36 (td, *J* = 15.6, 7.4 Hz, 3H).

^{13}C NMR (151 MHz, chloroform-d) δ 132.5 (s), 132.2 (s), 131.9–131.3 (m), 130.8 (s), 128.7–128.1 (m), 128.0–127.8 (m), 37.7 (s), 37.3 (s).

^{31}P NMR (121 MHz, chloroform-d) δ 31.75 (s).

IR: 3672, 3053, 2971, 2932, 2893, 1592, 1435, 1199, 1183, 117, 1018, 727, 693 cm^{-1}.

LC-MS (ESI) [M+1] = 431 m/z.

Synthesis of 1

The **Me-dppmO$_2$** precursor (50.0 mg, 0.116 mmol 3.0 eq.) and $Mn^{II}Cl_2 \cdot 4H_2O$ (15.3 mg, 0.072 mmol, 2.0 eq.) were dissolved together in MeCN (2 mL), then the reaction mixture was stirred for 2 h. The resulting precipitate was filtered on a sintered glass funnel, washed with Et_2O, and dried on vacuum. The obtained crude product was then

redissolved in a mixture of MeCN (1 mL) and MeOH (2 mL) and layered with Et_2O. After a few days, colorless crystals of **1** were filtered and washed with Et_2O. Yield: 48%.

The composition of **1**, $[Mn^{II}(Me\text{-}dppmO_2)_3][Mn^{II}Cl_4]\cdot H_2O$ (M_W = 1560.96 g·mol^{-1}), was determined by the SC-XRD experiment (Fig. 1 and Table S1) and CHN elemental analysis, while the phase purity and its air stability were proven by the P-XRD method (Fig. S10), as well as CHN elemental analysis and TG studies (Fig. S2). Elemental analysis calculated for **1** ($C_{78}H_{74}Cl_4Mn_2O_7P_6$; M_W = 1560.96 g mol^{-1}): C, 60.02 %; H, 4.78 %; N, 0.00%. Found: C, 60.41 %; H, 4.67 %; N, 0.00%. TG (Fig. S2): loss of 1 H_2O molecule per formula unit, calculated: 1.15%; found: 1.10%. IR spectrum (Fig. S1): a band located at 3514 cm^{-1} indicates the presence of water molecules.

Synthesis of 2

The **dppmO₂** precursor (50.0 mg, 0.12 mmol 3.0 eq.) and $Mn^{II}Cl_2\cdot 4H_2O$ (15.84 mg, 0.080 mmol, 2.0 eq.) were dissolved together in MeCN (2 mL). Similar to **1**, the reaction mixture was stirred for 2 h, then the precipitate was filtered on a sintered glass funnel, washed with Et_2O, and dried on vacuum. The obtained solid was then redissolved in a mixture of MeCN (1 mL) and MeOH (1 mL), and layered with Et_2O. After a few days, colorless crystals of **2** were filtered and washed with Et_2O. Yield 87%.

The composition of **2**, $[Mn^{II}(dppmO_2)_3][Mn^{II}Cl_4]\cdot 2MeCN$ (M_W = 1582.88 g·mol^{-1}), was determined by the SC-XRD experiment (Fig. S9 and Table S1), while the phase purity and its air stability were proven by the P-XRD method (Fig. S10), as well as CHN elemental analysis and TG studies (Fig. S2). Long-term exposition of the crystals of **2** to the air causes loss of MeCN molecules and sorption of water molecules which leads to the hydrated form with two water molecules per formula unit, phase $\mathbf{2^{hyd}}$. It does not cause a large structural transformation and these water molecules can be removed by an inert gas purge, which was confirmed by P-XRD results, CHN elemental analysis, and TG experiment (Fig. S2). Elemental analysis calculated for $\mathbf{2^{hyd}}$, $[Mn^{II}(dppmO_2)_3][Mn^{II}Cl_4]\cdot 2H_2O$ ($C_{75}H_{70}Cl_4Mn_2O_8P_6$; M_W = 1536.90 g mol^{-1}): C, 58.61 %; H, 4.59 %; N, 0.00%. Found: C, 58.61 %; H, 4.55 %; N, 0.00%. TG (Fig. S2): loss of 2 H_2O molecules per formula unit, calculated: 2.34 %; found: 2.40%. IR spectrum (Fig. S1): a band located at 3483 cm^{-1} indicates the presence of water molecules.

Synthetic details for the reference compounds **3** and **4** are presented in the comment on Page S121.

Structural studies

The single crystal X-ray diffraction (SC-XRD) data for **1**, **2**, and **Me-dppmO₂** were collected using a Bruker D8 Quest Eco Photon50 CMOS diffractometer, equipped with Mo*Kα* (0.71073 Å) X-ray radiation source, graphite monochromator, and the Oxford Cryostream cooling system. The single crystals of **1** were selected from the mother liquid, washed with diethyl ether three times, air-dried on a microscope slide, and mounted on the Micro Mounts™ holder. Then, the SC-XRD experiment was conducted at 100(2) K (the crystal structure abbreviated as $\mathbf{1^{100K}}$), 270(2) K ($\mathbf{1^{270K}}$), 300(2) K ($\mathbf{1^{deh,300K}}$, the label 'deh' added as the dehydrated phase was detected), and 330(2) K ($\mathbf{1^{deh,300K}}$). The structural data for all these temperatures were gathered on the same single crystal within a single measurement sequence with a relatively slow heating rate of 120 K/min. The single crystals of **2** and **Me-dppmO₂**, selected for the respective SC-XRD experiment, were taken directly from the mother liquid, covered in Apiezon N grease, mounted on the Micro Mounts™ holder, and measured at 100(2) K. Similarly, the single crystals of the reference compounds **3** and **4** were selected from the mother liquid, washed with diethyl ether three times, air-dried on a microscope slide, and mounted on the Micro Mounts™ holder, and measured at 100(2) K. The additional SC-XRD experiment was performed for the $\mathbf{1^{deh,100K}}$ phase (i.e., the dehydrated phase of **1**, measured at 100(2) K). The related diffraction data were collected using a Bruker D8 Venture diffractometer, equipped with Photon III CPAD detector, Mo *Kα* (0.71073 Å) INCOATEC INCOATEC IμS 3.0 microfocus sealed tube radiation source, Helios® optics, and Oxford Cryostream 800 Plus low-temperature device. For this experiment, the single crystal of **1** was selected from the mother liquid, washed with diethyl ether three times, air-dried on a microscope slide, and mounted on the Micro Mounts™ holder. Then, the crystal was heated up to 300(2) K and kept at this temperature under dry gas (nitrogen) flow for 2 hours, which was followed by cooling to 100(2) K. After that, the SC-XRD measurement for the $\mathbf{1^{deh,100K}}$ phase was performed.

For all crystal structures reported here, the SAINT and SADABS programs were used for data reduction and cell refinement processes. The absorption correction was performed using a multi-scan procedure using the TWINABS program.[S3] The crystal structures were solved by an intrinsic phasing method using a SHELXT program within the Apex3 package.[S4] The further crystal structures refinement was carried out by a weighted full-matrix least squares method on F^2 of SHELX-2014/7 within WinGX (ver. 2018.3) software.[S5] All non-hydrogen atoms were anisotropically refined. Hydrogen atoms were refined isotropically; those belonging to metal complexes, MeCN molecules, and **Me-dppmO$_2$** molecules were calculated in their idealized positions while those of water molecules were found from the residual electron density map. A riding model was used for the further refinement of all hydrogen atoms. Some restraints of the DFIX and ISOR types were applied for non-hydrogen atoms to ensure the proper geometries of molecular components. DELU and SIMU restraints were also applied for the MeCN solvent molecule in **2** to ensure the proper geometry and the convergence of the refinement procedure. Some of the reflections with intensities endowed with especially large errors (affected by the beamstop) were removed from the final refinement using the OMIT command. Using these procedures, satisfactory refinement parameters were achieved. The reference CCDC numbers are as follows: 2328045 (**Me-dppmO$_2$**), 2328046 (**1** measured at 100(2) K, named **1^{100K}**), 2328047 (**1** measured at 270(2) K, named **1^{270K}**), 2328049 (**1** measured at 300(2) K, named **1^{deh,300K}** as a dehydration process occurs at the related conditions), 2328048 (**1** measured at 330(2) K, named **1^{deh,330K}**), 2411622 (**1** measured at 100(2) K, named **1^{deh,100K}**), 2328050 (**2** measured at 100(2) K, named **2**), 2431595 (**3** measured at 100(2) K, named **3**), and 2431594 (**4** measured at 100(2) K, named **4**).

A few remarks should be added here on the refinement of water molecules of crystallization that appear for the crystal structure of **1^{100K}** and **1^{270K}**. For **1^{100K}** phase, the full occupation for a single, crystallographically independent, water molecule (at the same time, a single water molecule per the formula unit) was easy to identify; however, even at this temperature, the related thermal ellipsoid for an oxygen atom was much larger than for all the other atoms in the structure (Fig. S4). This indicates that this water molecule reveals a significant structural disorder which can be rationalized by their very weak stabilization in the crystal lattice, mainly through weak hydrogen bonds with chlorido ligands of $[Mn^{II}Cl_4]^{2-}$ ions (as represented by rather long O-(H)-Cl distance of 3.3–3.5 Å for two closest lying chlorido ligands). It is important to note that even for the low-temperature SC-XRD data at 100 K, it was not possible to describe the mentioned structural disorder present on this water molecule by proposing the two or more crystallographic positions of related oxygen atoms with partial occupancies (it leads to the instability of the refinement procedure). Therefore, it is not surprising that the structural disorder is even higher for the SC-XRD data gathered at 270 K (**1^{270K}** phase, Fig. S5). Primarily, we attempted to refine the structural data for the **1^{270K}** phase by inserting the water molecule with a partial (50%) occupancy to keep the related thermal ellipsoid of the oxygen atom at a reasonably small size when compared with other molecular components of the material. However, the resulting thermal ellipsoid was still larger than for all other atoms of the structure. More importantly, such results could suggest that partial dehydration occurs at this temperature when the crystal is placed in a dry nitrogen flow inside the diffractometer. This rather not the case as suggested by the air stability of the composition at room temperature (i.e., ca. 298 K) indicated by the CHN elemental analysis (see above), and the rather slow course of the dehydration process occurring at room temperature under the nitrogen flow as proven by the TG studies (Fig S2) and the necessity of spending of at least 30 minutes at 300 K to detect the disappearance of the electron density related to the water molecule in the SC-XRD experiment. Thus, taking into account the significant structural disorder observed for the water molecule even at 100 K, we postulate that the solvent content remains rather unchanged at 270 K. As a result, we decided to modify the occupancy of the related atoms (O, H) from 0.5 to 1. This makes the thermal ellipsoid of the related oxygen atom large in comparison to other atoms but it rather represents a large intrinsic structural disorder of the water molecule in the obtained compound investigated at 270 K. Similarly to the **1^{100K}** phase, this disorder could not be reasonably described by a few positions of the oxygen atoms of partial occupancies to the extremely diffuse electron density in this area. However, this can be still rationalized by the very weak bonding of these molecules through only weak H-bonds with chlorido ligands of $[Mn^{II}Cl_4]^{2-}$ ions. The structural

data obtained for **1** upon the further heating, i.e., $\mathbf{1}^{deh,300K}$ and $\mathbf{1}^{deh,330K}$ phases, do not contain significant electron density corresponding to solvent molecules of crystallization, thus the dehydration process is confirmed and the related crystal structures were refined with only considering two metal complexes (Mn1, Mn2) without any other molecular components (Fig. S6 and S7). This effect was also further checked by the crystal structure of $\mathbf{1}^{deh,100K}$ (see the Structural studies section of the main text and Fig. S8). Details of the crystal data and refinement of the structure are summarized in Tables S1 and S31, while representative structural parameters are gathered in Tables S2–S7 and S32. The structural figures (Fig. 1 and S3–S9, S60, and S61) were prepared using Mercury 3.8 software.

Powder XRD (P-XRD) data were collected using a Bruker D8 Advance Eco powder diffractometer equipped with a Cu Kα (1.5419 Å) radiation source and the add-on device for rotating capillaries. The P-XRD measurements were conducted at room temperature for the polycrystalline samples of **1**, **2**, **3**, and **4** which were inserted into the 0.7 mm glass capillaries. Separate measurements for the powder sample of **1,** conditioned (before the measurement) at various relative humidity conditions, were performed. They were done to measure the P-XRD patterns for the wet sample (under the water solution), the hydrated sample under ambient and high-humidity conditions, as well as the dehydrated sample. The details of these studies are provided in the caption to the related Fig. S10.

Physical techniques

All measurements were performed on freshly prepared, filtrated, and air-dried samples. The ^{1}H and ^{13}C NMR spectra were recorded at room temperature using a Bruker AVANCE III 600 MHz spectrometer, while the ^{31}P NMR spectra were recorded also at room temperature but using Bruker AVANCE II 300 MHz. CHN elemental analyses were performed with standard microanalysis procedures using an Elementar Vario Micro Cube CHN analyzer. The infrared (IR) absorption spectra for **1** and **2** were collected on a Thermo Scientific Nicolet iN10 MX FT-IR microscope in transmission mode in the range of 3800−670 cm^{-1} for selected single crystals placed on a CaF_2 window, while the analogous spectra for ligands dppmO_2 and Me-dppmO_2 were collected on an FT-IR Thermo Scientific Nicolet 6700 spectrometer (measurements were made in the range of 4000−400 cm^{-1} but shown in the manuscript in the identical range as used for the compounds **1** and **2**, see Fig. S1). Thermogravimetric (TG) measurements were performed on a NETZSCH TG 209 F1 Libra apparatus under inert gas at a heating rate of 1 °C·min^{-1} in the temperature range of 20–420 °C. Differential scanning calorimetry (DSC) measurements were performed using a NETZCH DSC214 POLYMA calorimeter at the sweeping rates of 2, 5, and 10 °C·min^{-1}, in the temperature range of (–100)–0 °C and (–100)–100 °C, under dry inert gas (nitrogen) flow, for the sample placed in a concave Al pan with a pierced lid. Mass spectra were acquired using the Shimadzu HPLC LCMS-2020 system with electrospray (ESI) ionization mode and a single quadrupole mass spectrometry. The sample was prepared in MeOH.

The second harmonic generation (SHG) experiment was carried out on a homemade optical setup using a 1040 nm femtosecond laser as an excitation light source.[S6] To verify the SHG nature of the observed optical output under this excitation, the power and wavelength dependences were investigated. To quantify the SHG intensities measured in this setup, a potassium dihydrogen phosphate (KDP) was employed as a reference sample. For the *in situ* SHG measurements upon the variable humidity and temperature conditions, the pellet sample was placed in the Oxford Instruments MicrostatHe cryostat and properly conditioned by the related gas and cryo-stage system.

PFM studies were conducted with a commercially available scanning probe microscope (Asylum MFP-3D) with a Pt/Ir-coated Si tip (NANOSENSORS, PPP-NCSTPt; the typical spring constant is ~7.4 N/m). To achieve a good signal-to-noise ratio, we employed the dual-frequency resonance-tracking technique.[S7] Pyroelectric current measurements of **1** were performed on the selected single crystal, placed on the sapphire plate on its *b*-plane, and connected gold wires (25 μm diameter) with silver paste along perpendicular with the silver paste along *a*-axis (longer direction) and *c*-axis (shorter directions) in the *b*-plane (Fig. S14). Succinic acid diethyl ester was used as a solvent for silver paste. Temperature was controlled in a commercial cryostat. Pyroelectric current was measured with an electrometer (Keithley, 651B) during a temperature sweeping between ca. 215 K and ca. 255 K.

Room-temperature solid-state UV-vis absorption spectra were measured in the range of 200−700 nm on a Shimadzu UV-3600i plus spectrometer using the thin films of powder samples of obtained compounds inserted

between two quartz plates. Solid-state photoluminescent properties were measured using an FS5 spectrofluorometer (Edinburgh Instruments) equipped with an Xe 150 W arc lamp as an excitation source and a Hamamatsu photomultiplier of the R928P type as a detector. Emission lifetime measurements were conducted on the FS5 spectrofluorometer using a time-correlated single photon counting method with a 5 W microsecond flashlamp. The emission and excitation spectra for free **dppmO$_2$** and **Me-dppmO$_2$** molecules at room temperature and 77 K were collected using a liquid nitrogen dewar module (SC-70) with a sample placed on the bottom of a quartz tube. The temperature-dependent emission and excitation spectra, as well as *T*-variable emission lifetime measurements, for compound **1** were collected using a CS204SI-FMX-1SS cooling power optical helium cryostat equipped with a DE-204SI closed cycle cryocooler (cold head), a water-cooled He compressor (ARS-4HW model), and a model 335 cryogenic temperature controller attached to the FS5 spectrofluorometer through an SC-80 Cryostat Module. For all types of temperature-dependent photoluminescence studies, freshly prepared polycrystalline ground samples were packed in thin heat-sealed PE foil (0.02 mm thickness), covered by a minimal amount of silicon grease (type C), and placed between two quartz plates. This hampers the possible dehydration that could have occurred upon heating above room temperature under vacuum conditions, excluding also the effect of relative humidity from consideration for these studies.

The humidity-dependent photoluminescence studies of **1**, including emission spectra and emission lifetime measurements, were performed on the polycrystalline sample using a homemade setup adapted to the mentioned FS5 spectrofluorometer (Edinburgh Instruments). This experimental setup, whose essential part is visualized in Scheme S1, employs a front-face sample holder module commercially available as the accessory for the mentioned spectrofluorometer (Horizontal, SC-15 module, Edinburgh Instruments). In this module, the excitation light coming from the excitation monochromator of the spectrofluorometer is directed by two mirrors onto the powder tray (made of spectralon) enabling placing the free powder of the sample in the horizontal position. Then, the emission light is gathered and directed toward the emission monochromator of the spectrofluorometer using two other mirrors. Therefore, with this sample holder module, it is achievable to measure photoluminescence for the freely lying powder sample without the risk of undesirable movement of the sample during the measurements which is crucial for studying the externally-stimulated variation in the overall emission intensity (such studies were performed for the investigated material). This commercially available sample holder module was modified by covering its part containing the powder tray with a Plexiglass®-made chamber equipped with the quartz-plate-made window (on the top) enabling the undisturbed pathway for the excitation and emission lights. The photo of this additional chamber incorporating the powder tray for the sample is presented in Scheme S1. The cover of this chamber is mobile as it is attached to the rest of the chamber using four pairs of magnets. It enables the convenient placement of the powder sample, simultaneously keeping the whole chamber relatively leak-tight. In addition, this sample chamber has two holes (on the front side) where the connectors with a humidity sensor and a humidity generator are installed. The first one enables the detection of the relative humidity in the sample chamber in the vicinity of the sample. The second one enables the introduction of the gas (argon) of the defined relative humidity (RH) inside the sample chamber. The gas of the defined RH was ensured by the HG-100 RH humidity generator (L&C Science and Technology). The flow of the gas was maintained as the sample chamber was close tightly enough to keep the constant RH value inside whereas the controlled leakage of the gas through the hole with the connector to the humidity sensor was left. Such a system enables *in situ* photoluminescence studies in the general range of 15–90% of RH; however, the exact value of the lowest accessible RH value which was ensured to be stable depends on the humidity conditions in the laboratory, varying between ca. 15% and 22%. For the emission spectra measurements presented in this article, the range of ca. 17–90% RH was accessible, while for the emission lifetime measurements, the range of ca. 20–87% RH was found. These RH-dependences of emission characteristics were gathered for two consecutive cycles of water vapor sorption/desorption in the case of emission spectra and the single cycle of sorption/desorption in the case of emission lifetimes. The RH changes were realized in the ca. 10% steps; for each step, the sample was conditioned at the specific RH value until it was constant for at least 30 minutes.

Then, the optical measurements were conducted. The detected RH values were dependent on the experiment and did not correspond to the exact 10%-of-RH steps which was due to the equilibrium between the leakages of humidity from the sample chamber and the gas flow from the humidity generator. However, the determined RH values are precise and correspond to the real conditions around the investigated powder sample as the humidity sensor was placed very close to the sample and the RH value was stable before and during the optical measurement. Absolute emission quantum yields (QYs) were determined by a direct excitation method using an integrating sphere module (SC-30) and barium sulfate as the reference material.[S8] Emission background corrections were performed within the Fluoracle software (Edinburgh Instruments).

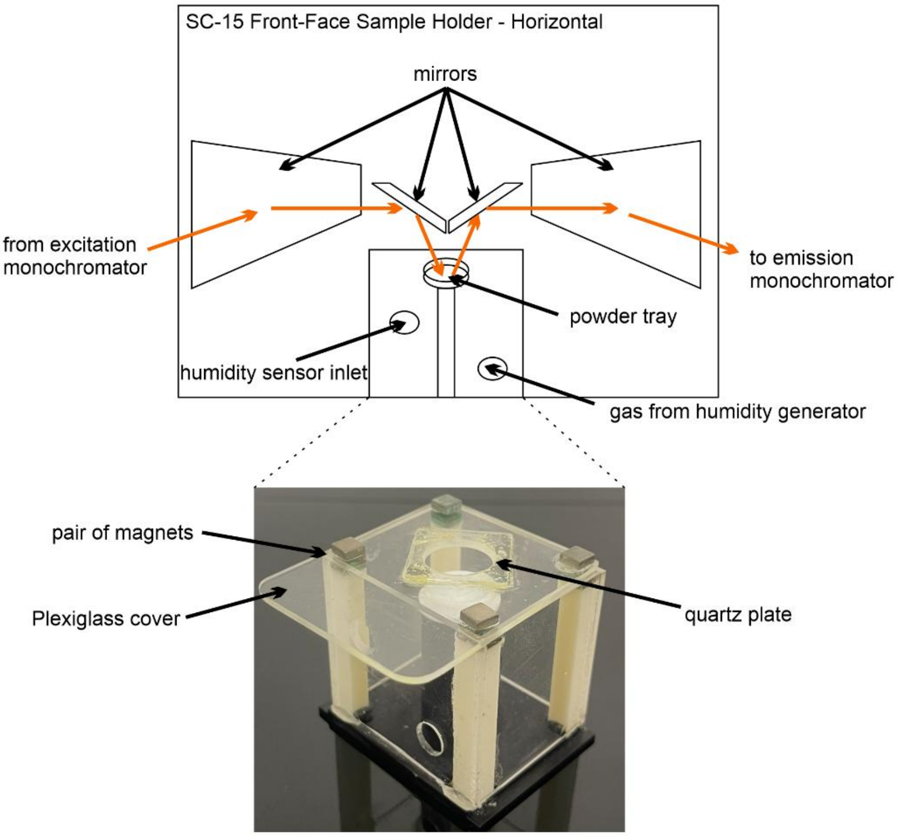

Scheme S1 The schematic visualization of the homemade experimental setup used for humidity-variable photo-luminescence studies performed for the polycrystalline sample of **1**. In the top part, the scheme of the whole applied sample holder module is presented while the the bottom part contains the photo of the smaller chamber where the sample was kept in the conditions of stable and precisely measured relative humidity (see the text above for technical details). The orange arrows represent the pathway for the excitation and emission lights.

The water sorption isotherms were measured at 298 K with a DVS method using an SMS DVS Resolution apparatus (Dual Vapor Gravimetric Sorption Analyzer) in the range of 0% < p/p_0 (vapor partial pressure) < 90%. Sorption measurements were conducted for the powder sample of **1** (with $1H_2O$ per the formula unit stable at ambient conditions), which before the measurement was stabilized at 0% RH (relative humidity) under dry nitrogen flow to obtain a stable mass. Every measurement step was performed until a stable sample mass was achieved (dm/dt = 0.001 mg·min^{-1}). Each measurement started at p/p_0 = 0%; thus, a single closed cycle consisted of sorption followed by desorption.
Dielectric measurements were performed using a Novocontrol Concept 11 dielectric spectrometer on the pellet samples of the investigated compound. The samples' pellets used for the measurements were prepared by pressing respective powder samples under 3.3 tons. The following metric parameters of the pellets were achieved: 5 mm of diameter and 1.02 mm of thickness for **1**, 5 mm of diameter and 0.95 mm of thickness for $\mathbf{1}^{\mathbf{reh}}$ (a rehydrated form

of **1**), 5 mm of diameter and 0.61 mm of thickness for **2**, 5 mm of diameter and 0.61 mm of thickness for $\mathbf{2}^{reh}$ (a rehydrated form of **2**). The obtained pellets were put between two gold-coated copper plates mounted in the sample chamber of the spectrometer. The dehydrated samples were prepared from the respective as-synthesized ones (**1** and **2**) by the *in situ* dehydration in the spectrometer. We checked that this dehydration demands the heating to 353 K as the pellet sample, due to the dense packing of crystallites, is much harder to dehydrate than the single-crystal or free-powder samples (which can be dehydrated in the dry inert gas atmosphere even at room temperature). After achieving the dehydration state of the pellet sample, visible in the disappearance of the dielectric relaxations characteristic of the hydrated phase, we attempted to rehydrate the same sample. We checked that it could not be done by the simple exposition of the pellet sample to the air atmosphere (ambient laboratory conditions close to room temperature with the RH close to 30%). The efficient rehydration (visible by the recovery of the dielectric relaxations) was done by grinding the pellet sample of the dehydrated phase and the exposition of the resulting free-powder sample for a few hours to the air atmosphere (the ambient laboratory conditions mentioned above). Then, the pellet of such a rehydrated phase was prepared and its dielectric response was investigated. Even though the dehydration of the pellet appeared to be much more difficult than the single-crystal or free-powder samples, we limited the temperature range for measuring the dielectric relaxations to 293 K. All these precautions resulted in the good stability of the hydrated phase during the dielectric studies, especially since the flow of dry inert gas (nitrogen, used also for the cooling in the dielectric spectrometer) is much weaker in this apparatus than in the case of SC-XRD diffractometer where the dehydration at room temperature was performed. No signs of dehydration during the dielectric studies in the 173–293 K range appear as depicted by the repeatability of the dielectric response upon cycles of cooling-heating (see Fig. S41).

Investigation of magnetic properties was performed using a Quantum Design MPMS-3 Evercool magnetometer. In general, for magnetic studies, the powder samples of the investigated compound were packed in a polycarbonate capsule, covered by paraffin oil and cotton wool. The sample of **1** was directly packed following this method at ambient laboratory conditions (RH of 23%). The $\mathbf{1}^{deh}$ sample was first packed in the capsule and dehydrated inside the SMS DVS instrument at 0% RH under dry nitrogen flow, then quickly covered by paraffin oil and cotton wool and then inserted into the magnetometer. The $\mathbf{1}^{HH}$ (i.e., the high-humidity sample of **1**) was packed in the capsule and stabilized in the SMS DVS instrument at 90% RH until the stable mass was reached, then quickly covered by paraffin oil and cotton wool, and then measured. Each of these samples was inserted into the sample chamber of the magnetometer which was quickly purged, and then the magnetometer was immediately cooled down to 10 K. Thus, the exposition of the sample to air was very short (several seconds for packing the sample into the capsule, checking the weight, and covering with paraffin oil) while the exposition to the vacuum conditions in the squid magnetometer around room temperature was also very short (a few minutes of purging the sample chamber and cooling below 300 K). After that, the *ac* magnetic measurements at low temperatures (below 5 K) were first performed, which were followed by the magnetization versus magnetic field studies at 1.8 K. At the end of the measurement sequence, the magnetization versus temperature characteristics upon heating from 1.8 to 300 K were gathered. All these procedures were done to stabilize the samples of **1** with three different contents of water molecules of crystallization and reliably measure their magnetic properties, thus avoiding the undesirable dehydration during packing of the sample and inserting it into the magnetometer, as well as upon further magnetic measurements. In general, we tested that the paraffin oil is a sufficient protectant to exclude the eventual solvent exchange under air or vacuum at room temperature, at least within the scale of minutes which is sufficient to prepare the system for the low-temperature studies (where the dehydration is fully blocked). In our studies, we did not detect any signs of solvent exchange for the sample treated as described. In ac magnetic studies, we observe single relaxation characteristics suggesting the presence of single pure phases of the specific solvent content without the impurity from the others. Moreover, the χT versus T dependences measured upon heating did not reveal any discrepancies at high temperatures close to room temperature suggesting that covering the sample with paraffin oil sufficiently protects the samples from the solvent content change. The sample of reference compound

4 was similarly packed at ambient laboratory conditions (RH of ca. 28%). Diamagnetic corrections from the sample, the paraffin oil, and the sample holder were taken into account.

Calculations and advanced data analyses

Continuous Shape Measure (CShM) analysis for metal complexes in **1** and **2** was performed using a SHAPE software ver. 2.1.21.[S9] The detailed analysis of *ac* magnetic and dielectric data, including the determination of magnetic or dielectric relaxation times from the simultaneous fitting of all *ac* magnetic or dielectric characteristics (frequency dependences of out-of-phase and in-phase magnetic susceptibilities/dielectric permittivities together with the related Argand plots) using models for complex-valued *ac* susceptibility/permittivity at the indicated conditions of *dc* magnetic/electric field and temperature, as well as further determination of final parameters of operating relaxation processes (in the case of magnetic data) from the simultaneous 3-D fitting of temperature- and field-dependences of relaxation times, was performed using the relACs program.[S10] It was designed to be used for the *ac* magnetic data; however, it could be also efficiently used for the analysis of analogous *ac* data of dielectric measurements. For computational details regarding the performed *ab initio* calculations see the Comment on theoretical calculations of optical transitions in Mn(II) complexes in **1** (Fig. S32–S35 and S66, as well as Tables S14–S22 and S35–S38).

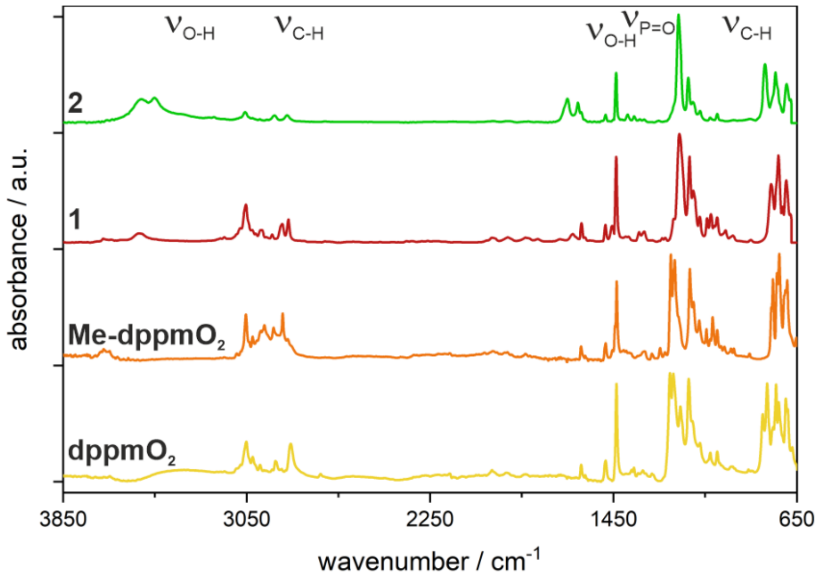

Fig. S1 Infrared (IR) absorption spectra of the selected crystals of **1** and **2**, compared with the analogous spectra of **dppmO$_2$** and **Me-dppmO$_2$** ligands, all collected in the 3850–650 cm^{-1} range. The representative vibrations responsible for the main absorption bands are indicated on the graph.

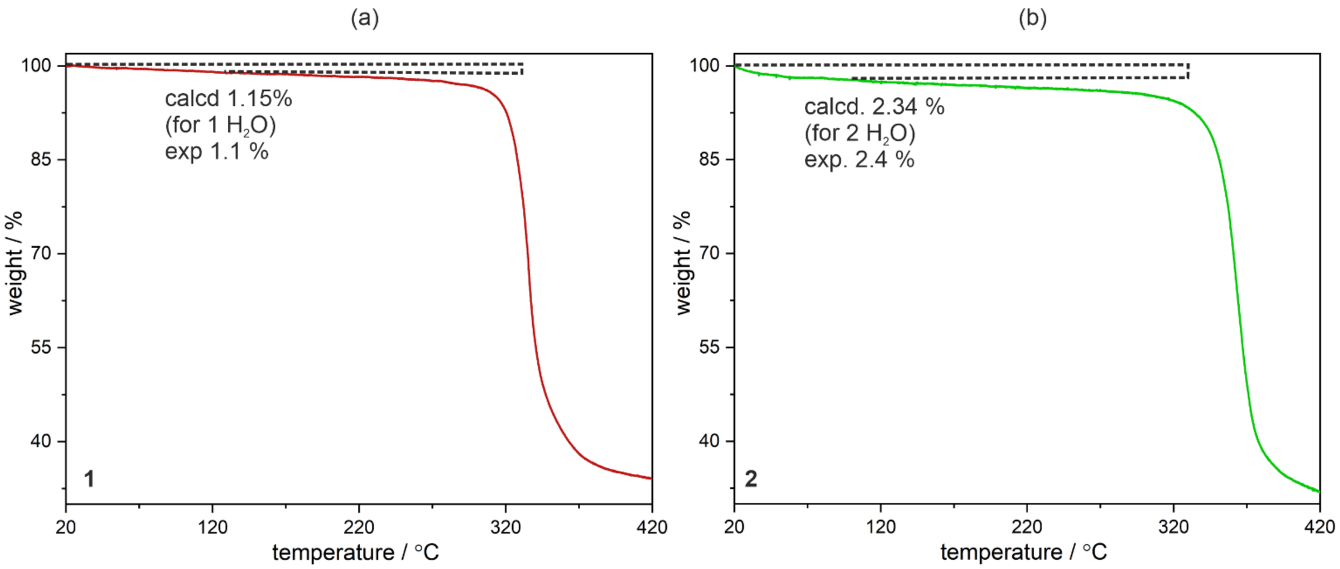

Fig. S2 Thermogravimetric (TG) curves of **1** (a) and **2** (b), collected in the 20–420 °C temperature range. The steps related to the loss of water molecules of crystallization were depicted on the graph (see Experimental section for details).

Table S1 Crystal data and structure refinement parameters for **Me-dppmO$_2$**, **1** (at various conditions), and **2**.

Compound	**Me-dppmO$_2$**	**1^{100K}**	**1^{270K}**	**$1^{deh,300K}$**	**$1^{deh,330K}$**	**$1^{deh,100K}$**	**2**
Formula	$C_{26}H_{24}O_2P_2$	$C_{78}H_{74}Cl_4Mn_2O_7P_6$	$C_{78}H_{74}Cl_4Mn_2O_7P_6$	$C_{78}H_{72}Cl_4Mn_2O_6P_6$	$C_{78}H_{72}Cl_4Mn_2O_6P_6$	$C_{78}H_{72}Cl_4Mn_2O_6P_6$	$C_{79}H_{72}Cl_{14}Mn_2N_2O_6P_6$
M_W / g·mol^{-1}	430.39	1560.87	1560.87	1542.82	1542.85	1542.85	1582.88
T / K	100(2)		270(2)	300(2)	330(2)	100(2)	
λ / Å	0.71073 (Mo Kα)						
Crystal system	monoclinic	monoclinic	monoclinic	monoclinic	monoclinic	monoclinic	monoclinic
Space group	$P2_1/n$	Cc	Cc	Cc	Cc	Cc	$P2_1/c$
a / Å	11.443(2)	11.2956(8)	11.3782(7)	11.3912(7)	11.4017(7)	11.2747(7)	13.0568(5)
b / Å	11.3282(13)	26.7171(18)	27.1370(15)	27.2867(18)	27.3917(17)	26.9136(19)	19.8458(8)
c / Å	127.464(3)	25.3047(17)	25.3156(14)	25.2528(16)	25.2074(16)	25.1091(17)	29.0464(12)
α / °	90	90	90	90	90	90	90
β / °	102.219(4)	100.865(2)	100.190(2)	100.008(2)	99.877(2)	100.442(2)	91.420(2)
γ / °	90	90	90	90	90	90	90
V / Å^3	2212.6(6)	7499.7(6)	7693.4(8)	7729.8(8)	7755.9(8)	7493.0(9)	7524.3(5)
Z	4	4	4	4	4	4	4
Density / g·cm^{-1}	1.292	1.382	1.348	1.326	1.321	1.368	1.397
Abs. coeff. / cm^{-1}	0.6938	0.660	0.643	0.639	0.637	0.659	0.659
F(000)	904	3224	3204	3184	3184	3184	3264
Θ range / °	2.379–26.372	2.346–25.027	2.219–25.026	2.752–25.025	2.214–25.026	1.986-25.026	2.341–25.027
Limiting indices	−14 < h < 14<br>−14 < k < 13<br>−21 < l < 21	−13 < h < 13<br>−31 < k < 31<br>−30 < l < 30	−13 < h < 13<br>−32 < k < 32<br>−30 < l < 30	−13 < h < 13<br>−32 < k < 32<br>−30 < l < 30	−13 < h < 13<br>−32 < k < 32<br>−29 < l < 29	−13 < h < 13<br>−32 < k < 32<br>−29 < l < 29	−15 < h < 15<br>−23 < k < 23<br>−34 < l < 34
Collected reflections	21459	39762	42081	41055	42207	142283	88987
R_{int}	0.0412	0.0315	0.0307	0.0327	0.0306	0.0357	0.0710
Completeness	0.999	0.998	0.998	0.997	0.997	0.999	0.999
Flack parameter	-	0.055(5)	0.066(5)	0.090(5)	0.063(5)	0.022(12)	0.056(4)
Data/restrains /parameters	4514/0/271	13197/0/880	13465/6/880	13501/2/865	13554/2/865	13263/2/865	13290/42/894
GOF on F^2	1.102	1.131	1.074	1.055	1.057	1.019	1.146
R_1 (for [$I > 2\sigma(I)$]); wR_2 (for all data)	R_1 = 0.0453<br>wR_2 = 0.1041	R_1 = 0.0423<br>wR_2 = 0.0971	R_1 = 0.0434<br>wR_2 = 0.1108	R_1 = 0.0044<br>wR_2 = 0.1139	R_1 = 0.0435<br>wR_2 = 0.1116	R_1 = 0.0357<br>wR_2 = 0.0907	R_1 = 0.0680<br>wR_2 = 0.1533
Largest diff. peak and hole /e·Å^{-3}	0.325/−0.41	0.814/−0.499	0.709/−0.369	0.705/−0.298	0.671/−0.302	0.329/−0.314	1.977/−0.662

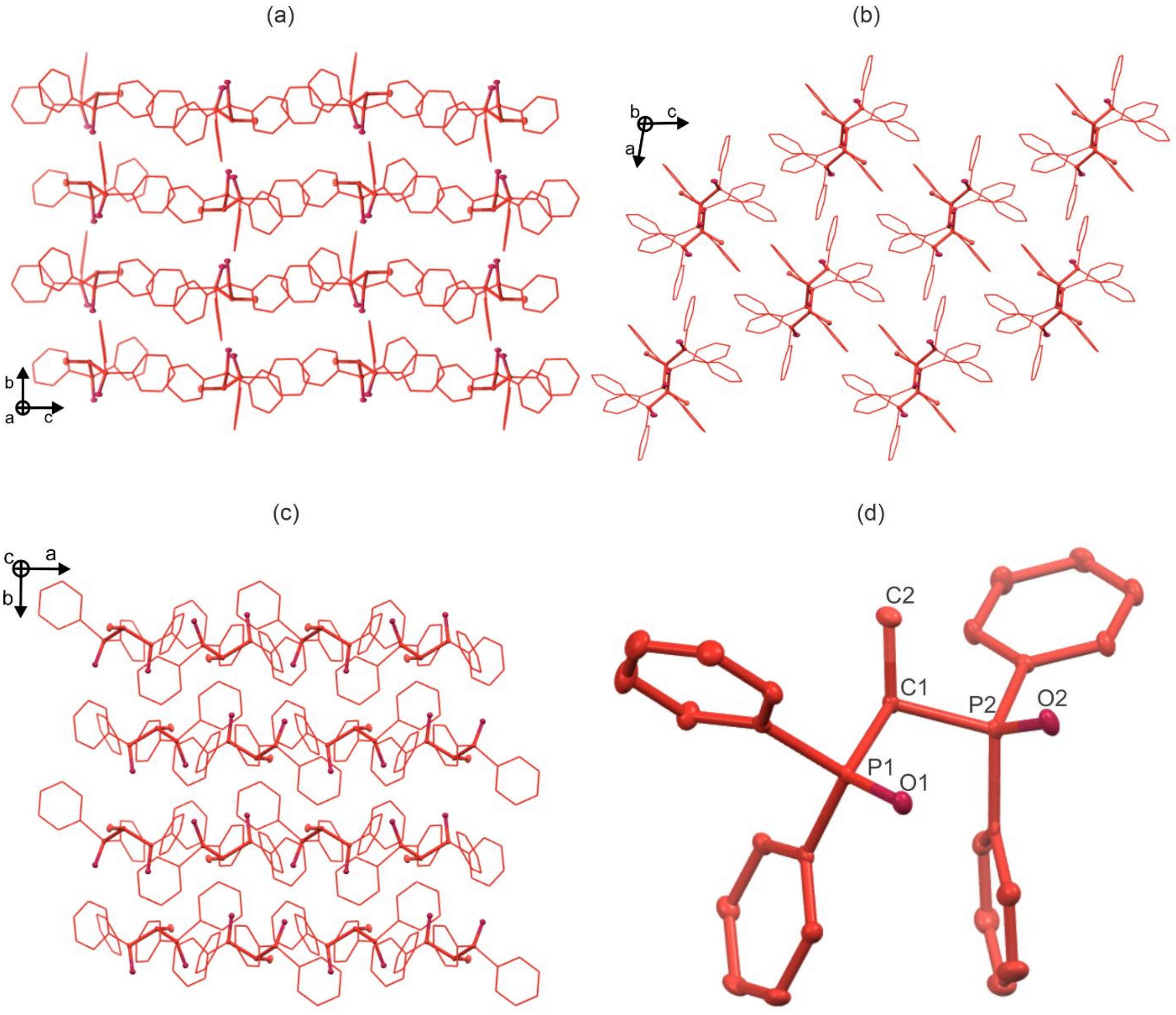

Fig. S3 The representative views of the crystal structure of **Me-dppmO₂** along the main **a**, **b**, and **c** crystallographic axes (a−c, respectively), and the asymmetric unit with the labeling scheme for selected symmetrically independent atoms (d). Thermal ellipsoids were presented at the 50% probability level. Hydrogen atoms were omitted for clarity.

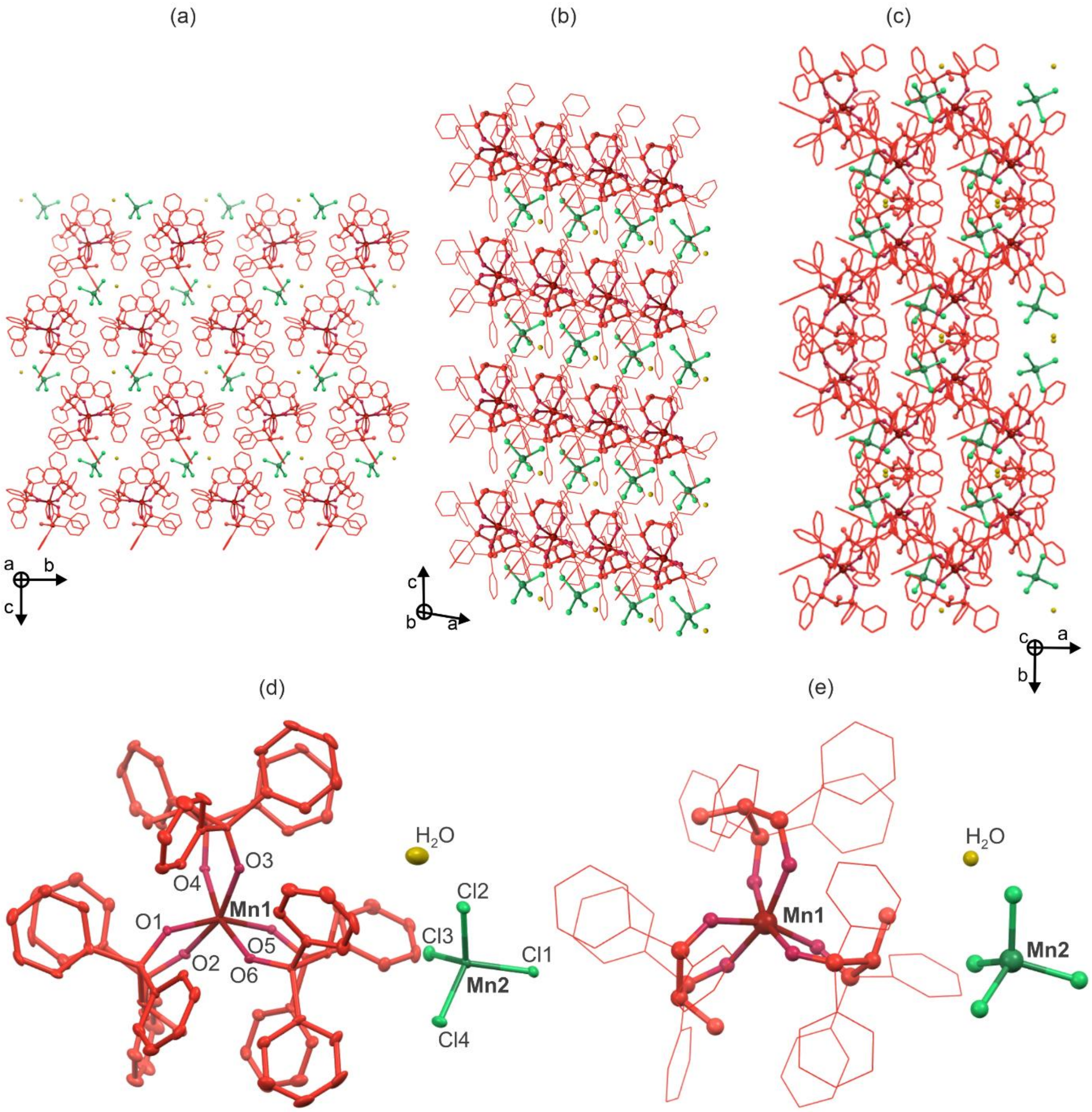

Fig. S4 The representative views of the crystal structure of **1**^{100K} along the main **a**, **b**, and **c** crystallographic axes (a−c, respectively), the asymmetric unit containing atoms with their thermal ellipsoids as well as the labeling scheme for selected symmetrically independent atoms (d), and the molecular building unit with the underlined coordination environment around Mn(II) centers, including the polar arrangement around the Mn1 one (e). Thermal ellipsoids in (d) are presented at the 50% probability level. Hydrogen atoms were omitted for clarity. Colors: red with various hues = Mn1 complexes with **Me-dppmO$_2$** ligands attached, green with hues = Mn2 complexes, yellow = water of crystallization.

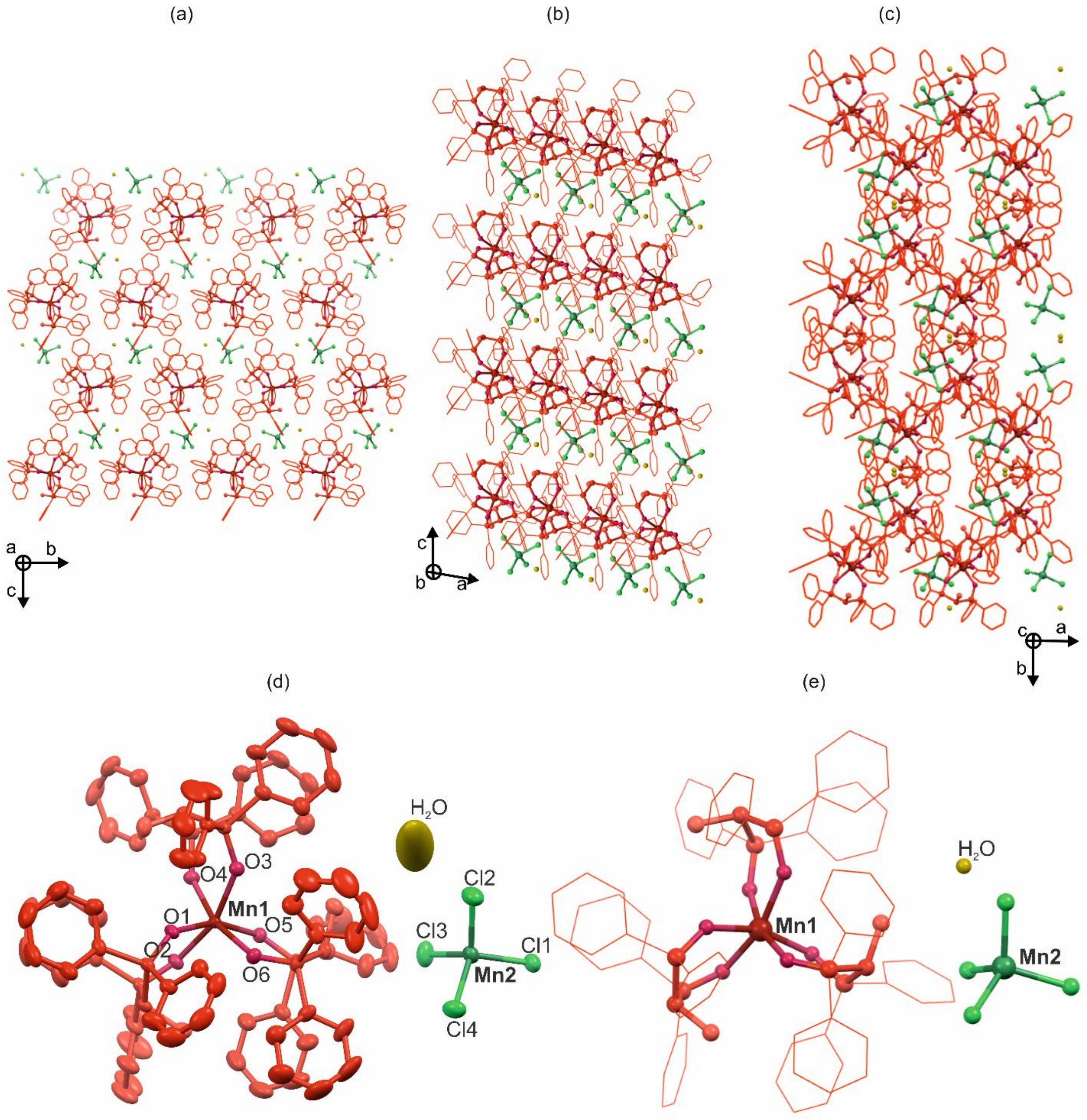

Fig. S5 The representative views of the crystal structure of $\mathbf{1}^{270K}$ along the main **a**, **b**, and **c** crystallographic axes (a−c, respectively), the asymmetric unit containing atoms with their thermal ellipsoids as well as the labeling scheme for selected symmetrically independent atoms (d), and the molecular building unit with the underlined coordination environment around Mn(II) centers, including the polar arrangement around the Mn1 one (e). Thermal ellipsoids in (d) are presented at the 50% probability level. Hydrogen atoms were omitted for clarity. Colors: red with various hues = Mn1 complexes with **Me-dppmO₂** ligands attached, green with hues = Mn2 complexes, yellow = water of crystallization.

Table S2 Selected detailed structure parameters of **1**^{100K}.

Selected bond lengths in **1**^{100K} / Å					
Mn1-O1	2.222(4)	Mn1-O4	2.165(4)	Mn2-Cl1	2.3689(17)
Mn1-O2	2.159(4)	Mn1-O5	2.185(4)	Mn2-Cl2	2.3574(18)
Mn1-O3	2.219(4)	Mn1-O6	2.199(4)	Mn2-Cl3	2.3674(18)
				Mn2-Cl4	2.3677(18)
Selected angles between bonds in **1**^{100K} / °					
O1-Mn1-O2	81.84(15)	O2-Mn1-O5	87.69(15)	O5-Mn1-O6	85.00(15)
O1-Mn1-O3	91.16(14)	O2-Mn1-O6	109.48(15)	Cl1-Mn2-Cl2	108.65(6)
O1-Mn1-O4	108.37(15)	O3-Mn1-O4	81.08(14)	Cl1-Mn2-Cl3	104.24(6)
O1-Mn1-O5	159.98(15)	O3-Mn1-O5	103.62(15)	Cl1-Mn2-Cl4	111.79(6)
O1-Mn1-O6	82.50(15)	O3-Mn1-O6	86.39(15)	Cl2-Mn2-C3	114.20(7)
O2-Mn1-O3	161.47(16)	O4-Mn1-O5	87.50(15)	Cl2-Mn2-Cl4	111.89(6)
O2-Mn1-O4	84.87(15)	O4-Mn1-O6	163.46(15)	Cl3-Mn2-Cl4	105.85(7)

Table S3 Selected detailed structure parameters of $\mathbf{1}^{270K}$.

Selected bond lengths in $\mathbf{1}^{270K}$ / Å					
Mn1-O1	2.214(4)	Mn1-O4	2.174(4)	Mn2-Cl1	2.365(2)
Mn1-O2	2.171(4)	Mn1-O5	2.187(4)	Mn2-Cl2	2.355(2)
Mn1-O3	2.218(4)	Mn1-O6	2.219(4)	Mn2-Cl3	2.363(2)
				Mn2-Cl4	2.3644(19)
Selected angles between bonds in $\mathbf{1}^{270K}$ / °					
O1-Mn1-O2	82.12(15)	O2-Mn1-O5	88.14(15)	O5-Mn1-O6	83.65(14)
O1-Mn1-O3	90.02(14)	O2-Mn1-O6	109.93(16)	Cl1-Mn2-Cl2	108.71(8)
O1-Mn1-O4	108.42715)	O3-Mn1-O4	80.80(14)	Cl1-Mn2-Cl3	104.15(8)
O1-Mn1-O5	159.47(15)	O3-Mn1-O5	104.50(14)	Cl1-Mn2-Cl4	111.64(8)
O1-Mn1-O6	82.75(15)	O3-Mn1-O6	86.78(14)	Cl2-Mn2-C3	114.77(9)
O2-Mn1-O3	160.30(16)	O4-Mn1-O5	88.43(15)	Cl2-Mn2-Cl4	111.24(8)
O2-Mn1-O4	84.60(15)	O4-Mn1-O6	163.12(14)	Cl3-Mn2-Cl4	106.16(8)

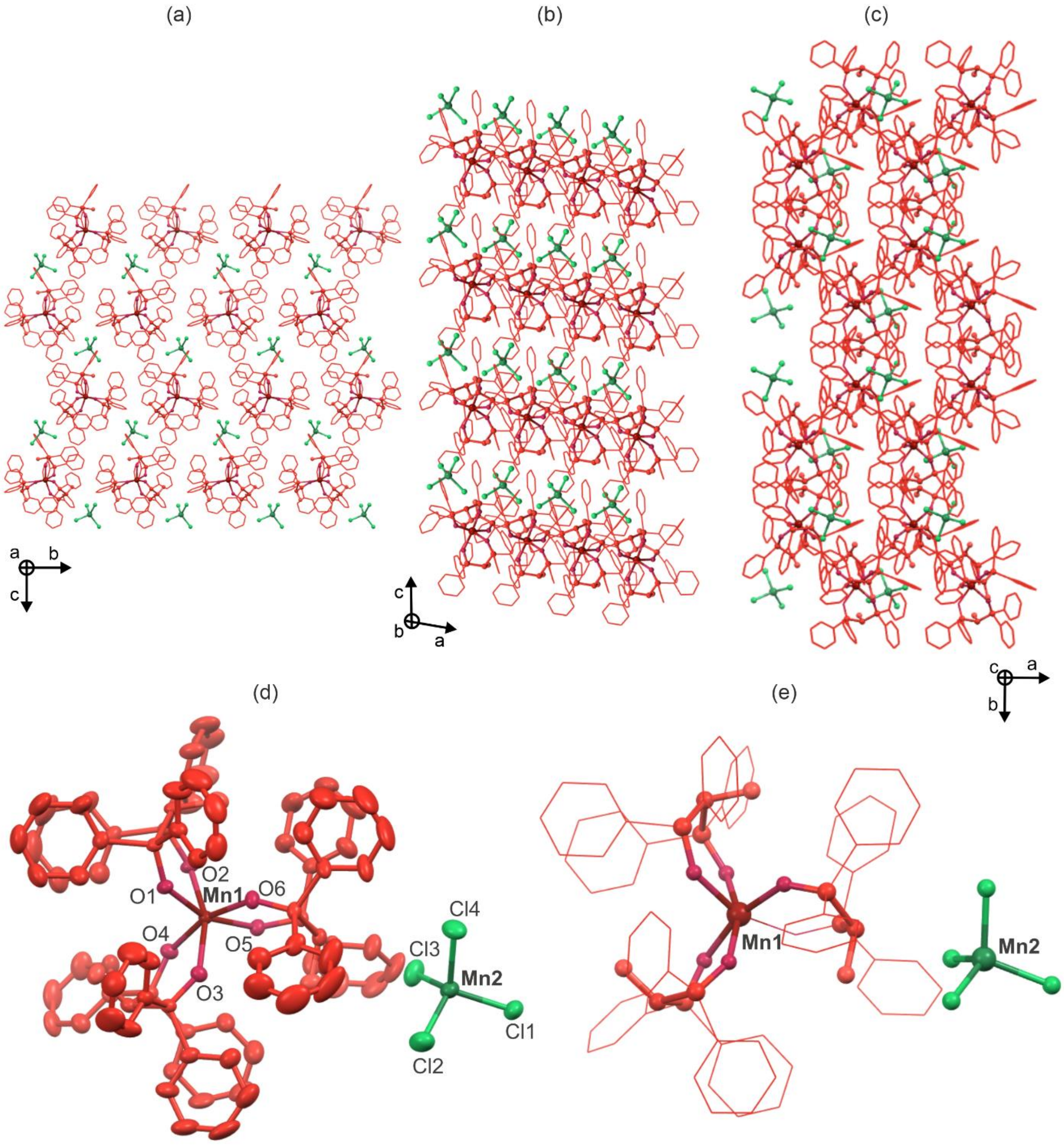

Fig. S6 The representative views of the crystal structure of $\mathbf{1}^{deh,300K}$ along the main **a**, **b**, and **c** crystallographic axes (a−c, respectively), the asymmetric unit containing atoms with their thermal ellipsoids as well as the labeling scheme for selected symmetrically independent atoms (d), and the molecular building unit with the underlined coordination environment around Mn(II) centers, including the polar arrangement around the Mn1 one (e). Thermal ellipsoids in (d) are presented at the 50% probability level. Hydrogen atoms were omitted for clarity. Colors: red with various hues = Mn1 complexes with **Me-dppmO$_2$** ligands attached, green with hues = Mn2 complexes.

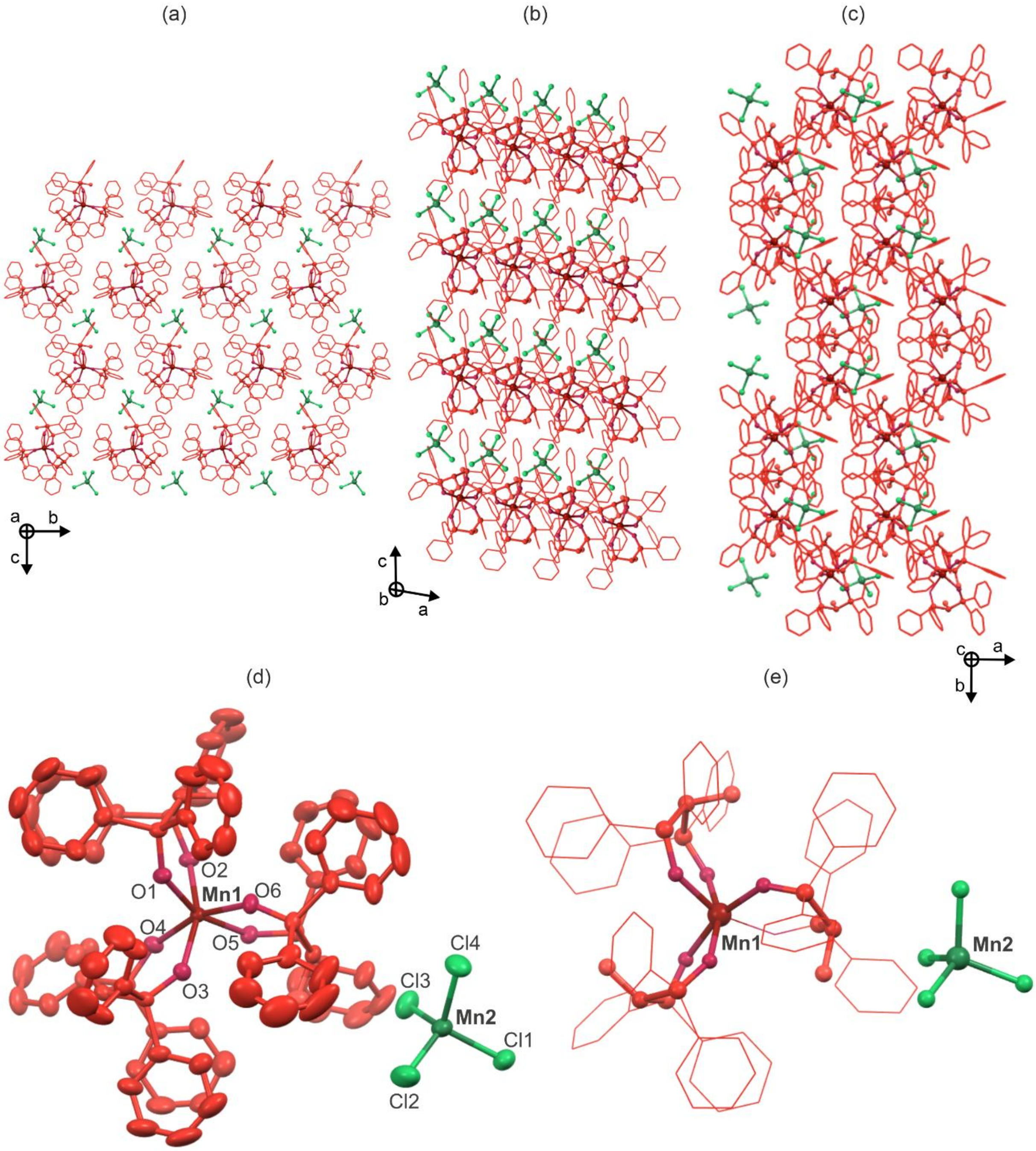

Fig. S7 The representative views of the crystal structure of **1**^{deh,330K} along the main **a**, **b**, and **c** crystallographic axes (a−c, respectively), the asymmetric unit containing atoms with their thermal ellipsoids as well as the labeling scheme for selected symmetrically independent atoms (d), and the molecular building unit with the underlined coordination environment around Mn(II) centers, including the polar arrangement around the Mn1 one (e). Thermal ellipsoids in (d) are presented at the 50% probability level. Hydrogen atoms were omitted for clarity. Colors: red with various hues = Mn1 complexes with **Me-dppmO$_2$** ligands attached, green with hues = Mn2 complexes.

Table S4 Selected detailed structure parameters of **1**^{deh,300K}.

Selected bond lengths in **1**^{deh,300K} / Å					
Mn1-O1	2.214(4)	Mn1-O4	2.172(4)	Mn2-Cl1	2.369(2)
Mn1-O2	2.173(4)	Mn1-O5	2.186(4)	Mn2-Cl2	2.346(2)
Mn1-O3	2.226(4)	Mn1-O6	2.226(4)	Mn2-Cl3	2.367(2)
				Mn2-Cl4	2.367(2)
Selected angles between bonds in **1**^{deh,300K} / °					
O1-Mn1-O2	82.29(15)	O2-Mn1-O5	88.35(15)	O5-Mn1-O6	83.34(15)
O1-Mn1-O3	89.52(15)	O2-Mn1-O6	109.95(16)	Cl1-Mn2-Cl2	108.97(8)
O1-Mn1-O4	108.76(15)	O3-Mn1-O4	80.83(14)	Cl1-Mn2-Cl3	103.70(9)
O1-Mn1-O5	159.44(15)	O3-Mn1-O5	104.76(15)	Cl1-Mn2-Cl4	111.16(8)
O1-Mn1-O6	82.74(15)	O3-Mn1-O6	86.91(15)	Cl2-Mn2-C3	115.69(10)
O2-Mn1-O3	159.98(16)	O4-Mn1-O5	88.38(15)	Cl2-Mn2-Cl4	111.14(9)
O2-Mn1-O4	84.55(15)	O4-Mn1-O6	162.98(15)	Cl3-Mn2-Cl4	105.94(9)

Table S5 Selected detailed structure parameters of $\mathbf{1}^{deh,330K}$.

Selected bond lengths in $\mathbf{1}^{deh,330K}$ / Å					
Mn1-O1	2.212(4)	Mn1-O4	2.172(4)	Mn2-Cl1	2.367(2)
Mn1-O2	2.176(4)	Mn1-O5	2.187(4)	Mn2-Cl2	2.339(3)
Mn1-O3	2.229(4)	Mn1-O6	2.224(4)	Mn2-Cl3	2.368(2)
				Mn2-Cl4	2.367(2)
Selected angles between bonds in $\mathbf{1}^{deh,330K}$ / °					
O1-Mn1-O2	82.38(15)	O2-Mn1-O5	88.45(15)	O5-Mn1-O6	82.85(14)
O1-Mn1-O3	89.27(14)	O2-Mn1-O6	110.08(15)	Cl1-Mn2-Cl2	109.00(9)
O1-Mn1-O4	109.01(15)	O3-Mn1-O4	80.65(14)	Cl1-Mn2-Cl3	103.48(9)
O1-Mn1-O5	159.17(14)	O3-Mn1-O5	105.01(14)	Cl1-Mn2-Cl4	110.81(8)
O1-Mn1-O6	82.82(15)	O3-Mn1-O6	87.12(14)	Cl2-Mn2-C3	116.12(11)
O2-Mn1-O3	159.60(15)	O4-Mn1-O5	88.55(15)	Cl2-Mn2-Cl4	111.25(10)
O2-Mn1-O4	84.53(15)	O4-Mn1-O6	162.72(14)	Cl3-Mn2-Cl4	105.90(9)

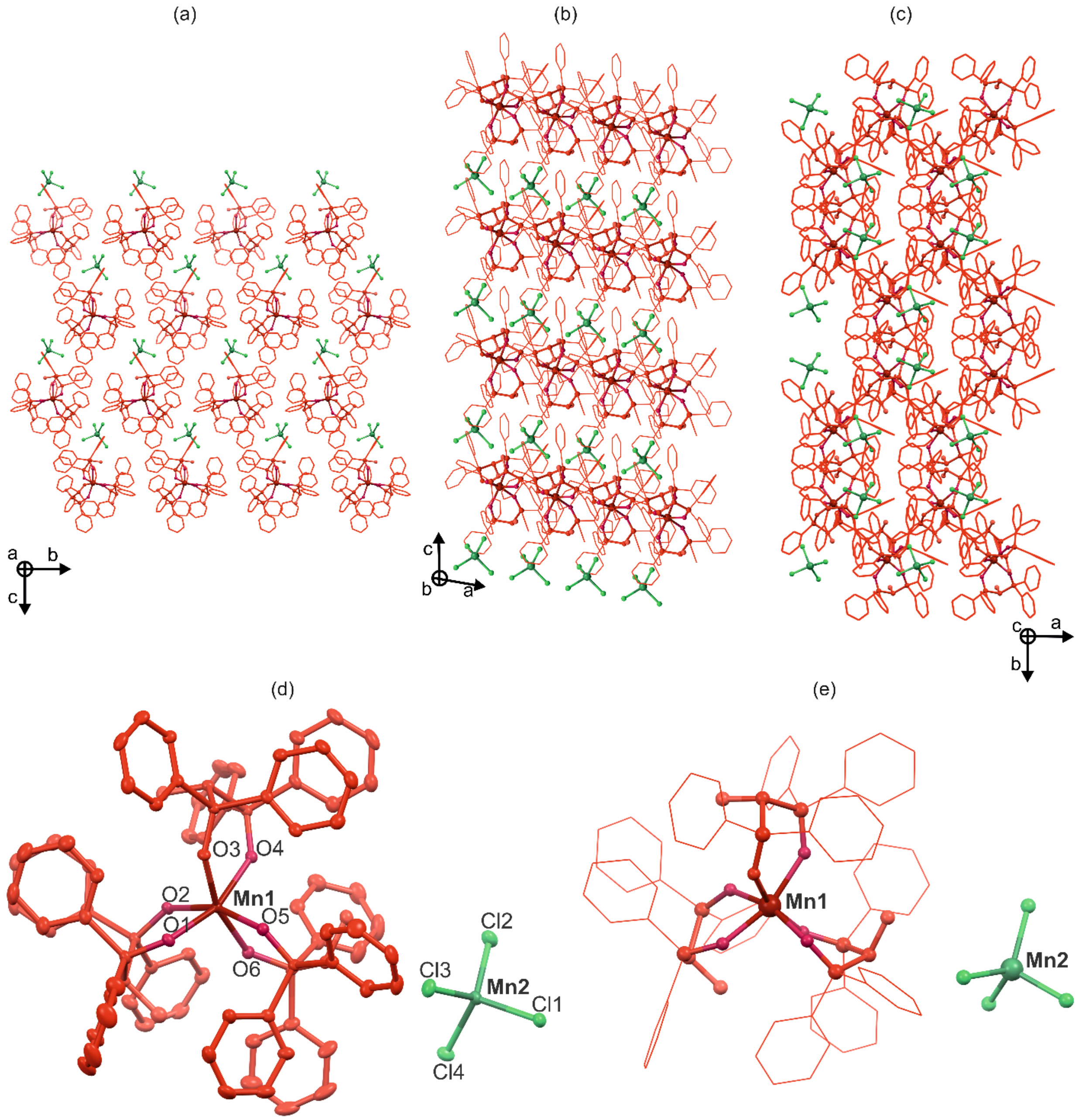

Fig. S8 The representative views of the crystal structure of $\mathbf{1}^{deh,100K}$ along the main **a**, **b**, and **c** crystallographic axes (a–c, respectively), the asymmetric unit containing atoms with their thermal ellipsoids as well as the labeling scheme for selected symmetrically independent atoms (d), and the molecular building unit with the underlined coordination environment around Mn(II) centers, including the polar arrangement around the Mn1 one (e). Thermal ellipsoids in (d) are presented at the 50% probability level. Hydrogen atoms were omitted for clarity. Colors: red with various hues = Mn1 complexes with **Me-dppmO$_2$** ligands attached, green with hues = Mn2 complexes.

Table S6 Selected detailed structure parameters of **1**^{deh,100K}.

Selected bond lengths in **1**^{deh,100K} / Å					
Mn1-O1	2.166(3)	Mn1-O4	2.216(3)	Mn2-Cl1	2.3709(13)
Mn1-O2	2.213(3)	Mn1-O5	2.198(3)	Mn2-Cl2	2.3393(14)
Mn1-O3	2.167(3)	Mn1-O6	2.190(3)	Mn2-Cl3	2.3635(14)
				Mn2-Cl4	2.3732(14)
Selected angles between bonds in **1**^{deh,100K} / °					
O1-Mn1-O2	82.43(11)	O2-Mn1-O5	82.63(12)	O5-Mn1-O6	84.50(12)
O1-Mn1-O3	85.08(12)	O2-Mn1-O6	160.45(11)	Cl1-Mn2-Cl2	109.08(5)
O1-Mn1-O4	161.58(12)	O3-Mn1-O4	81.20(11)	Cl1-Mn2-Cl3	103.40(5)
O1-Mn1-O5	108.94(12)	O3-Mn1-O5	163.38(11)	Cl1-Mn2-Cl4	110.79(5)
O1-Mn1-O6	87.94(11)	O3-Mn1-O6	87.23(12)	Cl2-Mn2-C3	116.80(6)
O2-Mn1-O3	108.72(12)	O4-Mn1-O5	86.75(11)	Cl2-Mn2-Cl4	111.34(5)
O2-Mn1-O4	90.37(11)	O4-Mn1-O6	103.54(11)	Cl3-Mn2-Cl4	105.10(5)

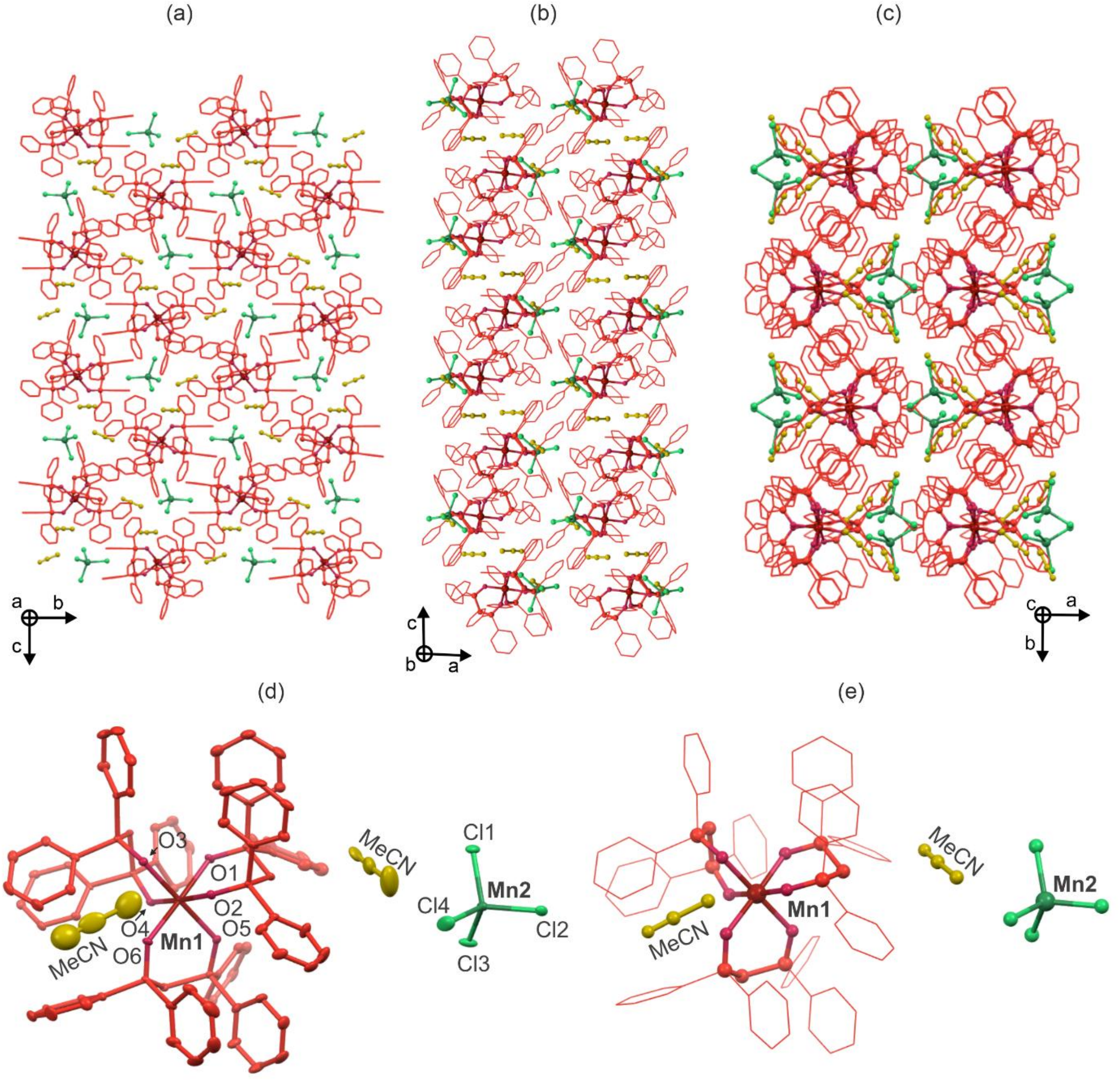

Fig. S9 The representative views of the crystal structure of **2** along the main **a**, **b**, and **c** crystallographic axes (a−c, respectively), the asymmetric unit containing atoms with their thermal ellipsoids as well as the labeling scheme for selected symmetrically independent atoms (d), and the molecular building unit with the underlined coordination environment around Mn(II) centers and solvent molecules of crystallization (e). Thermal ellipsoids in (d) are presented at the 50% probability level. Hydrogen atoms were omitted for clarity. Colors: red with various hues = Mn1 complexes with **dppmO$_2$** ligands attached, green with hues = Mn2 complexes, yellow = acetonitrile molecules of crystallization.

Table S7 Selected detailed structure parameters of **2**.

Selected bond lengths in **2** / Å					
Mn1-O1	2.170(3)	Mn1-O4	2.170(3)	Mn2-Cl1	2.3730(13)
Mn1-O2	2.160(3)	Mn1-O5	2.154(3)	Mn2-Cl2	2.3806(14)
Mn1-O3	2.151(3)	Mn1-O6	2.207(3)	Mn2-Cl3	2.3868(14)
				Mn2-Cl4	2.3476(15)
Selected angles between bonds in **2** / °					
O1-Mn1-O2	89.60(11)	O2-Mn1-O5	94.28(11)	O5-Mn1-O6	89.66(11)
O1-Mn1-O3	89.61(12)	O2-Mn1-O6	89.41(11)	Cl1-Mn2-Cl2	102.25(5)
O1-Mn1-O4	85.25(11)	O3-Mn1-O4	90.07(11)	Cl1-Mn2-Cl3	109.62(5)
O1-Mn1-O5	176.12(12)	O3-Mn1-O5	90.38(11)	Cl1-Mn2-Cl4	116.14(6)
O1-Mn1-O6	90.36(12)	O3-Mn1-O6	179.96(15)	Cl2-Mn2-C3	115.54(5)
O2-Mn1-O3	90.57(11)	O4-Mn1-O5	90.87(11)	Cl2-Mn2-Cl4	106.73(6)
O2-Mn1-O4	174.80(11)	O4-Mn1-O6	89.95(11)	Cl3-Mn2-Cl4	106.83(6)

Table S8 Results of Continuous Shape Measure (CShM) analysis for six- and four-coordinated Mn(II) complexes in compounds **1** and **2**.

Compound	CShM parameters for Mn1 centers (C.N. = 6)*					Geometry	CShM parameters for Mn2 centers (C.N. = 4)*			Geometry
	HP-6	PPY-6	OC-6	TPR-6	JPPY-6		SP-4	T-4	SS-4	
**1**^{100K}	31.733	19.842	**2.867**	6.563	23.675	**OC-6**	30.426	**0.160**	8.378	**T-4**
**1**^{270K}	32.268	19.881	**3.027**	6.289	23.749	**OC-6**	30.261	**0.166**	8.221	**T-4**
**1**^{deh,300K}	32.376	19.952	**3.074**	6.203	23.813	**OC-6**	39.095	**0.188**	8.011	**T-4**
**1**^{deh,330K}	32.562	19.941	**3.156**	6.093	23.815	**OC-6**	30.057	**0.198**	7.914	**T-4**
**1**^{deh,100K}	31.911	20.167	**2.805**	6.586	24.007	**OC-6**	29.799	**0.232**	7.770	**T-4**
**2**	33.236	29.063	**0.075**	15.436	32.618	**OC-6**	28.233	**0.373**	7.831	**T-4**

*Continuous Shape Measure (CShM) parameters:[S9,S11]

<u>six-coordinated complexes</u>

CShM HP-6 – the parameter related to the hexagon (D_{6h} symmetry)

CShM PPY-6 – the parameter related to the pentagonal pyramid (C_{5v})

CShM OC-6 – the parameter related to the octahedron (O_h)

CShM TPR-6 – the parameter related to the trigonal prism (D_{3h})

CShM JPPY-6 – the parameter related to the Johnson pentagonal pyramid (C_{5v})

<u>four-coordinated complexes</u>

CShM SP-4 – the parameter related to the square (D_{4h})

CShM T-4 – the parameter related to the tetrahedron (T_d)

CShM SS-4 – the parameter related to the seesaw or sawhorse (*cis*-divacant octahedron, C_{2v})

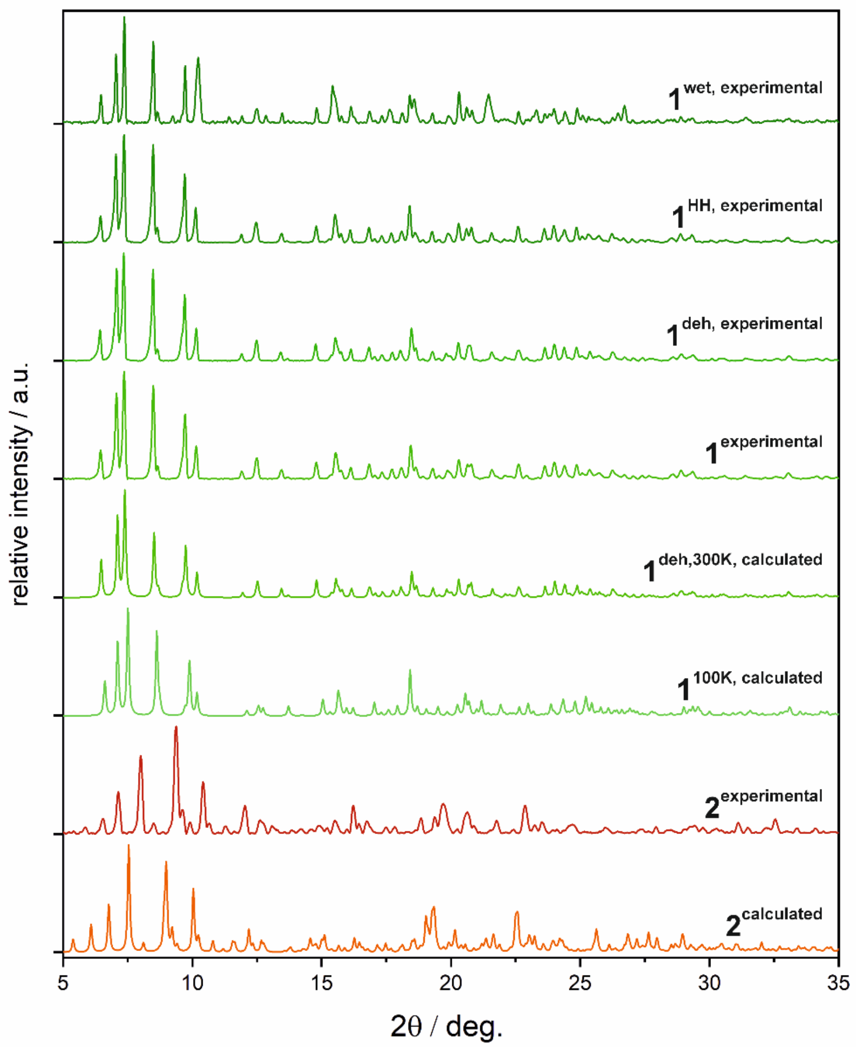

Fig. S10 Comparison of experimental (all experiments performed at room temperature) and calculated powder X-ray diffractograms (P-XRD) of compounds **1** and **2**, including (from top to bottom): (i) the experimental P-XRD pattern for the powder sample of **1** measured under the water solution (considered as the wet sample, abbrevatived here as $\mathbf{1}^{\text{wet, experimental}}$), (ii) the experimental P-XRD pattern for the high-humidity phase of **1** (HH phase as named for magnetic studies, see Fig. 10; abbreviated here as $\mathbf{1}^{\text{HH, experimental}}$; achieved by placing the air-dried sample of **1** in the open glass capillary, conditioning it at 90% RH for 24 hours, and closing the capillary before the measurement), (iii) the experimental P-XRD pattern for the dehydrated phase of **1** (the deh phase as named for other physical studies, see, e.g., Fig. 8 and 10; abbreviated here as $\mathbf{1}^{\text{deh, experimental}}$; achieved by placing the air-dried sample of **1** in the open glass capillary, conditioning it at the vacuum conditions for 12 hours, filling with argon, and closing the capillary before the measurement), (iv) the experimental P-XRD pattern for the air-dried hydrated phase of **1** (the air-stable phase of **1** placed in the glass capillary at ambient humidity conditions of ca. 40% RH; abbreviated here as $\mathbf{1}^{\text{experimental}}$), (v) the calculated P-XRD pattern based on the structural model of $\mathbf{1}^{\text{deh,300K}}$ (the dehydrated phase of **1** measured at 300(2) K, Table S1 and Fig. S6; abbreviated here as $\mathbf{1}^{\text{deh,300K, calculated}}$), (vi) the calculated P-XRD pattern based on the structural model of $\mathbf{1}^{\text{100K}}$ (the hydrated phase of **1** measured at 100(2) K, Table S1 and Fig. 1; abbreviated here as $\mathbf{1}^{\text{100K, calculated}}$), (vii) the experimental P-XRD pattern for the air-dried hydrated phase of the reference compound of **2** (the air-stable phase of **2** placed in the glass capillary at ambient humidity conditions of ca. 40% RH; abbreviated here as $\mathbf{2}^{\text{experimental}}$), and (viii) the calculated P-XRD pattern based on the structural model of **2** (measured at 100(2) K, Table S1 and Fig. S9; abbreviated here as $\mathbf{2}^{\text{calculated}}$).

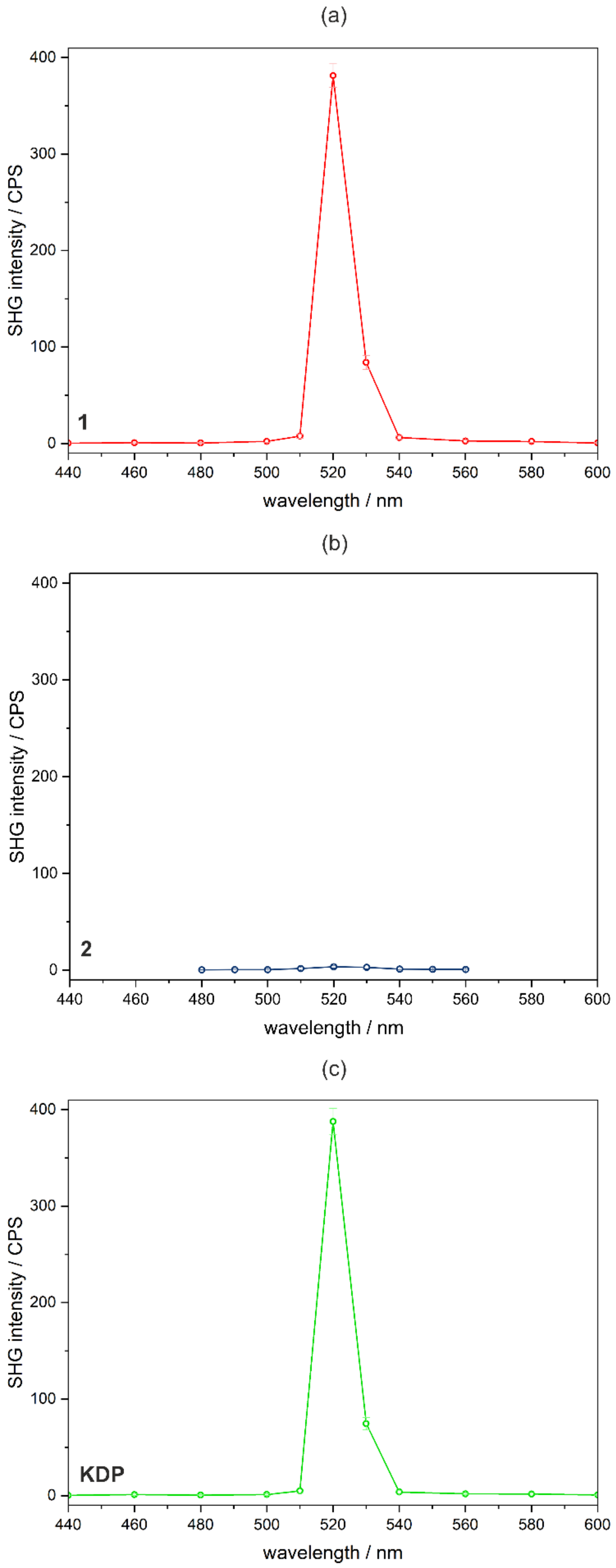

Fig. S11 Wavelength dependences of the SHG signal obtained under the 1040 nm irradiation for the powder samples of **1** (a), **2** (b), and the reference materials of potassium dihydrogen phosphate (**KDP**, c). The incident laser power used for this experiment reaches 0.897 $W\cdot m^{-2}$ for **1**, 0.138 $W\cdot cm^{-2}$ for **2**, and 0.080 $W\cdot m^{-2}$ for **KDP**.

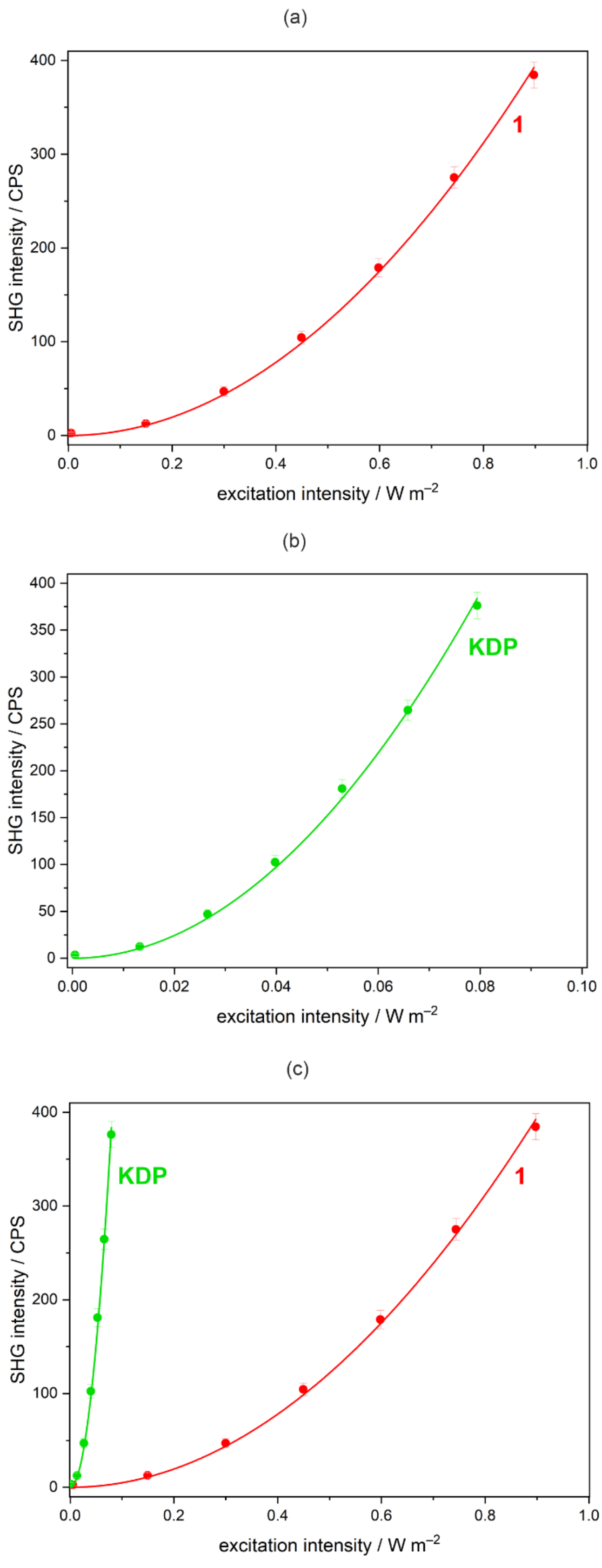

Fig. S12 Dependences of the SHG effect on the excitation intensity for the powder samples of **1** (a), and the reference material of **KDP** (b), as well as the comparison of the dependences for both samples (c). Colored points represent experimental data with the related intensity uncertainties. Solid lines correspond to the best-fit curves according to the quadratic equation pointing to the second harmonic (SHG) nature of emitted light.

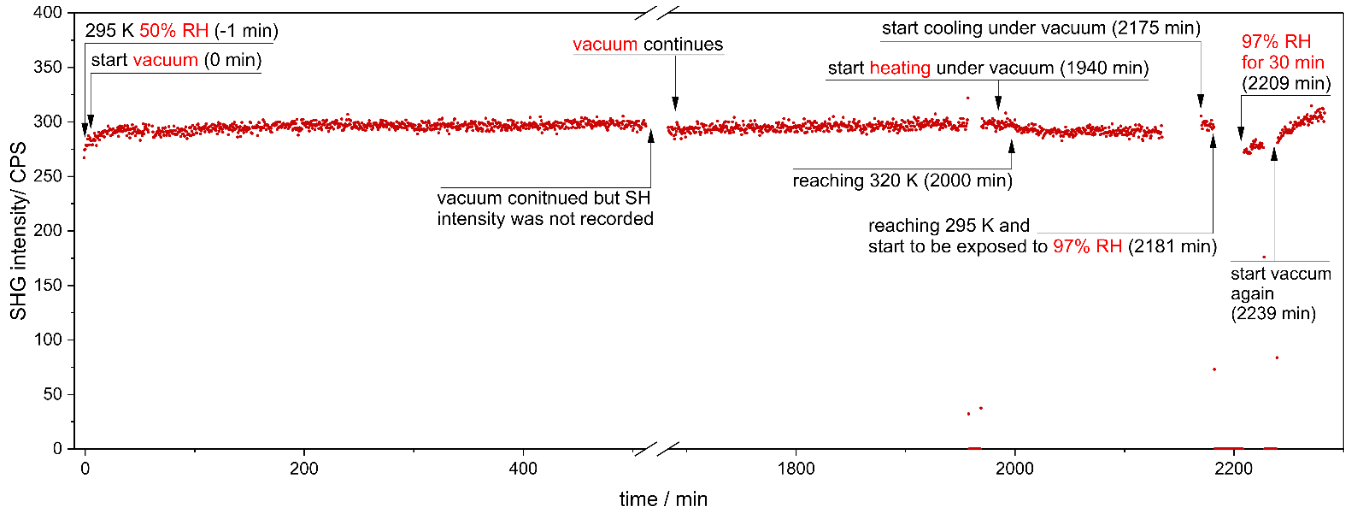

Fig. S13 The results of the *in situ* studies of the SHG effect intensity of the pellet sample of **1** under variable indicated relative humidity (RH) and temperature conditions. The results are presented on the scale of time of the performed experiment while the conditions' changes are depicted by the arrows and related texts. The incident laser power of the 1040 nm laser irradiation used for this experiment reached 0.13747 W·cm^{-2} and the 520 nm output light intensity was followed. The representative results of achieved SHG values are gathered in Table S9 below.

Table S9 Summary of the average SHG signals achieved in the *in situ* studies of the dependence of the SHG effect of **1** on the variable relative humidity (RH) and temperature (visualized in Fig. S13 above). Five main steps corresponding to five different conditions occurring subsequently within the related experiment are depicted together with the related average SHG signals and the percentage comparison of these values.

Step	Conditions	Average SHG signal / CPS	SHG signal level in comparison to the starting value of the experiment (Fig. S13),* i.e., for 50% RH and 295 K	SHG signal level in comparison to the lowest value obtained in the experiment (Fig. S13),* i.e., for 97% RH and 295 K
1	50% RH, 295 K	278	100%	102.6%
2	0% RH – vacuum conditions, 295 K	290	105.4%	107.0%
3	0% RH – vacuum conditions, 320 K	290	105.4%	107.0%
4	97% RH, 295 K	271	97.4%	100%
5	0% RH – vacuum conditions, 295 K	289	104.0%	106.6%

* Note that the average experimental error, estimated based on the signal variation upon continuous measurement within the performed experiment (Fig. S13), is related to ca. 10 CPS. This provides a relative error of ca. 3.4–3.7%.

Comment to Fig. S13 and Table S9:

In Fig. S13, the SHG experiment, performed on the pellet sample of **1** by measuring the intensity of output light, is presented. The SHG signal was continuously examined upon the changes in conditions divided into the following steps (summarized in Table S9). First (step 1), the pristine pellet sample was stabilized under 50% RH at 295 K. Then (step 2), the high vacuum was employed to dehydrate the sample. Its usage was continuous over 1940 minutes, keeping the 295 K conditions. As a result, the SHG signal increases slightly reaching ca. 105.4% of the starting value. In the next step (step 3), the vacuum was still turned on while the temperature was increased to 320 K (the heating was performed over 60 min, followed by the stabilization at 320 K for the next 175 min). It did not lead to a further increase of the SHG signal which was on almost the identical level as under the vacuum at 295 K. This means that the dehydration occurs under the vacuum at 295 K and leads to the tiny increase in the SHG signal by ca. 5.4% when compared with the hydrated sample conditioned at 50% RH at 295 K. Next (step 4), the sample was cooled down to 295 K and exposed to the wet atmosphere of 97% RH for ca. 30 min. This results in the decrease of the SHG signal to 97.4% of the starting value (50% RH, 295 K). This indicates that the RH increase rather results in a decrease in the SHG activity. To check if we can formulate some reliable conclusions from this experiment, in the final step (step 5), we turned on the vacuum again. This leads to the increase of the SHG signal to 104.0% of the starting value (50% RH, 295 K) which is close to 105.4% reached in the similar vacuum step 2. Therefore, it can be concluded that the dehydration leads to a noticeable but small increase in the SHG intensity. This can be better depicted when performing the comparison of the related SHG intensity values with the lowest value detected at 97% RH (295 K) (Table S9). Then, the decrease of the RH to 50% results in the SHG intensity increase to 102.6% while the dehydration by the vacuum (0% RH) leads to the further SHG enhancement reaching the level of 106.6–107%. However, it is important to note that the average experimental error, estimated based on the signal variation upon the continuous measurement within the performed experiment (Fig. S13), is related to ca. 10 CPS which provides a relative error of ca. 3.4–3.7%. Therefore, one can postulate that the SHG intensity is, in general, enhanced by the RH decrease (dehydration process) but this enhancement is rather weak, i.e., no more than by 7%, especially taking into account the relative experimental error of ca. 3.4–3.7%.

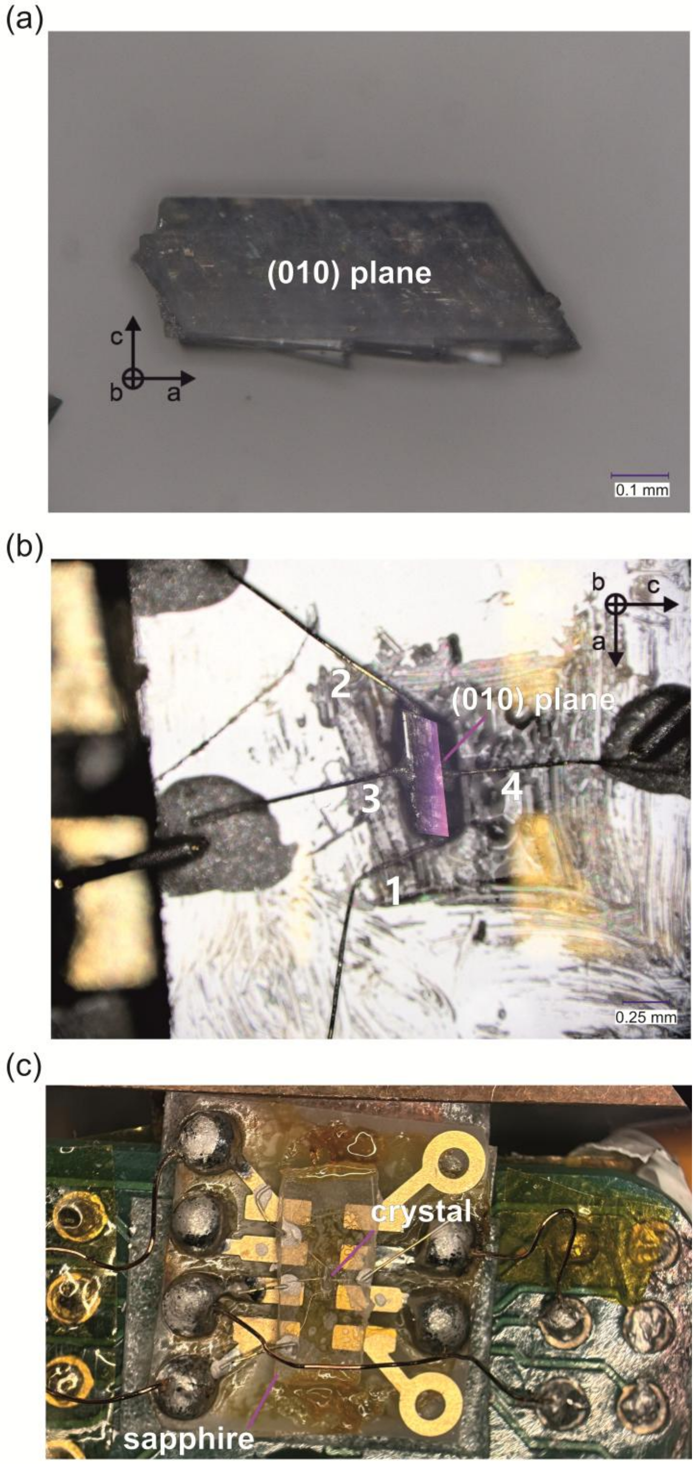

Fig. S14 Photo of the selected indexed single crystal of **1** for the pyroelectric measurement (a), photo of wiring of the sample for the pyroelectric measurement, with the crystal placed on its (010) plane (purple highlight), wires 1-2 attached to the longer direction (*a*-axis), and wires 3-4 attached to the shorter length direction of the crystal (*c*-axis) (b), photo of wiring of the sample for the pyroelectric measurement showing crystal placed on sapphire with broader wiring (c).

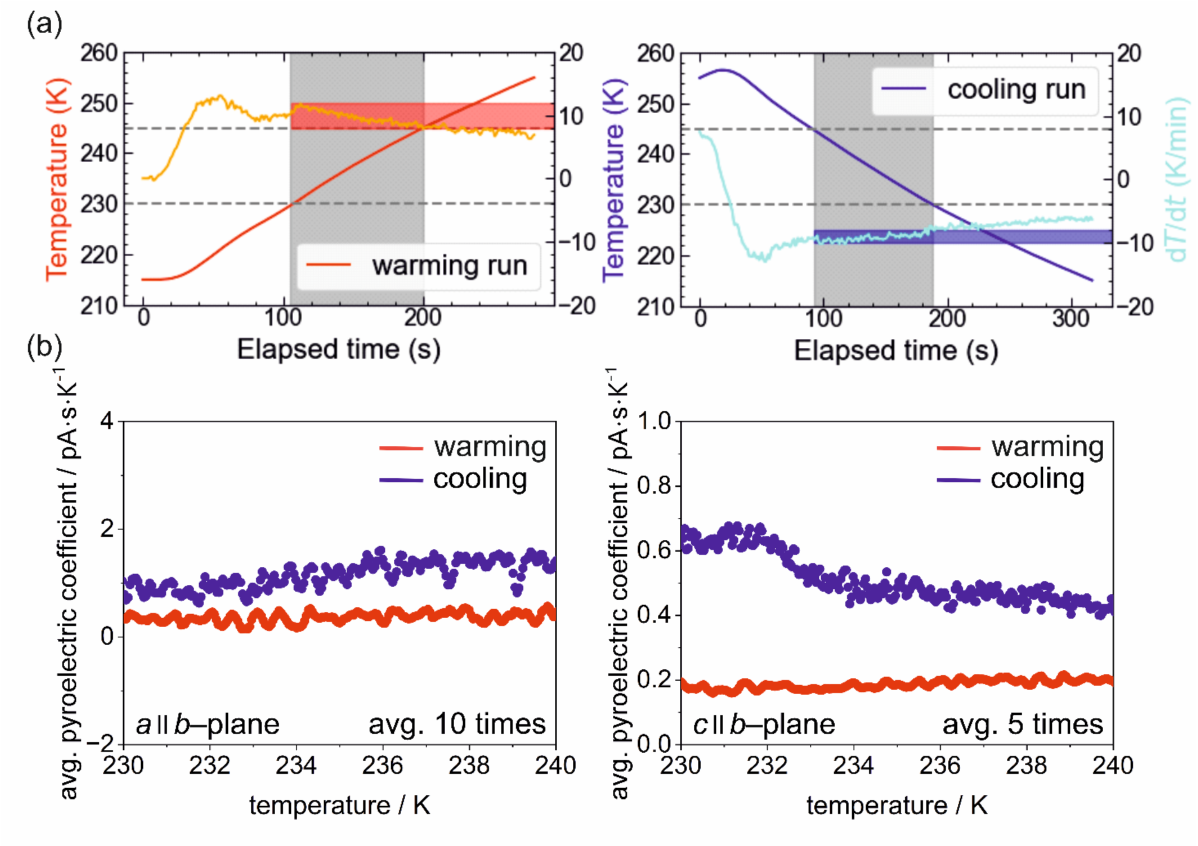

Fig. S15 Detailed curves of pyroelectric measurements performed for **1**, including temperature sweeping rate profiles for warming and cooling runs (a) and average pyroelectric coefficient measured on the *a*-axis along the *b*-plane (b, left), and on the *c*-axis along the *b*-plane (b, right).

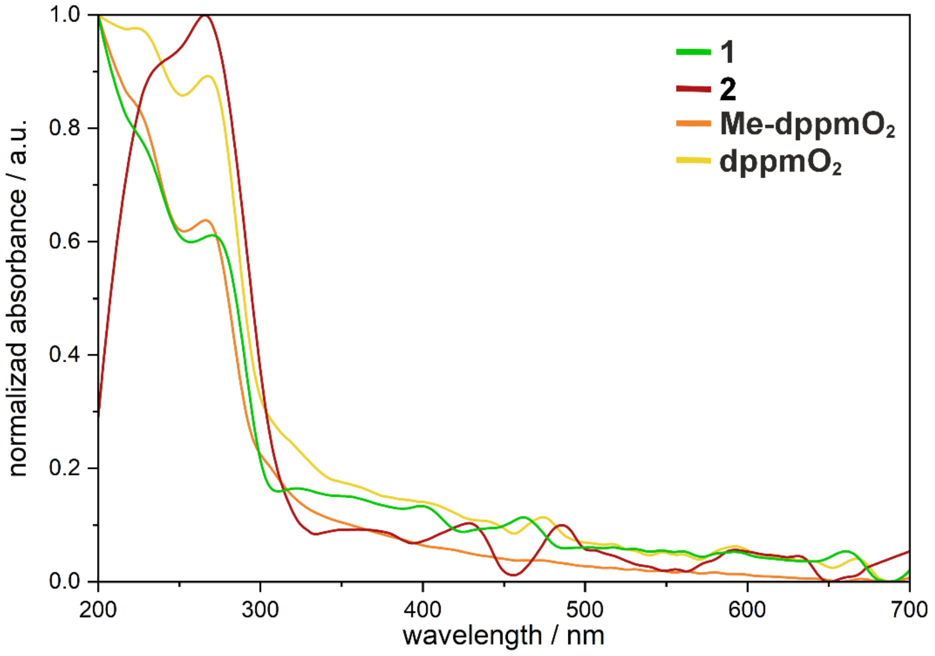

Fig. S16 Normalized room-temperature solid-state UV-vis absorption spectra for **1**, **2**, **Me-dppmO$_2$**, and **dppmO$_2$**, gathered in the 200−700 nm wavelength range. The normalization was done to the maximal absorbance value for each spectrum in the presented wavelength range.

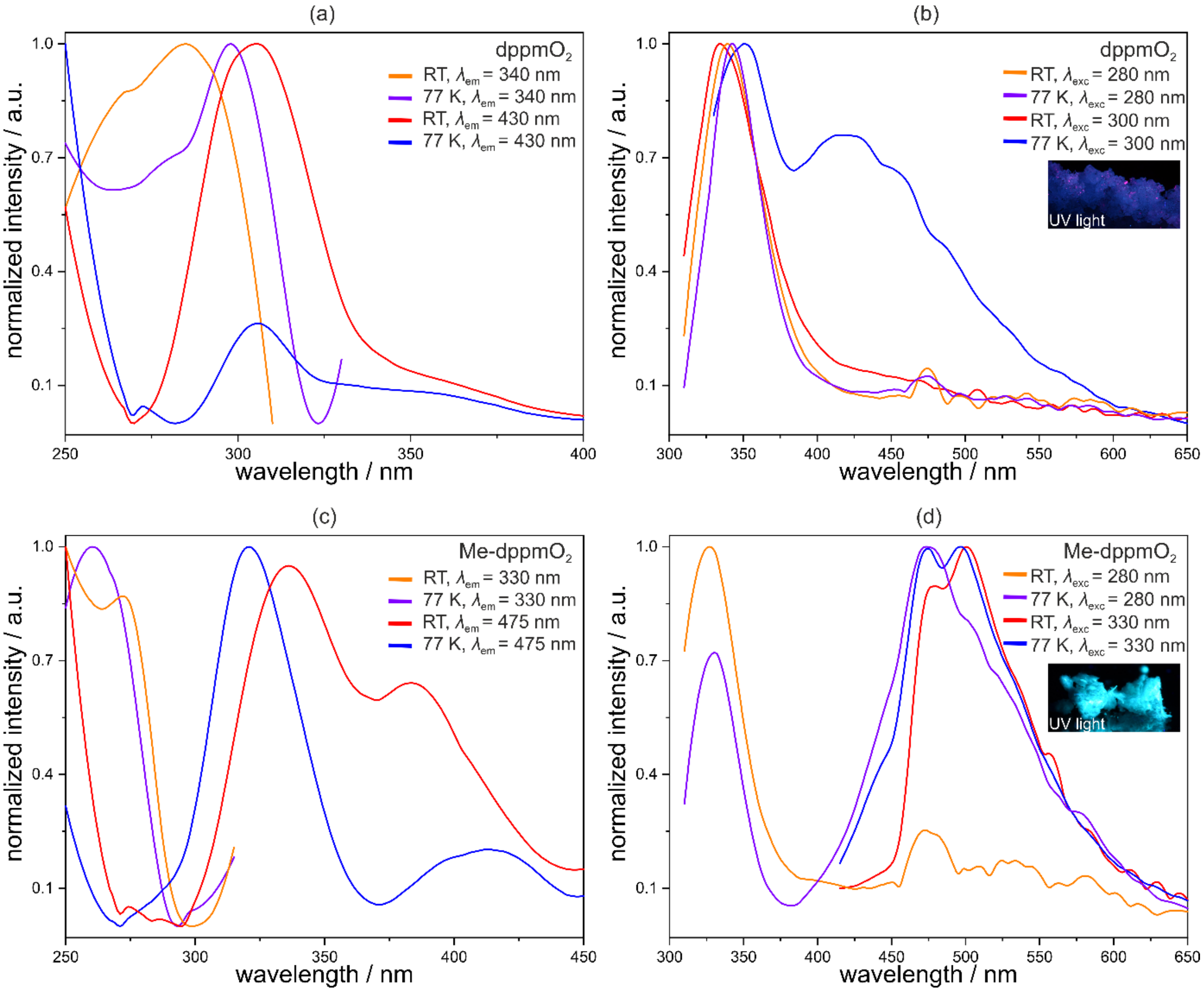

Fig. S17 Solid-state photoluminescence of free organic molecules of **dppmO$_2$** (a, b) and **Me-dppmO$_2$** (c, d), including their excitation (a, c) and emission (b, d) spectra for the indicated wavelength conditions, gathered at room temperature (RT) and 77 K. In the inset of (b) and (d) parts, the photos of the polycrystalline samples of both organic species under UV light irradiation (365 nm) at room temperature were presented.

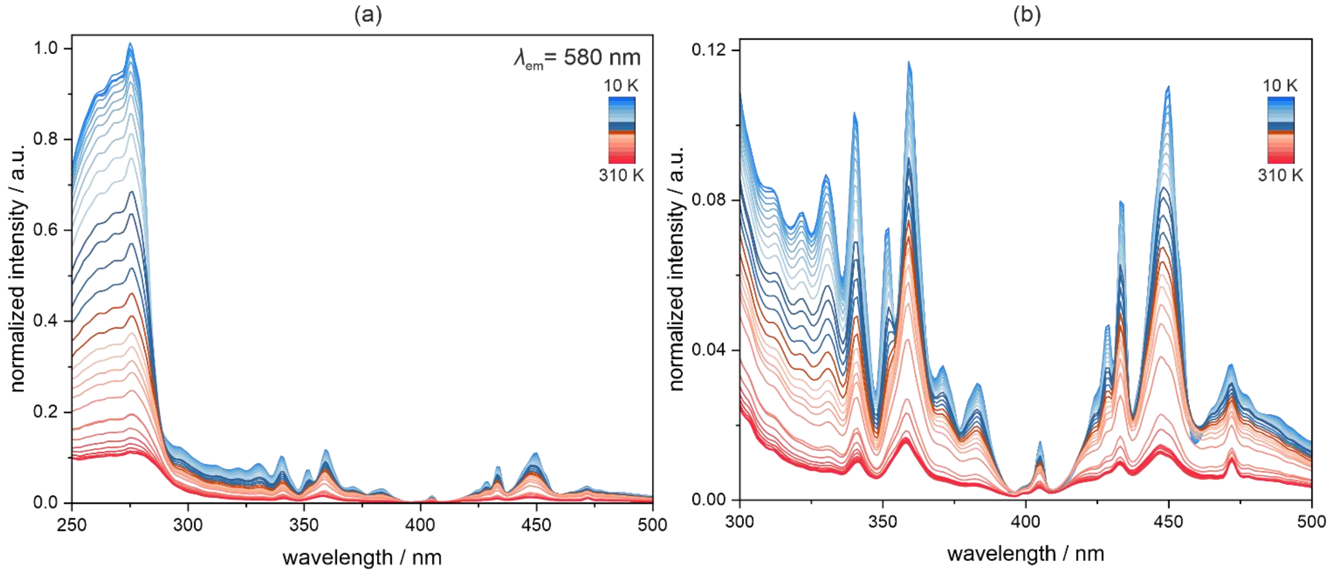

Fig. S18 Temperature-variable solid-state excitation spectra of **1** for the indicated emission of λ_{em} = 580 nm, gathered in the 10–310 K temperature range and 250–500 nm wavelength range (a) and the enlargement of the lower-energy part of the 300–500 nm range corresponding to d-d electronic transitions (b).

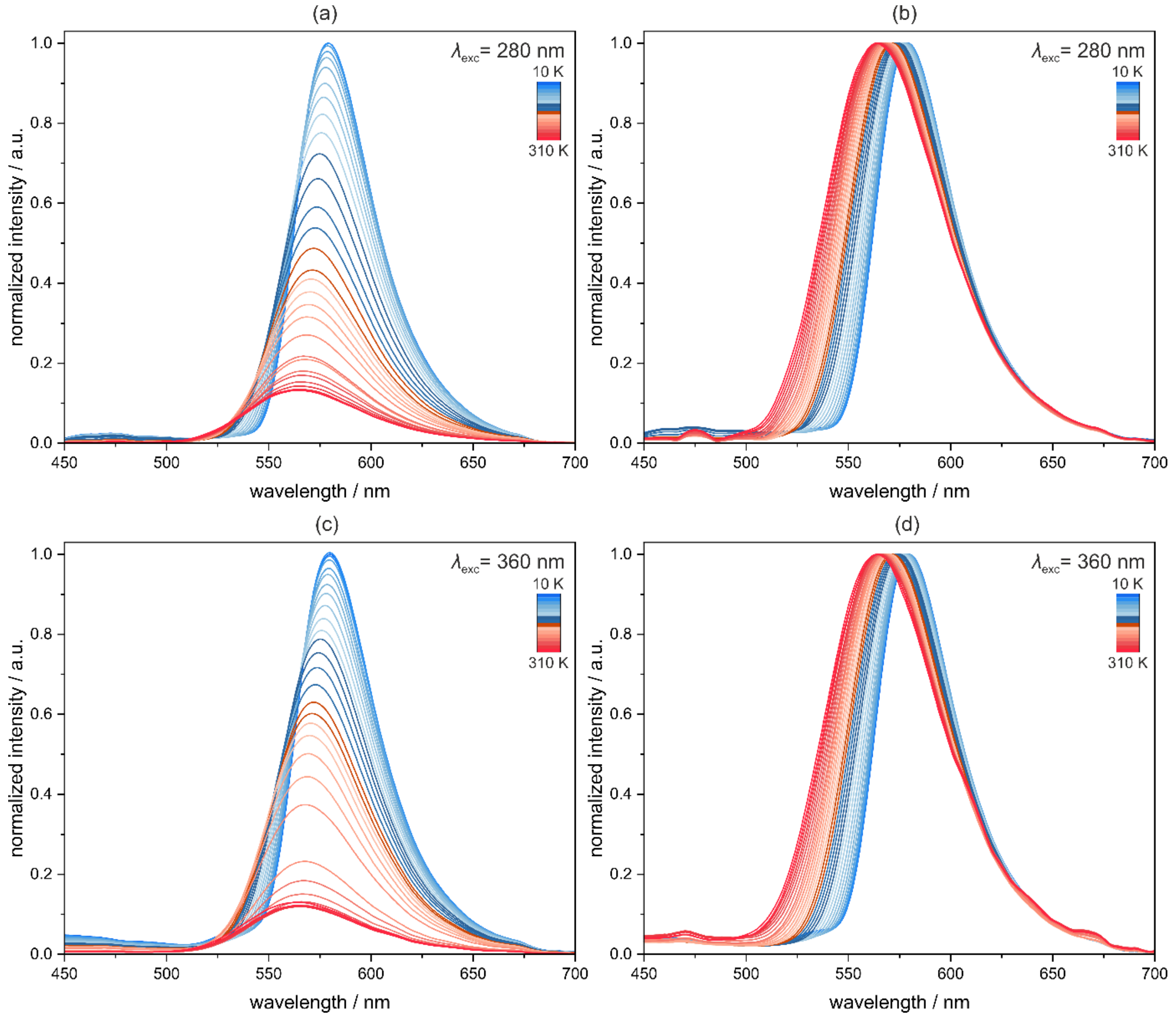

Fig. S19 Temperature-variable solid-state emission spectra of **1** gathered in the 10–310 K temperature range, under the excitation by the wavelengths of λ_{exc} = 280 nm (a) and λ_{exc} = 360 nm (c), shown by the intensity normalized to the maximum at 10 K, and the respective series of spectra with each spectrum normalized to its maximum (b and d, for λ_{exc} = 280 nm and λ_{exc} = 360 nm, respectively).

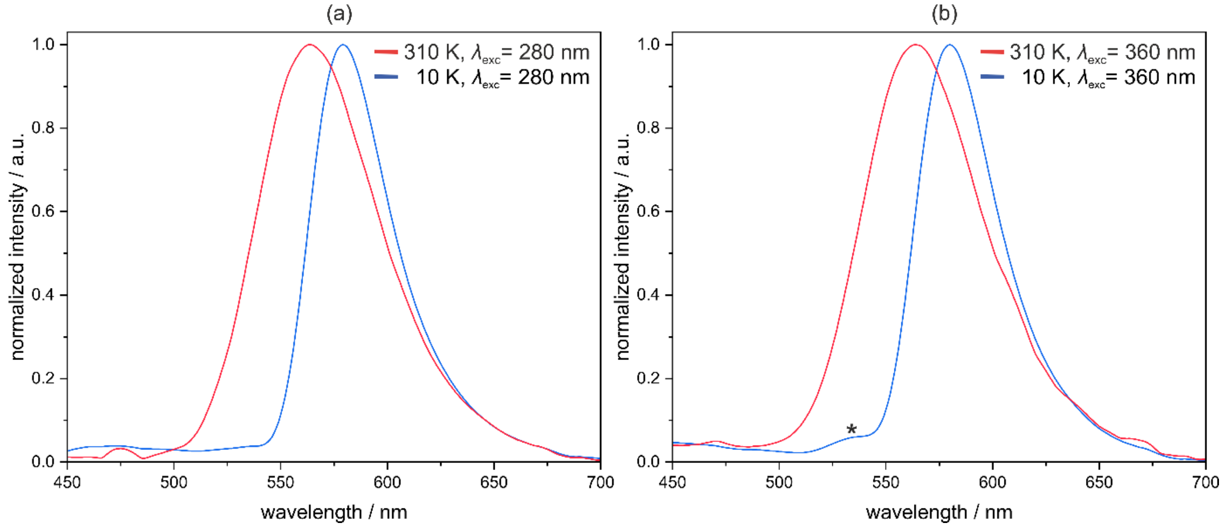

Fig. S20 Comparison for the solid-state emission spectra of **1** at 310 K and 10K under the excitation of λ_{exc} = 280 nm (a) and λ_{exc} = 360 nm (b). All spectra were normalized to their maximum. In (b), the weak emission peak assignable to tetrahedral Mn2 centers was marked with a star.

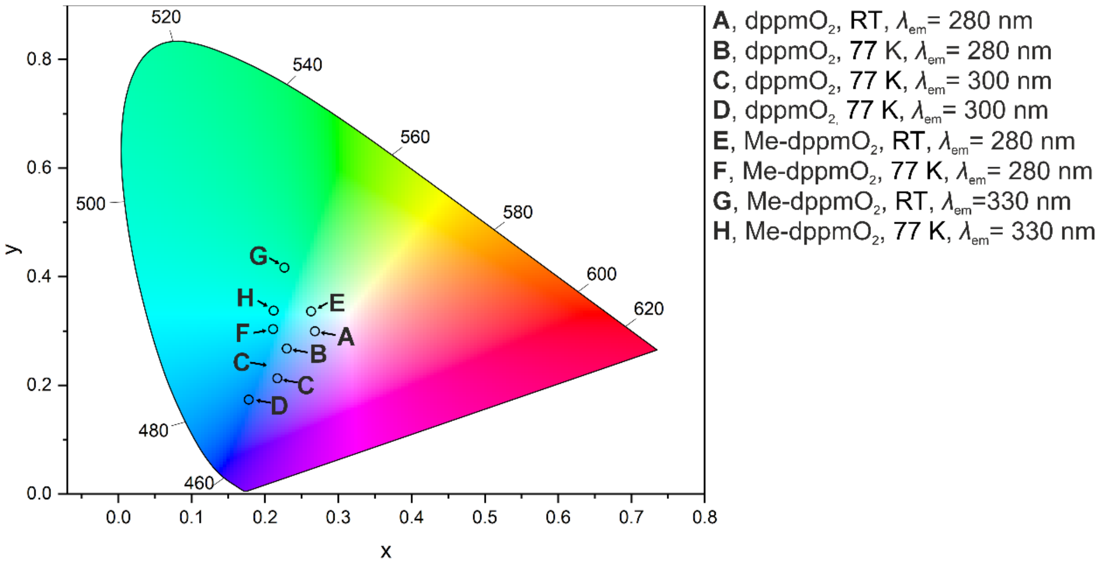

Fig. S21 Emission colors at room temperature (RT) and 77 K of the powder samples of **dppmO$_2$** (A–D) and **Me-dppmO$_2$** (E–H), presented on the CIE 1931 chromaticity diagram. The excitation wavelengths and temperature conditions are indicated. The respective emission spectra are shown in Fig. S17. The related CIE 1931 chromaticity parameters are gathered in Table S10.

Table S10 The xy CiE 1931 chromaticity parameters of solid-state emission patterns of free **dppmO$_2$** and **Me-dppmO$_2$** molecules under the indicated excitation and temperature conditions (RT = room temperature). The respective emission spectra are shown in Fig. S17 while the visualization of emission colors on the chromaticity diagram is presented in Fig. S21 above.

Sample	Temperature / K	λ_{exc} / nm	CIE 1931 chromaticity parameters	
			x	y
**dppmO$_2$**	RT	280	0.268	0.300
	77 K	280	0.230	0.268
	RT	300	0.217	0.213
	77 K	300	0.178	0.174
**Me-dppmO$_2$**	RT	280	0.263	0.336
	77 K	280	0.211	0.304
	RT	330	0.226	0.417
	77 K	330	0.212	0.338

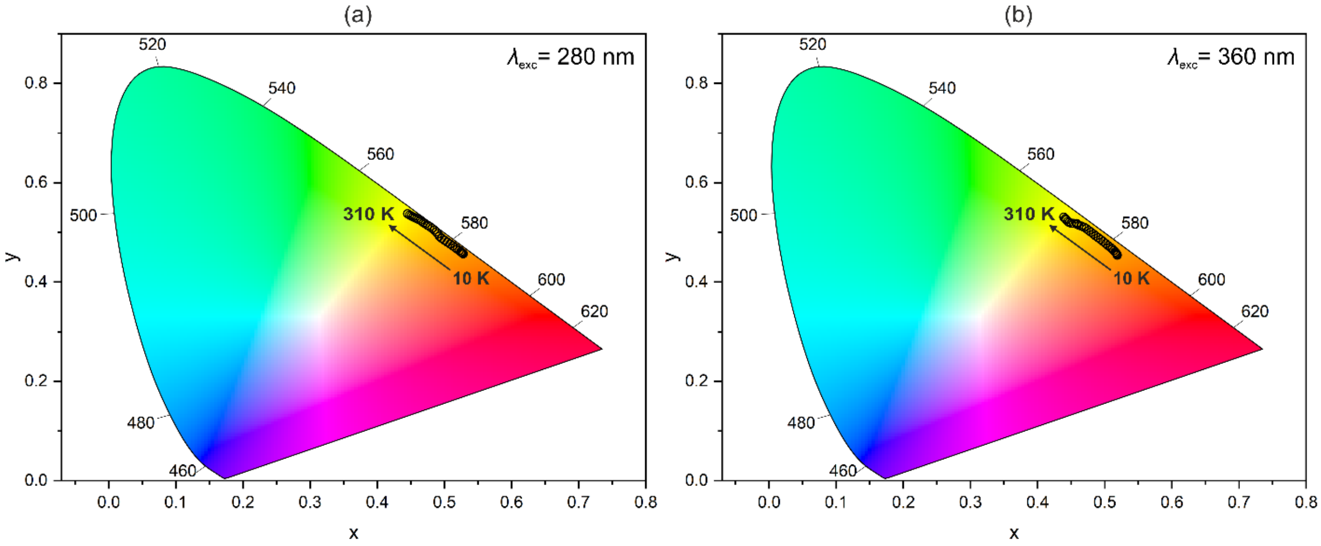

Fig. S22 Emission colors for the powder sample of **1** under the excitation of λ_{exc} = 280 nm (a) and λ_{exc} = 360 nm (b), for the 10−310 K temperature range, presented on the CIE 1931 chromaticity diagrams. The respective emission spectra are shown in Fig. S19. The related CIE 1931 chromaticity parameters are gathered in Table S11.

Table S11 Chromaticity parameters of the CIE 1931 scale of the solid-state emission of **1** detected at the indicated temperatures (for the respective emission spectra, see Fig. 19, while for the emission colors presented on the CIE 1931 chromaticity diagrams, see Fig. S22).

T / K	λ_{exc} = 280 nm		λ_{exc} = 360 nm	
	CIE 1931 chromaticity parameters			
	x	y	x	y
10	0.528	0.457	0.518	0.454
20	0.528	0.457	0.518	0.455
30	0.527	0.458	0.518	0.456
40	0.526	0.459	0.518	0.457
50	0.524	0.461	0.517	0.459
60	0.522	0.464	0.515	0.461
70	0.519	0.467	0.513	0.464
80	0.516	0.470	0.511	0.467
90	0.512	0.473	0.508	0.470
100	0.508	0.477	0.505	0.474
110	0.504	0.481	0.501	0.478
120	0.500	0.484	0.498	0.482
130	0.496	0.488	0.494	0.486
140	0.492	0.492	0.490	0.489
150	0.490	0.497	0.486	0.493
160	0.488	0.501	0.483	0.497
170	0.485	0.505	0.480	0.501
180	0.482	0.508	0.476	0.504
190	0.478	0.512	0.472	0.508
200	0.475	0.515	0.469	0.511
210	0.472	0.517	0.465	0.513
220	0.469	0.520	0.461	0.515
230	0.467	0.522	0.459	0.516
240	0.464	0.525	0.458	0.518
250	0.461	0.526	0.453	0.518
260	0.459	0.528	0.450	0.519
270	0.456	0.530	0.447	0.520
280	0.453	0.532	0.445	0.522
290	0.451	0.533	0.442	0.525
300	0.448	0.535	0.441	0.528
310	0.445	0.538	0.439	0.531

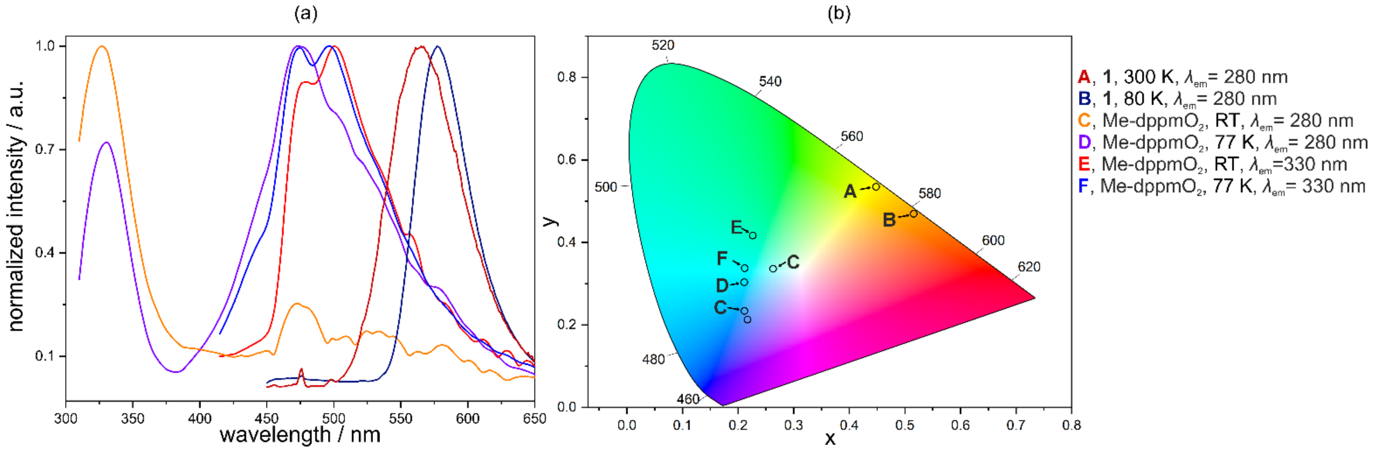

Fig. S23 Comparison of emission spectra (a) and resulting emission colors presented on the CIE 1931 chromaticity diagram (b) for the powder samples of **1** (A and B) and free **Me-dppmO₂** molecules (C–F). The excitation wavelength and temperature conditions are indicated. The related CIE 1931 chromaticity parameters are gathered in Tables S10 and S11.

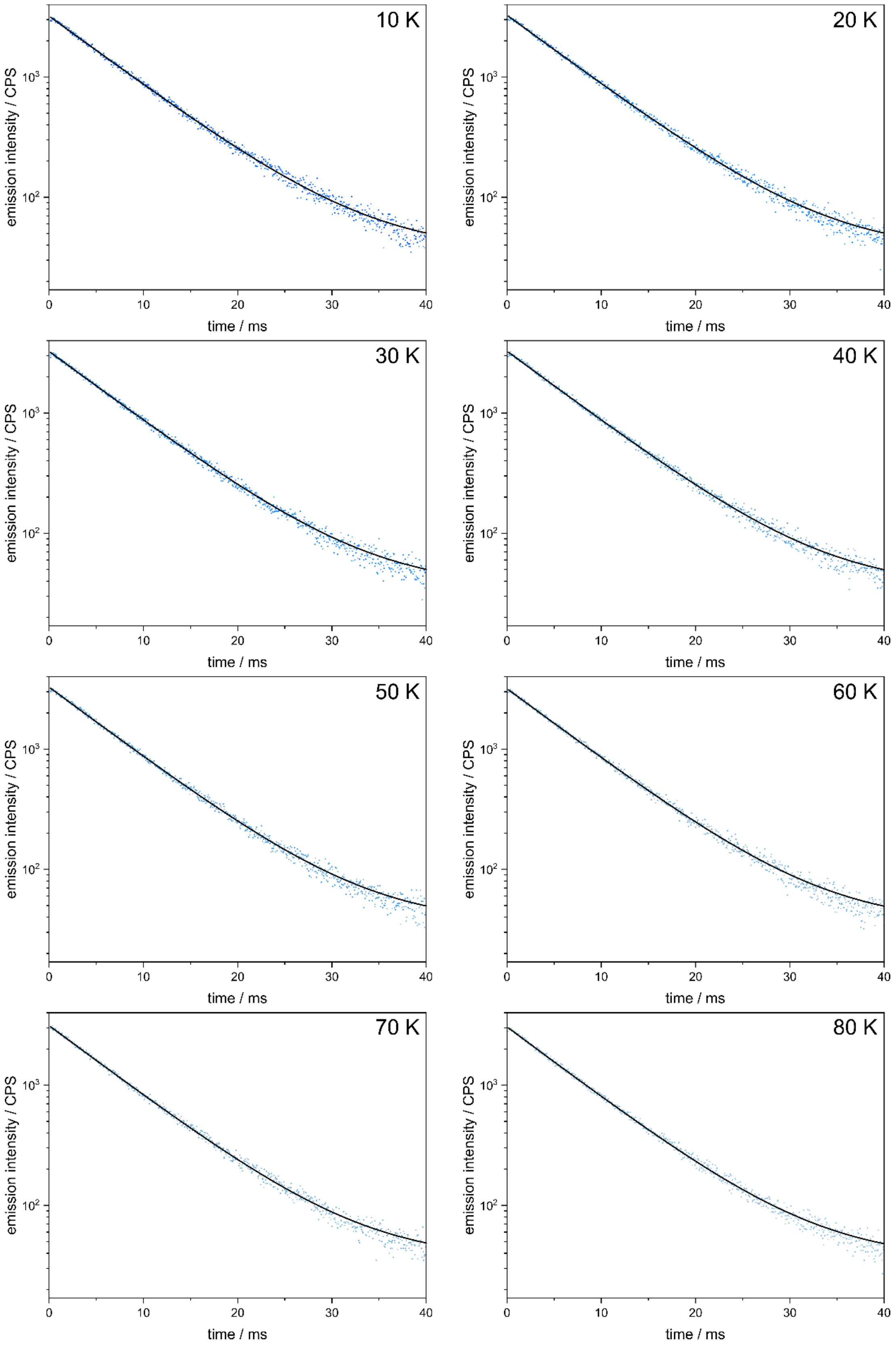

Fig. S24 Temperature-variable emission decay profiles for **1** under the excitation of λ_{exc} = 280 nm for the emission of λ_{em} = 580 nm, gathered in the 10−80 K temperature range. The mono-exponential fitting was applied for each temperature (solid line). The best-fit parameters are gathered in Table S12.

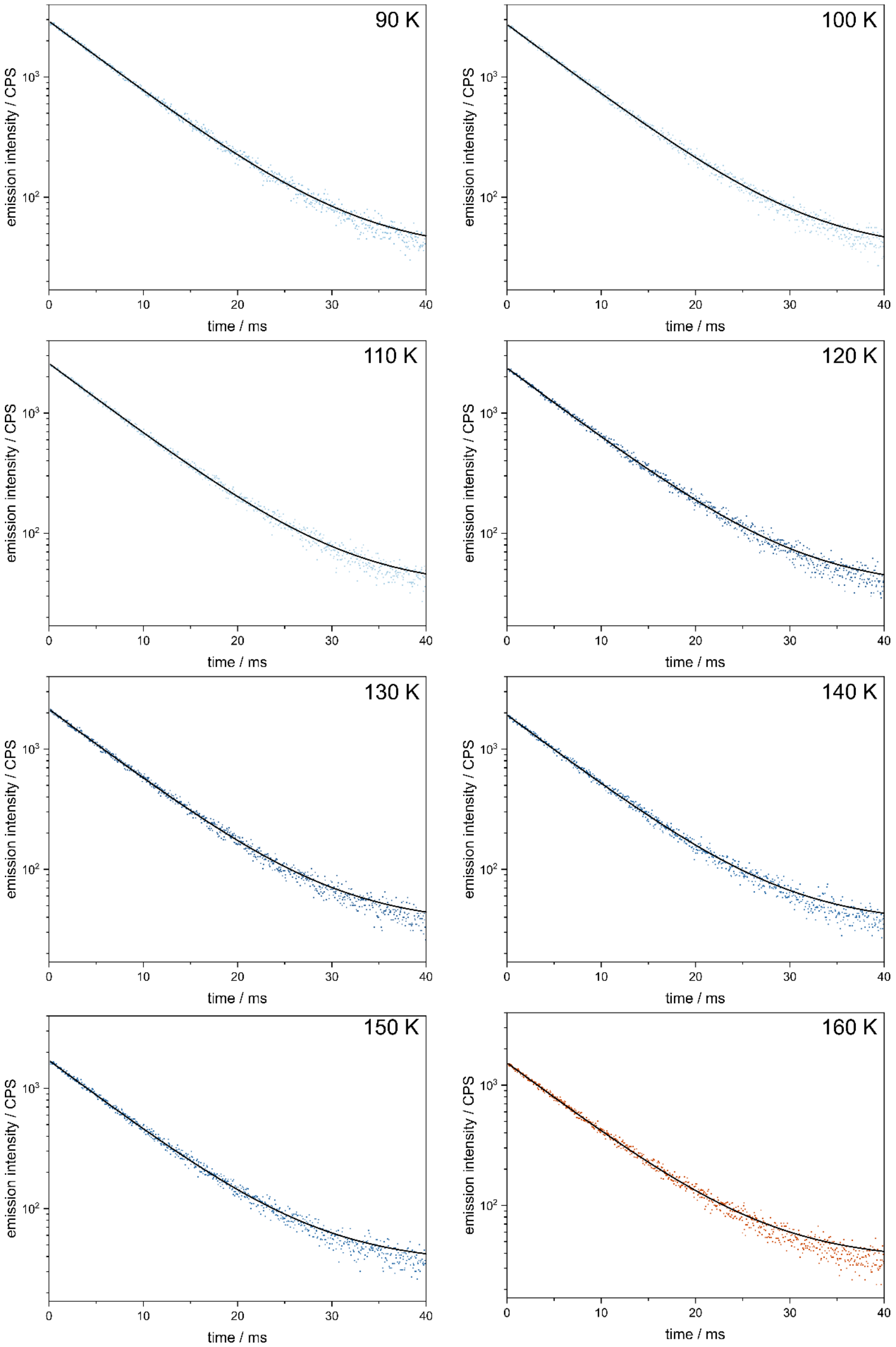

Fig. S25 Temperature-variable emission decay profiles for **1** under the excitation of λ_{exc} = 280 nm for the emission of λ_{em} = 580 nm, gathered in the 90−160 K temperature range. The mono-exponential fitting was applied for each temperature (solid line). The best-fit parameters are gathered in Table S12.

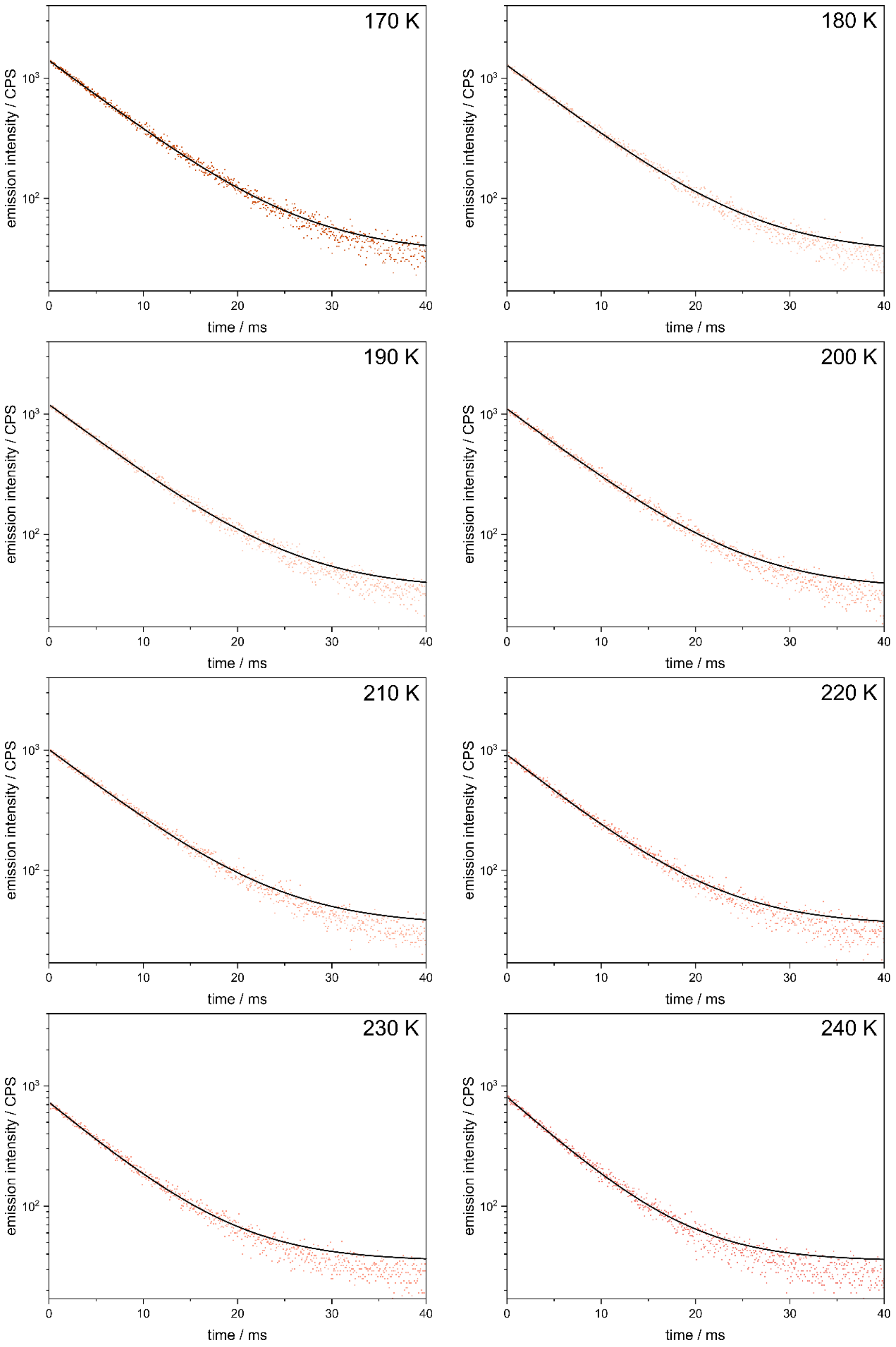

Fig. S26 Temperature-variable emission decay profiles for **1** under the excitation of λ_{exc} = 280 nm for the emission of λ_{em} = 580 nm, gathered in the 170–240 K temperature range. The mono-exponential fitting was applied for each temperature (solid line). The best-fit parameters are gathered in Table S12.

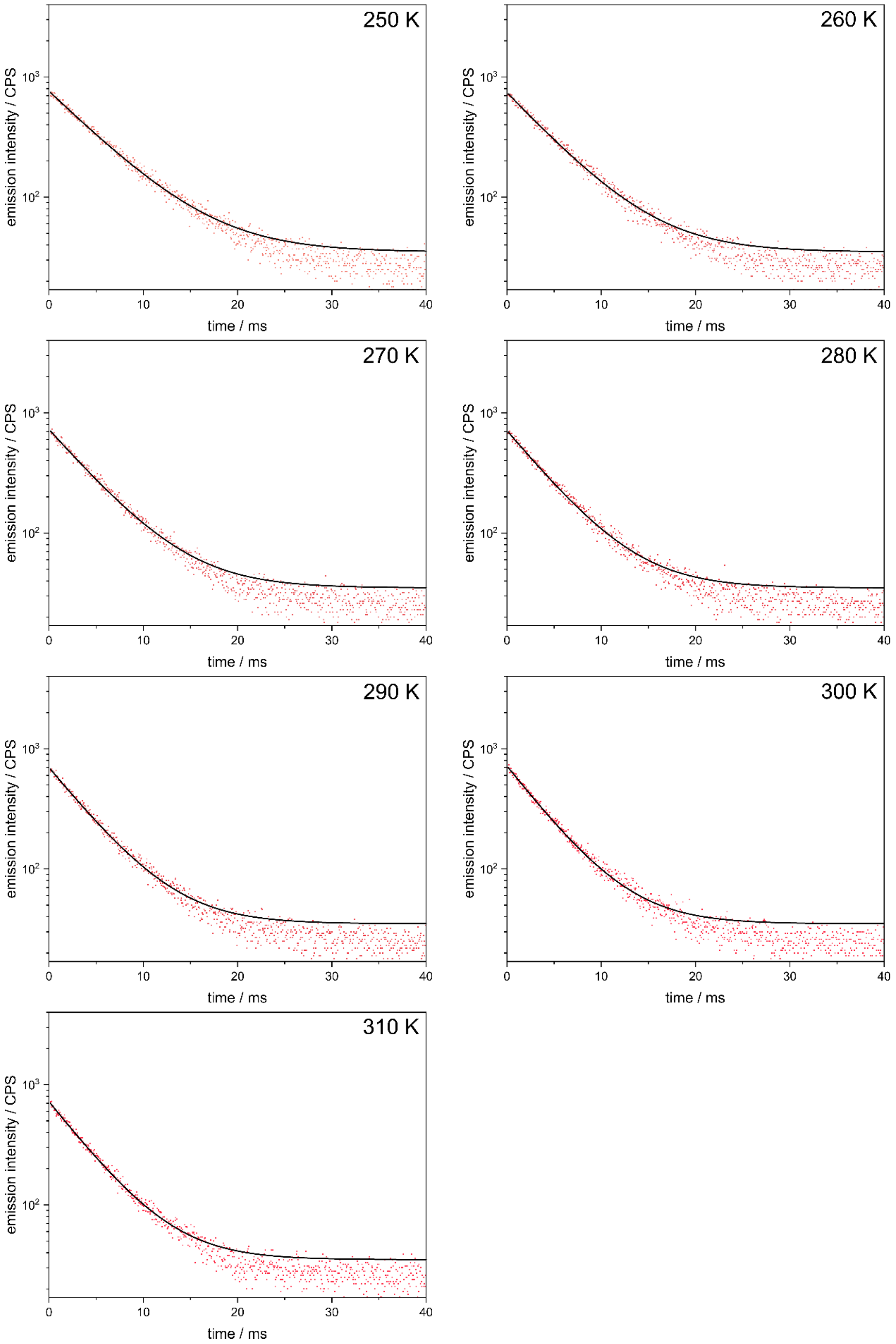

Fig. S27 Temperature-variable emission decay profiles for **1** under the excitation of λ_{exc} = 280 nm for the emission of λ_{em} = 580 nm, gathered in the 250–310 K temperature range. The mono-exponential fitting was applied for each temperature (solid line). The best-fit parameters are gathered in Table S12.

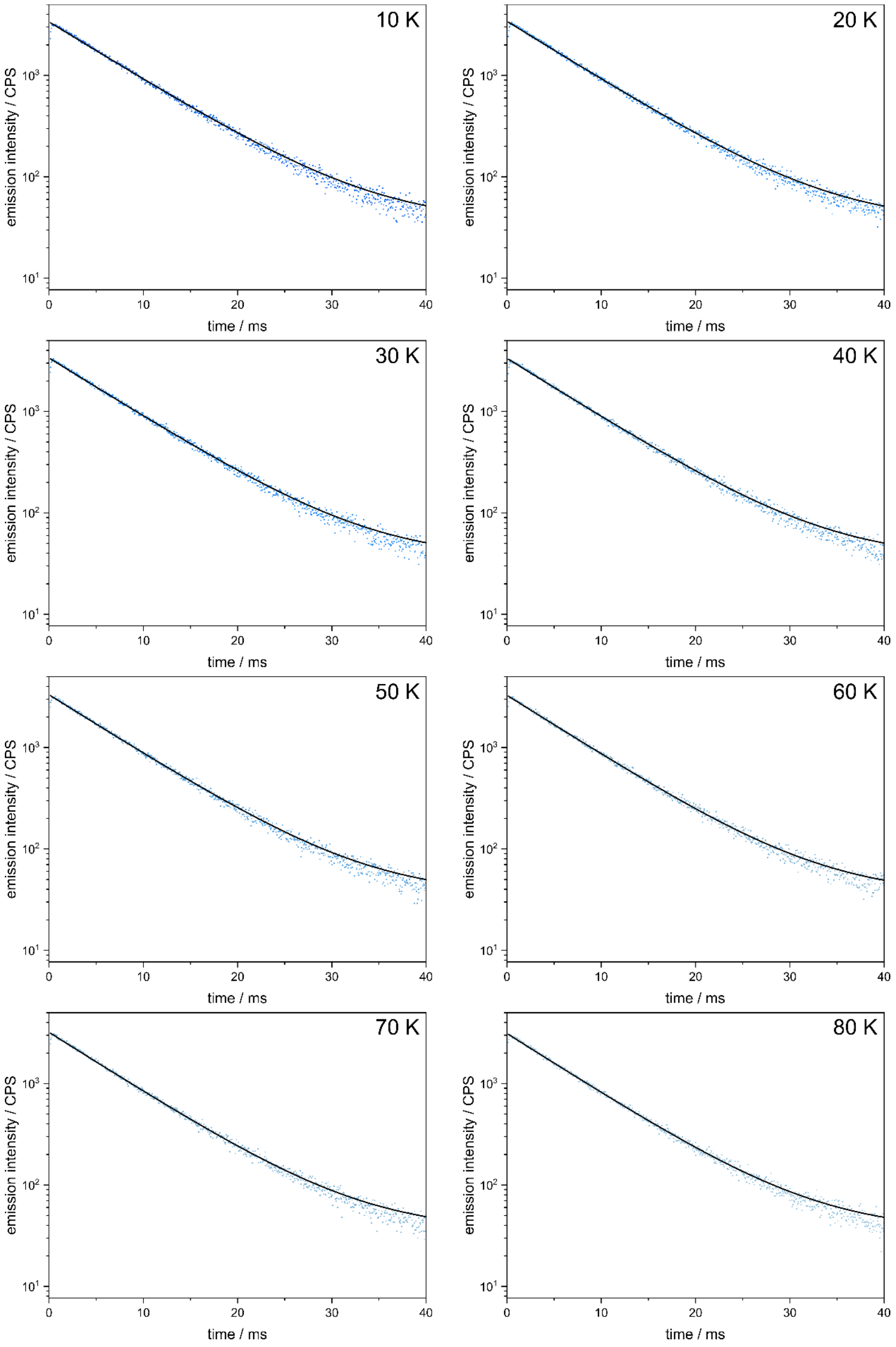

Fig. S28 Temperature-variable emission decay profiles for **1** under the excitation of λ_{exc} = 360 nm for the emission of λ_{em} = 580 nm, gathered in the 10−80 K temperature range. The mono-exponential fitting was applied for each temperature (solid line). The best-fit parameters are gathered in Table S12.

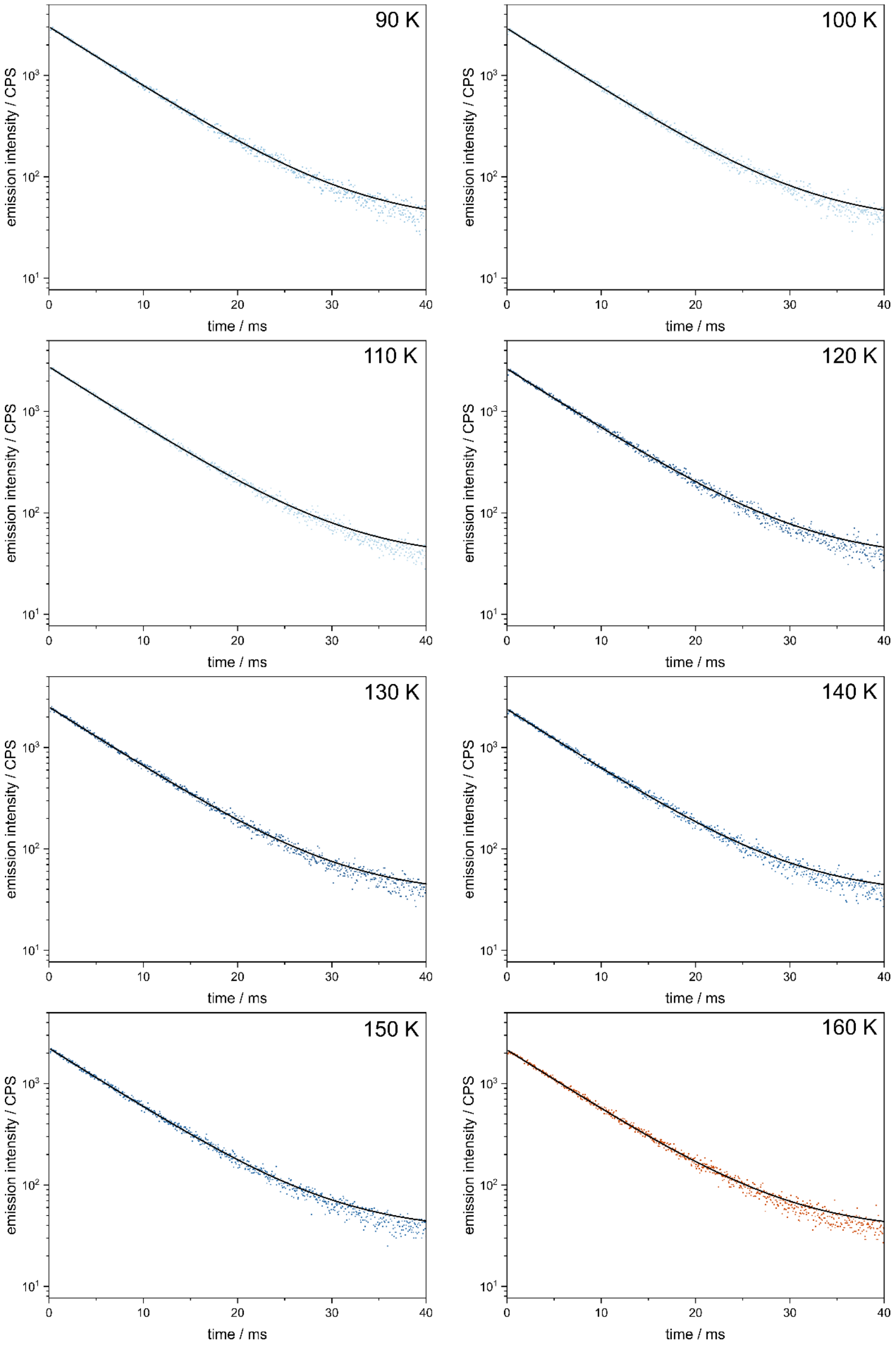

Fig. S29 Temperature-variable emission decay profiles for **1** under the excitation of λ_{exc} = 360 nm for the emission of λ_{em} = 580 nm, gathered in the 90–160 K temperature range. The mono-exponential fitting was applied for each temperature (solid line). The best-fit parameters are gathered in Table S12.

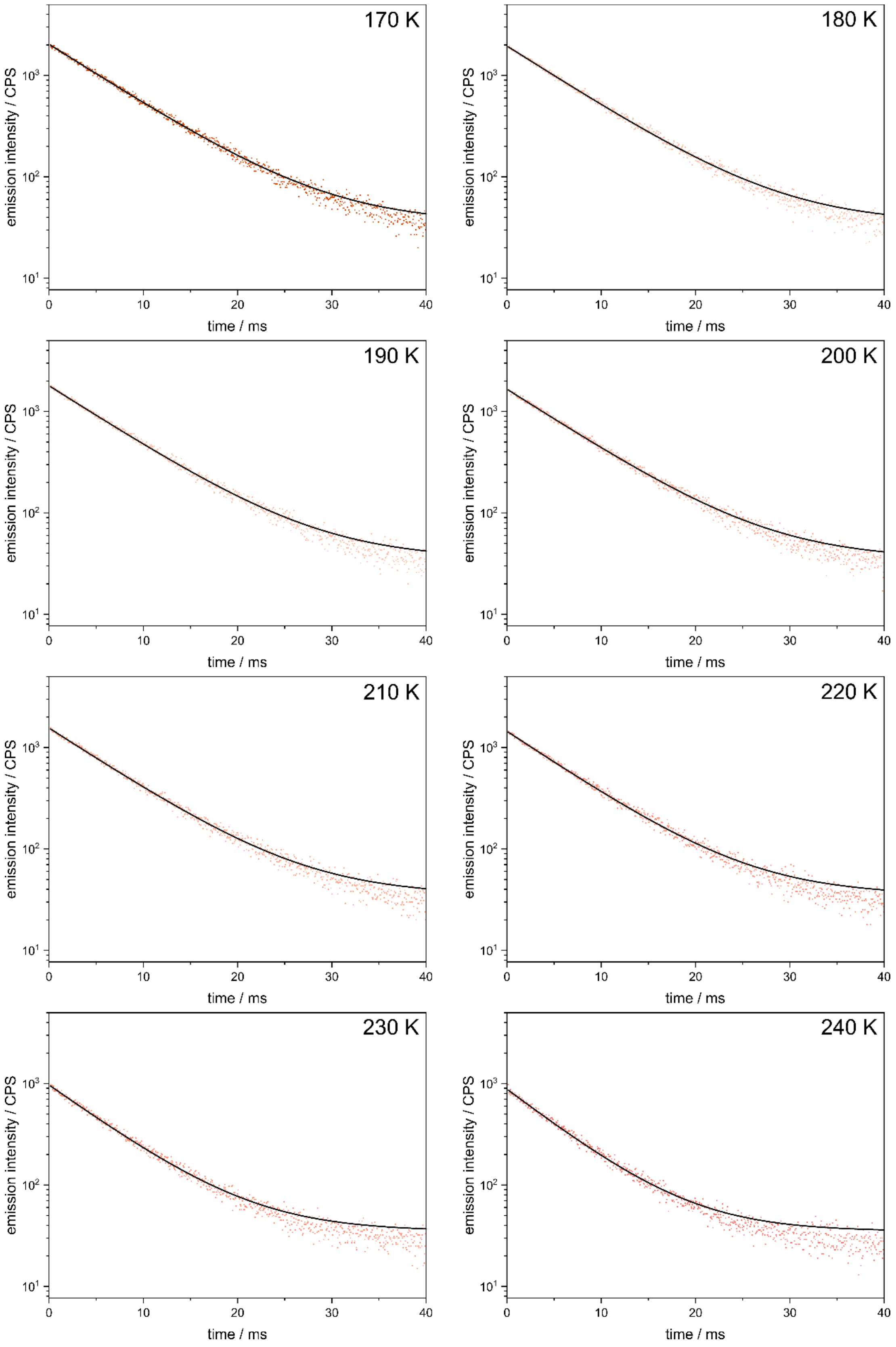

Fig. S30 Temperature-variable emission decay profiles for **1** under the excitation of λ_{exc} = 360 nm for the emission of λ_{em} = 580 nm, gathered in the 170−240 K temperature range. The mono-exponential fitting was applied for each temperature (solid line). The best-fit parameters are gathered in Table S12.

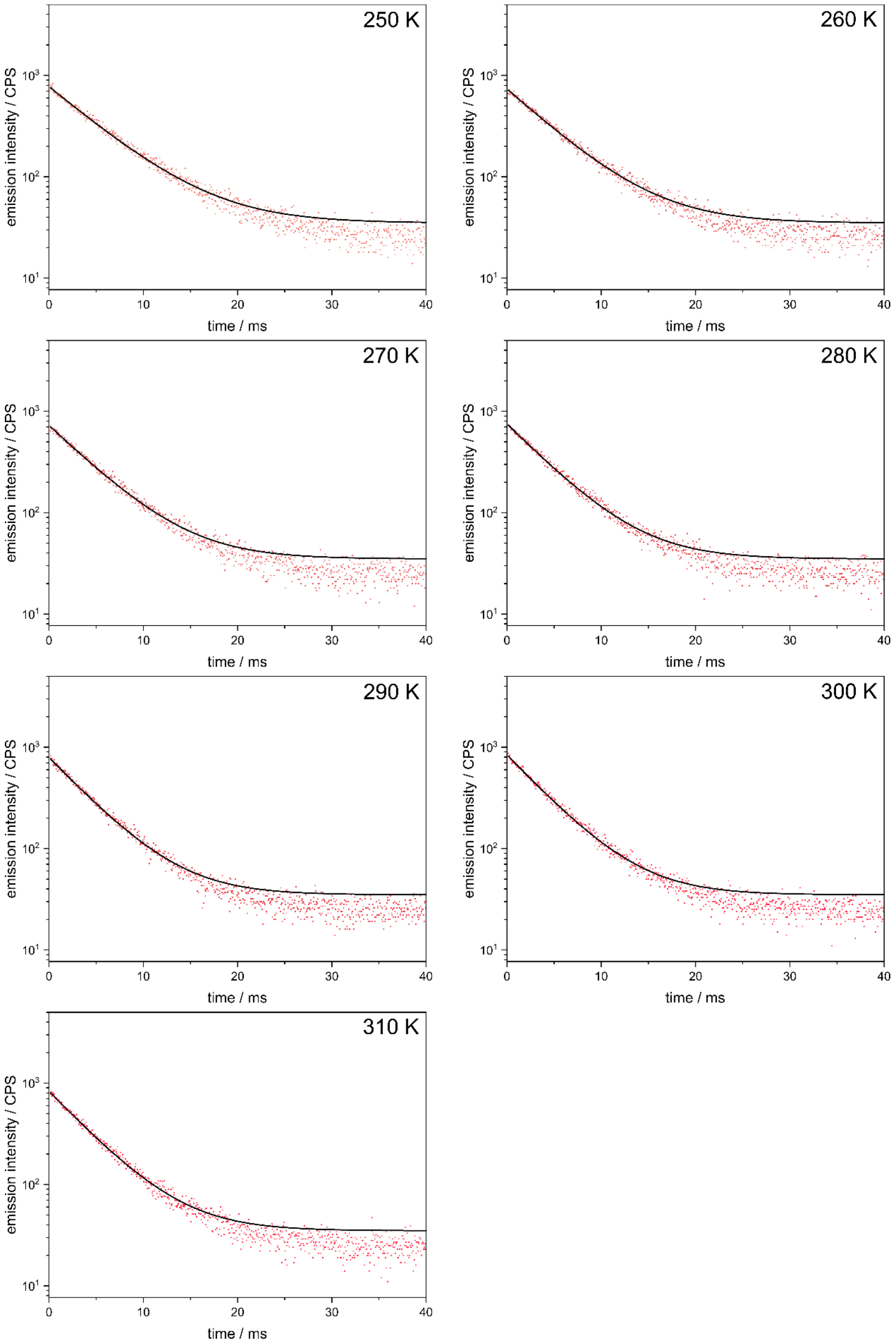

Fig. S31 Temperature-variable emission decay profiles for **1** under the excitation of λ_{exc} = 360 nm for the emission of λ_{em} = 580 nm, gathered in the 250−310 K temperature range. The mono-exponential fitting was applied for each temperature (solid line). The best-fit parameters are gathered in Table S12.

Table S12 Best-fit parameters to the mono-exponential decay function for the temperature-variable emission decay profiles of **1** (λ_{exc} = 280 nm and λ_{em} = 580 nm, as well as λ_{exc} = 360 nm and λ_{em} = 580 nm) which were gathered in the 10–310 K temperature (Fig. S24–S31).

T / K	λ_{exc} = 280 nm		λ_{exc} = 360 nm	
	τ_1 / ms	χ^2	τ_1 / ms	χ^2
10	7.51(1)	0.9988	7.56(2)	0.9966
20	7.48(1)	0.9989	7.51(2)	0.9970
30	7.46(1)	0.9989	7.47(2)	0.9973
40	7.42(1)	0.9989	7.45(2)	0.9967
50	7.41(1)	0.9989	7.41(1)	0.9977
60	7.43(1)	0.9990	7.37(1)	0.9980
70	7.38(1)	0.9990	7.34(1)	0.9978
80	7.35(1)	0.9989	7.32(1)	0.9981
90	7.37(2)	0.9988	7.32(1)	0.9982
100	7.36(2)	0.9967	7.29(1)	0.9985
110	7.34(1)	0.9987	7.30(2)	0.9984
120	7.36(2)	0.9986	7.31(2)	0.9985
130	7.35(2)	0.9984	7.29(2)	0.9985
140	7.34(2)	0.9980	7.27(1)	0.9982
150	7.32(2)	0.9976	7.31(3)	0.9981
160	7.33(2)	0.9974	7.27(1)	0.9982
170	7.27(2)	0.9968	7.21(2)	0.9980
180	7.24(2)	0.9960	7.26(2)	0.9980
190	7.31(2)	0.9960	7.25(2)	0.9976
200	7.26(2)	0.9947	7.21(2)	0.9975
210	7.20(2)	0.9942	7.13(2)	0.9972
220	6.91(2)	0.9929	6.94(2)	0.9969
230	6.54(3)	0.9898	6.46(2)	0.9934
240	6.12(3)	0.9904	6.04(2)	0.9913
250	5.59(3)	0.9886	5.53(3)	0.9883
260	5.12(3)	0.9871	5.11(3)	0.9876
270	4.80(3)	0.9855	4.78(3)	0.9864
280	4.51(3)	0.9848	4.55(2)	0.9873
290	4.42(2)	0.9842	4.39(2)	0.9874
300	4.24(2)	0.9844	4.35(2)	0.9888
310	4.27(3)	0.9847	4.39(2)	0.9886

Table S13 Absolute quantum yield of **1** measured at room temperature in the solid state under the indicated excitation conditions.

Compound	λ_{exc} / nm	Quantum yield (Φ)[S7]
**1**	250	33.33%

Comment on theoretical calculations of optical transitions in Mn(II) complexes in 1 (Fig. S32–S35 and S66, as well as Tables S14–S22 and S35–S38)

For the computational part of our work, we employed the ORCA 5.0.4 quantum chemistry package.[S12,S13] We considered two complexes, Mn1 centers of the formula of $[Mn^{II}(Me\text{-}dppmO_2)_3]^{2+}$ and Mn2 centers of the formula of $[Mn^{II}Cl_4]^{2-}$ (Fig. 1), as model clusters for theoretical investigation whose geometries were taken directly from the crystal structure of **1** determined by the SC-XRD experiment. We omitted solvent molecules and conducted calculations in the gas phase without solvation models. We used scalar relativistic ZORA Hamiltonian together with a minimally augmented ma-ZORA-def2-TVZP basis set for Mn(II) centers and, due to the large size of the Mn1 complex (163 atoms), ma-ZORA-def2-SVP for other atoms.[S14] We conducted SA-CASSCF[S15] + SC-NEVPT2[S16] using RIJCOSX approximation with AutoAux[S17] for auxiliary basis set generation to facilitate the treatment of Mn1 complexes. The active space was composed of five electrons and five 3d-orbitals of Mn CAS(5 in 5), see Fig. S32–S34, and we optimized 1 sextet, 24 quartet, and 75 doublet roots originating from different possible configurations within the d-shell (in some indicated cases, doublet states were omitted). In the next step, the resulting electronic states were mixed with spin-orbit coupling (SOC) adopting quasi-degenerate perturbation theory in the ORCA's SOC submodule. We report the state's energies for various levels of theory and limited or broadened sets of mixed states in Tables S14–S21. Thanks to the very small size of Mn2 complexes (5 atoms), we were able to optimize the geometry of the first quartet obtained within the SA-CASSCF+SC-NEVPT2 level of theory using numerical gradients (Fig. S34). Transition energies from the ground sextet to the ground quartet state within the optimized geometry of the excited state were calculated to represent the energy of photoluminescence (i.e., emissive transitions, Table S22). Analogous treatment is not feasible for Mn1 centers with the available computational resources. We can only guess that the energy would follow the indicated trends towards lower energies when going from absorption to emission properties as assumed for the Mn(II) centers in the octahedral crystal field. To facilitate the direct comparison of computational results with the experimental excitation spectra (Fig. 6 and S35), we applied the constant energy shift of 8000 cm^{-1} for the energy of states obtained from the SA-CASSCF and SA-CASSCF-SOC theory levels, and 5000 cm^{-1} for the energy of states obtained from the SA-CASSCF-NEVPT2 and SA-CASSCF-NEVPT2-SOC theory levels, effectively setting the 0-line, following obtained transition energies for Mn2 sites assumed as $^6A_1(S)\rightarrow{}^4E(D)$ to literature ca. 360 nm (see refs. 67, 82, 83 in the main manuscript).

As the above-presented computations were performed using the structural models directly taken from the results of SC-XRD analyses which included the refinement of hydrogen atoms in the Mn1 sites using a riding model (see Experimental section for details), for the sake of comparison, we also optimized the position of the hydrogen atoms for the Mn1 complexes (Mn2 sites do not contain the hydrogen atoms) at ZORA-B3LYP level of theory using "Tight" convergence criteria for wavefunction and gradients. Such a model of Mn1 complexes with the optimized H-atoms, named Mn1^{opt}, was found to contain noticeably longer C–H bond lengths as compared to the Mn1 complexes found in the SC-XRD analysis (Fig. S66, Table S34). However, we checked that it only subtly affects the energy-splitting scheme. The related energy differences for the CASSCF- and CASSCF-SOC-type calculations are up to ca. 10 cm^{-1} (below 0.04%, Tables S35 and S36), while the analogous calculations with the NEVPT2 correction reveal the related energy differences up to ca. 25 cm^{-1} (below 0.11%, Tables S37 and S38).

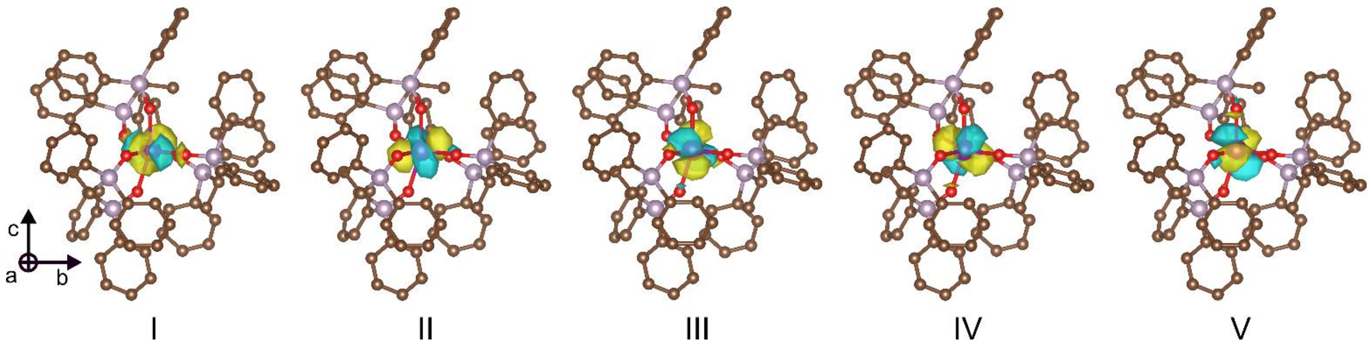

Fig. S32 Visualization of active space, composed of five 3d orbitals (I–V), applied for the computational procedures for Mn1 centers (ground state geometry). The orbitals were plotted with an isosurface level of 0.022. Hydrogen atoms are omitted for clarity. Note that the largest contribution (>30%) to the lowest energy electronic transition in **1** (found for Mn1 by the CASSCF+NEVPT2 with sextet and quartet states considered, Table S18), presented in Fig. 6b, is here represented by the I-to-IV transition. The above-presented active space was used for calculations employing all considered theory levels for Mn1 centers (Tables S14–S21).

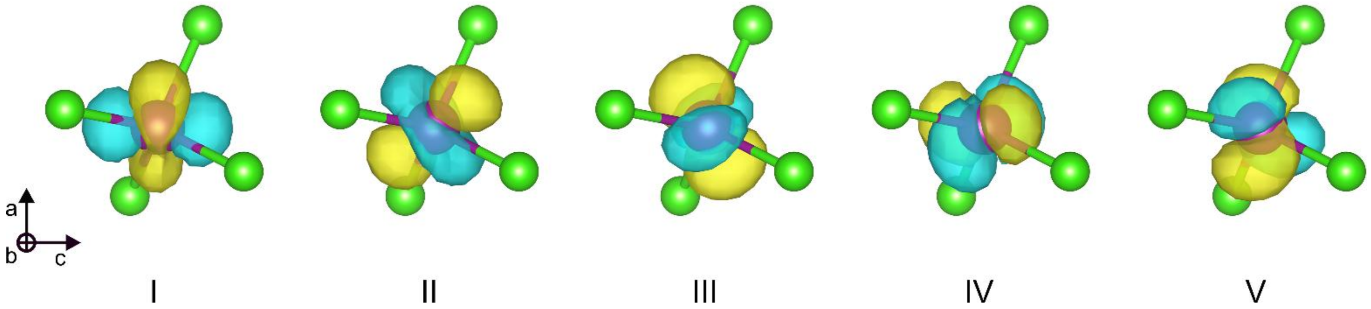

Fig. S33 Visualization of active space, composed of five 3d orbitals (I–V), applied for the computational procedures for Mn2 centers (ground state geometry). The orbitals were plotted with an isosurface level of 0.034. The above-presented active space was used for calculations employing all considered theory levels for Mn2 centers (Tables S14–S21).

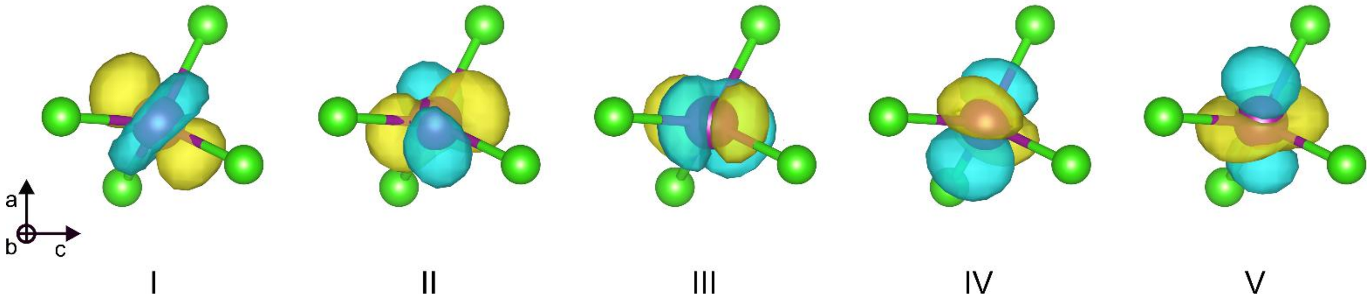

Fig. S34 Visualization of active space, composed of five 3d orbitals (I–V), applied for the computational procedures for Mn2 centers (optimized excited state geometry). The orbitals were plotted with an isosurface level of 0.034. The above-presented active space was used for calculations employing all considered theory levels for Mn2 centers (Tables S14–S21).

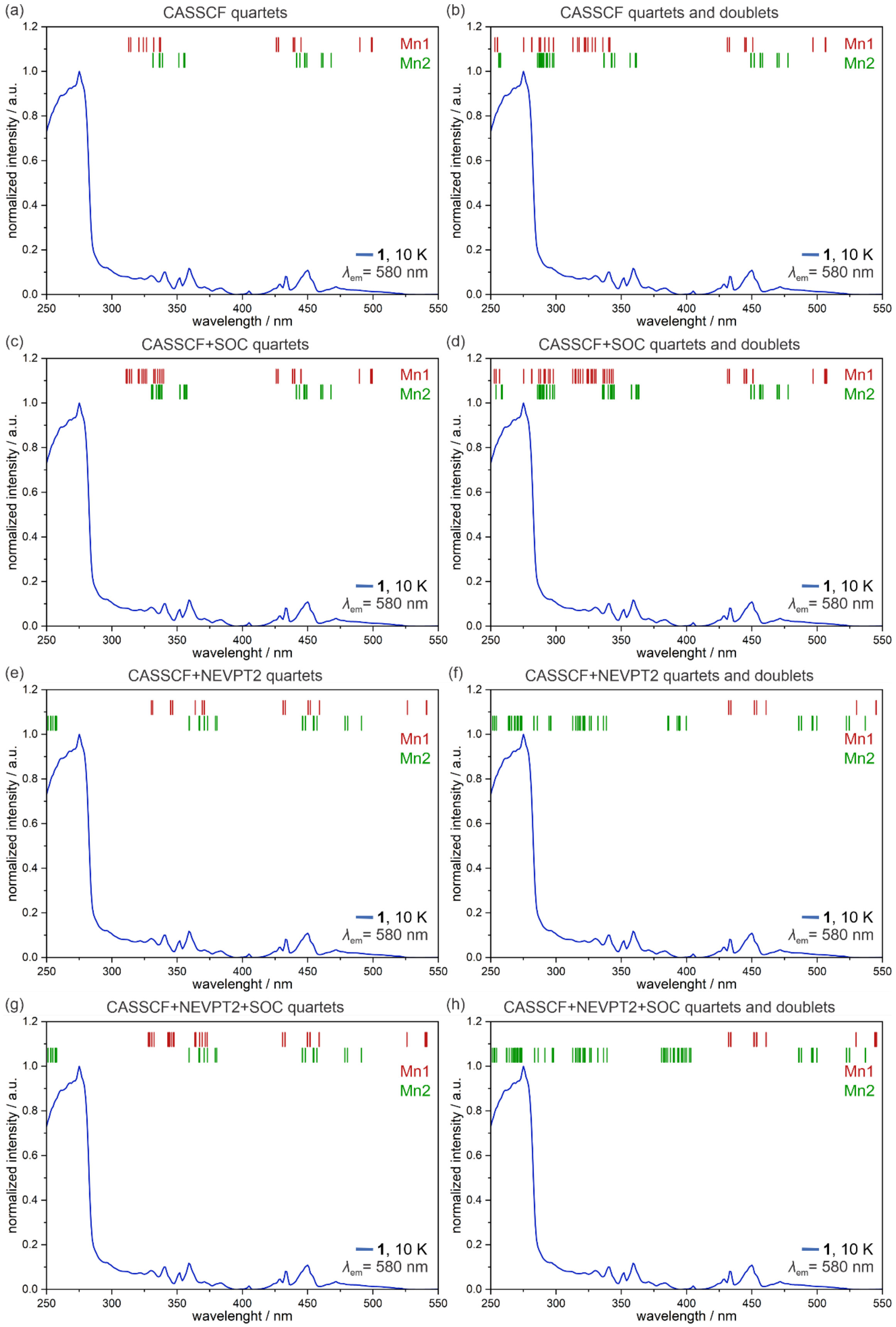

Fig. S35 The *ab initio*-calculated energy splitting schemes for Mn1 (dark red bars) and Mn2 (green bars) centers of **1** using various theory levels, including CASSCF with the quartet excited states (a, Table S14), CASSCF with the quartet and doublet excited states (b, Table S15), CASSCF+SOC with the quartet excited states (c, Table S16), CASSCF+SOC with the quartet and doublet excited states (d, Table S17), CASSCF+NEVPT2 with the quartet excited states (e, Table S18), CASSCF+NEVPT2 with the quartet and doublet excited states (f, Table S19), CASSCF+NEVPT2+SOC with the quartet excited states (g, Table S20), and CASSCF+NEVPT2+SOC with the quartet and doublet excited states (h, Table S21). All energy-splitting schemes are shown on the experimental excitation spectrum of **1**.

Table S14 Energies of excited states (corresponding to optical transition energies from the ground sextet state, *ΔE*, Fig. S35) obtained from CASSCF-type *ab initio* calculations for Mn1 and Mn2 centers of **1** (Figure 1) when taking into account only the quartet excited states, shown together with the resulting wavelengths of optical transitions and the values of energies and wavelengths after the scaling procedure (energy shift for scaling set at –8000 cm^{-1}, see the Comment on theoretical calculations of optical transitions in Mn(II) complexes in **1** above).

**Mn1** complexes					**Mn2** complexes				
State	*ΔE* / cm^{-1}	Wavelength / nm	Scaled *ΔE* / cm^{-1}	Scaled wavelength / nm	State	*ΔE* / cm^{-1}	Wavelength / nm	Scaled *ΔE* / cm^{-1}	Scaled wavelength / nm
1	28031.8	356.73771	20031.8	499.20626	1	29369.2	340.49276	21369.2	467.96324
2	28047.8	356.5342	20047.8	498.80785	2	29667.7	337.06691	21667.7	461.51645
3	28416	351.91441	20416	489.81191	3	29722.9	336.44093	21722.9	460.34369
4	30479.4	328.09045	22479.4	444.85173	4	30254.8	330.52607	22254.8	449.34127
5	30725.7	325.46045	22725.7	440.03045	5	30323.8	329.77397	22323.8	447.95241
6	30784.6	324.83774	22784.6	438.89294	6	30341.3	329.58377	22341.3	447.60153
7	31391	318.56265	23391	427.51486	7	30526.7	327.58208	22526.7	443.91766
8	31395.7	318.51496	23395.7	427.42897	8	30647.2	326.29408	22647.2	441.55569
9	31473.6	317.7266	23473.6	426.0105	9	30656.1	326.19935	22656.1	441.38223
10	37657	265.55488	29657	337.18852	10	36106.3	276.95998	28106.3	355.79212
11	37724.5	265.07972	29724.5	336.42282	11	36164.1	276.51732	28164.1	355.06194
12	38119.8	262.33086	30119.8	332.00752	12	36468.2	274.2115	28468.2	351.26914
13	38628.5	258.87622	30628.5	326.4933	13	37533.3	266.43008	29533.3	338.60083
14	38861.5	257.32409	30861.5	324.02832	14	37701.8	265.23933	29701.8	336.67993
15	39179.7	255.23422	31179.7	320.7215	15	37729.7	265.04319	29729.7	336.36397
16	39813	251.17424	31813	314.33691	16	38162.7	262.03597	30162.7	331.53531
17	39954.5	250.2847	31954.5	312.94497	17	38178.6	261.92684	30178.6	331.36063
18	52563.1	190.24753	44563.1	224.40091	18	51236.3	195.17412	43236.3	231.28714
19	52700.7	189.7508	44700.7	223.71014	19	51294.6	194.9523	43294.6	230.97569
20	53103.2	188.31257	45103.2	221.71376	20	51339.7	194.78104	43339.7	230.73533
21	53515.4	186.8621	45515.4	219.70586	21	51550.2	193.98567	43550.2	229.62007
22	54542.6	183.34293	46542.6	214.85693	22	51666.8	193.54789	43666.8	229.00693
23	54765.9	182.59537	46765.9	213.83102	23	51701.3	193.41873	43701.3	228.82614
24	54831.4	182.37725	46831.4	213.53195	24	51929.8	192.56766	43929.8	227.63591

Table S15 (part 1/3) Energies of excited states (corresponding to optical transition energies from the ground sextet state, *ΔE*, Fig. S35) obtained from CASSCF-type *ab initio* calculations for Mn1 and Mn2 centers of **1** (Figure 1) when taking into account the quartet and doublet excited states, shown together with the resulting wavelengths of optical transitions and the values of energies and wavelengths after the scaling procedure (energy shift for scaling set at –8000 cm^{-1}, see the Comment on theoretical calculations of optical transitions in Mn(II) complexes in **1** above).

**Mn1** complexes					**Mn2** complexes				
State	*ΔE* / cm^{-1}	Wavelength / nm	Scaled *ΔE* / cm^{-1}	Scaled wavelength / nm	State	*ΔE* / cm^{-1}	Wavelength / nm	Scaled *ΔE* / cm^{-1}	Scaled wavelength / nm
1	27743.7	360.44219	19743.7	506.49068	1	28934.4	20934.4	20934.4	477.68267
2	27758.7	360.24742	19758.7	506.10617	2	29244.9	21244.9	21244.9	470.7012
3	28129.8	355.49488	20129.8	496.77592	3	29302.9	21302.9	21302.9	469.41966
4	30193.3	331.19931	22193.3	450.58644	4	29826.4	21826.4	21826.4	458.16076
5	30441.2	328.50216	22441.2	445.60897	5	29899.4	21899.4	21899.4	456.63352
6	30501.2	327.85595	22501.2	444.42074	6	29917.4	21917.4	21917.4	456.2585
7	31106.5	321.47622	23106.5	432.77866	7	30139.5	22139.5	22139.5	451.68138
8	31110.7	321.43282	23110.7	432.7	8	30246.4	22246.4	22246.4	449.51093
9	31184.7	320.67007	23184.7	431.31893	9	30259.3	22259.3	22259.3	449.25043
10	37304.7	268.06274	29304.7	341.24219	10	35662.3	27662.3	27662.3	361.50284
11	37378	267.53705	29378	340.39077	11	35724.1	27724.1	27724.1	360.69701
12	37775.8	264.71974	29775.8	335.8432	12	36034.8	28034.8	28034.8	356.69953
13	38297.1	261.11638	30297.1	330.06459	13	37004.2	29004.2	29004.2	344.77765
14	38519.8	259.60675	30519.8	327.65614	14	37178.5	29178.5	29178.5	342.7181
15	38828	257.5461	30828	324.38043	15	37209.4	29209.4	29209.4	342.35554
16	38985	256.50891	30985	322.73681	16	37683.6	29683.6	29683.6	336.88636
17	39081.6	255.87489	31081.6	321.73376	17	37709.9	29709.9	29709.9	336.58814
18	39476.1	253.31783	31476.1	317.70137	18	41527	33527	33527	298.26707
19	39612.2	252.44748	31612.2	316.33357	19	41644.4	33644.4	33644.4	297.22628
20	39955.8	250.27656	31955.8	312.93224	20	41878.4	33878.4	33878.4	295.17333
21	41555.5	240.64203	33555.5	298.01374	21	42087.5	34087.5	34087.5	293.36267
22	41932.3	238.47964	33932.3	294.70446	22	42188.3	34188.3	34188.3	292.49773
23	41956.4	238.34266	33956.4	294.49529	23	42406.2	34406.2	34406.2	290.64529
24	42300.9	236.40159	34300.9	291.53754	24	42439.5	34439.5	34439.5	290.36426
25	42320.6	236.29155	34320.6	291.3702	25	42506.5	34506.5	34506.5	289.80047
26	42703.2	234.17449	34703.2	288.15786	26	42621.9	34621.9	34621.9	288.83452
27	42837	233.44305	34837	287.05112	27	42686.4	34686.4	34686.4	288.29743
28	43506.5	229.85071	35506.5	281.63857	28	42779.4	34779.4	34779.4	287.52652
29	43547.8	229.63273	35547.8	281.31136	29	42852.5	34852.5	34852.5	286.92346
30	44341.1	225.5244	36341.1	275.17054	30	42980.5	34980.5	34980.5	285.87356
31	47171.6	211.99196	39171.6	255.28699	31	46836.5	38836.5	38836.5	257.48973
32	47208.6	211.82581	39208.6	255.04609	32	46908.7	38908.7	38908.7	257.01193
33	47490.5	210.56843	39490.5	253.22546	33	47052.9	39052.9	39052.9	256.06293
34	48192.6	207.50074	40192.6	248.80202	34	48238.7	40238.7	40238.7	248.51697
35	48335.8	206.88599	40335.8	247.91872	35	48443.7	40443.7	40443.7	247.2573
36	50186.9	199.25518	42186.9	237.04041	36	49585.9	41585.9	41585.9	240.46612

Table S15 (part 2/3) Energies of excited states (corresponding to optical transition energies from the ground sextet state, *ΔE*, Fig. S35) obtained from CASSCF-type *ab initio* calculations for Mn1 and Mn2 centers of **1** (Figure 1) when taking into account the quartet and doublet excited states, shown together with the resulting wavelengths of optical transitions and the values of energies and wavelengths after the scaling procedure (energy shift for scaling set at –8000 cm^{-1}, see the Comment on theoretical calculations of optical transitions in Mn(II) complexes in **1** above).

**Mn1** complexes					**Mn2** complexes				
State	*ΔE* / cm^{-1}	Wavelength / nm	Scaled *ΔE* / cm^{-1}	Scaled wavelength / nm	State	*ΔE* / cm^{-1}	Wavelength / nm	Scaled *ΔE* / cm^{-1}	Scaled wavelength / nm
37	50386.1	198.46743	42386.1	235.9264	37	49715.7	41715.7	41715.7	239.7179
38	50614.1	197.5734	42614.1	234.66411	38	49744.1	41744.1	41744.1	239.55481
39	52098.3	191.94484	44098.3	226.76611	39	50171.5	42171.5	42171.5	237.12697
40	52156.6	191.73029	44156.6	226.46671	40	50531	42531	42531	235.12262
41	52235.9	191.43922	44235.9	226.06073	41	50593.5	42593.5	42593.5	234.77761
42	52244	191.40954	44244	226.01935	42	50644	42644	42644	234.49958
43	52551.6	190.28916	44551.6	224.45883	43	50706.6	42706.6	42706.6	234.15584
44	52633.6	189.9927	44633.6	224.04646	44	50790.1	42790.1	42790.1	233.69892
45	52902.5	189.02698	44902.5	222.70475	45	50821.9	42821.9	42821.9	233.52537
46	53031.3	188.56788	45031.3	222.06776	46	50932.7	42932.7	42932.7	232.92269
47	53884.8	185.58109	45884.8	217.93709	47	51016.4	43016.4	43016.4	232.46948
48	53897.5	185.53736	45897.5	217.87679	48	51052.4	43052.4	43052.4	232.27509
49	54015.3	185.13273	46015.3	217.31902	49	51291	43291	43291	230.9949
50	54071.4	184.94065	46071.4	217.0544	50	52516.3	44516.3	44516.3	224.63682
51	54295.7	184.17665	46295.7	216.00278	51	52672.8	44672.8	44672.8	223.84986
52	54361	183.95541	46361	215.69854	52	52701.1	44701.1	44701.1	223.70814
53	54826.1	182.39488	46826.1	213.55612	53	53049.7	45049.7	45049.7	221.97706
54	55013.7	181.7729	47013.7	212.70396	54	53161.7	45161.7	45161.7	221.42656
55	55368.9	180.6068	47368.9	211.10898	55	53193.4	45193.4	45193.4	221.27125
56	55655.3	179.67741	47655.3	209.84025	56	53256.3	45256.3	45256.3	220.96371
57	55961.2	178.69524	47961.2	208.50187	57	53352.5	45352.5	45352.5	220.49501
58	56273.7	177.70291	48273.7	207.15213	58	54181.3	46181.3	46181.3	216.53786
59	56860	175.87056	48860	204.66639	59	54237.5	46237.5	46237.5	216.27467
60	57236.7	174.71308	49236.7	203.10053	60	54371.6	46371.6	46371.6	215.64923
61	57444.1	174.08228	49444.1	202.2486	61	54629.2	46629.2	46629.2	214.45789
62	57681.5	173.36581	49681.5	201.28217	62	54862.4	46862.4	46862.4	213.39069
63	57768	173.10622	49768	200.93233	63	54993.5	46993.5	46993.5	212.79539
64	58025.2	172.33891	50025.2	199.89925	64	55034.7	47034.7	47034.7	212.60899
65	58319	171.4707	50319	198.73209	65	55205.2	47205.2	47205.2	211.84107
66	58393.6	171.25164	50393.6	198.4379	66	55278.4	47278.4	47278.4	211.51308
67	59128.3	169.12375	51128.3	195.5864	67	55321.3	47321.3	47321.3	211.32133
68	59642.3	167.66624	51642.3	193.63971	68	55574.6	47574.6	47574.6	210.1962
69	59687	167.54067	51687	193.47225	69	55639.4	47639.4	47639.4	209.91028
70	61111.3	163.63586	53111.3	188.28385	70	58380	50380	50380	198.49146
71	61218.3	163.34985	53218.3	187.90529	71	58549.8	50549.8	50549.8	197.82472
72	61277.3	163.19257	53277.3	187.6972	72	58594.9	50594.9	50594.9	197.64838

Table S15 (part 3/3) Energies of excited states (corresponding to optical transition energies from the ground sextet state, *ΔE*, Fig. S35) obtained from CASSCF-type *ab initio* calculations for Mn1 and Mn2 centers of **1** (Figure 1) when taking into account the quartet and doublet excited states, shown together with the resulting wavelengths of optical transitions and the values of energies and wavelengths after the scaling procedure (energy shift for scaling set at –8000 cm^{-1}, see the Comment on theoretical calculations of optical transitions in Mn(II) complexes in **1** above).

**Mn1** complexes					**Mn2** complexes				
State	*ΔE* / cm^{-1}	Wavelength / nm	Scaled *ΔE* / cm^{-1}	Scaled wavelength / nm	State	*ΔE* / cm^{-1}	Wavelength / nm	Scaled *ΔE* / cm^{-1}	Scaled wavelength / nm
73	61693.8	162.09084	53693.8	186.24124	73	58911.2	50911.2	50911.2	196.42043
74	61771.1	161.888	53771.1	185.97351	74	58932	50932	50932	196.34022
75	61991.6	161.31218	53991.6	185.214	75	59046.7	51046.7	51046.7	195.89905
76	62054.8	161.14789	54054.8	184.99745	76	59095.4	51095.4	51095.4	195.71233
77	66294.3	150.84253	58294.3	171.54336	77	64836.2	56836.2	56836.2	175.9442
78	74589.2	134.06767	66589.2	150.1745	78	71915.2	63915.2	63915.2	156.45731
79	74615.6	134.02023	66615.6	150.11499	79	71934.1	63934.1	63934.1	156.41105
80	75135.8	133.09235	67135.8	148.95183	80	72105.3	64105.3	64105.3	155.99334
81	75279.2	132.83882	67279.2	148.63435	81	72145	64145	64145	155.8968
82	75390.1	132.64341	67390.1	148.38975	82	72264.6	64264.6	64264.6	155.60666
83	81513.8	122.67861	73513.8	136.02888	83	79264.9	71264.9	71264.9	140.32153
84	81604.7	122.54196	73604.7	135.86089	84	79283.3	71283.3	71283.3	140.28531
85	82142.8	121.73922	74142.8	134.87486	85	79302.5	71302.5	71302.5	140.24754
86	82197.5	121.6582	74197.5	134.77543	86	79332.9	71332.9	71332.9	140.18777
87	82503.8	121.20654	74503.8	134.22134	87	79365.9	71365.9	71365.9	140.12294
88	82812.1	120.7553	74812.1	133.66822	88	79407.9	71407.9	71407.9	140.04053
89	82828.5	120.73139	74828.5	133.63892	89	79445.9	71445.9	71445.9	139.96604
90	82843	120.71026	74843	133.61303	90	79459.3	71459.3	71459.3	139.9398
91	83382.4	119.92939	75382.4	132.65696	91	79496.1	71496.1	71496.1	139.86777
92	100305.4	99.69553	92305.4	108.33602	92	97098.8	89098.8	89098.8	112.23496
93	100438.3	99.56361	92438.3	108.18027	93	97136	89136	89136	112.18812
94	100627.9	99.37602	92627.9	107.95883	94	97179.8	89179.8	89179.8	112.13302
95	108850.4	91.86921	100850.4	99.15677	95	104842	96842	96842	103.26098
96	108953.1	91.78261	100953.1	99.0559	96	104942.9	96942.9	96942.9	103.15351
97	109005.9	91.73815	101005.9	99.00412	97	104963.9	96963.9	96963.9	103.13117
98	109063.7	91.68954	101063.7	98.9475	98	105040.8	97040.8	97040.8	103.04944
99	109401.9	91.40609	101401.9	98.61748	99	105070.4	97070.4	97070.4	103.01802

Table S16 (part 1/3) Energies (*E*) of ground and excited states (the latter corresponding to optical transition energies from the ground sextet state, Fig. S35) obtained from CASSCF-SOC-type *ab initio* calculations for Mn1 and Mn2 centers of **1** (Figure 1) when taking into account only the quartet excited states, shown together with the resulting wavelengths of optical transitions and the values of energies and wavelengths after the scaling procedure (energy shift for scaling set at –8000 cm^{-1}, see the Comment on theoretical calculations of optical transitions in Mn(II) complexes in **1** above).

**Mn1** complexes					**Mn2** complexes				
State	*E* / cm^{-1}	Wavelength / nm	Scaled *E* / cm^{-1}	Scaled wavelength / nm	State	*E* / cm^{-1}	Wavelength / nm	Scaled *E* / cm^{-1}	Scaled wavelength / nm
1	0	-	-8000	-	1	0	-	-8000	-
2	0	-	-8000	-	2	0	-	-8000	-
3	0	-	-8000	-	3	0	-	-8000	-
4	0	-	-8000	-	4	0	-	-8000	-
5	0	-	-8000	-	5	0	-	-8000	-
6	28021.3	356.87138	20021.3	499.46807	6	29377.3	340.39888	21377.3	467.78592
7	28021.3	356.87138	20021.3	499.46807	7	29377.3	340.39888	21377.3	467.78592
8	28036	356.68426	20036	499.10162	8	29377.6	340.3954	21377.6	467.77936
9	28036	356.68426	20036	499.10162	9	29377.6	340.3954	21377.6	467.77936
10	28059.4	356.38681	20059.4	498.5194	10	29672.8	337.00898	21672.8	461.40785
11	28059.4	356.38681	20059.4	498.5194	11	29672.8	337.00898	21672.8	461.40785
12	28074.8	356.19132	20074.8	498.13697	12	29676.1	336.9715	21676.1	461.3376
13	28074.8	356.19132	20074.8	498.13697	13	29676.1	336.9715	21676.1	461.3376
14	28424.5	351.80918	20424.5	489.60807	14	29732.1	336.33682	21732.1	460.14881
15	28424.5	351.80918	20424.5	489.60807	15	29732.1	336.33682	21732.1	460.14881
16	28427.1	351.777	20427.1	489.54575	16	29735.7	336.2961	21735.7	460.0726
17	28427.1	351.777	20427.1	489.54575	17	29735.7	336.2961	21735.7	460.0726
18	30474.9	328.13889	22474.9	444.9408	18	30254.5	330.52934	22254.5	449.34732
19	30474.9	328.13889	22474.9	444.9408	19	30254.5	330.52934	22254.5	449.34732
20	30484.4	328.03664	22484.4	444.75281	20	30258.8	330.48237	22258.8	449.26052
21	30484.4	328.03664	22484.4	444.75281	21	30258.8	330.48237	22258.8	449.26052
22	30721	325.51024	22721	440.12147	22	30322.6	329.78702	22322.6	447.97649
23	30721	325.51024	22721	440.12147	23	30322.6	329.78702	22322.6	447.97649
24	30744.2	325.2646	22744.2	439.67253	24	30338.2	329.61745	22338.2	447.66364
25	30744.2	325.2646	22744.2	439.67253	25	30338.2	329.61745	22338.2	447.66364
26	30803.4	324.63949	22803.4	438.5311	26	30356.7	329.41657	22356.7	447.29321
27	30803.4	324.63949	22803.4	438.5311	27	30356.7	329.41657	22356.7	447.29321
28	30816	324.50675	22816	438.28892	28	30370.8	329.26363	22370.8	447.01128
29	30816	324.50675	22816	438.28892	29	30370.8	329.26363	22370.8	447.01128
30	31401.1	318.46018	23401.1	427.33034	30	30537.8	327.46301	22537.8	443.69903
31	31401.1	318.46018	23401.1	427.33034	31	30537.8	327.46301	22537.8	443.69903
32	31403.3	318.43787	23403.3	427.29017	32	30537.9	327.46194	22537.9	443.69706
33	31403.3	318.43787	23403.3	427.29017	33	30537.9	327.46194	22537.9	443.69706
34	31409	318.38008	23409	427.18612	34	30658.4	326.17488	22658.4	441.33743
35	31409	318.38008	23409	427.18612	35	30658.4	326.17488	22658.4	441.33743
36	31411	318.35981	23411	427.14963	36	30658.6	326.17275	22658.6	441.33353

Table S16 (part 2/3) Energies (*E*) of ground and excited states (the latter corresponding to optical transition energies from the ground sextet state, Fig. S35) obtained from CASSCF-SOC-type *ab initio* calculations for Mn1 and Mn2 centers of **1** (Figure 1) when taking into account only the quartet excited states, shown together with the resulting wavelengths of optical transitions and the values of energies and wavelengths after the scaling procedure (energy shift for scaling set at –8000 cm^{-1}, see the Comment on theoretical calculations of optical transitions in Mn(II) complexes in **1** above).

**Mn1** complexes					**Mn2** complexes				
State	*E* / cm^{-1}	Wavelength / nm	Scaled *E* / cm^{-1}	Scaled wavelength / nm	State	*E* / cm^{-1}	Wavelength / nm	Scaled *E* / cm^{-1}	Scaled wavelength / nm
37	31411	318.35981	23411	427.14963	37	30658.6	326.17275	22658.6	441.33353
38	31485.1	317.61055	23485.1	425.80189	38	30667.8	326.07491	22667.8	441.15441
39	31485.1	317.61055	23485.1	425.80189	39	30667.8	326.07491	22667.8	441.15441
40	31485.4	317.60753	23485.4	425.79645	40	30668	326.07278	22668	441.15052
41	31485.4	317.60753	23485.4	425.79645	41	30668	326.07278	22668	441.15052
42	37449	267.02983	29449	339.5701	42	35985.7	277.88816	27985.7	357.32535
43	37449	267.02983	29449	339.5701	43	35985.7	277.88816	27985.7	357.32535
44	37557	266.26195	29557	338.32933	44	36022.7	277.60273	28022.7	356.85355
45	37557	266.26195	29557	338.32933	45	36022.7	277.60273	28022.7	356.85355
46	37699.6	265.2548	29699.6	336.70487	46	36098.8	277.01752	28098.8	355.88708
47	37699.6	265.2548	29699.6	336.70487	47	36098.8	277.01752	28098.8	355.88708
48	37854.6	264.16869	29854.6	334.95676	48	36148.7	276.63512	28148.7	355.25619
49	37854.6	264.16869	29854.6	334.95676	49	36148.7	276.63512	28148.7	355.25619
50	38004.4	263.12743	30004.4	333.28445	50	36397	274.74792	28397	352.14987
51	38004.4	263.12743	30004.4	333.28445	51	36397	274.74792	28397	352.14987
52	38110.3	262.39626	30110.3	332.11227	52	36412.4	274.63172	28412.4	351.959
53	38110.3	262.39626	30110.3	332.11227	53	36412.4	274.63172	28412.4	351.959
54	38612.5	258.98349	30612.5	326.66394	54	37558.1	266.25415	29558.1	338.31674
55	38612.5	258.98349	30612.5	326.66394	55	37558.1	266.25415	29558.1	338.31674
56	38722.7	258.24645	30722.7	325.49223	56	37576.2	266.1259	29576.2	338.1097
57	38722.7	258.24645	30722.7	325.49223	57	37576.2	266.1259	29576.2	338.1097
58	38857	257.35389	30857	324.07557	58	37671.4	265.45337	29671.4	337.02488
59	38857	257.35389	30857	324.07557	59	37671.4	265.45337	29671.4	337.02488
60	38962	256.66034	30962	322.97655	60	37735.8	265.00034	29735.8	336.29497
61	38962	256.66034	30962	322.97655	61	37735.8	265.00034	29735.8	336.29497
62	39152.2	255.41349	31152.2	321.00462	62	37801.3	264.54117	29801.3	335.55583
63	39152.2	255.41349	31152.2	321.00462	63	37801.3	264.54117	29801.3	335.55583
64	39225.8	254.93425	31225.8	320.248	64	37938.3	263.58587	29938.3	334.0203
65	39225.8	254.93425	31225.8	320.248	65	37938.3	263.58587	29938.3	334.0203
66	39737.1	251.654	31737.1	315.08865	66	38179.8	261.91861	30179.8	331.34746
67	39737.1	251.654	31737.1	315.08865	67	38179.8	261.91861	30179.8	331.34746
68	39900.9	250.62091	31900.9	313.47078	68	38227.3	261.59315	30227.3	330.82677
69	39900.9	250.62091	31900.9	313.47078	69	38227.3	261.59315	30227.3	330.82677
70	40077.6	249.51594	32077.6	311.74402	70	38236.9	261.52748	30236.9	330.72173
71	40077.6	249.51594	32077.6	311.74402	71	38236.9	261.52748	30236.9	330.72173
72	40153	249.04739	32153	311.01297	72	38273.2	261.27943	30273.2	330.32517

Table S16 (part 3/3) Energies (*E*) of ground and excited states (the latter corresponding to optical transition energies from the ground sextet state, Fig. S35) obtained from CASSCF-SOC-type *ab initio* calculations for Mn1 and Mn2 centers of **1** (Figure 1) when taking into account only the quartet excited states, shown together with the resulting wavelengths of optical transitions and the values of energies and wavelengths after the scaling procedure (energy shift for scaling set at –8000 cm^{-1}, see the Comment on theoretical calculations of optical transitions in Mn(II) complexes in **1** above).

**Mn1** complexes					**Mn2** complexes				
State	E / cm^{-1}	Wavelength / nm	Scaled E / cm^{-1}	Scaled wavelength / nm	State	E / cm^{-1}	Wavelength / nm	Scaled E / cm^{-1}	Scaled wavelength / nm
73	40153	249.04739	32153	311.01297	73	38273.2	261.27943	30273.2	330.32517
74	52576.5	190.19904	44576.5	224.33345	74	51250	195.12195	43250	231.21387
75	52576.5	190.19904	44576.5	224.33345	75	51250	195.12195	43250	231.21387
76	52580.6	190.18421	44580.6	224.31282	76	51254.3	195.10558	43254.3	231.19089
77	52580.6	190.18421	44580.6	224.31282	77	51254.3	195.10558	43254.3	231.19089
78	52717.7	189.68961	44717.7	223.6251	78	51310.5	194.89188	43310.5	230.89089
79	52717.7	189.68961	44717.7	223.6251	79	51310.5	194.89188	43310.5	230.89089
80	52729.2	189.64824	44729.2	223.5676	80	51321.5	194.85011	43321.5	230.83227
81	52729.2	189.64824	44729.2	223.5676	81	51321.5	194.85011	43321.5	230.83227
82	53127	188.22821	45127	221.59683	82	51363.7	194.69002	43363.7	230.60763
83	53127	188.22821	45127	221.59683	83	51363.7	194.69002	43363.7	230.60763
84	53132.3	188.20943	45132.3	221.5708	84	51370.7	194.66349	43370.7	230.57041
85	53132.3	188.20943	45132.3	221.5708	85	51370.7	194.66349	43370.7	230.57041
86	53525.9	186.82544	45525.9	219.65519	86	51554.1	193.97099	43554.1	229.59951
87	53525.9	186.82544	45525.9	219.65519	87	51554.1	193.97099	43554.1	229.59951
88	53529.8	186.81183	45529.8	219.63637	88	51556.3	193.96272	43556.3	229.58791
89	53529.8	186.81183	45529.8	219.63637	89	51556.3	193.96272	43556.3	229.58791
90	54534.6	183.36982	46534.6	214.89386	90	51668.2	193.54264	43668.2	228.99959
91	54534.6	183.36982	46534.6	214.89386	91	51668.2	193.54264	43668.2	228.99959
92	54562.1	183.2774	46562.1	214.76695	92	51689	193.46476	43689	228.89057
93	54562.1	183.2774	46562.1	214.76695	93	51689	193.46476	43689	228.89057
94	54749.8	182.64907	46749.8	213.90466	94	51724.6	193.33161	43724.6	228.70421
95	54749.8	182.64907	46749.8	213.90466	95	51724.6	193.33161	43724.6	228.70421
96	54799.4	182.48375	46799.4	213.67795	96	51753.2	193.22477	43753.2	228.55471
97	54799.4	182.48375	46799.4	213.67795	97	51753.2	193.22477	43753.2	228.55471
98	54880.8	182.21309	46880.8	213.30694	98	51968.6	192.42389	43968.6	227.43503
99	54880.8	182.21309	46880.8	213.30694	99	51968.6	192.42389	43968.6	227.43503
100	54918.7	182.08734	46918.7	213.13464	100	51971	192.415	43971	227.42262
101	54918.7	182.08734	46918.7	213.13464	101	51971	192.415	43971	227.42262

Table S17 (part 1/7) Energies (*E*) of ground and excited states (the latter corresponding to optical transition energies from the ground sextet state, Fig. S35) obtained from CASSCF-SOC-type *ab initio* calculations for Mn1 and Mn2 centers of **1** (Figure 1) when taking into account the quartet and doublet excited states, shown together with the resulting wavelengths of optical transitions and the values of energies and wavelengths after the scaling procedure (energy shift for scaling set at –8000 cm^{-1}, see the Comment on theoretical calculations of optical transitions in Mn(II) complexes in **1** above).

**Mn1** complexes					**Mn2** complexes				
State	*E* / cm^{-1}	Wavelength / nm	Scaled *E* / cm^{-1}	Scaled wavelength / nm	State	*E* / cm^{-1}	Wavelength / nm	Scaled *E* / cm^{-1}	Scaled wavelength / nm
1	0	-	-8000	-	1	0	-	-8000	-
2	0	-	-8000	-	2	0	-	-8000	-
3	0	-	-8000	-	3	0	-	-8000	-
4	0	-	-8000	-	4	0	-	-8000	-
5	0	-	-8000	-	5	0	-	-8000	-
6	27712.6	360.84669	19712.6	27712.6	6	28926.1	345.70855	20926.1	477.87213
7	27712.6	360.84669	19712.6	27712.6	7	28926.1	345.70855	20926.1	477.87213
8	27732.6	360.58646	19732.6	27732.6	8	28933.1	345.62491	20933.1	477.71233
9	27732.6	360.58646	19732.6	27732.6	9	28933.1	345.62491	20933.1	477.71233
10	27756.2	360.27987	19756.2	27756.2	10	29235.7	342.04757	21235.7	470.90513
11	27756.2	360.27987	19756.2	27756.2	11	29235.7	342.04757	21235.7	470.90513
12	27775.1	360.03471	19775.1	27775.1	12	29242	341.97387	21242	470.76546
13	27775.1	360.03471	19775.1	27775.1	13	29242	341.97387	21242	470.76546
14	28124.9	355.55682	20124.9	28124.9	14	29296.3	341.34003	21296.3	469.56514
15	28124.9	355.55682	20124.9	28124.9	15	29296.3	341.34003	21296.3	469.56514
16	28126.4	355.53786	20126.4	28126.4	16	29304.2	341.24801	21304.2	469.39101
17	28126.4	355.53786	20126.4	28126.4	17	29304.2	341.24801	21304.2	469.39101
18	30173.1	331.42103	22173.1	30173.1	18	29815.6	335.39489	21815.6	458.38758
19	30173.1	331.42103	22173.1	30173.1	19	29815.6	335.39489	21815.6	458.38758
20	30185.8	331.2816	22185.8	30185.8	20	29819.6	335.3499	21819.6	458.30354
21	30185.8	331.2816	22185.8	30185.8	21	29819.6	335.3499	21819.6	458.30354
22	30421.6	328.71381	22421.6	30421.6	22	29887.7	334.5858	21887.7	456.87761
23	30421.6	328.71381	22421.6	30421.6	23	29887.7	334.5858	21887.7	456.87761
24	30448	328.4288	22448	30448	24	29903	334.41461	21903	456.55846
25	30448	328.4288	22448	30448	25	29903	334.41461	21903	456.55846
26	30509.4	327.76784	22509.4	30509.4	26	29922.1	334.20114	21922.1	456.16068
27	30509.4	327.76784	22509.4	30509.4	27	29922.1	334.20114	21922.1	456.16068
28	30520.7	327.64648	22520.7	30520.7	28	29928.6	334.12856	21928.6	456.02546
29	30520.7	327.64648	22520.7	30520.7	29	29928.6	334.12856	21928.6	456.02546
30	31102.5	321.51756	23102.5	31102.5	30	30138.1	331.80592	22138.1	451.70995
31	31102.5	321.51756	23102.5	31102.5	31	30138.1	331.80592	22138.1	451.70995
32	31105.7	321.48449	23105.7	31105.7	32	30139.2	331.79381	22139.2	451.6875
33	31105.7	321.48449	23105.7	31105.7	33	30139.2	331.79381	22139.2	451.6875
34	31110.3	321.43695	23110.3	31110.3	34	30245.1	330.63207	22245.1	449.5372
35	31110.3	321.43695	23110.3	31110.3	35	30245.1	330.63207	22245.1	449.5372
36	31114.1	321.39769	23114.1	31114.1	36	30246.5	330.61677	22246.5	449.50891

Table S17 (part 2/7) Energies (*E*) of ground and excited states (the latter corresponding to optical transition energies from the ground sextet state, Fig. S35) obtained from CASSCF-SOC-type *ab initio* calculations for Mn1 and Mn2 centers of **1** (Figure 1) when taking into account the quartet and doublet excited states, shown together with the resulting wavelengths of optical transitions and the values of energies and wavelengths after the scaling procedure (energy shift for scaling set at –8000 cm^{-1}, see the Comment on theoretical calculations of optical transitions in Mn(II) complexes in **1** above).

**Mn1** complexes					**Mn2** complexes				
State	*E* / cm^{-1}	Wavelength / nm	Scaled *E* / cm^{-1}	Scaled wavelength / nm	State	*E* / cm^{-1}	Wavelength / nm	Scaled *E* / cm^{-1}	Scaled wavelength / nm
37	31114.1	321.39769	23114.1	432.63636	37	30246.5	330.61677	22246.5	449.50891
38	31182.5	320.6927	23182.5	431.35986	38	30258.3	330.48783	22258.3	449.27061
39	31182.5	320.6927	23182.5	431.35986	39	30258.3	330.48783	22258.3	449.27061
40	31184.6	320.6711	23184.6	431.32079	40	30260.8	330.46053	22260.8	449.22015
41	31184.6	320.6711	23184.6	431.32079	41	30260.8	330.46053	22260.8	449.22015
42	37091.4	269.60427	29091.4	343.7442	42	35518.9	281.54025	27518.9	363.38662
43	37091.4	269.60427	29091.4	343.7442	43	35518.9	281.54025	27518.9	363.38662
44	37199.7	268.81937	29199.7	342.46927	44	35555	281.25439	27555	362.91054
45	37199.7	268.81937	29199.7	342.46927	45	35555	281.25439	27555	362.91054
46	37342.8	267.78924	29342.8	340.79911	46	35633.9	280.63165	27633.9	361.87436
47	37342.8	267.78924	29342.8	340.79911	47	35633.9	280.63165	27633.9	361.87436
48	37493.8	266.71076	29493.8	339.05431	48	35686	280.22194	27686	361.19338
49	37493.8	266.71076	29493.8	339.05431	49	35686	280.22194	27686	361.19338
50	37649.5	265.60778	29649.5	337.27382	50	35944.6	278.20591	27944.6	357.85089
51	37649.5	265.60778	29649.5	337.27382	51	35944.6	278.20591	27944.6	357.85089
52	37755	264.86558	29755	336.07797	52	35961.4	278.07594	27961.4	357.63588
53	37755	264.86558	29755	336.07797	53	35961.4	278.07594	27961.4	357.63588
54	38254.8	261.4051	30254.8	330.52607	54	37022.3	270.10748	29022.3	344.56263
55	38254.8	261.4051	30254.8	330.52607	55	37022.3	270.10748	29022.3	344.56263
56	38363.9	260.66172	30363.9	329.33846	56	37042	269.96382	29042	344.3289
57	38363.9	260.66172	30363.9	329.33846	57	37042	269.96382	29042	344.3289
58	38494.9	259.77467	30494.9	327.92369	58	37135.8	269.28193	29135.8	343.22037
59	38494.9	259.77467	30494.9	327.92369	59	37135.8	269.28193	29135.8	343.22037
60	38599.4	259.07138	30599.4	326.80379	60	37203	268.79553	29203	342.43057
61	38599.4	259.07138	30599.4	326.80379	61	37203	268.79553	29203	342.43057
62	38786.7	257.82034	30786.7	324.81559	62	37276.9	268.26265	29276.9	341.56622
63	38786.7	257.82034	30786.7	324.81559	63	37276.9	268.26265	29276.9	341.56622
64	38840.9	257.46056	30840.9	324.24475	64	37415.7	267.26748	29415.7	339.95451
65	38840.9	257.46056	30840.9	324.24475	65	37415.7	267.26748	29415.7	339.95451
66	38875	257.23473	30875	323.88664	66	37697.2	265.27169	29697.2	336.73208
67	38875	257.23473	30875	323.88664	67	37697.2	265.27169	29697.2	336.73208
68	39201.8	255.09033	31201.8	320.49433	68	37743	264.94979	29743	336.21356
69	39201.8	255.09033	31201.8	320.49433	69	37743	264.94979	29743	336.21356
70	39387.4	253.8883	31387.4	318.59918	70	37757.2	264.85015	29757.2	336.05312
71	39387.4	253.8883	31387.4	318.59918	71	37757.2	264.85015	29757.2	336.05312
72	39546	252.87008	31546	316.9974	72	37789.7	264.62237	29789.7	335.6865

Table S17 (part 3/7) Energies (*E*) of ground and excited states (the latter corresponding to optical transition energies from the ground sextet state, Fig. S35) obtained from CASSCF-SOC-type *ab initio* calculations for Mn1 and Mn2 centers of **1** (Figure 1) when taking into account the quartet and doublet excited states, shown together with the resulting wavelengths of optical transitions and the values of energies and wavelengths after the scaling procedure (energy shift for scaling set at –8000 cm^{-1}, see the Comment on theoretical calculations of optical transitions in Mn(II) complexes in **1** above).

**Mn1** complexes					**Mn2** complexes				
State	*E* / cm^{-1}	Wavelength / nm	Scaled *E* / cm^{-1}	Scaled wavelength / nm	State	*E* / cm^{-1}	Wavelength / nm	Scaled *E* / cm^{-1}	Scaled wavelength / nm
73	39546	252.87008	31546	316.9974	73	37789.7	264.62237	29789.7	335.6865
74	39717.5	251.77818	31717.5	315.28336	74	41472.4	241.12422	33472.4	298.7536
75	39717.5	251.77818	31717.5	315.28336	75	41472.4	241.12422	33472.4	298.7536
76	39784.4	251.3548	31784.4	314.61975	76	41625.2	240.23909	33625.2	297.396
77	39784.4	251.3548	31784.4	314.61975	77	41625.2	240.23909	33625.2	297.396
78	39978.9	250.13194	31978.9	312.70619	78	41871.6	238.82536	33871.6	295.23258
79	39978.9	250.13194	31978.9	312.70619	79	41871.6	238.82536	33871.6	295.23258
80	41578.5	240.50892	33578.5	297.80961	80	42123.3	237.3983	34123.3	293.05489
81	41578.5	240.50892	33578.5	297.80961	81	42123.3	237.3983	34123.3	293.05489
82	41859.1	238.89668	33859.1	295.34158	82	42174.5	237.1101	34174.5	292.61584
83	41859.1	238.89668	33859.1	295.34158	83	42174.5	237.1101	34174.5	292.61584
84	42003.1	238.07767	34003.1	294.09083	84	42393.6	235.88466	34393.6	290.75177
85	42003.1	238.07767	34003.1	294.09083	85	42393.6	235.88466	34393.6	290.75177
86	42279	236.52404	34279	291.7238	86	42436.9	235.64398	34436.9	290.38618
87	42279	236.52404	34279	291.7238	87	42436.9	235.64398	34436.9	290.38618
88	42389	235.91026	34389	290.79066	88	42523.1	235.1663	34523.1	289.66113
89	42389	235.91026	34389	290.79066	89	42523.1	235.1663	34523.1	289.66113
90	42713.8	234.11637	34713.8	288.06987	90	42643.5	234.50233	34643.5	288.65444
91	42713.8	234.11637	34713.8	288.06987	91	42643.5	234.50233	34643.5	288.65444
92	42865.3	233.28893	34865.3	286.81813	92	42712.9	234.12131	34712.9	288.07734
93	42865.3	233.28893	34865.3	286.81813	93	42712.9	234.12131	34712.9	288.07734
94	43517.7	229.79156	35517.7	281.54976	94	42790.6	233.69619	34790.6	287.43396
95	43517.7	229.79156	35517.7	281.54976	95	42790.6	233.69619	34790.6	287.43396
96	43561.5	229.56051	35561.5	281.20299	96	42861.8	233.30798	34861.8	286.84692
97	43561.5	229.56051	35561.5	281.20299	97	42861.8	233.30798	34861.8	286.84692
98	44342.9	225.51525	36342.9	275.15691	98	42985.7	232.6355	34985.7	285.83107
99	44342.9	225.51525	36342.9	275.15691	99	42985.7	232.6355	34985.7	285.83107
100	46953.8	212.97531	38953.8	256.71436	100	46616	214.51862	38616	258.96002
101	46953.8	212.97531	38953.8	256.71436	101	46616	214.51862	38616	258.96002
102	47359.8	211.14954	39359.8	254.06633	102	46730.8	213.99163	38730.8	258.19245
103	47359.8	211.14954	39359.8	254.06633	103	46730.8	213.99163	38730.8	258.19245
104	47572.4	210.20592	39572.4	252.70138	104	47375.3	211.08046	39375.3	253.96632
105	47572.4	210.20592	39572.4	252.70138	105	47375.3	211.08046	39375.3	253.96632
106	48113.3	207.84274	40113.3	249.29388	106	48147.8	207.69381	40147.8	249.07965
107	48113.3	207.84274	40113.3	249.29388	107	48147.8	207.69381	40147.8	249.07965
108	48396.8	206.62523	40396.8	247.54436	108	48366.9	206.75297	40366.9	247.72772

Table S17 (part 4/7) Energies (*E*) of ground and excited states (the latter corresponding to optical transition energies from the ground sextet state, Fig. S35) obtained from CASSCF-SOC-type *ab initio* calculations for Mn1 and Mn2 centers of **1** (Figure 1) when taking into account the quartet and doublet excited states, shown together with the resulting wavelengths of optical transitions and the values of energies and wavelengths after the scaling procedure (energy shift for scaling set at –8000 cm^{-1}, see the Comment on theoretical calculations of optical transitions in Mn(II) complexes in **1** above).

**Mn1** complexes					**Mn2** complexes				
State	*E* / cm^{-1}	Wavelength / nm	Scaled *E* / cm^{-1}	Scaled wavelength / nm	State	*E* / cm^{-1}	Wavelength / nm	Scaled *E* / cm^{-1}	Scaled wavelength / nm
109	48396.8	206.62523	40396.8	247.54436	109	48366.9	206.75297	40366.9	247.72772
110	50215	199.14368	42215	236.88262	110	49587.1	201.66535	41587.1	240.45918
111	50215	199.14368	42215	236.88262	111	49587.1	201.66535	41587.1	240.45918
112	50373.6	198.51668	42373.6	235.996	112	49755.2	200.98402	41755.2	239.49113
113	50373.6	198.51668	42373.6	235.996	113	49755.2	200.98402	41755.2	239.49113
114	50679.5	197.31844	42679.5	234.30453	114	49856.5	200.57565	41856.5	238.91152
115	50679.5	197.31844	42679.5	234.30453	115	49856.5	200.57565	41856.5	238.91152
116	51978.8	192.38613	43978.8	227.38228	116	50105.3	199.57969	42105.3	237.49979
117	51978.8	192.38613	43978.8	227.38228	117	50105.3	199.57969	42105.3	237.49979
118	52040.9	192.15655	44040.9	227.06166	118	50467.4	198.14772	42467.4	235.47474
119	52040.9	192.15655	44040.9	227.06166	119	50467.4	198.14772	42467.4	235.47474
120	52108.2	191.90838	44108.2	226.71521	120	50541.1	197.85877	42541.1	235.06679
121	52108.2	191.90838	44108.2	226.71521	121	50541.1	197.85877	42541.1	235.06679
122	52188.3	191.61383	44188.3	226.30425	122	50569.4	197.74805	42569.4	234.91052
123	52188.3	191.61383	44188.3	226.30425	123	50569.4	197.74805	42569.4	234.91052
124	52287.8	191.2492	44287.8	225.79582	124	50603.4	197.61518	42603.4	234.72305
125	52287.8	191.2492	44287.8	225.79582	125	50603.4	197.61518	42603.4	234.72305
126	52356.4	190.99862	44356.4	225.44661	126	50632.4	197.50199	42632.4	234.56338
127	52356.4	190.99862	44356.4	225.44661	127	50632.4	197.50199	42632.4	234.56338
128	52445.6	190.67376	44445.6	224.99415	128	50649.9	197.43376	42649.9	234.46714
129	52445.6	190.67376	44445.6	224.99415	129	50649.9	197.43376	42649.9	234.46714
130	52634.1	189.9909	44634.1	224.04395	130	50706.7	197.2126	42706.7	234.1553
131	52634.1	189.9909	44634.1	224.04395	131	50706.7	197.2126	42706.7	234.1553
132	52750.9	189.57023	44750.9	223.45919	132	50784.6	196.91009	42784.6	233.72896
133	52750.9	189.57023	44750.9	223.45919	133	50784.6	196.91009	42784.6	233.72896
134	53001.3	188.67462	45001.3	222.2158	134	50804.8	196.8318	42804.8	233.61866
135	53001.3	188.67462	45001.3	222.2158	135	50804.8	196.8318	42804.8	233.61866
136	53073.9	188.41653	45073.9	221.85788	136	50966.7	196.20654	42966.7	232.73838
137	53073.9	188.41653	45073.9	221.85788	137	50966.7	196.20654	42966.7	232.73838
138	53091.1	188.35549	45091.1	221.77325	138	50984	196.13997	42984	232.64471
139	53091.1	188.35549	45091.1	221.77325	139	50984	196.13997	42984	232.64471
140	53802.7	185.86428	45802.7	218.32774	140	51007.4	196.04998	43007.4	232.51812
141	53802.7	185.86428	45802.7	218.32774	141	51007.4	196.04998	43007.4	232.51812
142	53956.4	185.33483	45956.4	217.59755	142	51062.3	195.8392	43062.3	232.22169
143	53956.4	185.33483	45956.4	217.59755	143	51062.3	195.8392	43062.3	232.22169
144	54016.6	185.12828	46016.6	217.31288	144	51131.8	195.57301	43131.8	231.8475

Table S17 (part 5/7) Energies (*E*) of ground and excited states (the latter corresponding to optical transition energies from the ground sextet state, Fig. S35) obtained from CASSCF-SOC-type *ab initio* calculations for Mn1 and Mn2 centers of **1** (Figure 1) when taking into account the quartet and doublet excited states, shown together with the resulting wavelengths of optical transitions and the values of energies and wavelengths after the scaling procedure (energy shift for scaling set at –8000 cm^{-1}, see the Comment on theoretical calculations of optical transitions in Mn(II) complexes in **1** above).

**Mn1** complexes					**Mn2** complexes				
State	*E* / cm^{-1}	Wavelength / nm	Scaled *E* / cm^{-1}	Scaled wavelength / nm	State	*E* / cm^{-1}	Wavelength / nm	Scaled *E* / cm^{-1}	Scaled wavelength / nm
145	54016.6	185.12828	46016.6	217.31288	145	51131.8	195.57301	43131.8	231.8475
146	54053.8	185.00087	46053.8	217.13735	146	51198.8	195.31708	43198.8	231.48791
147	54053.8	185.00087	46053.8	217.13735	147	51198.8	195.31708	43198.8	231.48791
148	54099.2	184.84562	46099.2	216.9235	148	51311.2	194.88922	43311.2	230.88716
149	54099.2	184.84562	46099.2	216.9235	149	51311.2	194.88922	43311.2	230.88716
150	54226.7	184.411	46226.7	216.3252	150	51337	194.79128	43337	230.74971
151	54226.7	184.411	46226.7	216.3252	151	51337	194.79128	43337	230.74971
152	54276.1	184.24316	46276.1	216.09427	152	52509.4	190.44209	44509.4	224.67164
153	54276.1	184.24316	46276.1	216.09427	153	52509.4	190.44209	44509.4	224.67164
154	54333.1	184.04987	46333.1	215.82843	154	52635.2	189.98693	44635.2	224.03843
155	54333.1	184.04987	46333.1	215.82843	155	52635.2	189.98693	44635.2	224.03843
156	54399.9	183.82387	46399.9	215.51771	156	52775.4	189.48222	44775.4	223.33692
157	54399.9	183.82387	46399.9	215.51771	157	52775.4	189.48222	44775.4	223.33692
158	54946.3	181.99588	46946.3	213.00933	158	53082.9	188.38458	45082.9	221.81359
159	54946.3	181.99588	46946.3	213.00933	159	53082.9	188.38458	45082.9	221.81359
160	55122.7	181.41346	47122.7	212.21195	160	53149.1	188.14994	45149.1	221.48836
161	55122.7	181.41346	47122.7	212.21195	161	53149.1	188.14994	45149.1	221.48836
162	55313	180.78933	47313	211.3584	162	53216.4	187.912	45216.4	221.15869
163	55313	180.78933	47313	211.3584	163	53216.4	187.912	45216.4	221.15869
164	55694.3	179.55159	47694.3	209.66866	164	53285	187.67008	45285	220.82367
165	55694.3	179.55159	47694.3	209.66866	165	53285	187.67008	45285	220.82367
166	56048.6	178.41659	48048.6	208.12261	166	53379	187.33959	45379	220.36625
167	56048.6	178.41659	48048.6	208.12261	167	53379	187.33959	45379	220.36625
168	56340.9	177.49095	48340.9	206.86417	168	54175.5	184.58528	46175.5	216.56506
169	56340.9	177.49095	48340.9	206.86417	169	54175.5	184.58528	46175.5	216.56506
170	56896.2	175.75866	48896.2	204.51487	170	54271.8	184.25775	46271.8	216.11435
171	56896.2	175.75866	48896.2	204.51487	171	54271.8	184.25775	46271.8	216.11435
172	57256.3	174.65327	49256.3	203.01972	172	54445.1	183.67126	46445.1	215.30797
173	57256.3	174.65327	49256.3	203.01972	173	54445.1	183.67126	46445.1	215.30797
174	57454.6	174.05047	49454.6	202.20566	174	54600.9	183.14716	46600.9	214.58813
175	57454.6	174.05047	49454.6	202.20566	175	54600.9	183.14716	46600.9	214.58813
176	57742.8	173.18176	49742.8	201.03412	176	54934.5	182.03497	46934.5	213.06289
177	57742.8	173.18176	49742.8	201.03412	177	54934.5	182.03497	46934.5	213.06289
178	57850.7	172.85876	49850.7	200.59899	178	55061.5	181.6151	47061.5	212.48791
179	57850.7	172.85876	49850.7	200.59899	179	55061.5	181.6151	47061.5	212.48791
180	58068.8	172.20952	50068.8	199.72518	180	55145.3	181.33912	47145.3	212.11022

Table S17 (part 6/7) Energies (*E*) of ground and excited states (the latter corresponding to optical transition energies from the ground sextet state, Fig. S35) obtained from CASSCF-SOC-type *ab initio* calculations for Mn1 and Mn2 centers of **1** (Figure 1) when taking into account the quartet and doublet excited states, shown together with the resulting wavelengths of optical transitions and the values of energies and wavelengths after the scaling procedure (energy shift for scaling set at –8000 cm^{-1}, see the Comment on theoretical calculations of optical transitions in Mn(II) complexes in **1** above).

**Mn1** complexes					**Mn2** complexes				
State	*E* / cm^{-1}	Wavelength / nm	Scaled *E* / cm^{-1}	Scaled wavelength / nm	State	*E* / cm^{-1}	Wavelength / nm	Scaled *E* / cm^{-1}	Scaled wavelength / nm
181	58068.8	172.20952	50068.8	199.72518	181	55145.3	181.33912	47145.3	212.11022
182	58342.6	171.40134	50342.6	198.63893	182	55224.4	181.07938	47224.4	211.75494
183	58342.6	171.40134	50342.6	198.63893	183	55224.4	181.07938	47224.4	211.75494
184	58470.1	171.02759	50470.1	198.13711	184	55328.9	180.73737	47328.9	211.2874
185	58470.1	171.02759	50470.1	198.13711	185	55328.9	180.73737	47328.9	211.2874
186	59172.2	168.99828	51172.2	195.41861	186	55390.7	180.53572	47390.7	211.01187
187	59172.2	168.99828	51172.2	195.41861	187	55390.7	180.53572	47390.7	211.01187
188	59615.9	167.74049	51615.9	193.73875	188	55655.8	179.67579	47655.8	209.83805
189	59615.9	167.74049	51615.9	193.73875	189	55655.8	179.67579	47655.8	209.83805
190	59806.6	167.20563	51806.6	193.0256	190	55870.1	178.98661	47870.1	208.89867
191	59806.6	167.20563	51806.6	193.0256	191	55870.1	178.98661	47870.1	208.89867
192	61129	163.58848	53129	188.22112	192	58362.3	171.34349	50362.3	198.56123
193	61129	163.58848	53129	188.22112	193	58362.3	171.34349	50362.3	198.56123
194	61234.6	163.30637	53234.6	187.84775	194	58581.5	170.70235	50581.5	197.70074
195	61234.6	163.30637	53234.6	187.84775	195	58581.5	170.70235	50581.5	197.70074
196	61295.3	163.14465	53295.3	187.63381	196	58654.9	170.48874	50654.9	197.41427
197	61295.3	163.14465	53295.3	187.63381	197	58654.9	170.48874	50654.9	197.41427
198	61707.7	162.05433	53707.7	186.19304	198	58930.2	169.69228	50930.2	196.34716
199	61707.7	162.05433	53707.7	186.19304	199	58930.2	169.69228	50930.2	196.34716
200	61813.1	161.778	53813.1	185.82836	200	58971	169.57488	50971	196.18999
201	61813.1	161.778	53813.1	185.82836	201	58971	169.57488	50971	196.18999
202	61921.5	161.4948	53921.5	185.45478	202	59076.1	169.27319	51076.1	195.78629
203	61921.5	161.4948	53921.5	185.45478	203	59076.1	169.27319	51076.1	195.78629
204	62311.1	160.48505	54311.1	184.12442	204	59126.1	169.13005	51126.1	195.59481
205	62311.1	160.48505	54311.1	184.12442	205	59126.1	169.13005	51126.1	195.59481
206	66310.2	150.80636	58310.2	171.49658	206	64849.7	154.20272	56849.7	175.90242
207	66310.2	150.80636	58310.2	171.49658	207	64849.7	154.20272	56849.7	175.90242
208	74611.1	134.02831	66611.1	150.12513	208	71938.9	139.00685	63938.9	156.39931
209	74611.1	134.02831	66611.1	150.12513	209	71938.9	139.00685	63938.9	156.39931
210	74639.3	133.97768	66639.3	150.0616	210	71960.6	138.96493	63960.6	156.34625
211	74639.3	133.97768	66639.3	150.0616	211	71960.6	138.96493	63960.6	156.34625
212	75146.1	133.0741	67146.1	148.92898	212	72114.7	138.66798	64114.7	155.97047
213	75146.1	133.0741	67146.1	148.92898	213	72114.7	138.66798	64114.7	155.97047
214	75288	132.82329	67288	148.61491	214	72163	138.57517	64163	155.85306
215	75288	132.82329	67288	148.61491	215	72163	138.57517	64163	155.85306
216	75446.5	132.54425	67446.5	148.26566	216	72312.3	138.28906	64312.3	155.49125

Table S17 (part 7/7) Energies (E) of ground and excited states (the latter corresponding to optical transition energies from the ground sextet state, Fig. S35) obtained from CASSCF-SOC-type *ab initio* calculations for Mn1 and Mn2 centers of **1** (Figure 1) when taking into account the quartet and doublet excited states, shown together with the resulting wavelengths of optical transitions and the values of energies and wavelengths after the scaling procedure (energy shift for scaling set at –8000 cm^{-1}, see the Comment on theoretical calculations of optical transitions in Mn(II) complexes in **1** above).

**Mn1** complexes					**Mn2** complexes				
State	E / cm^{-1}	Wavelength / nm	Scaled E / cm^{-1}	Scaled wavelength / nm	State	E / cm^{-1}	Wavelength / nm	Scaled E / cm^{-1}	Scaled wavelength / nm
217	75446.5	132.54425	67446.5	148.26566	217	72312.3	138.28906	64312.3	155.49125
218	81530.2	122.65394	73530.2	135.99854	218	79276.1	126.14142	71276.1	140.29948
219	81530.2	122.65394	73530.2	135.99854	219	79276.1	126.14142	71276.1	140.29948
220	81622.6	122.51509	73622.6	135.82786	220	79294.1	126.11279	71294.1	140.26406
221	81622.6	122.51509	73622.6	135.82786	221	79294.1	126.11279	71294.1	140.26406
222	82151.6	121.72617	74151.6	134.85886	222	79322.5	126.06764	71322.5	140.20821
223	82151.6	121.72617	74151.6	134.85886	223	79322.5	126.06764	71322.5	140.20821
224	82226.6	121.61515	74226.6	134.72259	224	79349	126.02553	71349	140.15613
225	82226.6	121.61515	74226.6	134.72259	225	79349	126.02553	71349	140.15613
226	82528.9	121.16968	74528.9	134.17614	226	79391.9	125.95743	71391.9	140.07191
227	82528.9	121.16968	74528.9	134.17614	227	79391.9	125.95743	71391.9	140.07191
228	82830.4	120.72862	74830.4	133.63553	228	79438.6	125.88339	71438.6	139.98035
229	82830.4	120.72862	74830.4	133.63553	229	79438.6	125.88339	71438.6	139.98035
230	82852	120.69715	74852	133.59696	230	79469.4	125.8346	71469.4	139.92002
231	82852	120.69715	74852	133.59696	231	79469.4	125.8346	71469.4	139.92002
232	82873.2	120.66627	74873.2	133.55914	232	79482.4	125.81402	71482.4	139.89458
233	82873.2	120.66627	74873.2	133.55914	233	79482.4	125.81402	71482.4	139.89458
234	83404.6	119.89746	75404.6	132.6179	234	79521.6	125.752	71521.6	139.8179
235	83404.6	119.89746	75404.6	132.6179	235	79521.6	125.752	71521.6	139.8179
236	100294.3	99.70656	92294.3	108.34905	236	97089.3	102.99796	89089.3	112.24693
237	100294.3	99.70656	92294.3	108.34905	237	97089.3	102.99796	89089.3	112.24693
238	100461	99.54112	92461	108.15371	238	97156.3	102.92693	89156.3	112.16257
239	100461	99.54112	92461	108.15371	239	97156.3	102.92693	89156.3	112.16257
240	100644.3	99.35982	92644.3	107.93972	240	97192	102.88913	89192	112.11768
241	100644.3	99.35982	92644.3	107.93972	241	97192	102.88913	89192	112.11768
242	108863.5	91.85815	100863.5	99.14389	242	104867.4	95.35852	96867.4	103.23391
243	108863.5	91.85815	100863.5	99.14389	243	104867.4	95.35852	96867.4	103.23391
244	108965.5	91.77217	100965.5	99.04373	244	104956.7	95.27739	96956.7	103.13882
245	108965.5	91.77217	100965.5	99.04373	245	104956.7	95.27739	96956.7	103.13882
246	109034.8	91.71384	101034.8	98.9758	246	104993.8	95.24372	96993.8	103.09937
247	109034.8	91.71384	101034.8	98.9758	247	104993.8	95.24372	96993.8	103.09937
248	109101.2	91.65802	101101.2	98.91079	248	105055.2	95.18805	97055.2	103.03415
249	109101.2	91.65802	101101.2	98.91079	249	105055.2	95.18805	97055.2	103.03415
250	109438.5	91.37552	101438.5	98.5819	250	105115.5	95.13345	97115.5	102.97017
251	109438.5	91.37552	101438.5	98.5819	251	105115.5	95.13345	97115.5	102.97017

Table S18 Energies of excited states (corresponding to optical transition energies from the ground sextet state, *ΔE*, Fig. S35) obtained from CASSCF-NEVPT2-type *ab initio* calculations for Mn1 and Mn2 centers of **1** (Figure 1) when taking into account only the quartet excited states, shown together with the resulting wavelengths of optical transitions and the values of energies and wavelengths after the scaling procedure (energy shift for scaling set at –5000 cm^{-1}, see the Comment on theoretical calculations of optical transitions in Mn(II) complexes in **1** above).

**Mn1** complexes					**Mn2** complexes				
State	*ΔE* / cm^{-1}	Wavelength / nm	Scaled *ΔE* / cm^{-1}	Scaled wavelength / nm	State	*ΔE* / cm^{-1}	Wavelength / nm	Scaled *ΔE* / cm^{-1}	Scaled wavelength / nm
1	23481.9	425.85992	18481.9	541.06991	1	25358.2	394.34976	20358.2	491.20256
2	23490.8	425.69857	18490.8	540.80948	2	25809.6	387.45273	20809.6	480.54744
3	23999.3	416.67882	18999.3	526.33518	3	25899	386.11529	20899	478.49179
4	26789.9	373.275	21789.9	458.92822	4	26878.1	372.05011	21878.1	457.07808
5	27124	368.67719	22124	451.99783	5	26987.2	370.54604	21987.2	454.81007
6	27206.9	367.55382	22206.9	450.31049	6	27014.9	370.16609	22014.9	454.23781
7	28100.4	355.86682	23100.4	432.89294	7	27314.1	366.11128	22314.1	448.14714
8	28105.4	355.80351	23105.4	432.79926	8	27417.8	364.72656	22417.8	446.0741
9	28199	354.6225	23199	431.05306	9	27430.4	364.55903	22430.4	445.82353
10	31978.3	312.71206	26978.3	370.66828	10	31288.1	319.61033	26288.1	380.40026
11	32091.9	311.60511	27091.9	369.11402	11	31369.3	318.78301	26369.3	379.22888
12	32489	307.79648	27489	363.78188	12	31797.1	314.49409	26797.1	373.17471
13	33862	295.31628	28862	346.47634	13	31976.5	312.72966	26976.5	370.69301
14	33976.2	294.32367	28976.2	345.11082	14	32230.6	310.26416	27230.6	367.23392
15	34003.1	294.09083	29003.1	344.79073	15	32265	309.93336	27265	366.77058
16	35190.7	284.16599	30190.7	331.22783	16	32833.3	304.56884	27833.3	359.28187
17	35289.2	283.37282	30289.2	330.15068	17	32845.9	304.452	27845.9	359.1193
18	45415.2	220.1906	40415.2	247.43166	18	43816.1	228.22661	38816.1	257.62506
19	45589.8	219.34731	40589.8	246.36731	19	43899.7	227.79199	38899.7	257.07139
20	46020.1	217.29636	41020.1	243.78293	20	43966.1	227.44797	38966.1	256.63333
21	46226	216.32847	41226	242.56537	21	44249.8	225.98972	39249.8	254.77837
22	47765.2	209.35744	42765.2	233.83499	22	44462.8	224.90711	39462.8	253.40321
23	48030.1	208.20277	43030.1	232.39546	23	44508.7	224.67518	39508.7	253.10881
24	48124.1	207.79609	43124.1	231.8889	24	44822.1	223.10423	39822.1	251.11684

Table S19 (part 1/3) Energies of excited states (corresponding to optical transition energies from the ground sextet state, *ΔE*, Fig. S35) obtained from CASSCF-NEVPT2-type *ab initio* calculations for Mn1 and Mn2 centers of **1** (Figure 1) when taking into account the quartet and doublet excited states, shown together with the resulting wavelengths of optical transitions and the values of energies and wavelengths after the scaling procedure (energy shift for scaling set at –5000 cm^{-1}, see the Comment on theoretical calculations of optical transitions in Mn(II) complexes in **1** above).

**Mn1** complexes					**Mn2** complexes				
State	*ΔE* / cm^{-1}	Wavelength / nm	Scaled *ΔE* / cm^{-1}	Scaled wavelength / nm	State	*ΔE* / cm^{-1}	Wavelength / nm	Scaled *ΔE* / cm^{-1}	Scaled wavelength / nm
1	23341.6	428.41965	18341.6	545.20871	1	23630.6	423.18011	18630.6	536.75137
2	23352	428.22885	18352	544.89974	2	24063	415.57578	19063	524.5764
3	23867.6	418.97803	18867.6	530.00912	3	24148.1	414.11126	19148.1	522.24503
4	26700.2	374.52903	21700.2	460.82525	4	25016.5	399.73617	20016.5	499.58784
5	27043.2	369.77872	22043.2	453.65464	5	25133.1	397.88168	20133.1	496.6945
6	27128.6	368.61467	22128.6	451.90387	6	25162.6	397.41521	20162.6	495.96778
7	28041.7	356.61176	23041.7	433.99576	7	25493.8	392.25223	20493.8	487.95245
8	28046.3	356.55327	23046.3	433.90913	8	25571.2	391.06495	20571.2	486.11651
9	28137.3	355.40013	23137.3	432.20255	9	25583.1	390.88304	20583.1	485.83547
Due to the computational limitations further states were not calculated for Mn1 complexes in this approach.					10	30014.3	333.17452	25014.3	399.77133
					11	30322.2	329.79137	25322.2	394.91039
					12	30356.6	329.41766	25356.6	394.37464
					13	30378.7	329.17801	25378.7	394.03122
					14	30464.5	328.25091	25464.5	392.70357
					15	30888.1	323.74928	25888.1	386.27787
					16	30930.7	323.30338	25930.7	385.64327
					17	30934.2	323.26681	25934.2	385.59123
					18	34535.7	289.55545	29535.7	338.57332
					19	34729.9	287.93633	29729.9	336.36171
					20	35115.4	284.77534	30115.4	332.05602
					21	35586.7	281.00386	30586.7	326.93949
					22	35717.3	279.97637	30717.3	325.54945
					23	36051.4	277.38174	31051.4	322.04667
					24	36107	276.95461	31107	321.47105
					25	36193.1	276.29576	31193.1	320.58372
					26	36374.8	274.9156	31374.8	318.72713
					27	36483.1	274.09951	31483.1	317.63073
					28	36612	273.13449	31612	316.33557
					29	36735.1	272.21921	31735.1	315.10851
					30	36974.8	270.45447	31974.8	312.74629
					31	38775.4	257.89547	33775.4	296.07347
					32	38821.8	257.58723	33821.8	295.66729
					33	38946.3	256.7638	33946.3	294.58291
					34	39996.8	250.02	34996.8	285.74041
					35	40331.1	247.94761	35331.1	283.03676
					36	41533.9	240.76718	36533.9	273.71838

Table S19 (part 2/3) Energies of excited states (corresponding to optical transition energies from the ground sextet state, *ΔE*, Fig. S35) obtained from CASSCF-NEVPT2-type *ab initio* calculations for Mn1 and Mn2 centers of **1** (Figure 1) when taking into account the quartet and doublet excited states, shown together with the resulting wavelengths of optical transitions and the values of energies and wavelengths after the scaling procedure (energy shift for scaling set at –5000 cm^{-1}, see the Comment on theoretical calculations of optical transitions in Mn(II) complexes in **1** above).

**Mn1** complexes					**Mn2** complexes				
State	*ΔE* / cm^{-1}	Wavelength / nm	Scaled *ΔE* / cm^{-1}	Scaled wavelength / nm	State	*ΔE* / cm^{-1}	Wavelength / nm	Scaled *ΔE* / cm^{-1}	Scaled wavelength / nm
					37	41619.6	240.27141	36619.6	273.07781
					38	41686.3	239.88697	36686.3	272.58132
					39	41689.2	239.87028	36689.2	272.55977
					40	41890.7	238.71647	36890.7	271.07103
					41	41932.1	238.48078	36932.1	270.76716
					42	42017.5	237.99607	37017.5	270.1425
					43	42238	236.75363	37238	268.54289
					44	42287.6	236.47594	37287.6	268.18567
					45	42602	234.73076	37602	265.9433
					46	42621.5	234.62337	37621.5	265.80546
					47	42792.3	233.6869	37792.3	264.60417
					48	42890	233.15458	37890	263.92188
					49	42944.3	232.85977	37944.3	263.5442
					50	44314.9	225.65774	39314.9	254.35649
					51	44519.4	224.62118	39519.4	253.04028
					52	44559.6	224.41853	39559.6	252.78314
					53	44749.9	223.46419	39749.9	251.57296
					54	45018.8	222.12942	40018.8	249.88256
					55	45297.5	220.76274	40297.5	248.15435
					56	45390.4	220.3109	40390.4	247.58358
					57	45409.5	220.21824	40409.5	247.46656
					58	45586.7	219.36223	40586.7	246.38613
					59	45815.8	218.26531	40815.8	245.00316
					60	45865.5	218.0288	40865.5	244.70519
					61	46006.7	217.35965	41006.7	243.86259
					62	46054	217.13641	41054	243.58162
					63	46073.5	217.0445	41073.5	243.46598
					64	46410.2	215.46988	41410.2	241.4864
					65	46582.4	214.67335	41582.4	240.48636
					66	46607.1	214.55958	41607.1	240.3436
					67	46678.7	214.23047	41678.7	239.93071
					68	47313.2	211.35751	42313.2	236.33287
					69	47476.8	210.62919	42476.8	235.42263
					70	47679.7	209.73286	42679.7	234.30343
					71	48019.5	208.24873	43019.5	232.45272
					72	48119	207.81812	43119	231.91632

Table S19 (part 3/3) Energies of excited states (corresponding to optical transition energies from the ground sextet state, *ΔE*, Fig. S35) obtained from CASSCF-NEVPT2-type *ab initio* calculations for Mn1 and Mn2 centers of **1** (Figure 1) when taking into account the quartet and doublet excited states, shown together with the resulting wavelengths of optical transitions and the values of energies and wavelengths after the scaling procedure (energy shift for scaling set at –5000 cm^{-1}, see the Comment on theoretical calculations of optical transitions in Mn(II) complexes in **1** above).

**Mn1** complexes					**Mn2** complexes				
State	*ΔE* / cm^{-1}	Wavelength / nm	Scaled *ΔE* / cm^{-1}	Scaled wavelength / nm	State	*ΔE* / cm^{-1}	Wavelength / nm	Scaled *ΔE* / cm^{-1}	Scaled wavelength / nm
					73	48432.8	206.47165	43432.8	230.24074
					74	48452.7	206.38685	43452.7	230.1353
					75	48597.1	205.7736	43597.1	229.37305
					76	48775.4	205.02138	43775.4	228.4388
					77	52406	190.81785	47406	210.94376
					78	58828.1	169.98679	53828.1	185.77657
					79	58894.9	169.79399	53894.9	185.54631
					80	59160.9	169.03056	54160.9	184.63504
					81	59204.8	168.90522	54204.8	184.48551
					82	59326.3	168.55931	54326.3	184.07291
					83	64689.9	154.58364	59689.9	167.53253
					84	64784.2	154.35862	59784.2	167.26827
					85	64800.3	154.32027	59800.3	167.22324
					86	64800.6	154.31956	59800.6	167.2224
					87	64916.3	154.04452	59916.3	166.89949
					88	65033.4	153.76714	60033.4	166.57394
					89	65051.5	153.72436	60051.5	166.52373
					90	65078.5	153.66058	60078.5	166.4489
					91	65105.7	153.59638	60105.7	166.37357
					92	77691.4	128.71438	72691.4	137.56786
					93	77732.3	128.64665	72732.3	137.4905
					94	77797.5	128.53883	72797.5	137.36735
					95	84731.1	118.02042	79731.1	125.42157
					96	84832.9	117.87879	79832.9	125.26164
					97	84882.5	117.80991	79882.5	125.18386
					98	84936.1	117.73557	79936.1	125.09992
					99	84976.4	117.67973	79976.4	125.03689

Table S20 (part 1/3) Energies (*E*) of ground and excited states (the latter corresponding to optical transition energies from the ground sextet state, Fig. S35) obtained from CASSCF-NEVPT2-SOC-type *ab initio* calculations for Mn1 and Mn2 centers of **1** (Figure 1) when taking into account only the quartet excited states, shown together with the resulting wavelengths of optical transitions and the values of energies and wavelengths after the scaling procedure (energy shift for scaling set at –5000 cm^{-1}, see the Comment on theoretical calculations of optical transitions in Mn(II) complexes in **1** above).

**Mn1** complexes					**Mn2** complexes				
State	*E* / cm^{-1}	Wavelength / nm	Scaled *E* / cm^{-1}	Scaled wavelength / nm	State	*E* / cm^{-1}	Wavelength / nm	Scaled *E* / cm^{-1}	Scaled wavelength / nm
1	0	--	-5000	-	1	0	--	-5000	-
2	0	--	-5000	-	2	0	--	-5000	-
3	0	--	-5000	-	3	0	--	-5000	-
4	0	--	-5000	-	4	0	--	-5000	-
5	0	--	-5000	-	5	0	--	-5000	-
6	23471.2	426.05406	18471.2	541.38334	6	25367.6	394.20363	20367.6	490.97586
7	23471.2	426.05406	18471.2	541.38334	7	25367.6	394.20363	20367.6	490.97586
8	23487	425.76745	18487	540.92065	8	25367.8	394.20052	20367.8	490.97104
9	23487	425.76745	18487	540.92065	9	25367.8	394.20052	20367.8	490.97104
10	23506.1	425.42149	18506.1	540.36237	10	25817.2	387.33867	20817.2	480.372
11	23506.1	425.42149	18506.1	540.36237	11	25817.2	387.33867	20817.2	480.372
12	23522.6	425.12307	18522.6	539.88101	12	25819.2	387.30867	20819.2	480.32585
13	23522.6	425.12307	18522.6	539.88101	13	25819.2	387.30867	20819.2	480.32585
14	24009.6	416.50007	19009.6	526.05	14	25909.1	385.96478	20909.1	478.26066
15	24009.6	416.50007	19009.6	526.05	15	25909.1	385.96478	20909.1	478.26066
16	24011.8	416.46191	19011.8	525.98912	16	25911.6	385.92754	20911.6	478.20349
17	24011.8	416.46191	19011.8	525.98912	17	25911.6	385.92754	20911.6	478.20349
18	26791.4	373.2541	21791.4	458.89663	18	26882.7	371.98644	21882.7	456.982
19	26791.4	373.2541	21791.4	458.89663	19	26882.7	371.98644	21882.7	456.982
20	26798.1	373.16078	21798.1	458.75558	20	26885.8	371.94355	21885.8	456.91727
21	26798.1	373.16078	21798.1	458.75558	21	26885.8	371.94355	21885.8	456.91727
22	27124.1	368.67583	22124.1	451.99579	22	26990.2	370.50485	21990.2	454.74802
23	27124.1	368.67583	22124.1	451.99579	23	26990.2	370.50485	21990.2	454.74802
24	27142.3	368.42862	22142.3	451.62427	24	27001.7	370.34705	22001.7	454.51033
25	27142.3	368.42862	22142.3	451.62427	25	27001.7	370.34705	22001.7	454.51033
26	27225.4	367.30406	22225.4	449.93566	26	27030	369.9593	22030	453.92646
27	27225.4	367.30406	22225.4	449.93566	27	27030	369.9593	22030	453.92646
28	27236.7	367.15167	22236.7	449.70702	28	27040.8	369.81154	22040.8	453.70404
29	27236.7	367.15167	22236.7	449.70702	29	27040.8	369.81154	22040.8	453.70404
30	28111.9	355.72124	23111.9	432.67754	30	27326.4	365.94648	22326.4	447.90024
31	28111.9	355.72124	23111.9	432.67754	31	27326.4	365.94648	22326.4	447.90024
32	28113.9	355.69594	23113.9	432.6401	32	27326.7	365.94247	22326.7	447.89423
33	28113.9	355.69594	23113.9	432.6401	33	27326.7	365.94247	22326.7	447.89423
34	28119.7	355.62257	23119.7	432.53156	34	27430.1	364.56302	22430.1	445.82949
35	28119.7	355.62257	23119.7	432.53156	35	27430.1	364.56302	22430.1	445.82949
36	28121.6	355.59854	23121.6	432.49602	36	27430.5	364.5577	22430.5	445.82154

Table S20 (part 2/3) Energies (*E*) of ground and excited states (the latter corresponding to optical transition energies from the ground sextet state, Fig. S35) obtained from CASSCF-NEVPT2-SOC-type *ab initio* calculations for Mn1 and Mn2 centers of **1** (Figure 1) when taking into account only the quartet excited states, shown together with the resulting wavelengths of optical transitions and the values of energies and wavelengths after the scaling procedure (energy shift for scaling set at –5000 cm^{-1}, see the Comment on theoretical calculations of optical transitions in Mn(II) complexes in **1** above).

**Mn1** complexes					**Mn2** complexes				
State	*E* / cm^{-1}	Wavelength / nm	Scaled *E* / cm^{-1}	Scaled wavelength / nm	State	*E* / cm^{-1}	Wavelength / nm	Scaled *E* / cm^{-1}	Scaled wavelength / nm
37	28121.6	355.59854	23121.6	432.49602	37	27430.5	364.5577	22430.5	445.82154
38	28211.9	354.46035	23211.9	430.81351	38	27443.2	364.38899	22443.2	445.56926
39	28211.9	354.46035	23211.9	430.81351	39	27443.2	364.38899	22443.2	445.56926
40	28212.4	354.45407	23212.4	430.80423	40	27443.4	364.38634	22443.4	445.56529
41	28212.4	354.45407	23212.4	430.80423	41	27443.4	364.38634	22443.4	445.56529
42	31803.4	314.43179	26803.4	373.087	42	31140.2	321.12832	26140.2	382.55254
43	31803.4	314.43179	26803.4	373.087	43	31140.2	321.12832	26140.2	382.55254
44	31915.5	313.32738	26915.5	371.53313	44	31163.4	320.88925	26163.4	382.21332
45	31915.5	313.32738	26915.5	371.53313	45	31163.4	320.88925	26163.4	382.21332
46	32090.5	311.6187	27090.5	369.13309	46	31244.9	320.05223	26244.9	381.02641
47	32090.5	311.6187	27090.5	369.13309	47	31244.9	320.05223	26244.9	381.02641
48	32230	310.26993	27230	367.24201	48	31295.1	319.53884	26295.1	380.29899
49	32230	310.26993	27230	367.24201	49	31295.1	319.53884	26295.1	380.29899
50	32443.1	308.23195	27443.1	364.39032	50	31644.4	316.01168	26644.4	375.31339
51	32443.1	308.23195	27443.1	364.39032	51	31644.4	316.01168	26644.4	375.31339
52	32499.5	307.69704	27499.5	363.64298	52	31658.3	315.87293	26658.3	375.11769
53	32499.5	307.69704	27499.5	363.64298	53	31658.3	315.87293	26658.3	375.11769
54	33765.1	296.16379	28765.1	347.6435	54	32055.5	311.95895	27055.5	369.61062
55	33765.1	296.16379	28765.1	347.6435	55	32055.5	311.95895	27055.5	369.61062
56	33802.7	295.83436	28802.7	347.18967	56	32069.6	311.82179	27069.6	369.41809
57	33802.7	295.83436	28802.7	347.18967	57	32069.6	311.82179	27069.6	369.41809
58	33921.6	294.79742	28921.6	345.76234	58	32249.4	310.08329	27249.4	366.98056
59	33921.6	294.79742	28921.6	345.76234	59	32249.4	310.08329	27249.4	366.98056
60	34036.9	293.79879	29036.9	344.38938	60	32318.4	309.42126	27318.4	366.05365
61	34036.9	293.79879	29036.9	344.38938	61	32318.4	309.42126	27318.4	366.05365
62	34099.7	293.25771	29099.7	343.64615	62	32362.3	309.00152	27362.3	365.46635
63	34099.7	293.25771	29099.7	343.64615	63	32362.3	309.00152	27362.3	365.46635
64	34163.4	292.71091	29163.4	342.89555	64	32544.9	307.26781	27544.9	363.04361
65	34163.4	292.71091	29163.4	342.89555	65	32544.9	307.26781	27544.9	363.04361
66	35091.2	284.97173	30091.2	332.32307	66	32855.2	304.36582	27855.2	358.9994
67	35091.2	284.97173	30091.2	332.32307	67	32855.2	304.36582	27855.2	358.9994
68	35244.3	283.73382	30244.3	330.64081	68	32918.3	303.78239	27918.3	358.188
69	35244.3	283.73382	30244.3	330.64081	69	32918.3	303.78239	27918.3	358.188
70	35399.1	282.49306	30399.1	328.95711	70	32922.4	303.74456	27922.4	358.1354
71	35399.1	282.49306	30399.1	328.95711	71	32922.4	303.74456	27922.4	358.1354
72	35485.4	281.80604	30485.4	328.02587	72	32971.4	303.29316	27971.4	357.50803

Table S20 (part 3/3) Energies (*E*) of ground and excited states (the latter corresponding to optical transition energies from the ground sextet state, Fig. S35) obtained from CASSCF-NEVPT2-SOC-type *ab initio* calculations for Mn1 and Mn2 centers of **1** (Figure 1) when taking into account only the quartet excited states, shown together with the resulting wavelengths of optical transitions and the values of energies and wavelengths after the scaling procedure (energy shift for scaling set at –5000 cm^{-1}, see the Comment on theoretical calculations of optical transitions in Mn(II) complexes in **1** above).

**Mn1** complexes					**Mn2** complexes				
State	*E* / cm^{-1}	Wavelength / nm	Scaled *E* / cm^{-1}	Scaled wavelength / nm	State	*E* / cm^{-1}	Wavelength / nm	Scaled *E* / cm^{-1}	Scaled wavelength / nm
73	35485.4	281.80604	30485.4	328.02587	73	32971.4	303.29316	27971.4	357.50803
74	45434	220.09948	40434	247.31661	74	43836.1	228.12248	38836.1	257.49238
75	45434	220.09948	40434	247.31661	75	43836.1	228.12248	38836.1	257.49238
76	45437.4	220.08302	40437.4	247.29582	76	43840	228.10219	38840	257.46653
77	45437.4	220.08302	40437.4	247.29582	77	43840	228.10219	38840	257.46653
78	45610.7	219.2468	40610.7	246.24052	78	43921.1	227.681	38921.1	256.93005
79	45610.7	219.2468	40610.7	246.24052	79	43921.1	227.681	38921.1	256.93005
80	45621.3	219.19586	40621.3	246.17627	80	43930	227.63487	38930	256.87131
81	45621.3	219.19586	40621.3	246.17627	81	43930	227.63487	38930	256.87131
82	46047.6	217.16658	41047.6	243.6196	82	43992.9	227.30941	38992.9	256.45694
83	46047.6	217.16658	41047.6	243.6196	83	43992.9	227.30941	38992.9	256.45694
84	46052.5	217.14348	41052.5	243.59052	84	43999.2	227.27686	38999.2	256.41552
85	46052.5	217.14348	41052.5	243.59052	85	43999.2	227.27686	38999.2	256.41552
86	46243.3	216.24754	41243.3	242.46362	86	44263.8	225.91824	39263.8	254.68752
87	46243.3	216.24754	41243.3	242.46362	87	44263.8	225.91824	39263.8	254.68752
88	46247.8	216.2265	41247.8	242.43717	88	44265.1	225.91161	39265.1	254.67909
89	46247.8	216.2265	41247.8	242.43717	89	44265.1	225.91161	39265.1	254.67909
90	47765.8	209.35481	42765.8	233.83171	90	44470.1	224.87019	39470.1	253.35634
91	47765.8	209.35481	42765.8	233.83171	91	44470.1	224.87019	39470.1	253.35634
92	47787	209.26193	42787	233.71585	92	44488.7	224.77618	39488.7	253.237
93	47787	209.26193	42787	233.71585	93	44488.7	224.77618	39488.7	253.237
94	48020.1	208.24613	43020.1	232.44948	94	44536.4	224.53544	39536.4	252.93148
95	48020.1	208.24613	43020.1	232.44948	95	44536.4	224.53544	39536.4	252.93148
96	48065.4	208.04987	43065.4	232.20497	96	44556.9	224.43213	39556.9	252.8004
97	48065.4	208.04987	43065.4	232.20497	97	44556.9	224.43213	39556.9	252.8004
98	48171.2	207.59292	43171.2	231.63591	98	44859.3	222.91922	39859.3	250.88248
99	48171.2	207.59292	43171.2	231.63591	99	44859.3	222.91922	39859.3	250.88248
100	48206	207.44306	43206	231.44934	100	44861.5	222.90828	39861.5	250.86863
101	48206	207.44306	43206	231.44934	101	44861.5	222.90828	39861.5	250.86863

Table S21 (part 1/7) Energies (*E*) of ground and excited states (the latter corresponding to optical transition energies from the ground sextet state, Fig. S35) obtained from CASSCF-NEVPT2-SOC-type *ab initio* calculations for Mn1 and Mn2 centers of **1** (Figure 1) when taking into account the quartet and doublet excited states, shown together with the resulting wavelengths of optical transitions and the values of energies and wavelengths after the scaling procedure (energy shift for scaling set at –5000 cm^{-1}, see the Comment on theoretical calculations of optical transitions in Mn(II) complexes in **1** above).

**Mn1** complexes					**Mn2** complexes				
State	*E* / cm^{-1}	Wavelength / nm	Scaled *E* / cm^{-1}	Scaled wavelength / nm	State	*E* / cm^{-1}	Wavelength / nm	Scaled *E* / cm^{-1}	Scaled wavelength / nm
1	0	-	-5000	-	1	0	--	-5000	-
2	0	-	-5000	-	2	0	--	-5000	-
3	0	-	-5000	-	3	0	--	-5000	-
4	0	-	-5000	-	4	0	--	-5000	-
5	0	-	-5000	-	5	0	--	-5000	-
6	23331	428.61429	18331	545.52398	6	23621.3	423.34673	18621.3	537.01943
7	23331	428.61429	18331	545.52398	7	23621.3	423.34673	18621.3	537.01943
8	23345.2	428.35358	18345.2	545.10172	8	23629.2	423.20519	18629.2	536.7917
9	23345.2	428.35358	18345.2	545.10172	9	23629.2	423.20519	18629.2	536.7917
10	23364.2	428.00524	18364.2	544.53774	10	24053.1	415.74683	19053.1	524.84897
11	23364.2	428.00524	18364.2	544.53774	11	24053.1	415.74683	19053.1	524.84897
12	23379.7	427.72148	18379.7	544.07852	12	24060.5	415.61896	19060.5	524.64521
13	23379.7	427.72148	18379.7	544.07852	13	24060.5	415.61896	19060.5	524.64521
14	23875	418.84817	18875	529.80132	14	24139.9	414.25192	19139.9	522.46877
15	23875	418.84817	18875	529.80132	15	24139.9	414.25192	19139.9	522.46877
16	23877.2	418.80958	18877.2	529.73958	16	24148.3	414.10783	19148.3	522.23957
17	23877.2	418.80958	18877.2	529.73958	17	24148.3	414.10783	19148.3	522.23957
18	26694.9	374.60339	21694.9	460.93782	18	25008.4	399.86565	20008.4	499.79009
19	26694.9	374.60339	21694.9	460.93782	19	25008.4	399.86565	20008.4	499.79009
20	26702.2	374.50098	21702.2	460.78278	20	25011.6	399.81449	20011.6	499.71017
21	26702.2	374.50098	21702.2	460.78278	21	25011.6	399.81449	20011.6	499.71017
22	27037.1	369.86215	22037.1	453.78022	22	25123.9	398.02738	20123.9	496.92157
23	27037.1	369.86215	22037.1	453.78022	23	25123.9	398.02738	20123.9	496.92157
24	27054	369.63111	22054	453.43248	24	25134.8	397.85477	20134.8	496.65256
25	27054	369.63111	22054	453.43248	25	25134.8	397.85477	20134.8	496.65256
26	27139.9	368.4612	22139.9	451.67322	26	25164.9	397.37889	20164.9	495.91121
27	27139.9	368.4612	22139.9	451.67322	27	25164.9	397.37889	20164.9	495.91121
28	27149.4	368.33227	22149.4	451.4795	28	25168.8	397.31731	20168.8	495.81532
29	27149.4	368.33227	22149.4	451.4795	29	25168.8	397.31731	20168.8	495.81532
30	28046	356.55708	23046	433.91478	30	25491.6	392.28609	20491.6	488.00484
31	28046	356.55708	23046	433.91478	31	25491.6	392.28609	20491.6	488.00484
32	28048.2	356.52912	23048.2	433.87336	32	25492.8	392.26762	20492.8	487.97626
33	28048.2	356.52912	23048.2	433.87336	33	25492.8	392.26762	20492.8	487.97626
34	28053.8	356.45795	23053.8	433.76797	34	25569.1	391.09707	20569.1	486.16614
35	28053.8	356.45795	23053.8	433.76797	35	25569.1	391.09707	20569.1	486.16614
36	28055.9	356.43127	23055.9	433.72846	36	25570.8	391.07107	20570.8	486.12596

Table S21 (part 2/7) Energies (*E*) of ground and excited states (the latter corresponding to optical transition energies from the ground sextet state, Fig. S35) obtained from CASSCF-NEVPT2-SOC-type *ab initio* calculations for Mn1 and Mn2 centers of **1** (Figure 1) when taking into account the quartet and doublet excited states, shown together with the resulting wavelengths of optical transitions and the values of energies and wavelengths after the scaling procedure (energy shift for scaling set at –5000 cm^{-1}, see the Comment on theoretical calculations of optical transitions in Mn(II) complexes in **1** above).

**Mn1** complexes					**Mn2** complexes				
State	*E* / cm^{-1}	Wavelength / nm	Scaled *E* / cm^{-1}	Scaled wavelength / nm	State	*E* / cm^{-1}	Wavelength / nm	Scaled *E* / cm^{-1}	Scaled wavelength / nm
37	28055.9	356.43127	23055.9	433.72846	37	25570.8	391.07107	20570.8	486.12596
38	28143.3	355.32436	23143.3	432.0905	38	25581.3	390.91055	20581.3	485.87796
39	28143.3	355.32436	23143.3	432.0905	39	25581.3	390.91055	20581.3	485.87796
40	28143.5	355.32183	23143.5	432.08676	40	25584.1	390.86777	20584.1	485.81186
41	28143.5	355.32183	23143.5	432.08676	41	25584.1	390.86777	20584.1	485.81186
Due to the computational limitations further states were not calculated for Mn1 complexes in this approach.					42	29802.5	335.54232	24802.5	403.18516
					43	29802.5	335.54232	24802.5	403.18516
					44	29873	334.75044	24873	402.04238
					45	29873	334.75044	24873	402.04238
					46	30007.5	333.25002	25007.5	399.88004
					47	30007.5	333.25002	25007.5	399.88004
					48	30079.8	332.44902	25079.8	398.72726
					49	30079.8	332.44902	25079.8	398.72726
					50	30182.2	331.32111	25182.2	397.10589
					51	30182.2	331.32111	25182.2	397.10589
					52	30249.7	330.58179	25249.7	396.04431
					53	30249.7	330.58179	25249.7	396.04431
					54	30377.9	329.18668	25377.9	394.04364
					55	30377.9	329.18668	25377.9	394.04364
					56	30436.8	328.54965	25436.8	393.13121
					57	30436.8	328.54965	25436.8	393.13121
					58	30599.6	326.80166	25599.6	390.6311
					59	30599.6	326.80166	25599.6	390.6311
					60	30647.7	326.28876	25647.7	389.89851
					61	30647.7	326.28876	25647.7	389.89851
					62	30797.2	324.70484	25797.2	387.63897
					63	30797.2	324.70484	25797.2	387.63897
					64	30940.8	323.19785	25940.8	385.49312
					65	30940.8	323.19785	25940.8	385.49312
					66	31019.1	322.38202	26019.1	384.33305
					67	31019.1	322.38202	26019.1	384.33305
					68	31105.2	321.48965	26105.2	383.06544
					69	31105.2	321.48965	26105.2	383.06544
					70	31155.2	320.97371	26155.2	382.33315
					71	31155.2	320.97371	26155.2	382.33315
					72	31260.6	319.89149	26260.6	380.79861

Table S21 (part 3/7) Energies (*E*) of ground and excited states (the latter corresponding to optical transition energies from the ground sextet state, Fig. S35) obtained from CASSCF-NEVPT2-SOC-type *ab initio* calculations for Mn1 and Mn2 centers of **1** (Figure 1) when taking into account the quartet and doublet excited states, shown together with the resulting wavelengths of optical transitions and the values of energies and wavelengths after the scaling procedure (energy shift for scaling set at –5000 cm^{-1}, see the Comment on theoretical calculations of optical transitions in Mn(II) complexes in **1** above).

**Mn1** complexes					**Mn2** complexes				
State	*E* / cm^{-1}	Wavelength / nm	Scaled *E* / cm^{-1}	Scaled wavelength / nm	State	*E* / cm^{-1}	Wavelength / nm	Scaled *E* / cm^{-1}	Scaled wavelength / nm
					73	31260.6	319.89149	26260.6	380.79861
					74	34498	289.87188	29498	339.00603
					75	34498	289.87188	29498	339.00603
					76	34726.7	287.96286	29726.7	336.39792
					77	34726.7	287.96286	29726.7	336.39792
					78	35121.5	284.72588	30121.5	331.98878
					79	35121.5	284.72588	30121.5	331.98878
					80	35601	280.89099	30601	326.78671
					81	35601	280.89099	30601	326.78671
					82	35703.7	280.08302	30703.7	325.69365
					83	35703.7	280.08302	30703.7	325.69365
					84	36039.2	277.47564	31039.2	322.17325
					85	36039.2	277.47564	31039.2	322.17325
					86	36104.7	276.97225	31104.7	321.49482
					87	36104.7	276.97225	31104.7	321.49482
					88	36206.2	276.19579	31206.2	320.44914
					89	36206.2	276.19579	31206.2	320.44914
					90	36386	274.83098	31386	318.61339
					91	36386	274.83098	31386	318.61339
					92	36498.9	273.98086	31498.9	317.4714
					93	36498.9	273.98086	31498.9	317.4714
					94	36618	273.08974	31618	316.27554
					95	36618	273.08974	31618	316.27554
					96	36739.8	272.18439	31739.8	315.06185
					97	36739.8	272.18439	31739.8	315.06185
					98	36977.6	270.43399	31977.6	312.7189
					99	36977.6	270.43399	31977.6	312.7189
					100	38554.4	259.37377	33554.4	298.02351
					101	38554.4	259.37377	33554.4	298.02351
					102	38647.6	258.74828	33647.6	297.19802
					103	38647.6	258.74828	33647.6	297.19802
					104	39307.6	254.40373	34307.6	291.4806
					105	39307.6	254.40373	34307.6	291.4806
					106	39925.2	250.46838	34925.2	286.32621
					107	39925.2	250.46838	34925.2	286.32621
					108	40255.8	248.41141	35255.8	283.64127

Table S21 (part 4/7) Energies (*E*) of ground and excited states (the latter corresponding to optical transition energies from the ground sextet state, Fig. S35) obtained from CASSCF-NEVPT2-SOC-type *ab initio* calculations for Mn1 and Mn2 centers of **1** (Figure 1) when taking into account the quartet and doublet excited states, shown together with the resulting wavelengths of optical transitions and the values of energies and wavelengths after the scaling procedure (energy shift for scaling set at –5000 cm^{-1}, see the Comment on theoretical calculations of optical transitions in Mn(II) complexes in **1** above).

**Mn1** complexes					**Mn2** complexes				
State	*E* / cm^{-1}	Wavelength / nm	Scaled *E* / cm^{-1}	Scaled wavelength / nm	State	*E* / cm^{-1}	Wavelength / nm	Scaled *E* / cm^{-1}	Scaled wavelength / nm
					109	40255.8	248.41141	35255.8	283.64127
					110	41526.1	240.8124	36526.1	273.77683
					111	41526.1	240.8124	36526.1	273.77683
					112	41540.7	240.72777	36540.7	273.66744
					113	41540.7	240.72777	36540.7	273.66744
					114	41609.2	240.33147	36609.2	273.15538
					115	41609.2	240.33147	36609.2	273.15538
					116	41640.2	240.15254	36640.2	272.92427
					117	41640.2	240.15254	36640.2	272.92427
					118	41676.9	239.94107	36676.9	272.65118
					119	41676.9	239.94107	36676.9	272.65118
					120	41698.8	239.81505	36698.8	272.48847
					121	41698.8	239.81505	36698.8	272.48847
					122	41732.9	239.6191	36732.9	272.23552
					123	41732.9	239.6191	36732.9	272.23552
					124	41900.8	238.65893	36900.8	270.99683
					125	41900.8	238.65893	36900.8	270.99683
					126	41951	238.37334	36951	270.62867
					127	41951	238.37334	36951	270.62867
					128	41986.5	238.17179	36986.5	270.36892
					129	41986.5	238.17179	36986.5	270.36892
					130	42074.5	237.67365	37074.5	269.72717
					131	42074.5	237.67365	37074.5	269.72717
					132	42215.3	236.88094	37215.3	268.70669
					133	42215.3	236.88094	37215.3	268.70669
					134	42235.6	236.76709	37235.6	268.5602
					135	42235.6	236.76709	37235.6	268.5602
					136	42280.6	236.51509	37280.6	268.23603
					137	42280.6	236.51509	37280.6	268.23603
					138	42303.1	236.3893	37303.1	268.07424
					139	42303.1	236.3893	37303.1	268.07424
					140	42445.3	235.59735	37445.3	267.05621
					141	42445.3	235.59735	37445.3	267.05621
					142	42621	234.62612	37621	265.80899
					143	42621	234.62612	37621	265.80899
					144	42623.6	234.61181	37623.6	265.79062

Table S21 (part 5/7) Energies (*E*) of ground and excited states (the latter corresponding to optical transition energies from the ground sextet state, Fig. S35) obtained from CASSCF-NEVPT2-SOC-type *ab initio* calculations for Mn1 and Mn2 centers of **1** (Figure 1) when taking into account the quartet and doublet excited states, shown together with the resulting wavelengths of optical transitions and the values of energies and wavelengths after the scaling procedure (energy shift for scaling set at –5000 cm^{-1}, see the Comment on theoretical calculations of optical transitions in Mn(II) complexes in **1** above).

**Mn1** complexes					**Mn2** complexes				
State	*E* / cm^{-1}	Wavelength / nm	Scaled *E* / cm^{-1}	Scaled wavelength / nm	State	*E* / cm^{-1}	Wavelength / nm	Scaled *E* / cm^{-1}	Scaled wavelength / nm
					145	42623.6	234.61181	37623.6	265.79062
					146	42857.2	233.33302	37857.2	264.15054
					147	42857.2	233.33302	37857.2	264.15054
					148	43126.3	231.87707	38126.3	262.28614
					149	43126.3	231.87707	38126.3	262.28614
					150	43163	231.67991	38163	262.03391
					151	43163	231.67991	38163	262.03391
					152	44308.2	225.69186	39308.2	254.39985
					153	44308.2	225.69186	39308.2	254.39985
					154	44481.1	224.81458	39481.1	253.28575
					155	44481.1	224.81458	39481.1	253.28575
					156	44617.6	224.1268	39617.6	252.41307
					157	44617.6	224.1268	39617.6	252.41307
					158	44797.9	223.22475	39797.9	251.26954
					159	44797.9	223.22475	39797.9	251.26954
					160	45055.7	221.9475	40055.7	249.65236
					161	45055.7	221.9475	40055.7	249.65236
					162	45241.5	221.036	40241.5	248.49968
					163	45241.5	221.036	40241.5	248.49968
					164	45299.1	220.75494	40299.1	248.1445
					165	45299.1	220.75494	40299.1	248.1445
					166	45448.2	220.03072	40448.2	247.22979
					167	45448.2	220.03072	40448.2	247.22979
					168	45656.5	219.02686	40656.5	245.96313
					169	45656.5	219.02686	40656.5	245.96313
					170	45844.7	218.12772	40844.7	244.82981
					171	45844.7	218.12772	40844.7	244.82981
					172	45932	217.71314	40932	244.30763
					173	45932	217.71314	40932	244.30763
					174	46017.6	217.30816	41017.6	243.79778
					175	46017.6	217.30816	41017.6	243.79778
					176	46121.7	216.81768	41121.7	243.18061
					177	46121.7	216.81768	41121.7	243.18061
					178	46201.1	216.44506	41201.1	242.71197
					179	46201.1	216.44506	41201.1	242.71197
					180	46550.8	214.81908	41550.8	240.66925

Table S21 (part 6/7) Energies (*E*) of ground and excited states (the latter corresponding to optical transition energies from the ground sextet state, Fig. S35) obtained from CASSCF-NEVPT2-SOC-type *ab initio* calculations for Mn1 and Mn2 centers of **1** (Figure 1) when taking into account the quartet and doublet excited states, shown together with the resulting wavelengths of optical transitions and the values of energies and wavelengths after the scaling procedure (energy shift for scaling set at –5000 cm^{-1}, see the Comment on theoretical calculations of optical transitions in Mn(II) complexes in **1** above).

**Mn1** complexes					**Mn2** complexes				
State	*E* / cm^{-1}	Wavelength / nm	Scaled *E* / cm^{-1}	Scaled wavelength / nm	State	*E* / cm^{-1}	Wavelength / nm	Scaled *E* / cm^{-1}	Scaled wavelength / nm
					181	46550.8	214.81908	41550.8	240.66925
					182	46627	214.46801	41627	240.2287
					183	46627	214.46801	41627	240.2287
					184	46694.3	214.1589	41694.3	239.84094
					185	46694.3	214.1589	41694.3	239.84094
					186	46798.3	213.68298	41798.3	239.24418
					187	46798.3	213.68298	41798.3	239.24418
					188	47367.4	211.11566	42367.4	236.03053
					189	47367.4	211.11566	42367.4	236.03053
					190	47637.3	209.91954	42637.3	234.53643
					191	47637.3	209.91954	42637.3	234.53643
					192	47694.3	209.66866	42694.3	234.2233
					193	47694.3	209.66866	42694.3	234.2233
					194	48049.7	208.11784	43049.7	232.28966
					195	48049.7	208.11784	43049.7	232.28966
					196	48176.8	207.56879	43176.8	231.60586
					197	48176.8	207.56879	43176.8	231.60586
					198	48460	206.35576	43460	230.09664
					199	48460	206.35576	43460	230.09664
					200	48500.2	206.18472	43500.2	229.884
					201	48500.2	206.18472	43500.2	229.884
					202	48634.9	205.61366	43634.9	229.17435
					203	48634.9	205.61366	43634.9	229.17435
					204	48812	204.86766	43812	228.24797
					205	48812	204.86766	43812	228.24797
					206	52422.4	190.75815	47422.4	210.87081
					207	52422.4	190.75815	47422.4	210.87081
					208	58858.1	169.90015	53858.1	185.67309
					209	58858.1	169.90015	53858.1	185.67309
					210	58925.9	169.70466	53925.9	185.43965
					211	58925.9	169.70466	53925.9	185.43965
					212	59174.5	168.99171	54174.5	184.58869
					213	59174.5	168.99171	54174.5	184.58869
					214	59226.9	168.8422	54226.9	184.41032
					215	59226.9	168.8422	54226.9	184.41032
					216	59379.1	168.40942	54379.1	183.89418

Table S21 (part 7/7) Energies (*E*) of ground and excited states (the latter corresponding to optical transition energies from the ground sextet state, Fig. S35) obtained from CASSCF-NEVPT2-SOC-type *ab initio* calculations for Mn1 and Mn2 centers of **1** (Figure 1) when taking into account the quartet and doublet excited states, shown together with the resulting wavelengths of optical transitions and the values of energies and wavelengths after the scaling procedure (energy shift for scaling set at –5000 cm^{-1}, see the Comment on theoretical calculations of optical transitions in Mn(II) complexes in **1** above).

**Mn1** complexes					**Mn2** complexes				
State	E / cm^{-1}	Wavelength / nm	Scaled E / cm^{-1}	Scaled wavelength / nm	State	E / cm^{-1}	Wavelength / nm	Scaled E / cm^{-1}	Scaled wavelength / nm
					217	59379.1	168.40942	54379.1	183.89418
					218	64710.2	154.53514	59710.2	167.47557
					219	64710.2	154.53514	59710.2	167.47557
					220	64796.4	154.32956	59796.4	167.23415
					221	64796.4	154.32956	59796.4	167.23415
					222	64819.2	154.27528	59819.2	167.17041
					223	64819.2	154.27528	59819.2	167.17041
					224	64838.1	154.23031	59838.1	167.11761
					225	64838.1	154.23031	59838.1	167.11761
					226	64944.7	153.97715	59944.7	166.82042
					227	64944.7	153.97715	59944.7	166.82042
					228	65059.7	153.70498	60059.7	166.501
					229	65059.7	153.70498	60059.7	166.501
					230	65078.4	153.66082	60078.4	166.44917
					231	65078.4	153.66082	60078.4	166.44917
					232	65106.8	153.59379	60106.8	166.37053
					233	65106.8	153.59379	60106.8	166.37053
					234	65133.6	153.53059	60133.6	166.29638
					235	65133.6	153.53059	60133.6	166.29638
					236	77684.9	128.72514	72684.9	137.58016
					237	77684.9	128.72514	72684.9	137.58016
					238	77756	128.60744	72756	137.44571
					239	77756	128.60744	72756	137.44571
					240	77810.7	128.51703	72810.7	137.34245
					241	77810.7	128.51703	72810.7	137.34245
					242	84761.4	117.97823	79761.4	125.37393
					243	84761.4	117.97823	79761.4	125.37393
					244	84856.3	117.84629	79856.3	125.22494
					245	84856.3	117.84629	79856.3	125.22494
					246	84910.9	117.77051	79910.9	125.13937
					247	84910.9	117.77051	79910.9	125.13937
					248	84958.1	117.70508	79958.1	125.0655
					249	84958.1	117.70508	79958.1	125.0655
					250	85025	117.61247	80025	124.96095
					251	85025	117.61247	80025	124.96095

Table S22 (part 1/2) Energies (*E*) of ground and excited states obtained from CASSCF-NEVPT2-SOC-type *ab initio* calculations for Mn2 centers of **1** in the optimized geometry of the lowest-lying excited quartet state (obtained, in the previous step, within the CASSCF-NEVPT2 theory of level, Table S18), with taking into account only the quartet excited states, shown together with the resulting wavelengths of optical transitions representing the hypothetical luminescent transitions for these complexes. The related optimized geometry of the complex together with the used active space is presented in Fig. S34.

State	Energy / cm^{-1}	Wavelength / nm	State	Energy / cm^{-1}	Wavelength / nm
1	0	-	37	27494.7	363.7
2	0	-	38	27495	363.7
3	0	-	39	27495	363.7
4	0	-	40	27496.3	363.7
5	0	-	41	27496.3	363.7
6	24210.6	413	42	30528.4	327.6
7	24210.6	413	43	30528.4	327.6
8	24211.5	413	44	30638.7	326.4
9	24211.5	413	45	30638.7	326.4
10	26244.8	381	46	30777	324.9
11	26244.8	381	47	30777	324.9
12	26250	381	48	30977.2	322.8
13	26250	381	49	30977.2	322.8
14	26259.5	380.8	50	31754.6	314.9
15	26259.5	380.8	51	31754.6	314.9
16	26264.9	380.7	52	31839.2	314.1
17	26264.9	380.7	53	31839.2	314.1
18	26832.9	372.7	54	32335.2	309.3
19	26832.9	372.7	55	32335.2	309.3
20	26837.8	372.6	56	32434.9	308.3
21	26837.8	372.6	57	32434.9	308.3
22	26860.6	372.3	58	32470.9	308
23	26860.6	372.3	59	32470.9	308
24	26884	372	60	32599.6	306.8
25	26884	372	61	32599.6	306.8
26	26905.8	371.7	62	32664.4	306.1
27	26905.8	371.7	63	32664.4	306.1
28	26928.1	371.4	64	32887.8	304.1
29	26928.1	371.4	65	32887.8	304.1
30	27030.9	369.9	66	32990.4	303.1
31	27030.9	369.9	67	32990.4	303.1
32	27033.6	369.9	68	33096.7	302.1
33	27033.6	369.9	69	33096.7	302.1
34	27493.6	363.7	70	33136.7	301.8
35	27493.6	363.7	71	33136.7	301.8
36	27494.7	363.7	72	33170	301.5

Table S22 (part 2/2) Energies (*E*) of ground and excited states obtained from CASSCF-NEVPT2-SOC-type *ab initio* calculations for Mn2 centers of **1** in the optimized geometry of the lowest-lying excited quartet state (obtained, in the previous step, within the CASSCF-NEVPT2 theory of level, Table S18), with taking into account only the quartet excited states, shown together with the resulting wavelengths of optical transitions representing the hypothetical luminescent transitions for these complexes. The related optimized geometry of the complex together with the used active space is presented in Fig. S34.

State	Energy / cm^{-1}	Wavelength / nm	State	Energy / cm^{-1}	Wavelength / nm
72	33170	301.5	87	44343.8	225.5
73	33170	301.5	88	44361.5	225.4
74	44172.6	226.4	89	44361.5	225.4
75	44172.6	226.4	90	44373.4	225.4
76	44173.8	226.4	91	44373.4	225.4
77	44173.8	226.4	92	44390.8	225.3
78	44290.2	225.8	93	44390.8	225.3
79	44290.2	225.8	94	44403.3	225.2
80	44300.3	225.7	95	44403.3	225.2
81	44300.3	225.7	96	44438.4	225
82	44317.4	225.6	97	44438.4	225
83	44317.4	225.6	98	45768.8	218.5
84	44328.2	225.6	99	45768.8	218.5
85	44328.2	225.6	100	45770.8	218.5
86	44343.8	225.5	101	45770.8	218.5

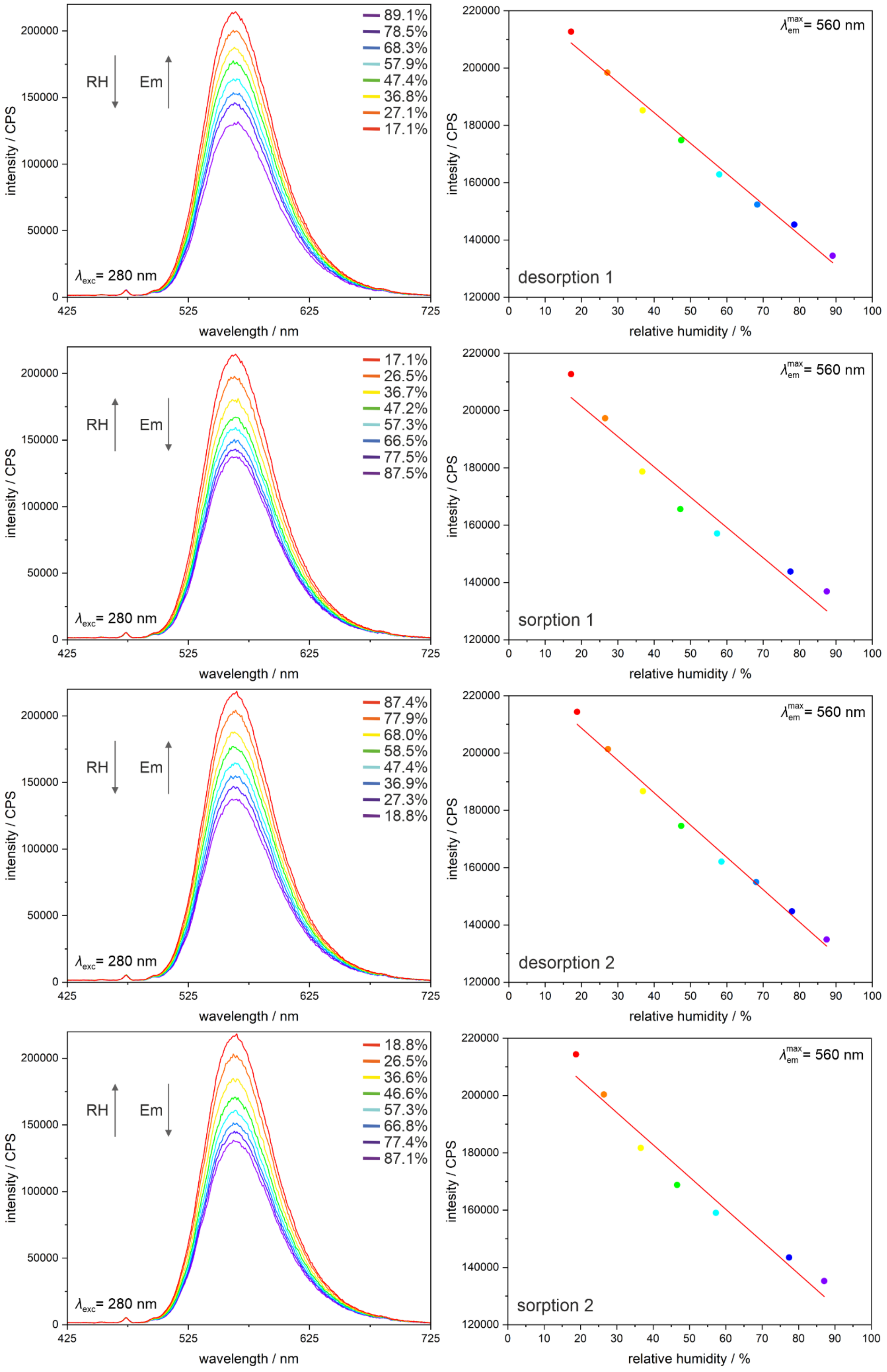

Fig. S36 Humidity-dependent emission (Em) spectra of **1** in the 17–90% relative humidity (RH) range at room temperature (left panel) and the related RH-dependences of the intensity of an emission maximum (right panel) with linear fits, measured for two desorption–sorption cycles. The best-fit parameters are gathered in Table S23.

Table S23 Best-fit parameters for the relative humidity-dependent intensity of the emission maximum of **1** (λ_{exc} = 280 nm, λ^{max} = 560 nm, see Fig. S17 for the experimental data and best-fit curves) in the 17–90% relative humidity range at room temperature for two consecutive cycles of desorption and sorption. The parameters are the results of fitting to the linear function: y = *a*x + *b*, where y stands for the intensity at the emission maximum, while x corresponds to the relative humidity value from the 17–90% range.

Cycle (Fig. S17)	*a*	*b*	R^2
desorption 1	-1065(40)	227000(2300)	0.9914
sorption 1	-1054(98)	222700(5600)	0.9513
desorption 2	-1128(47)	231300(2700)	0.9896
sorption 2	-1124(99)	227800(5000)	0.9645

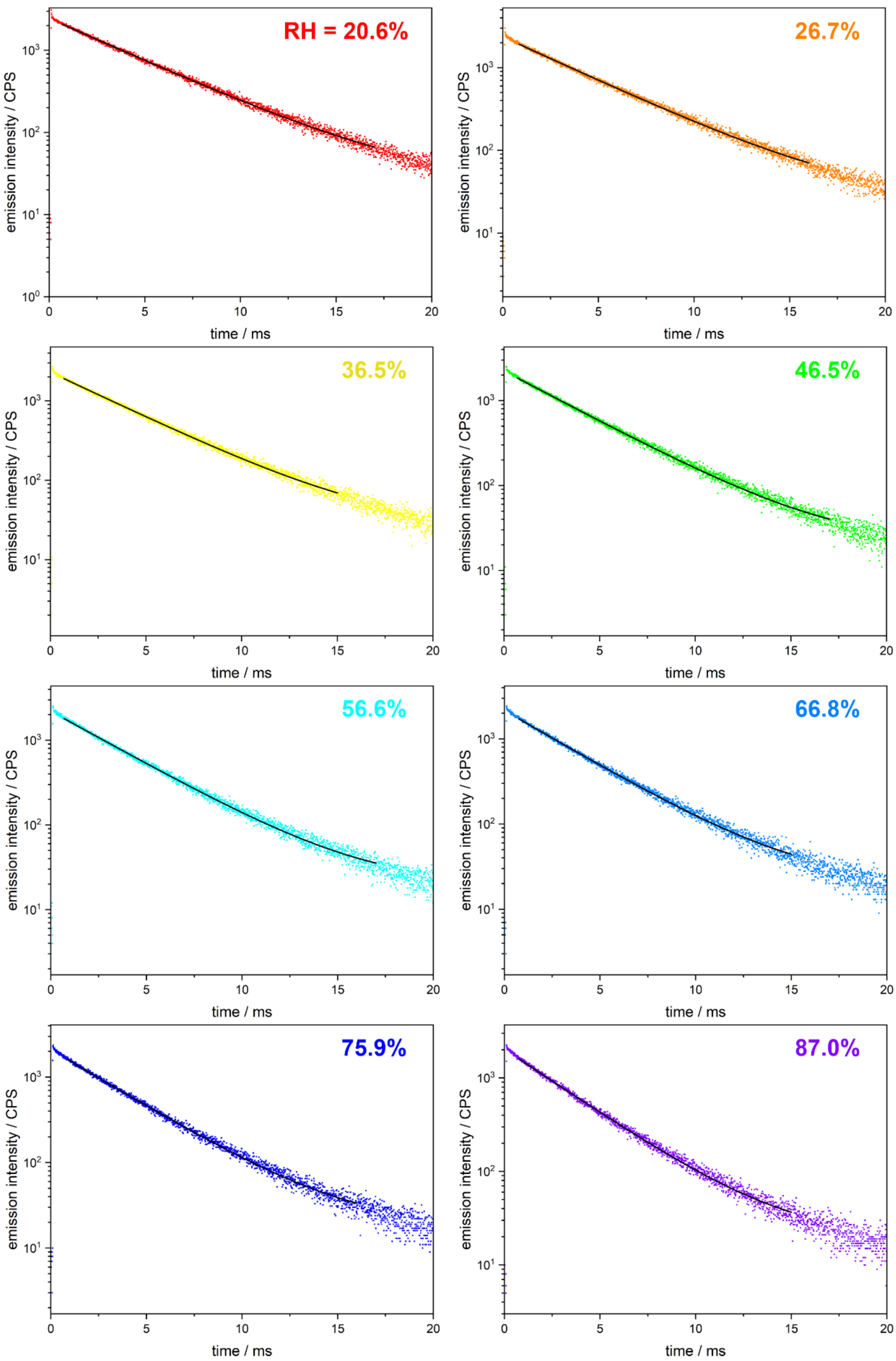

Fig. S37 Humidity-dependent emission decay profiles of **1** (λ_{exc} = 280 nm and λ_{em} = 560 nm) in the 20–87% relative humidity (RH) range at room temperature for the sorption cycle. The mono-exponential fitting was applied for the measurement at each indicated relative humidity. The best-fit parameters are gathered in Table S24.

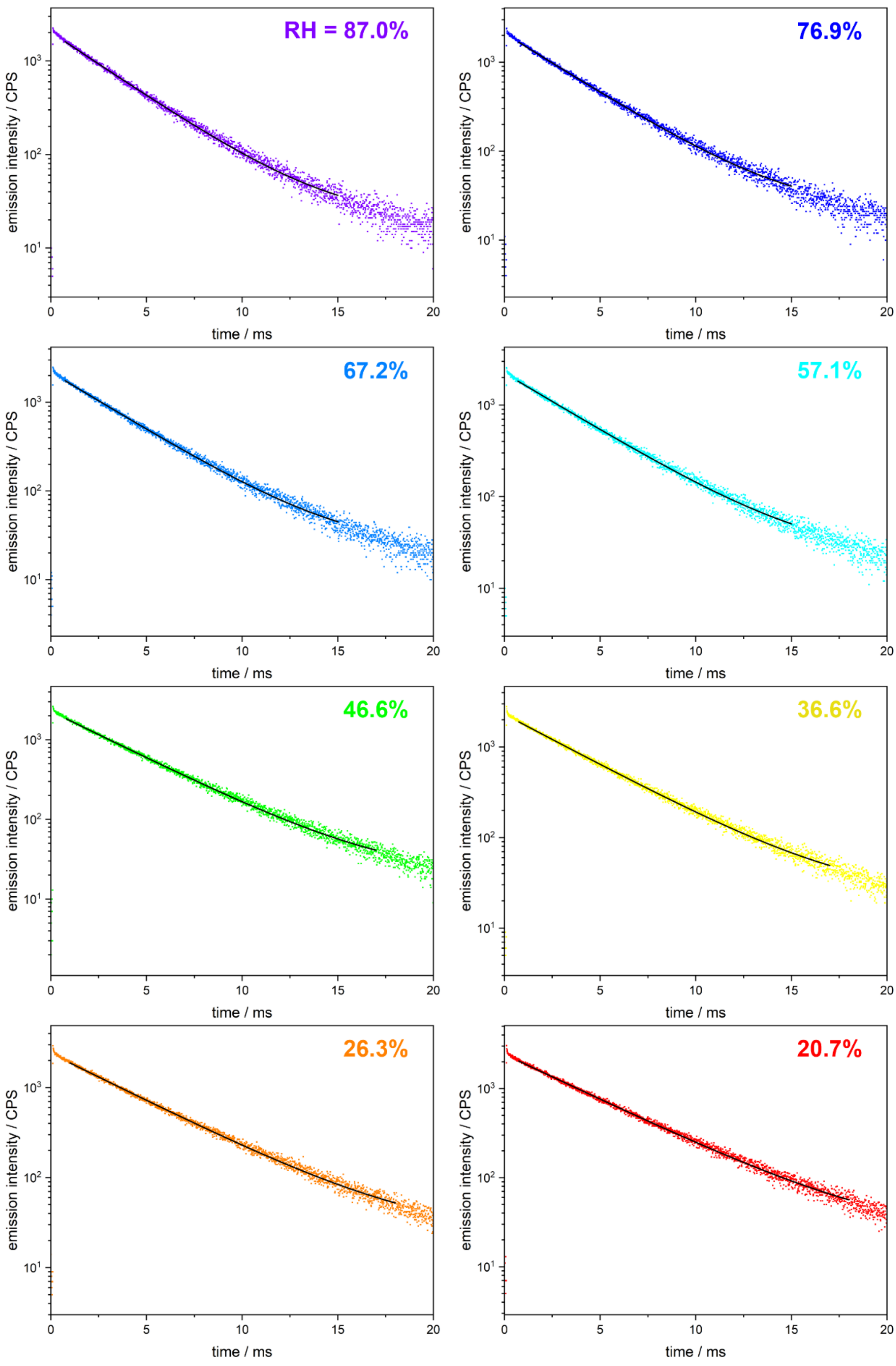

Fig. S38 Humidity-dependent emission decay profiles of **1** (λ_{exc} = 280 nm and λ_{em} = 560 nm) in the 20–87% relative humidity (RH) range at room temperature for the desorption cycle. The mono-exponential fitting was applied for the measurement at each indicated relative humidity. The best-fit parameters are gathered in Table S25.

Table S24 Best-fit parameters to the mono-exponential decay function for the humidity-dependent emission decay profiles of **1** (λ_{exc} = 280 nm and λ_{em} = 560 nm), which were gathered in the 20–87% relative humidity range at room temperature for the sorption cycle (Fig. S37).

Relative humidity / %	τ_1 / ms	χ^2
20.6	4.193(12)	1.0418
26.7	4.070(13)	1.0750
36.5	3.792(12)	1.0177
46.5	3.633(9)	1.0056
56.5	3.455(9)	1.0443
66.8	3.324(10)	1.0802
75.9	3.260(9)	1.0771
87.0	3.148(9)	1.0952

Table S25 Best-fit parameters to the mono-exponential decay function for the humidity-dependent emission decay profiles of **1** (λ_{exc} = 280 nm and λ_{em} = 560 nm), which were gathered in the 20–87% relative humidity range at room temperature for the desorption cycle (Fig. S38).

Relative humidity / %	τ_1 / ms	χ^2
87.0	3.148(9)	1.0952
76.9	3.222(10)	1.0231
67.2	3.309(10)	1.0796
57.1	3.458(10)	1.0705
46.6	3.669(10)	1.0787
36.6	3.857(10)	1.0644
26.3	4.103(11)	1.0734
20.7	4.249(11)	1.0654

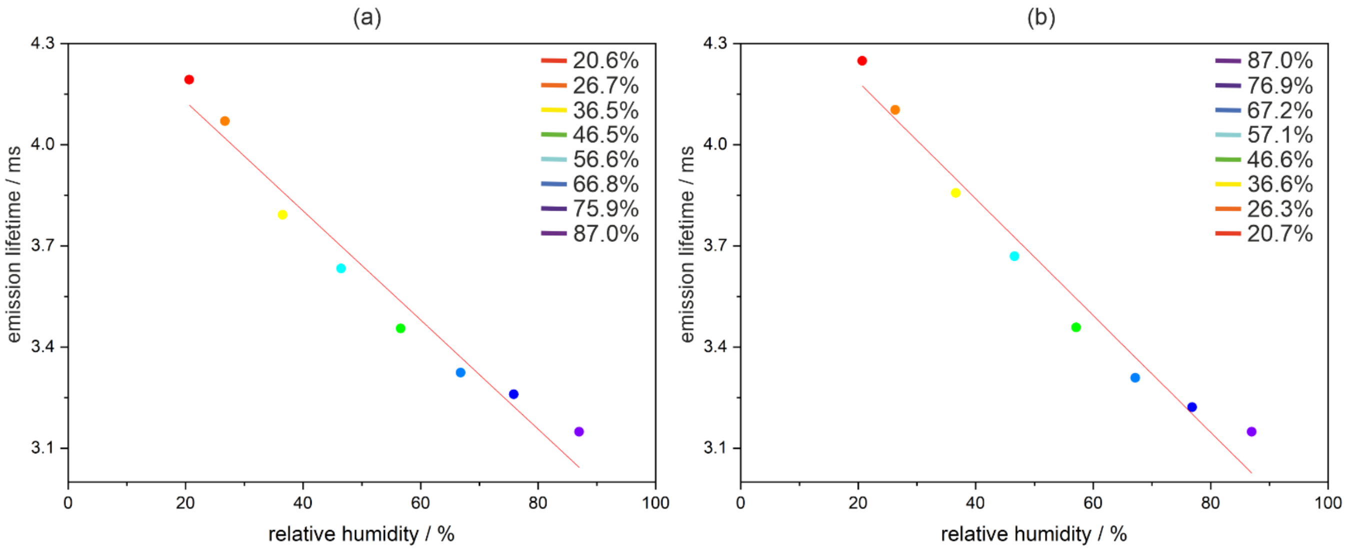

Fig. S39 Humidity dependencies (20–87% RH range) of emission lifetime of **1** at room temperature, for the sorption cycle (a) and the desorption cycle (b), shown with the best linear fits (red solid lines). The related best-fit parameters are gathered in Table S26, while the related emission decay profiles are shown in Fig. S37 and S38.

Table S26 Best-fit parameters for the humidity-dependences of emission lifetime of **1** for the sorption and desorption cycles in the 20–87% relative humidity range at room temperature. The parameters are the results of fitting to the linear function: y = *a*x + *b*, where y stands for the emission lifetime, while x corresponds to the relative humidity value from the 20–87% range (see Fig. 39 for comparison).

Cycle	*a*	*b*	R^2
sorption	-0.0173(13)	4.53(7)	0.9842
desorption	-0.0162(13)	4.45(8)	0.9813

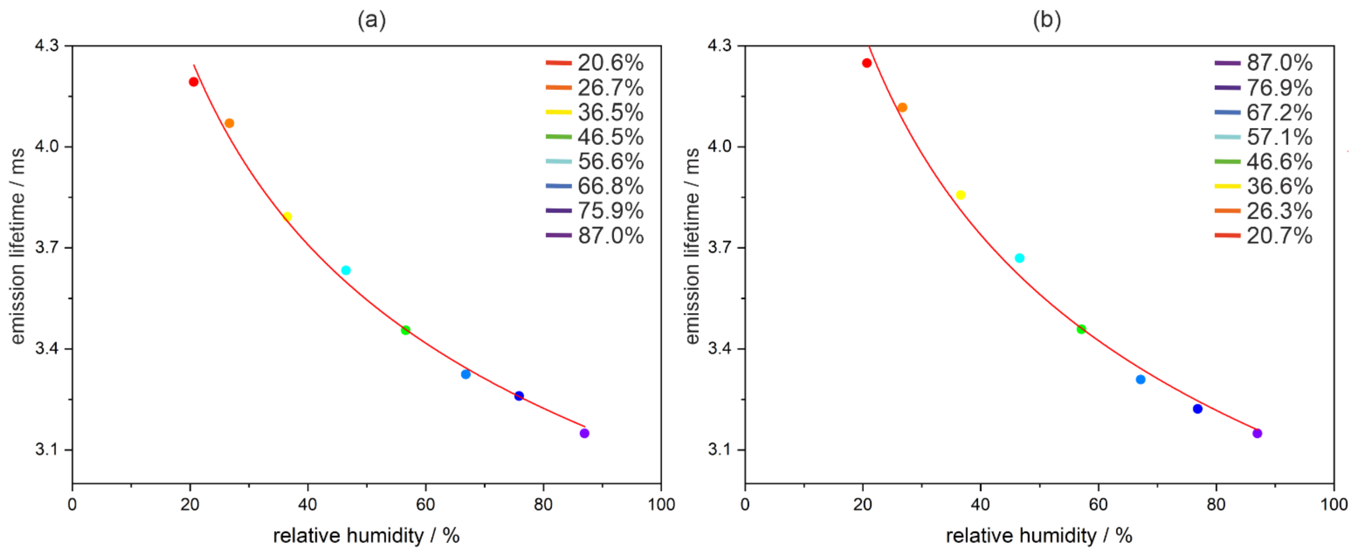

Fig. S40 Humidity dependencies (20–87% RH range) of emission lifetime of **1** at room temperature, for the sorption cycle (a) and the desorption cycle (b), shown with the best fits to the allometric function (red solid lines, see Table S21 for details). The related best-fit parameters are gathered in Table S27, while the related emission decay profiles are shown in Fig. S37 and S38.

Table S27 Best-fit parameters for the humidity-dependences of emission lifetime of **1** for the sorption and desorption cycles in the 20–87% relative humidity range at room temperature. The parameters are the results of fitting to the allometric function: y = ax^b, where y stands for the emission lifetime, while x corresponds to the relative humidity value from the 20–87% range (see Fig. S40 for comparison).

Cycle	*a*	*b*	R^2
sorption	7.83(19)	-0.202(7)	0.9926
desorption	8.31(28)	-0.217(9)	0.9883

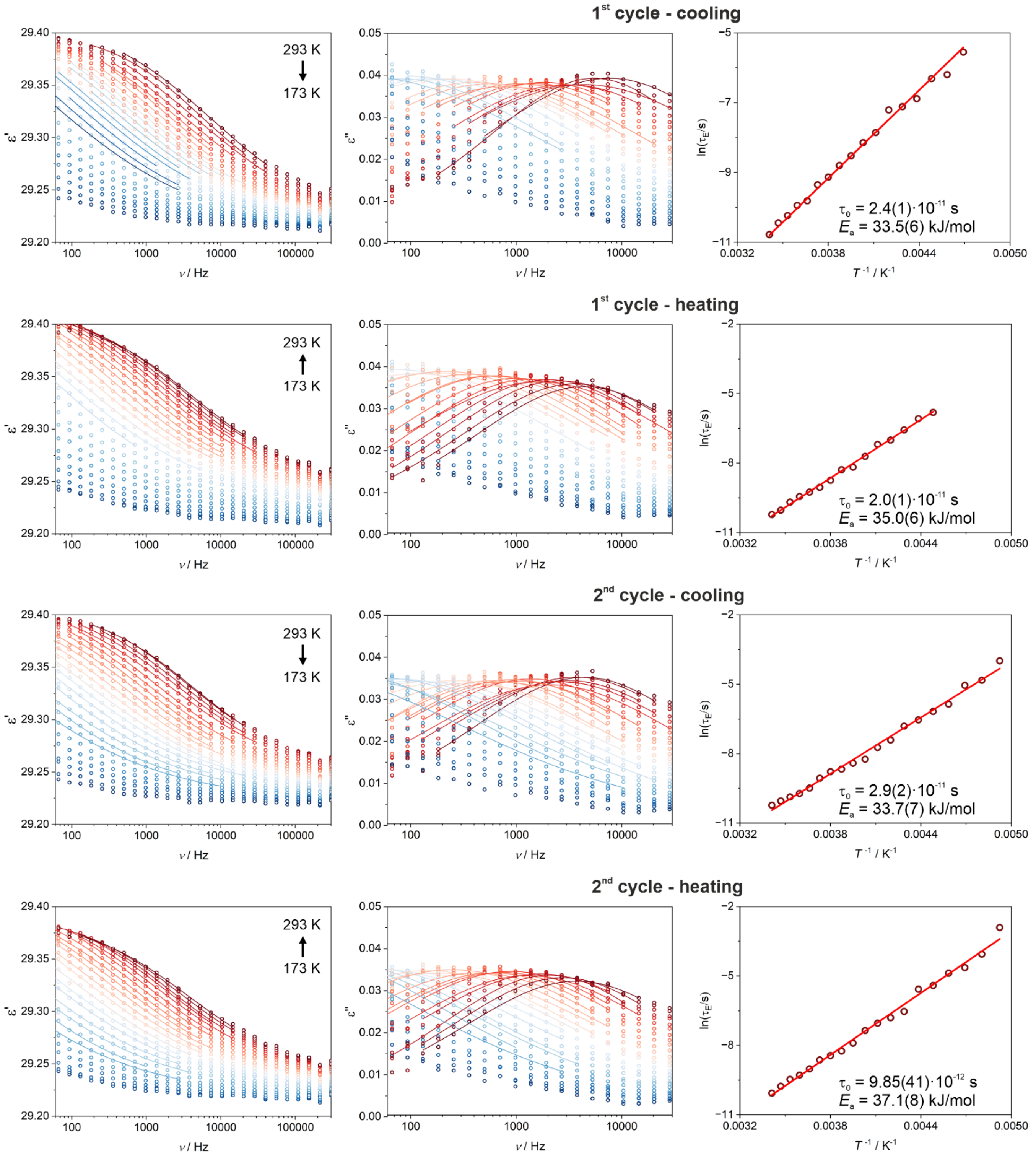

Fig. S41 Temperature-variable dielectric characteristics of **1** upon two cycles of cooling-heating (the 1st cycle and the 2nd cycle) within the 173–293 K range (5 K step, the cooling-heating-cooling-heating sequence starting from 293 K), including frequency dependences of the real part, ε' (left panel), and the imaginary part, ε'' (central panel), of the complex electrical permittivity, shown with the best-fit curves to the generalized Debye model for a single relaxation process (solid lines), and the related temperature dependencies of resulting dielectric relaxation times (right panel), τ_E, with the best fit to the Arrhenius equation (red solid lines). The best-fit parameters for each cycle are given on the respective graphs. They are also gathered in Table S28.

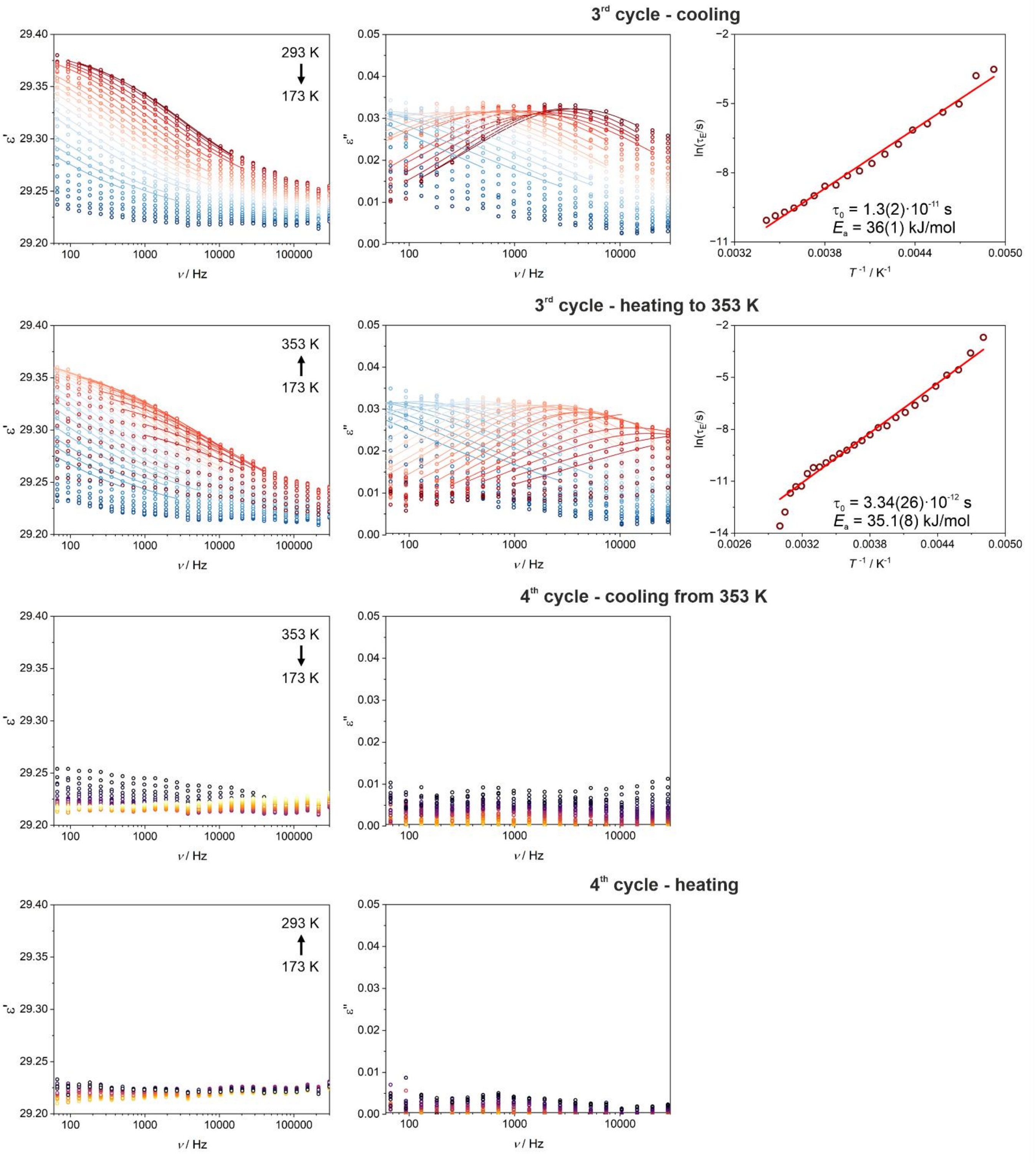

Fig. S42 Temperature-variable dielectric characteristics of **1** upon two cycles of cooling-heating (the 3rd cycle and the 4th cycle, after the cycles shown in Fig. S22) within the 173–353 K range (5 K step, the cooling from 293 to 173 K, then the heating from 173 to 353 K, which leads to the dehydration, then the cooling from 353 to 173 K, and finally the heating from 173 to 293 K), including frequency dependences of the real part, ε' (left panel), and the imaginary part, ε'' (central panel), of the complex electrical permittivity, shown with the best-fit curves to the generalized Debye model for a single relaxation process (solid lines, for the 3rd cycle only), and the related temperature dependencies of resulting dielectric relaxation times (right panel), τ_E, with the best fit to the Arrhenius equation (red solid lines, for the 3rd cycle only). The best-fit parameters are given on the respective graphs. They are also gathered in Table S28.

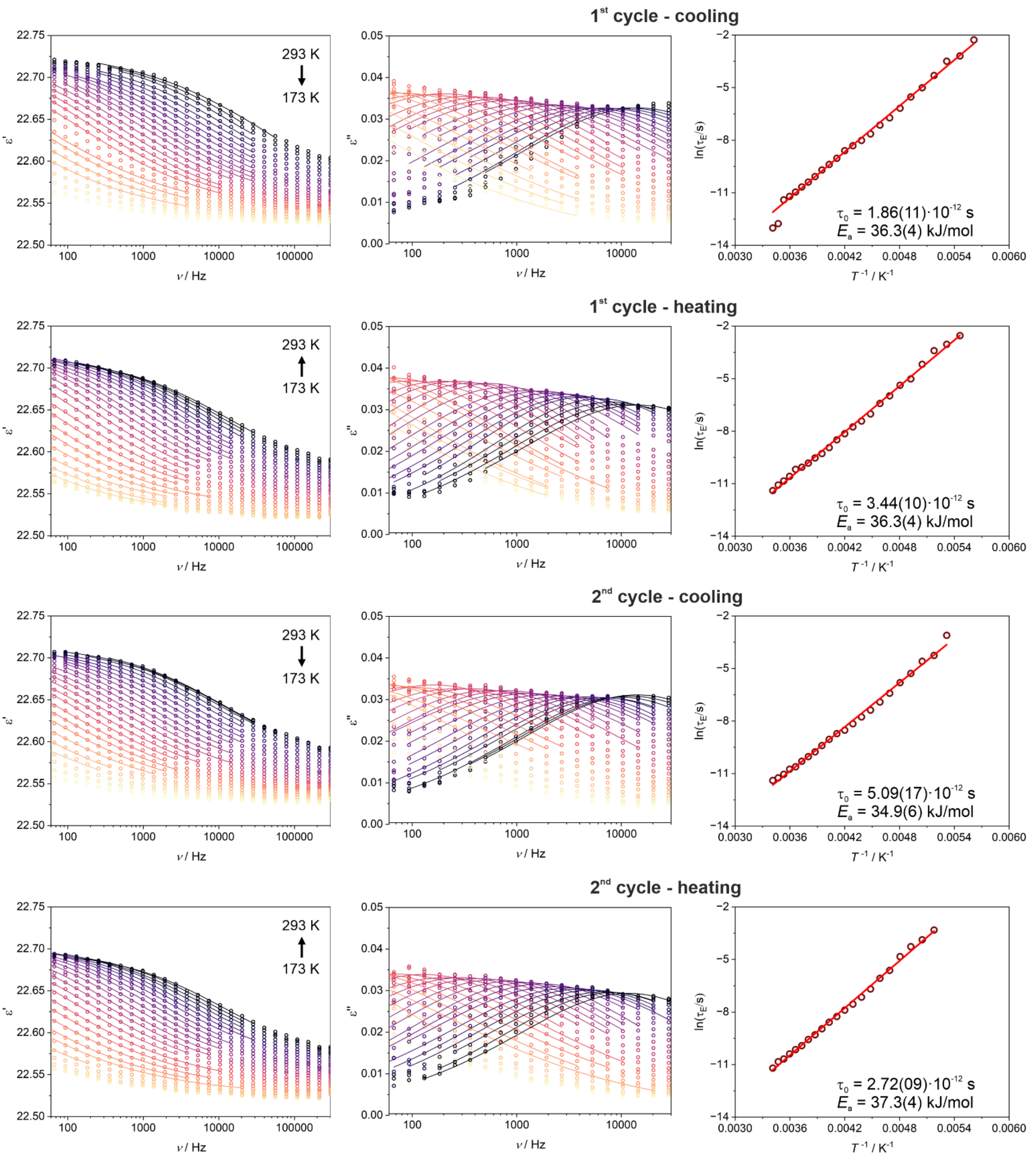

Fig. S43 Temperature-variable dielectric characteristics of **1^reh^** (the re-hydrated sample of **1**) upon two cycles of cooling-heating (the 1st cycle and the 2nd cycle) within the 173–293 K range (5 K step, the cooling-heating-cooling-heating sequence starting from 293 K), including frequency dependences of the real part, *ε*' (left panel), and the imaginary part, *ε*" (central panel), of the complex electrical permittivity, shown with the best-fit curves to the generalized Debye model for a single relaxation process (solid lines), and the related temperature dependencies of resulting dielectric relaxation times (right panel), τ_E, with the best fit to the Arrhenius equation (red solid lines). The best-fit parameters for each cycle are given on the respective graphs. They are also gathered in Table S28.

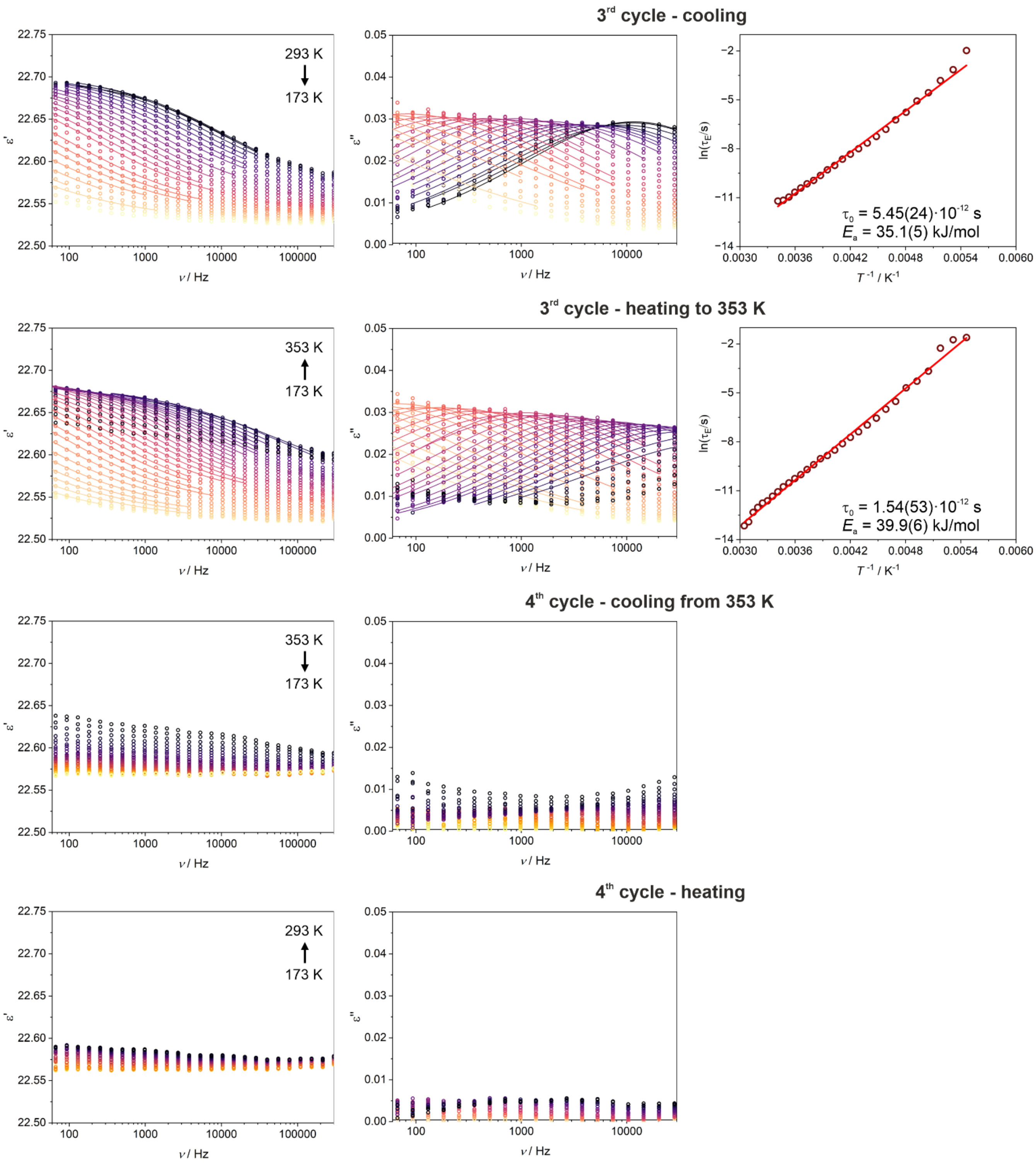

Fig. S44 Temperature-variable dielectric characteristics of **1**^{reh} (the re-hydrated sample of **1**) upon two cycles of cooling-heating (the 3rd cycle and the 4th cycle, after the cycles shown in Fig. S24) within the 173–353 K range (5 K step, the cooling from 293 to 173 K, then the heating from 173 to 353 K, which leads to the dehydration, then the cooling from 353 to 173 K, and finally the heating from 173 to 293 K), including frequency dependences of the real part, ε' (left panel), and the imaginary part, ε'' (central panel), of the complex electrical permittivity, shown with the best-fit curves to the generalized Debye model for a single relaxation process (solid lines, for the 3rd cycle only), and the related temperature dependencies of resulting dielectric relaxation times (right panel), τ_E, with the best fit to the Arrhenius equation (red solid lines, for the 3rd cycle only). The best-fit parameters are given on the respective graphs. They are also gathered in Table S28.

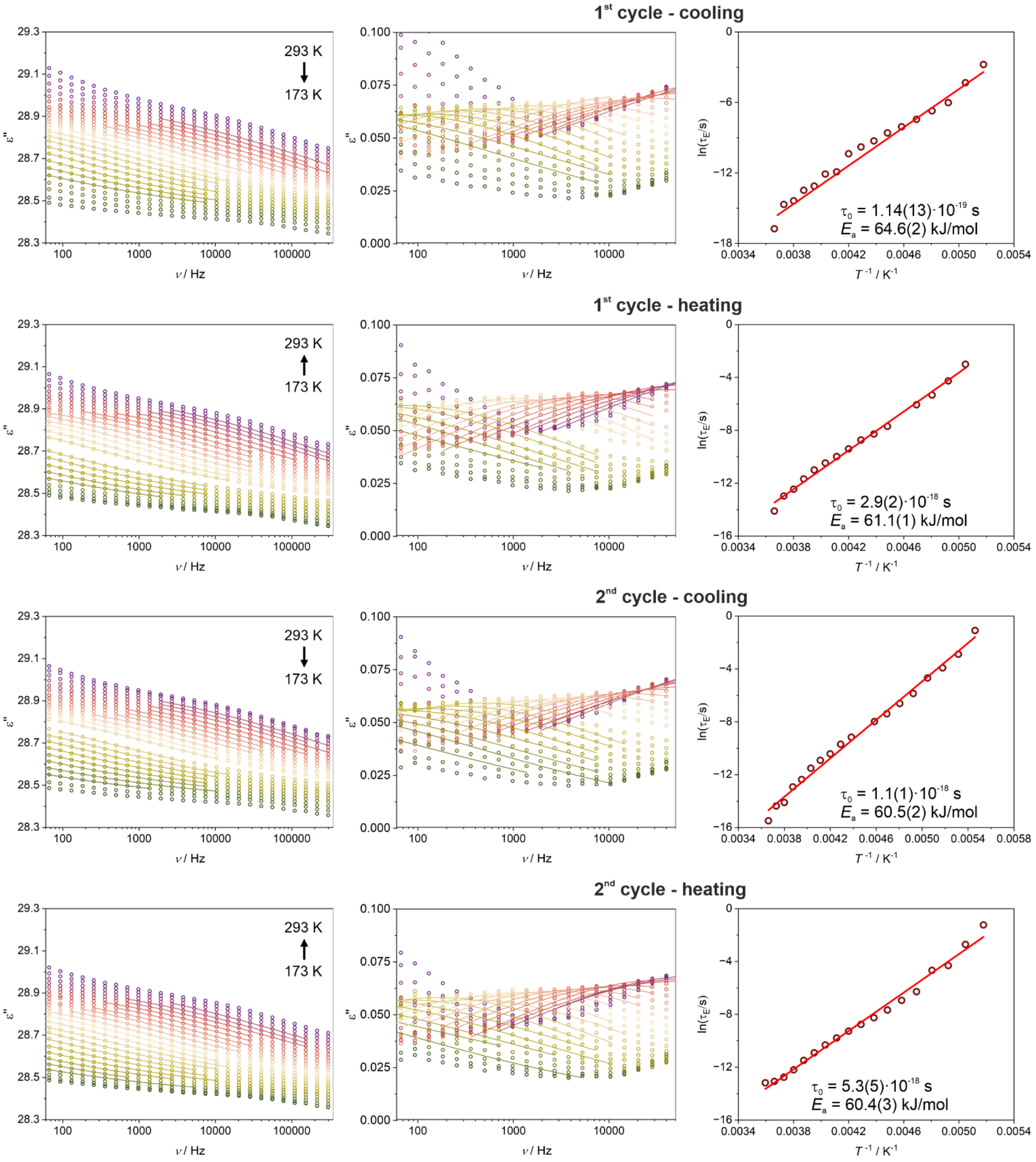

Fig. S45 Temperature-variable dielectric characteristics of **2** upon two cycles of cooling-heating (the 1st cycle and the 2nd cycle) within the 173–293 K range (5 K step, the cooling-heating-cooling-heating sequence starting from 293 K), including frequency dependences of the real part, ε' (left panel), and the imaginary part, ε'' (central panel), of the complex electrical permittivity, shown with the best-fit curves to the generalized Debye model for a single relaxation process (solid lines), and the related temperature dependencies of resulting dielectric relaxation times (right panel), τ_E, with the best fit to the Arrhenius equation (red solid lines). The best-fit parameters for each cycle are given on the respective graphs. They are also gathered in Table S28.

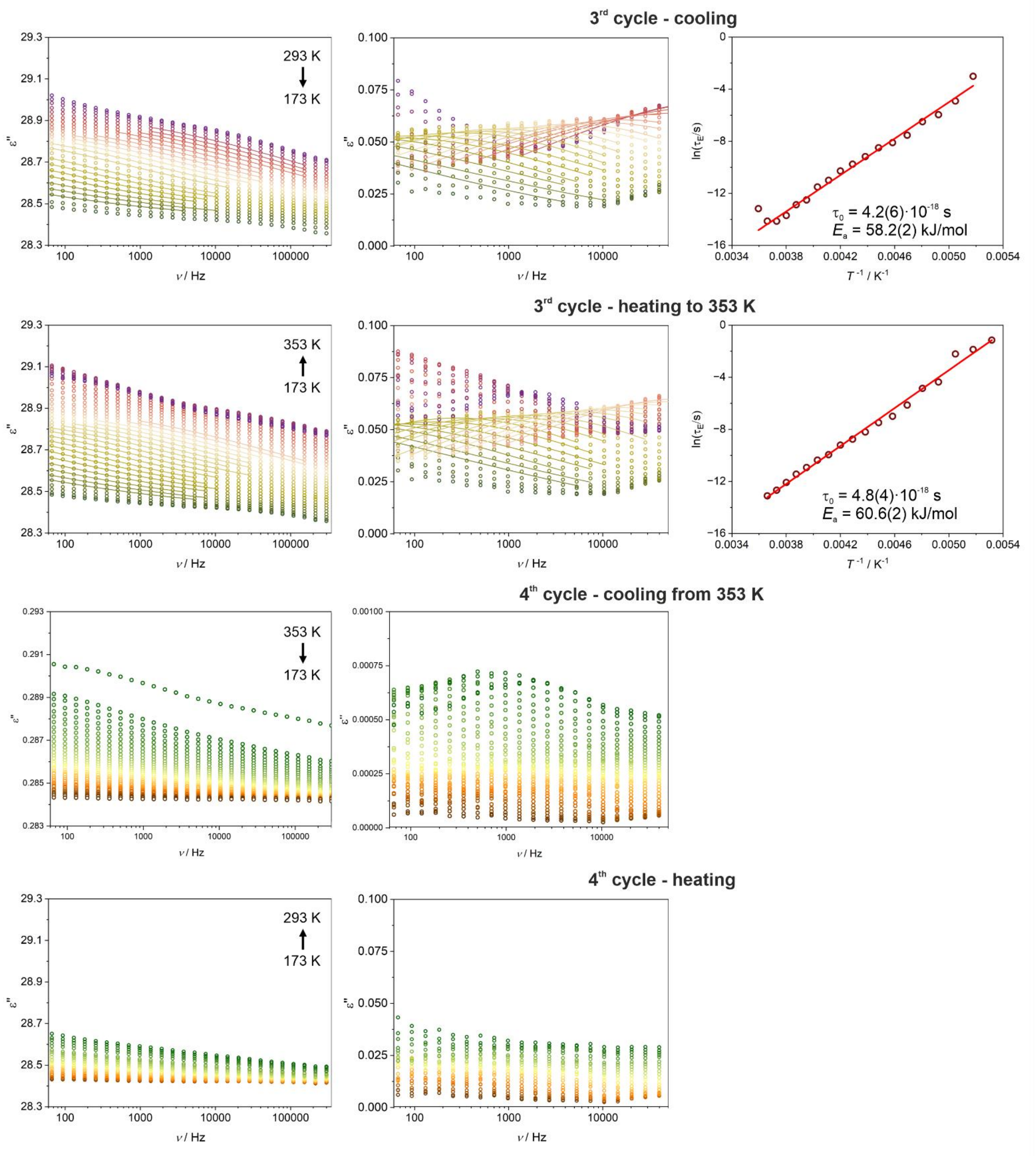

Fig. S46 Temperature-variable dielectric characteristics of **2** upon two cycles of cooling-heating (the 3rd cycle and the 4th cycle, after the cycles shown in Fig. S26) within the 173–353 K range (5 K step, the cooling from 293 to 173 K, then the heating from 173 to 353 K, which leads to the dehydration, then the cooling from 353 to 173 K, and finally the heating from 173 to 293 K), including frequency dependences of the real part, *ε*' (left panel), and the imaginary part, *ε*" (central panel), of the complex electrical permittivity, shown with the best-fit curves to the generalized Debye model for a single relaxation process (solid lines, for the 3rd cycle only), and the related temperature dependencies of resulting dielectric relaxation times (right panel), τ_E, with the best fit to the Arrhenius equation (red solid lines, for the 3rd cycle only). The best-fit parameters are given on the respective graphs. They are also gathered in Table S28.

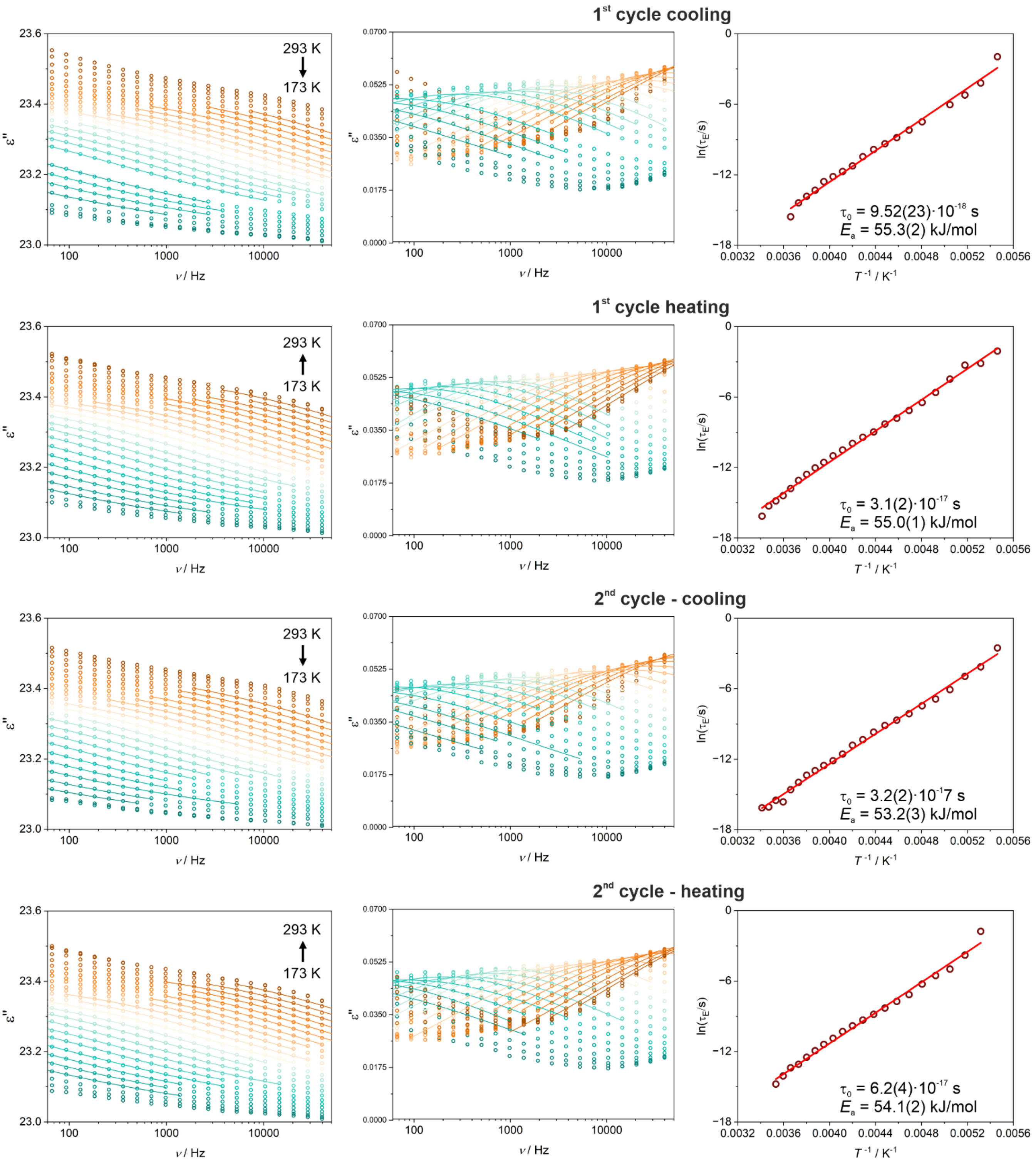

Fig. S47 Temperature-variable dielectric characteristics of **2**$^{\mathbf{reh}}$ (the re-hydrated sample of **2**) upon two cycles of cooling-heating (the 1st cycle and the 2nd cycle) within the 173–293 K range (5 K step, the cooling-heating-cooling-heating sequence starting from 293 K), including frequency dependences of the real part, ε' (left panel), and the imaginary part, ε'' (central panel), of the complex electrical permittivity, shown with the best-fit curves to the generalized Debye model for a single relaxation process (solid lines), and the related temperature dependencies of resulting dielectric relaxation times (right panel), τ_E, with the best fit to the Arrhenius equation (red solid lines). The best-fit parameters for each cycle are given on the respective graphs. They are also gathered in Table S28.

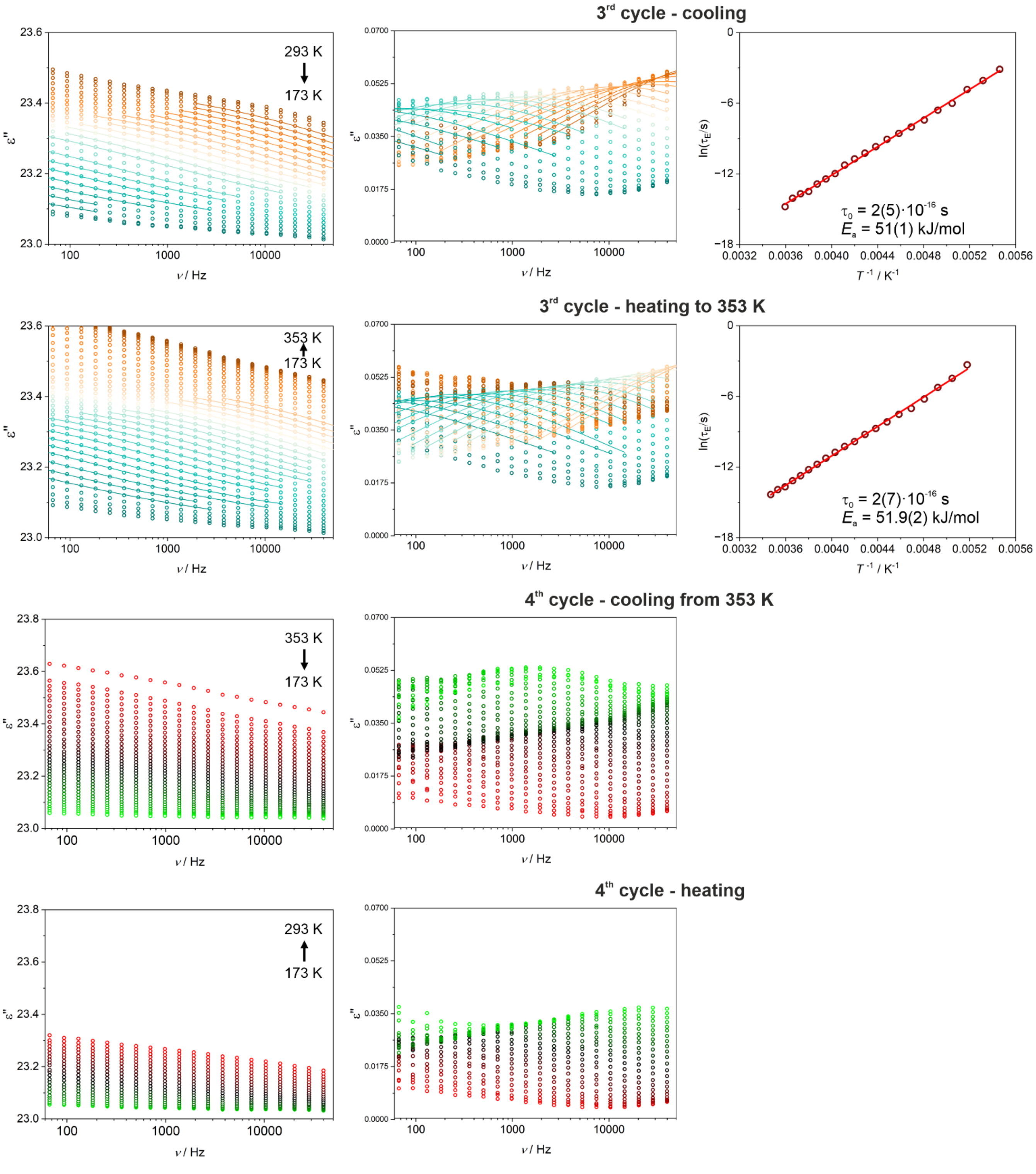

Fig. S48 Temperature-variable dielectric characteristics of **2**^{reh} (the re-hydrated sample of **2**) upon two cycles of cooling-heating (the 3rd cycle and the 4th cycle, after the cycles shown in Fig. S28) within the 173–353 K range (5 K step, the cooling from 293 to 173 K, then the heating from 173 to 353 K, which leads to the dehydration, then the cooling from 353 to 173 K, and finally the heating from 173 to 293 K), including frequency dependences of the real part, ε' (left panel), and the imaginary part, ε'' (central panel), of the complex electrical permittivity, shown with the best-fit curves to the generalized Debye model for a single relaxation process (solid lines, for the 3rd cycle only), and the related temperature dependencies of resulting dielectric relaxation times (right panel), τ_E, with the best fit to the Arrhenius equation (red solid lines, for the 3rd cycle only). The best-fit parameters are given on the respective graphs. They are also gathered in Table S28.

Table S28 Summary of best-fit parameters to the Arrhenius equation used for the temperature dependencies of dielectric relaxation times for compounds **1**, $\mathbf{1^{reh}}$, **2**, and $\mathbf{2^{reh}}$, determined separately for the indicated cycles of cooling and heating (Fig. S41–S48).

Compound	Cycle	τ_0 / s	E_a / kJ/mol
**1**	1st cooling	2.4(1)·10^{-11}	33.5(6)
	1st heating	2.0(1)·10^{-11}	35.0(6)
	2nd cooling	2.9(2)·10^{-11}	33.7(7)
	2nd heating	9.85(41)·10^{-12}	37.1(8)
	3rd cooling	1.3(2)·10^{-11}	36(1)
	3rd heating	3.34(26)·10^{-12}	39.8(8)
$\mathbf{1^{reh}}$ (the re-hydrated sample of **1**)	1st cooling	1.86(11)·10^{-12}	36.3(4)
	1st heating	3.44(10)·10^{-12}	36.3(4)
	2nd cooling	5.09(17) ·10^{-12}	34.9(6)
	2nd heating	2.72(09) ·10^{-12}	37.3(4)
	3rd cooling	5.45(24) ·10^{-12}	35.1(5)
	3rd heating	1.54(53) ·10^{-12}	39.9(6)
**2**	1st cooling	1.14(13)·10^{-19}	64.6(2)
	1st heating	2.9(2)·10^{-18}	61.1(1)
	2nd cooling	1.1(1)·10^{-18}	60.5(2)
	2nd heating	5.3(5)·10^{-18}	60.4(3)
	3rd cooling	4.2(6)·10^{-18}	58.2(2)
	3rd heating	4.8(4)·10^{-18}	60.6(2)
$\mathbf{2^{reh}}$ (the re-hydrated sample of **2**)	1st cooling	9.52(23)·10^{-18}	55.3(2)
	1st heating	3.1(2)·10^{-17}	55.0(1)
	2nd cooling	3.2(2)·10^{-17}	53.2(3)
	2nd heating	6.2(4)·10^{-17}	54.1(2)
	3rd cooling	2(5)·10^{-16}	51(1)
	3rd heating	2(7)·10^{-16}	51.9(2)

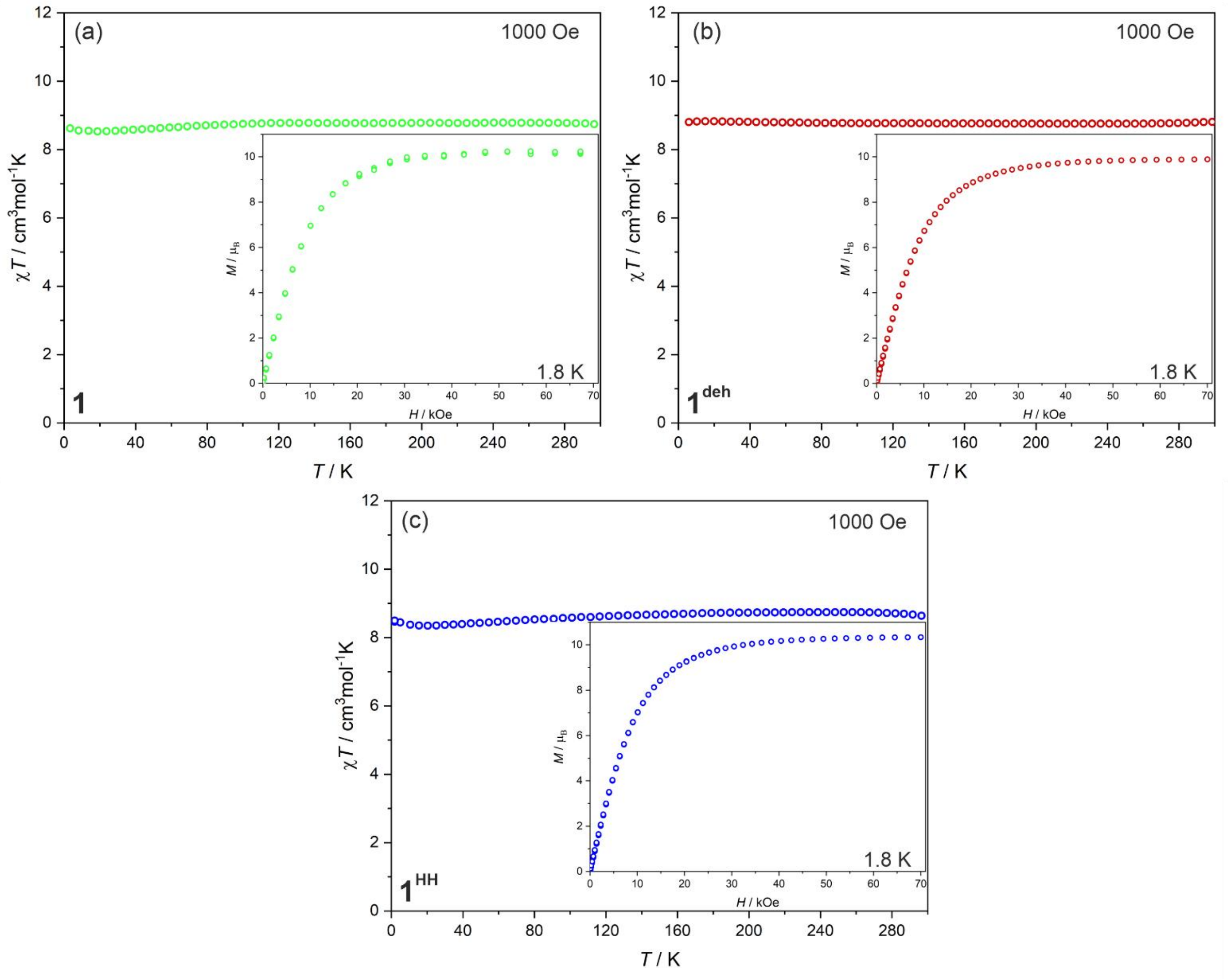

Fig. S49 Direct-current (*dc*) magnetic characteristics of **1** (a, 23% RH), $\mathbf{1^{deh}}$ (b, 0% RH), and $\mathbf{1^{HH}}$ (c, the sample of **1** at 90% RH), including the temperature dependencies of the $\chi_M T$ product under the indicated external magnetic field and the field dependencies of molar magnetization (*M*) collected at 1.8 K (the insets).

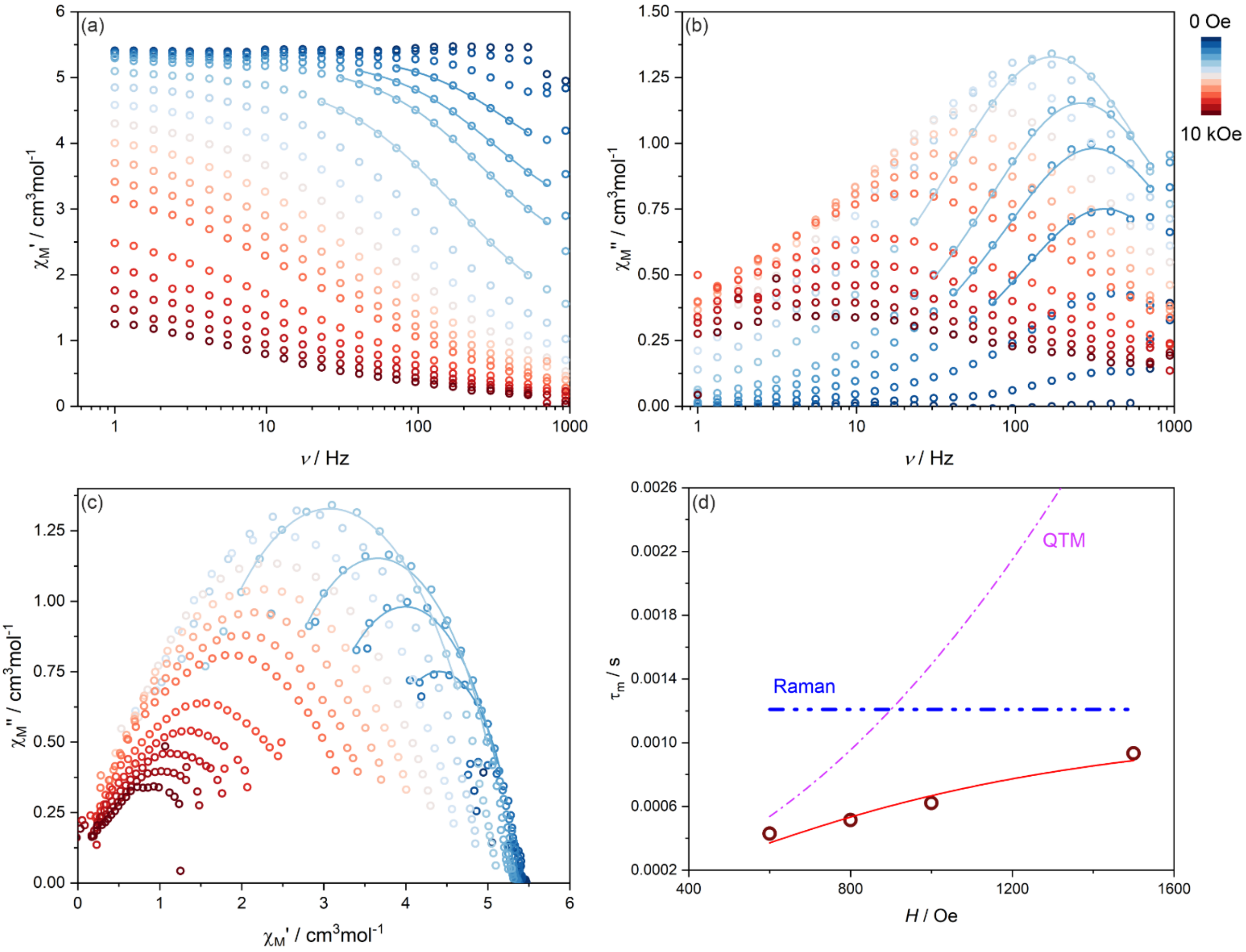

Fig. S50 Magnetic-field-variable alternate-current (*ac*) magnetic characteristics of **1** (23% RH) at T = 1.8 K, including the frequency dependences of the in-phase susceptibility, χ_M', (a) and the out-of-phase susceptibility, χ_M'', (b) under variable indicated *dc* fields, the related Argand plots (c), and the field dependence of resulting magnetic relaxation times, τ_m (d). Colored solid lines in (a–c) represent the best fits using the generalized Debye model for a single relaxation process. The red solid line in (d) shows the best fit taking into account indicated relaxation processes (the result of the simultaneous fit with the temperature-dependent relaxation times shown in Fig. S51) while the dashed colored lines represent the respective course of individual magnetic relaxation processes. Best-fit parameters for the (d) part are gathered in Table S29.

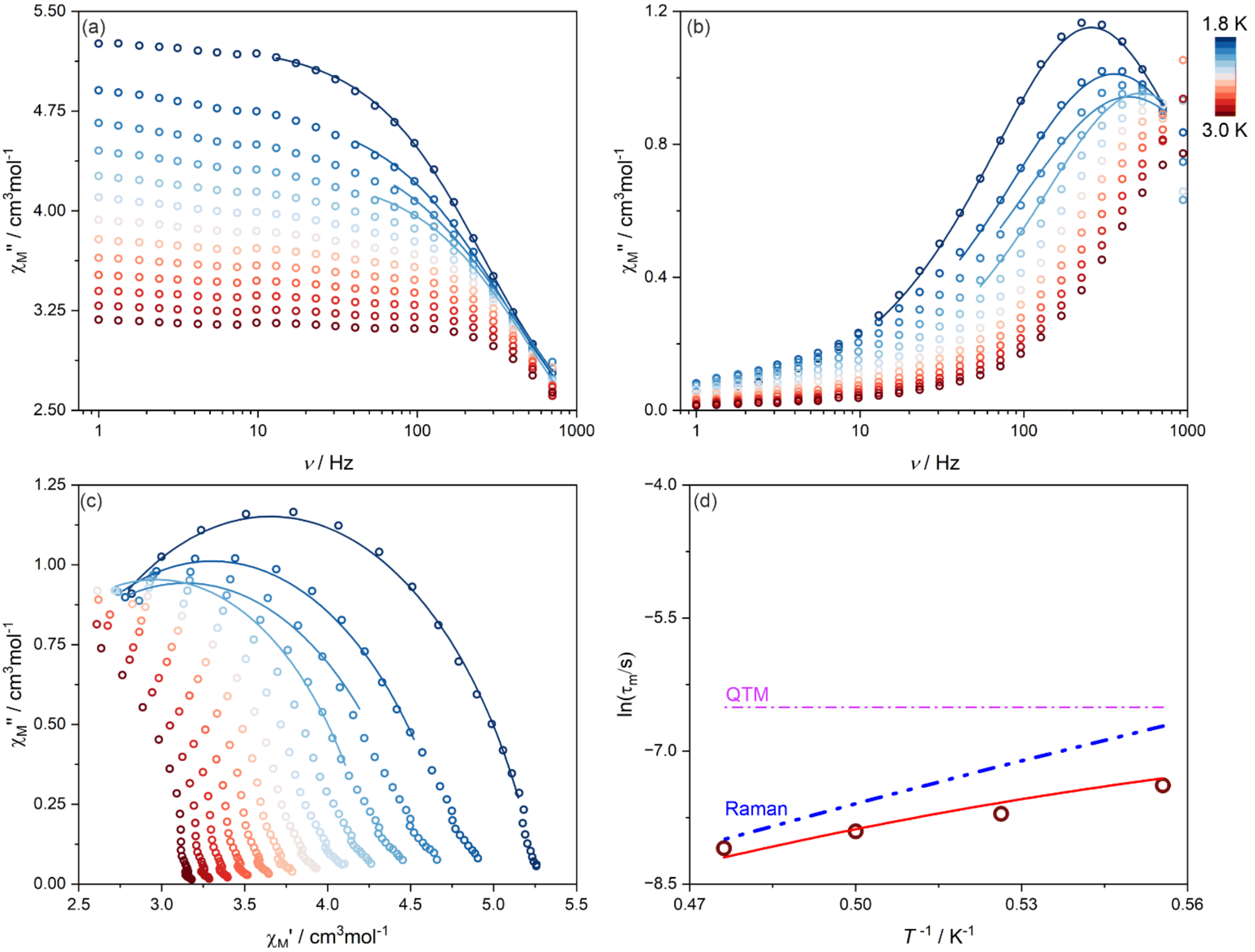

Fig. S51 Temperature-variable alternate-current (*ac*) magnetic characteristics of **1** (23% RH) at H_{dc} = 1000 Oe, including the frequency dependences of the in-phase susceptibility, χ_M', (a) and the out-of-phase susceptibility, χ_M'', (b) under variable indicated *dc* fields, the related Argand plots (c), and the field dependence of resulting magnetic relaxation times, τ_m (d). Colored solid lines in (a–c) represent the best fits using the generalized Debye model for a single relaxation process. The red solid line in (d) shows the best fit taking into account indicated relaxation processes (the result of the simultaneous fit with the magnetic-field-dependent relaxation times shown in Fig. S50) while the dashed colored lines represent the respective course of individual magnetic relaxation processes. Best-fit parameters for the (d) part are gathered in Table S29.

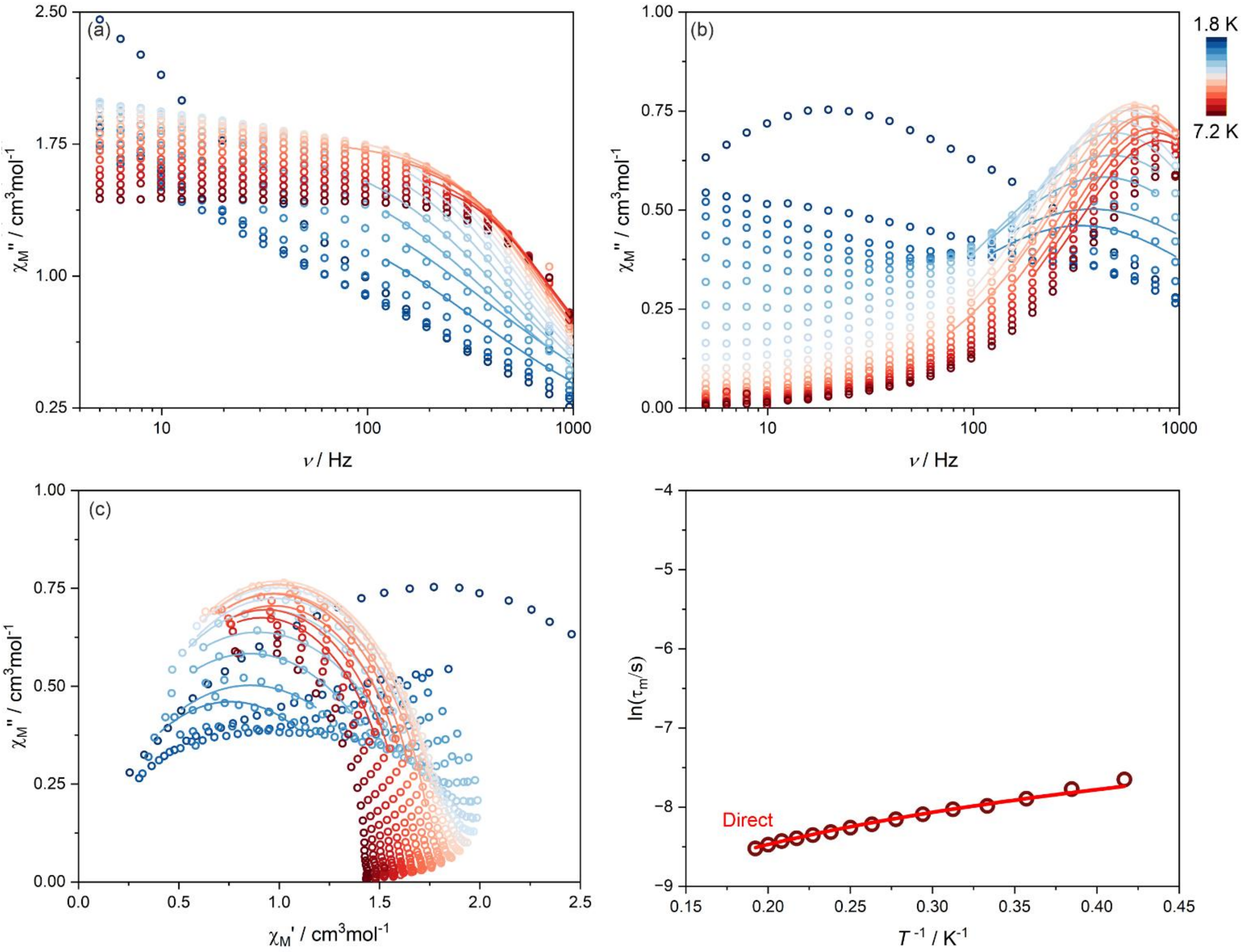

Fig. S52 Temperature-variable alternate-current (*ac*) magnetic characteristics of **1** (23% RH) at H_{dc} = 5000 Oe, including the frequency dependences of the in-phase susceptibility, χ_M', (a) and the out-of-phase susceptibility, χ_M'', (b) under variable indicated *dc* fields, the related Argand plots (c), and the field dependence of resulting magnetic relaxation times, τ_m (d). Colored solid lines in (a–c) represent the best fits using the generalized Debye model for a single relaxation process. The red solid line in (d) shows the best fit taking into account the indicated relaxation process. Best-fit parameters for the (d) part are gathered in Table S30.

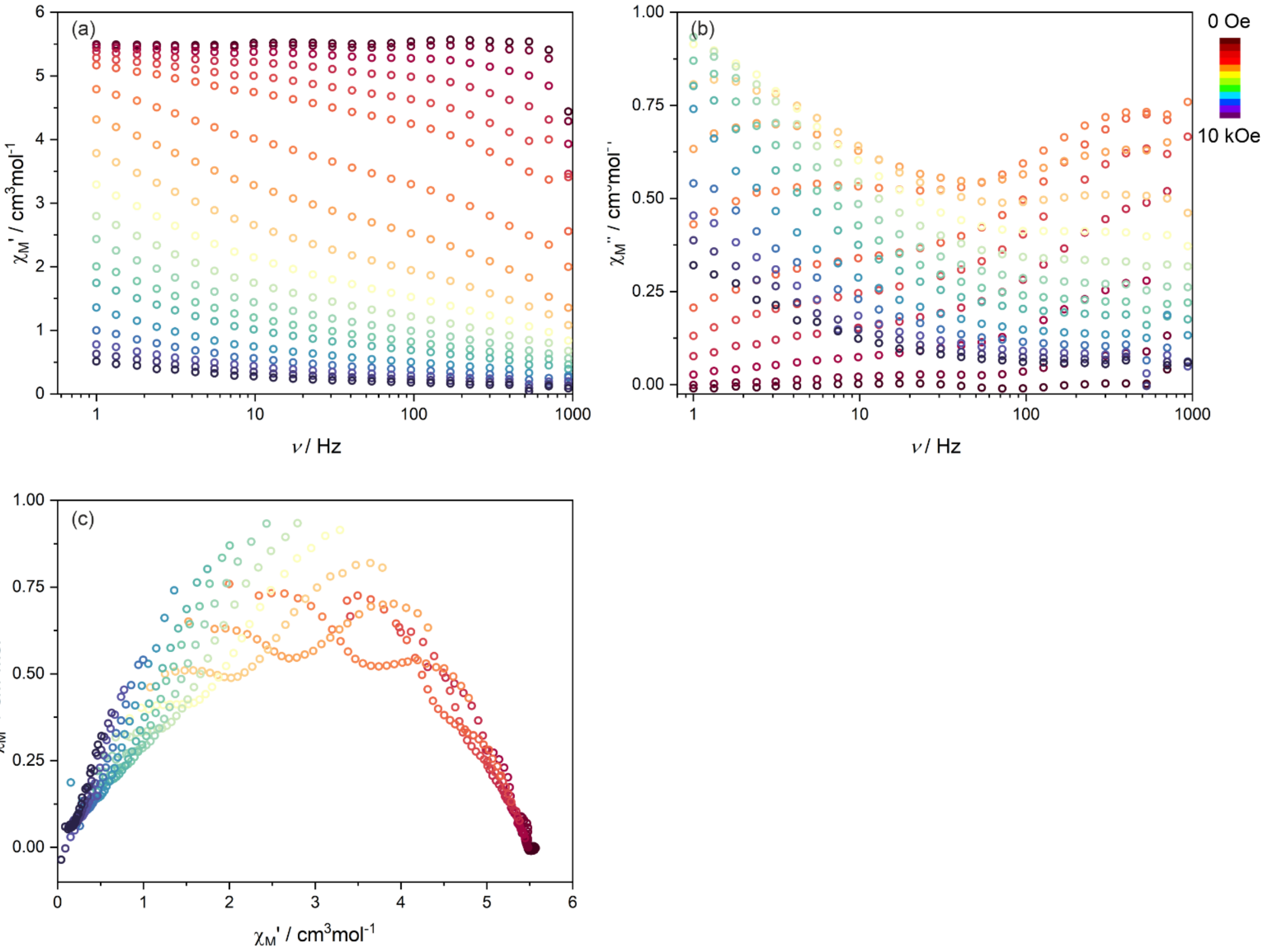

Fig. S53 Magnetic-field-variable alternate-current (*ac*) magnetic characteristics of **1**^{deh} (0% RH) at *T* = 1.8 K, including the frequency dependences of the in-phase susceptibility, χ_M', (a) and the out-of-phase susceptibility, χ_M'', (b) under variable indicated *dc* fields, and the related Argand plots (c). Due to the lack of distinct maxima on the χ_M'' versus *ac* field frequency plots (b), the related magnetic relaxation times could not be determined from this set of data.

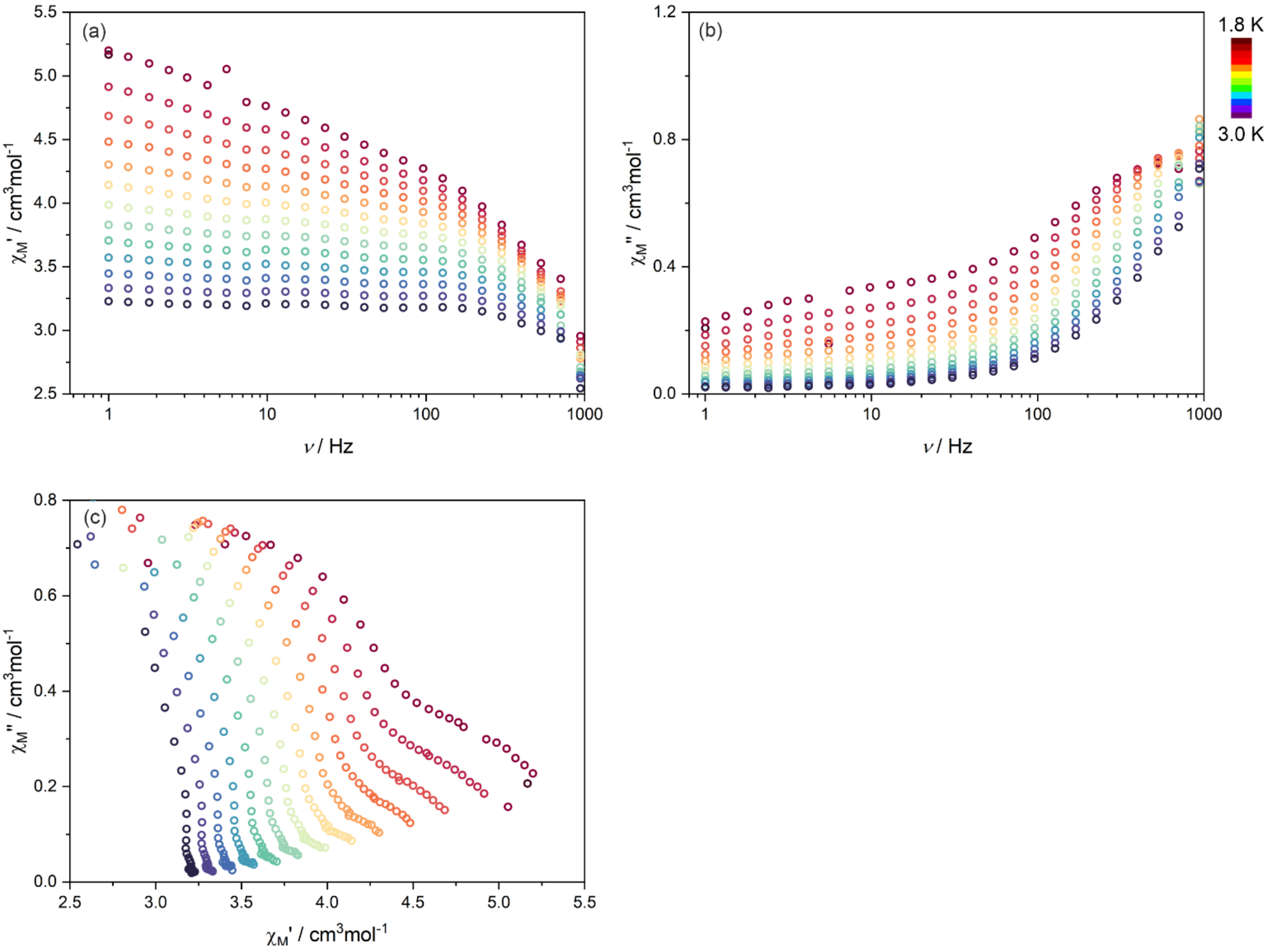

Fig. S54 Temperature-variable alternate-current (*ac*) magnetic characteristics of **1**$^{\mathbf{deh}}$ (0% RH) at H_{dc} = 1000 Oe, including the frequency dependences of the in-phase susceptibility, χ_M', (a) and the out-of-phase susceptibility, χ_M'', (b) under variable indicated *dc* fields, and the related Argand plots (c). Due to the lack of distinct maxima on the χ_M'' versus *ac* field frequency plots (b), the related magnetic relaxation times could not be determined from this set of data.

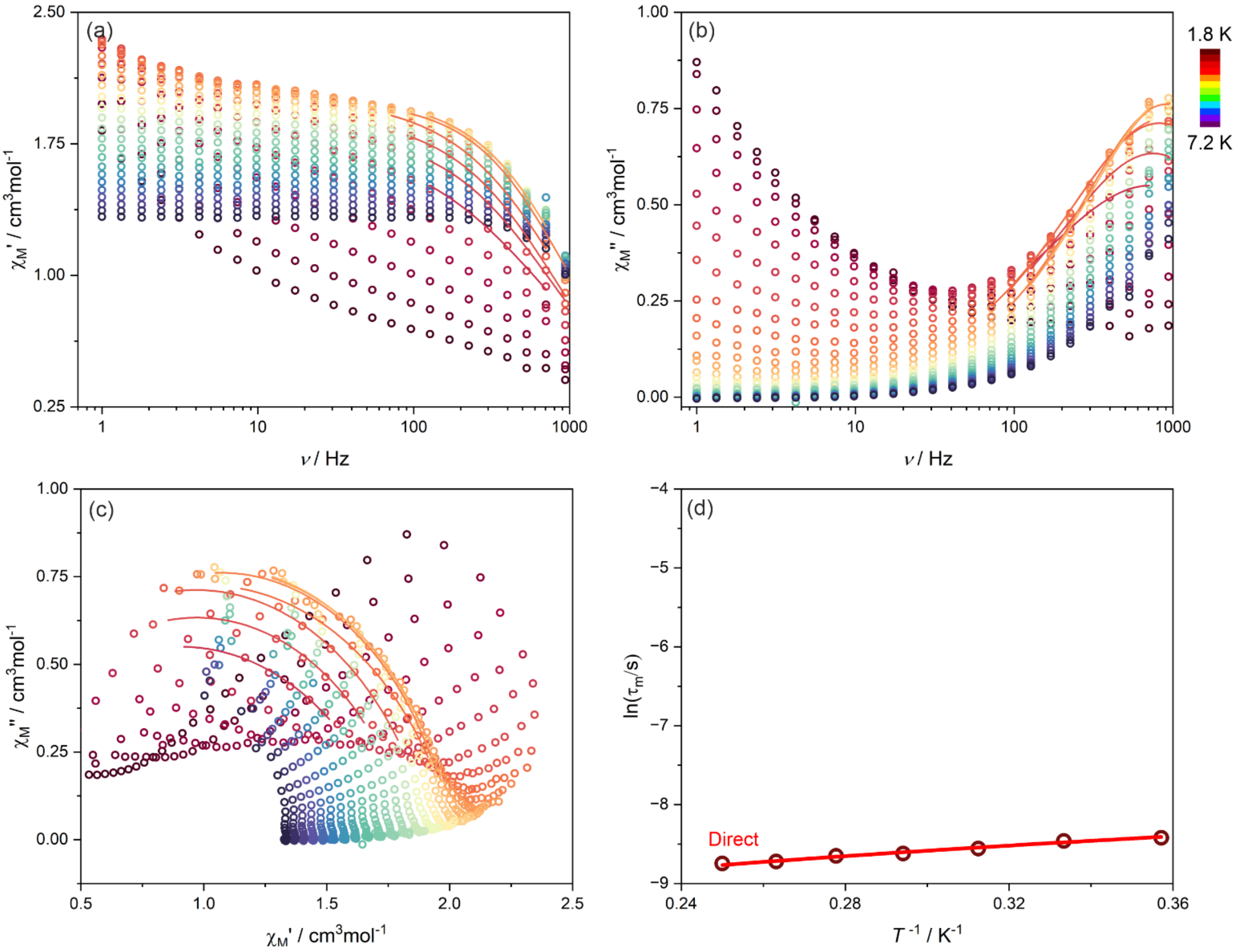

Fig. S55 Temperature-variable alternate-current (*ac*) magnetic characteristics of $\mathbf{1^{deh}}$ (0% RH) at H_{dc} = 5000 Oe, including the frequency dependences of the in-phase susceptibility, χ_M', (a) and the out-of-phase susceptibility, χ_M'', (b) under variable indicated *dc* fields, the related Argand plots (c), and the field dependence of resulting magnetic relaxation times, τ_m (d). Colored solid lines in (a–c) represent the best fits using the generalized Debye model for a single relaxation process. The red solid line in (d) shows the best fit taking into account the indicated relaxation process. Best-fit parameters for the (d) part are gathered in Table S30.

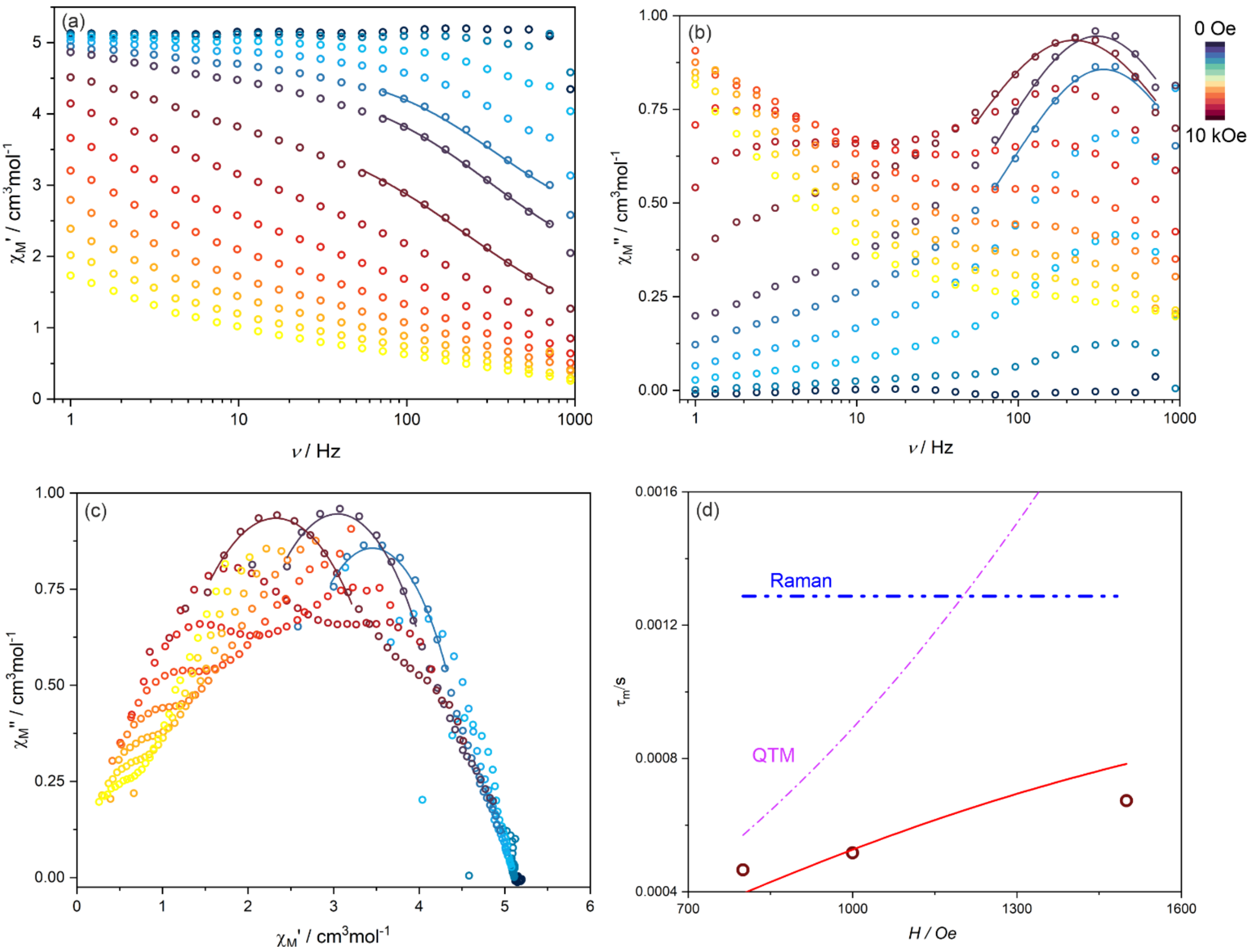

Fig. S56 Magnetic-field-variable alternate-current (*ac*) magnetic characteristics of **1^HH^** (90% RH) at T = 1.8 K, including the frequency dependences of the in-phase susceptibility, χ_M', (a) and the out-of-phase susceptibility, χ_M'', (b) under variable indicated *dc* fields, the related Argand plots (c), and the field dependence of resulting magnetic relaxation times, τ_m (d). Colored solid lines in (a–c) represent the best fits using the generalized Debye model for a single relaxation process. The red solid line in (d) shows the best fit taking into account indicated relaxation processes (the result of the simultaneous fit with the temperature-dependent relaxation times shown in Fig. S57) while the dashed colored lines represent the respective course of individual magnetic relaxation processes. Best-fit parameters for the (d) part are gathered in Table S29.

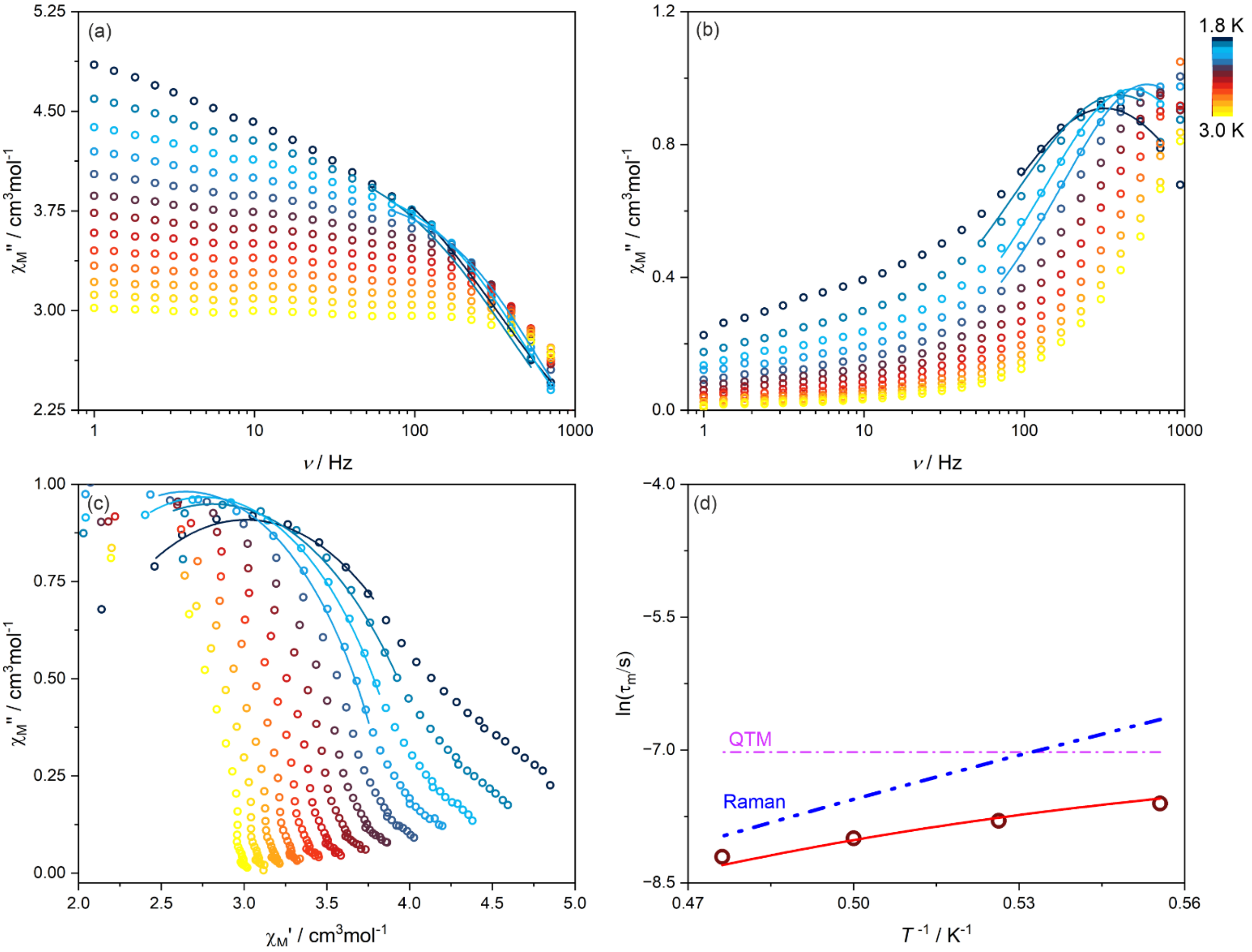

Fig. S57 Temperature-variable alternate-current (*ac*) magnetic characteristics of $\mathbf{1}^{\mathbf{HH}}$ (90% RH) at H_{dc} = 1000 Oe, including the frequency dependences of the in-phase susceptibility, χ_M', (a) and the out-of-phase susceptibility, χ_M'', (b) under variable indicated *dc* fields, the related Argand plots (c), and the field dependence of resulting magnetic relaxation times, τ_m (d). Colored solid lines in (a–c) represent the best fits using the generalized Debye model for a single relaxation process. The red solid line in (d) shows the best fit taking into account indicated relaxation processes (the result of the simultaneous fit with the magnetic-field-dependent relaxation times shown in Fig. S56) while the dashed colored lines represent the respective course of individual magnetic relaxation processes. Best-fit parameters for the (d) part are gathered in Table S29.

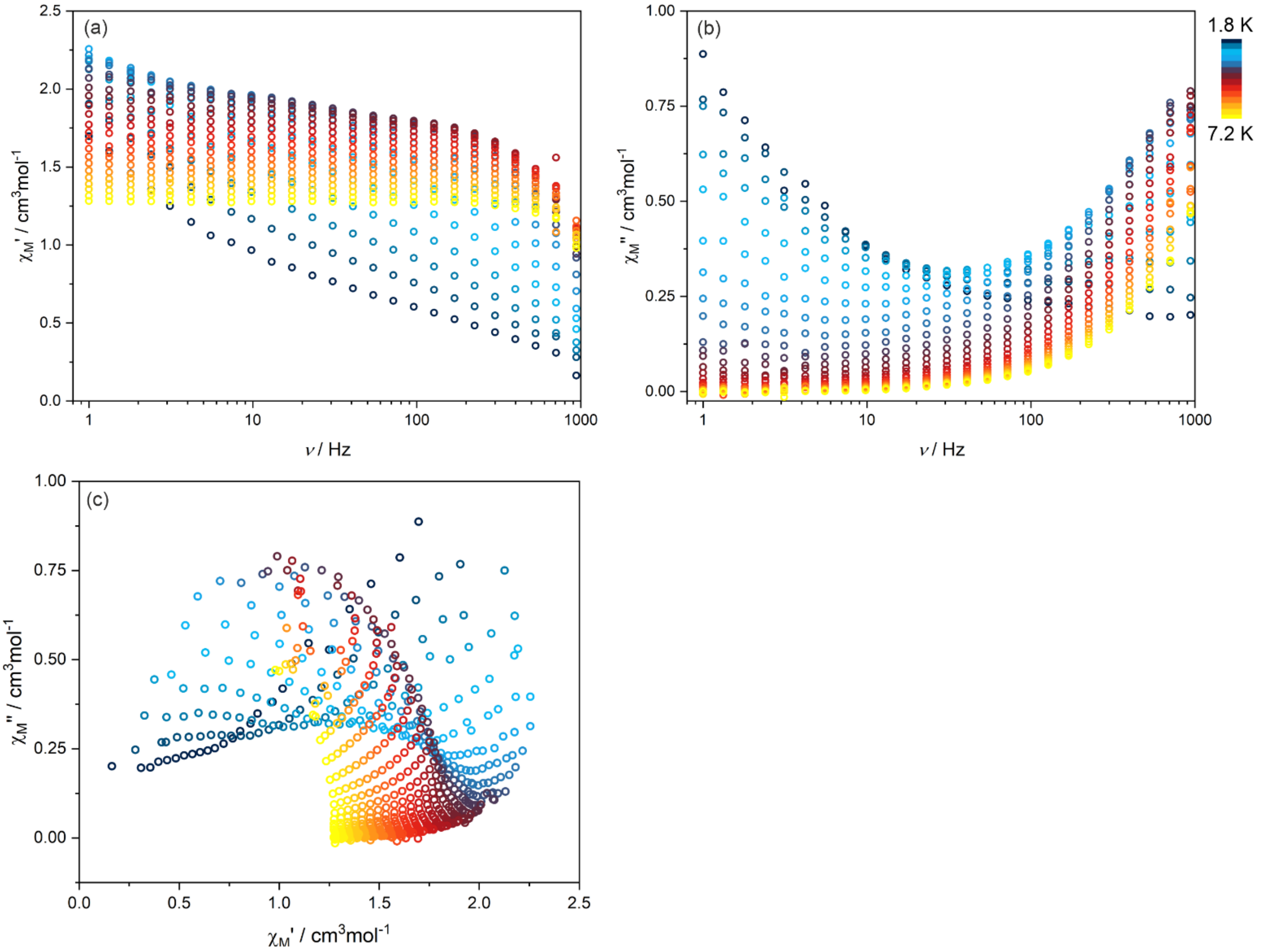

Fig. S58 Temperature-variable alternate-current (*ac*) magnetic characteristics of **1**$^{\mathbf{HH}}$ (90% RH) at H_{dc} = 5000 Oe, including the frequency dependences of the in-phase susceptibility, χ_M', (a) and the out-of-phase susceptibility, χ_M'', (b) under variable indicated *dc* fields, and the related Argand plots (c). Due to the lack of distinct maxima on the χ_M'' versus *ac* field frequency plots (b), the related magnetic relaxation times could not be determined from this set of data.

Table S29 Summary of the best-fit slow magnetic relaxation parameters for **1** and **1^{HH}** obtained within the three-dimensional simultaneous fitting of the field dependence at $T = 1.8$ K and the temperature dependence at H_{dc} =1000 Oe of magnetic relaxation times (Fig. S50, S51, S56, and S57). For the related equation and discussion see the main text, i.e., equation (4).

Compound	a / s^{-1}	b / Oe^{-2}	c / Oe^{-2}	B_{Raman} / s^{-1} K^{-n}	n
**1** (23% RH)	$4.27{\cdot}10^{11}$	636.42	0 (fixed)	6.29	8.30
**1^{HH}** (90% RH)	$3.16{\cdot}10^{11}$	281.84	0 (fixed)	5.11	8.55

Table S30 Summary of the best-fit slow magnetic relaxation parameters for **1** and **1^{deh}** obtained within the fitting of the temperature dependences at H_{dc} = 5000 Oe of magnetic relaxation times (Fig. S52 and S55). For the related equation and discussion see the main text, i.e., equation (5).

Compound	A_{direct} / s^{-1} K^{-1} Oe^{-m}	m
**1** (23% RH)	5.57	0.60
**1^{deh}** (0% RH)	6.33	0.65

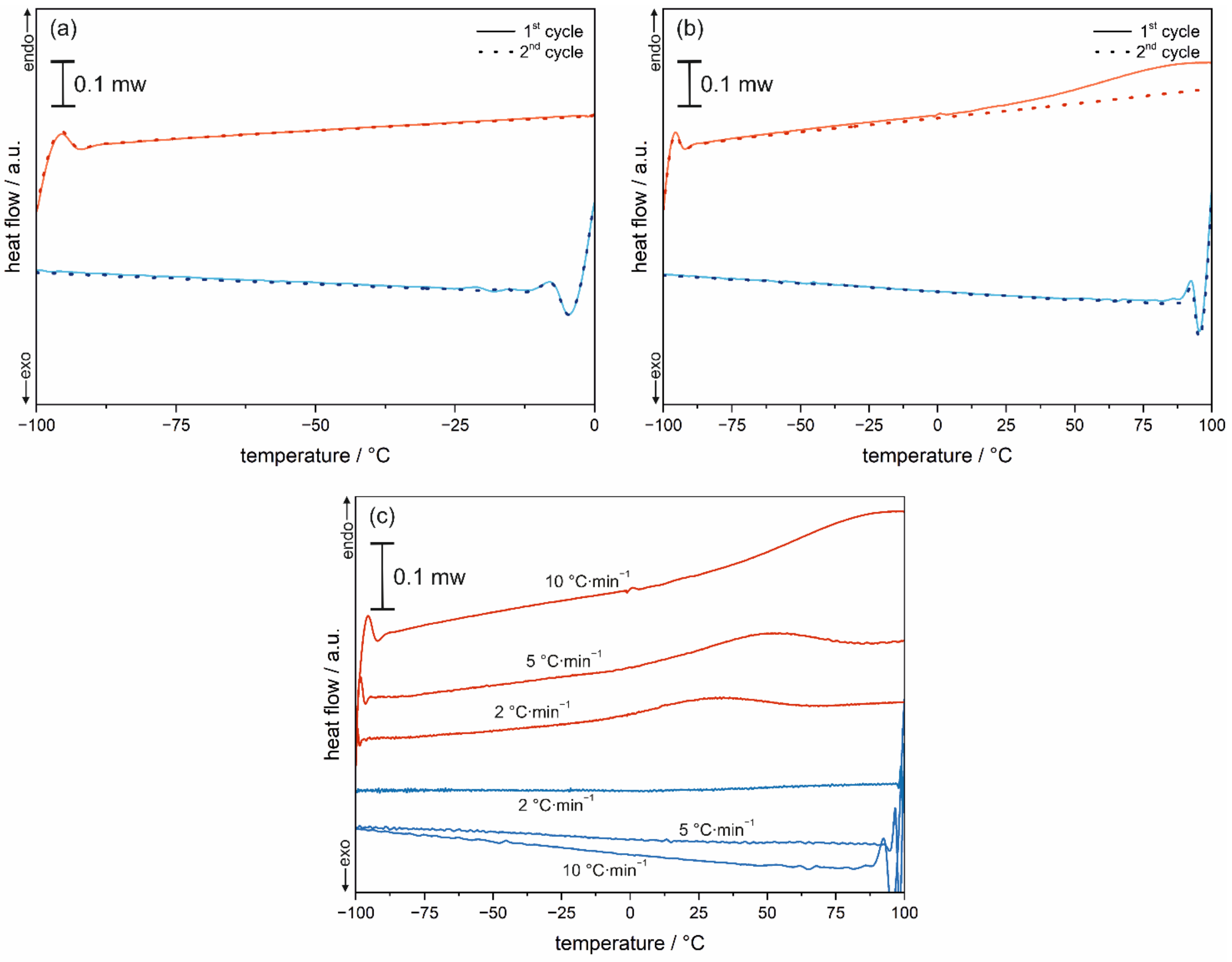

Fig. S59 DSC curves of **1** gathered in the variable temperature ranges and sweeping rates, including two consecutive cycles of heating and cooling in the (–100)–0 °C range with the sweeping rate of 10 °C·min^{-1} (a), two consecutive cycles of heating and cooling in the (–100)–100 °C range with the sweeping rate of 10 °C·min^{-1} (b), and three separate cycles of heating and cooling in the (–100)–100 °C range with three different indicated sweeping rates of 2, 5, and 10 °C·min^{-1} (c). Red curves correspond to the heating mode, while blue ones to the cooling mode.

Comment to Fig. S59:

To additionally support the interpretation of dielectric relaxations in **1** as well as investigate more precisely the dehydration process in this material, the DSC curves were gathered in the variable temperature ranges and sweeping rates. First, two consecutive measurement cycles upon heating and cooling in the (–100)–0 °C range with the sweep rate of 10 °C·min^{-1} were performed (Fig. S59a). In this range, the dielectric relaxations were observed (Fig. 8 and 9). The DSC curves for this range do not reveal any, even very shallow, peaks (except for the experimental deviations in the signal upon starting the cooling or heating sequences). This indicates the lack of typical phase transitions in the investigated range which agrees with the assignment of the observed dielectric relaxations to the Bjerrum-type orientation defects of water molecules of crystallization embedded in the crystal structure of **1** (for such dielectric relaxation effects, the calorimetric signatures are not observed).[S18–S23] Thus, the eventual order-disorder phase transitions, which are sometimes detectable for the tetrahedral $[Mn^{II}Cl_4]^{2-}$ ions (or other tetrahedral 3d metal complexes) embedded in molecular materials,[S24,S25] can be excluded due to the lack of related peaks on the DSC curves. The order-disorder phase transitions were also not visible in the structural studies performed at various temperatures (Fig. S4 and S5). We limited this first part of the DSC studies to 0 °C to avoid the dehydration that occurs around room temperature, e.g., at 300 K in the flow of dry inert gas (Fig. S6).

In the second part of the DSC studies, we performed the calorimetric measurements upon two consecutive cycles of heating and cooling in the broader (–100)–100 °C range with the sweeping rate of 10 °C·min^{-1} (Fig. S59b). In the first cycle of heating, the broad positive (endothermic) DSC peak was observed in the 25–100 °C range. The analogous negative (exothermic) DSC peak did not appear upon the subsequent heating. In the second cycle, upon heating, the positive DSC peak also disappears. Therefore, it can be undoubtedly assigned to the dehydration process which is irreversible as the measurement cycles are performed in the atmosphere of dry inert gas, so rehydration is not possible. This dehydration-related DSC peak is very broad due to the fast sweeping rate typically used for DSC studies to better detect often weak calorimetric effects. Moreover, the position of this DSC peak in the broad 25–100 °C range (the shoulder is expected to be even lying at higher temperatures, besides the accessible temperature range) might suggest that the dehydration occurs in the very broad temperature range and it is not completed even at 100 °C. On the other hand, the structural studies indicate that even at 300 K (27 °C) under the dry inert gas atmosphere, it is possible to fully dehydrate this material (Fig. S6), which stays in contradiction to the discussed DSC curve. However, there are two technical reasons for this discrepancy. First, the sweeping rate was high for the DSC studies while the dehydration for the structural studies was performed over the time of at least 2 hours at 300 K (27 °C) under the dry inert gas atmosphere. Second, the DSC studies were performed on the powder sample closed in the Al pan with only the pierced lid which enables dehydration but makes the removal of solvent molecules of crystallization much slower than for the other experiments (e.g., for the structural studies where a single crystal is placed on the pin directly in the flow of dry inert gas).

Therefore, to get a better insight into the temperature range for the dehydration of **1**, we performed the third part of the DSC studies which consisted of three separate cycles of heating and cooling in the (–100)–100 °C range with two other, slower, sweeping rates of 2 and 5 °C·min^{-1} (Fig. S59c). Similarly to the DSC curve at the sweeping rate of 10 °C·min^{-1}, upon the first heating, the positive DSC peaks were found. However, upon the decrease of the sweeping rate, these DSC peaks become weaker but sharper (e.g., appear in the narrower temperature range). Moreover, they are gradually shifted towards lower temperatures. As a result, for the slowest sweeping rate 2 °C·min^{-1}, the DSC peak is observed in the 10–60 °C range. Thus, it can be concluded that the full dehydration of **1** can be thermally achieved by heating the sample to 60 °C in the dry inert gas atmosphere with the sweeping rate of 2 °C·min^{-1}. Taking into account the specific character and technical details of the DSC measurements (mentioned above), it is expected that full thermal dehydration should be achieved at even lower temperatures by the slowdown of the heating rate. Alternatively, complete dehydration can be achieved at constant temperature by a longer exposition of the sample of **1** to the dry inert gas atmosphere as indicated by the structural studies (dehydration at 300 K, i.e., 27 °C, see Fig. S6 and the Structural studies section of the article) and the DVS measurements (dehydration at 298 K, i.e., 25 °C, see Fig. 4 and the related text in the article).

Table S31 Crystal data and structure refinement parameters for Zn(II)-containing reference compounds **3** and **4** (see Comment on Page S121 for details).

Compound	**3**	**4**
Formula	$C_{52}H_{48}Cl_4O_5P_4Zn_2$	$C_{78}H_{74}Cl_4Mn_1O_7P_6Zn_1$
M_W / g·mol^{-1}	1149.36	1571.33
T / K	100(2)	
λ / Å	0.71073 (Mo Kα)	
Crystal system	trigonal	monoclinic
Space group	$R\bar{3}$	*Cc*
a / Å	26.4176(14)	11.2591(5)
b / Å	26.4176(14)	26.7307(12)
c / Å	20.5907(17)	25.1411(11)
α / °	90	90
β / °	90	100.5200(10)
γ / °	120	90
V / Å^3	12444.8(17)	7439.4(6)
Z	9	4
Density / g·cm^{-1}	1.380	1.403
Absorption coefficient / cm^{-1}	1.219	0.818
F(000)	5292	3244
Θ range / °	2.555–26.393	2.361–25.35
Limiting indices	−32 < h < 33<br>−32 < k < 33<br>−25 < l < 25	−13 < h < 13<br>−32 < k < 32<br>−30 < l < 30
Collected reflections	66417	44000
R_{int}	0.0766	0.0378
Completeness	0.999	0.999
Flack parameter	-	0.055(5)
Data/restraints/parameters	5659/6/310	13591/11/880
GOF on F^2	1.220	1.140
R_1 (for [$I > 2\sigma(I)$]);<br>wR_2 (for all data)	R_1 = 0.0757<br>wR_2 = 0.1681	R_1 = 0.0428<br>wR_2 = 0.0870
Largest diff. peak and hole /e·Å^{-3}	2.034/−0.697	0.613/−0.426

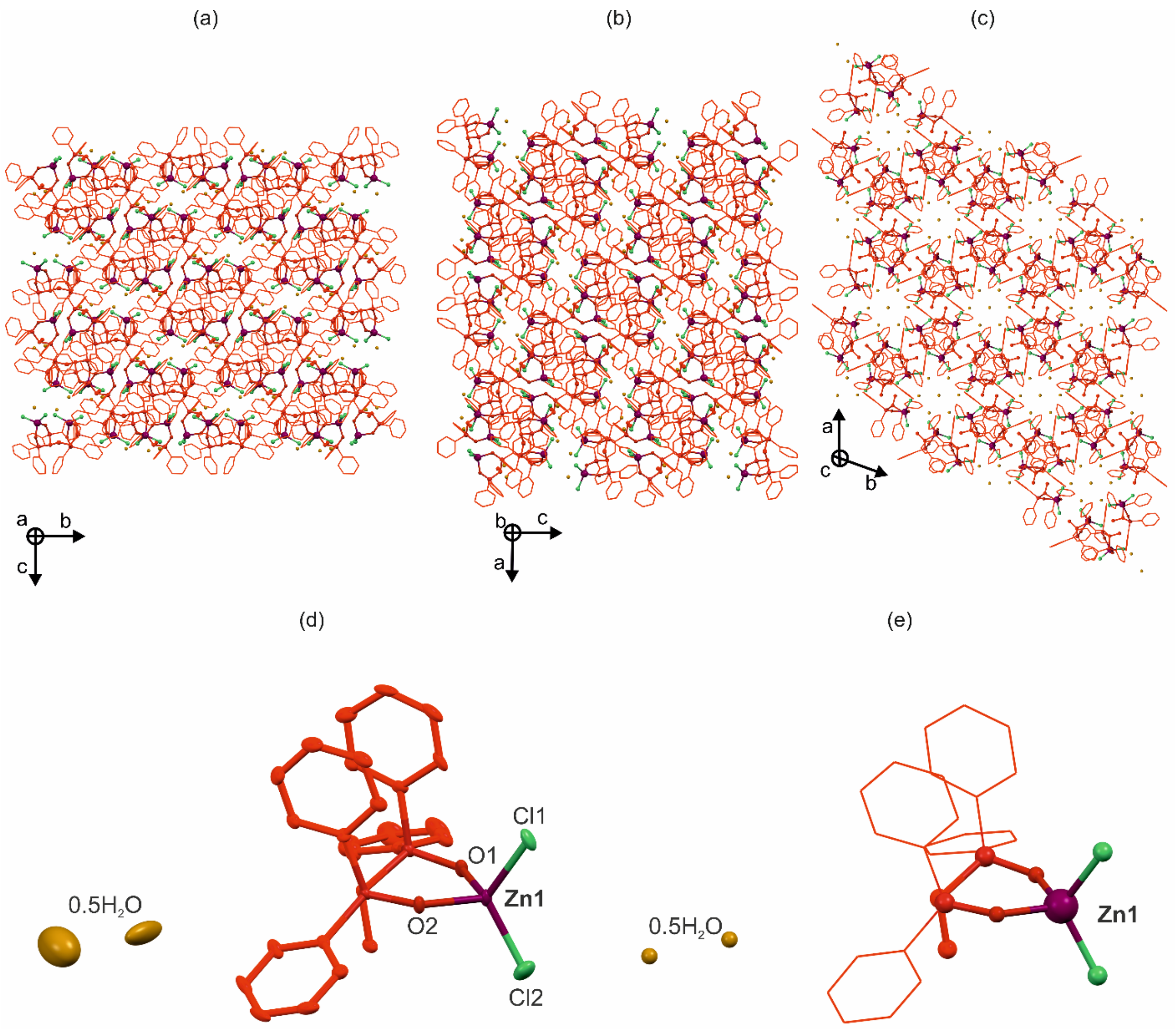

Fig. S60 The representative views of the crystal structure of Zn(II)-containing reference compound **3** along the main **a**, **b**, and **c** crystallographic axes (a–c, respectively), the asymmetric unit with the atoms shown with their thermal ellipsoids as well as the labeling scheme for selected symmetrically independent atoms (d), and the molecular building unit of the metal complex with the underlined coordination environment around the Zn(II) center (e). Thermal ellipsoids in (d) are presented at the 50% probability level. Hydrogen atoms were omitted for clarity. Colors: red with various hues = **Me-dppmO$_2$** ligand, purple = Zn(II), green = chlorido ligands, yellow = water molecules of crystallization (partial occupancies leading to the 0.5 H_2O per a single Zn(II) complex, see Comment on Page S121 for details).

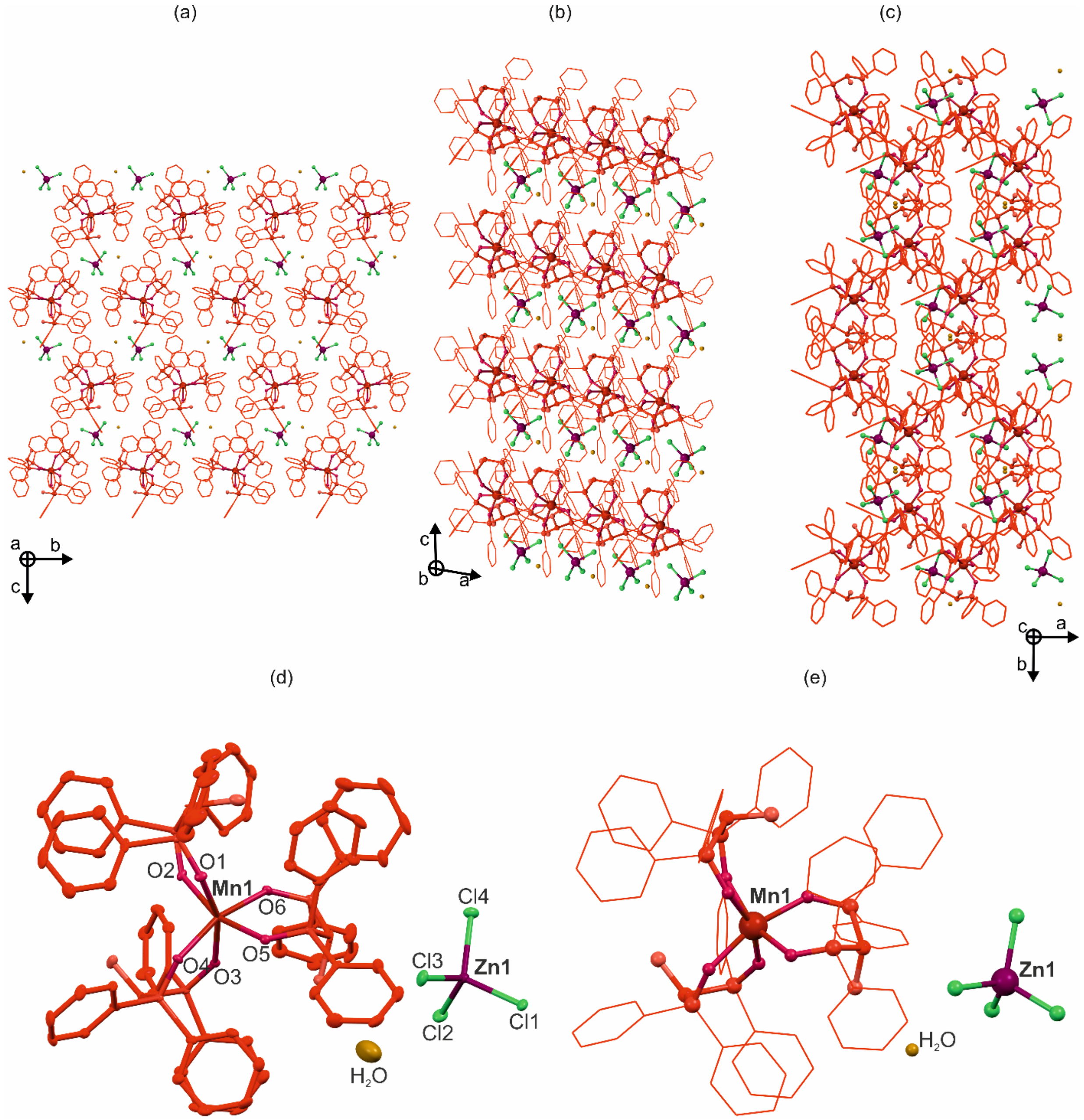

Fig. S61 The representative views of the crystal structure of Zn(II)-containing reference compound **4** (the structural analog of **1**, see Fig. S62 and Comment on Page S121 for details) along the main **a**, **b**, and **c** crystallographic axes (a−c, respectively), the asymmetric unit with the atoms shown with their thermal ellipsoids as well as the labeling scheme for selected symmetrically independent atoms (d), and the molecular building unit of the metal complexes with the underlined coordination environment around Mn(II) centers, including the polar arrangement around the Mn1 one (e). Thermal ellipsoids in (d) are presented at the 50% probability level. Hydrogen atoms were omitted for clarity. Colors: red with various hues = Mn1 complexes with **Me-dppmO$_2$** ligands attached, purple = Zn1, green = chlorido ligands, yellow = water molecules of crystallization.

Table S32 Selected detailed structure parameters of Zn(II)-containing reference compound **4** (the structural analog of **1**, see Fig. S62 and Comment on Page S121 for details, as well as Table S2 for the comparison of detailed structure parameters with $\mathbf{1}^{100K}$).

Selected bond lengths in **4** / Å					
Mn1-O1	2.223(4)	Mn1-O4	2.164(4)	Zn1-Cl1	2.2781(15)
Mn1-O2	2.165(4)	Mn1-O5	2.194(4)	Zn1-Cl2	2.2660(16)
Mn1-O3	2.218(4)	Mn1-O6	2.198(4)	Zn1-Cl3	2.2778(16)
				Zn1-Cl4	2.2794(16)
Selected angles between bonds in **4** / °					
O1-Mn1-O2	82.36(14)	O2-Mn1-O5	87.74(14)	O5-Mn1-O6	84.90(14)
O1-Mn1-O3	90.76(13)	O2-Mn1-O6	109.01(14)	Cl1-Zn1-Cl2	108.60(6)
O1-Mn1-O4	108.68(14)	O3-Mn1-O4	81.15(14)	Cl1-Zn1-Cl3	105.46(6)
O1-Mn1-O5	160.31(14)	O3-Mn1-O5	103.42(13)	Cl1-Zn1-Cl4	111.41(6)
O1-Mn1-O6	82.32(14)	O3-Mn1-O6	86.68(14)	Cl2-Zn1-C3	113.75(7)
O2-Mn1-O3	161.70(15)	O4-Mn1-O5	87.24(14)	Cl2-Zn1-Cl4	110.94(6)
O2-Mn1-O4	85.02(14)	O4-Mn1-O6	163.58(14)	Cl3-Zn1-Cl4	106.58(6)

Comment to Fig. S60–S62, as well as Tables S31 and S32:

Exploring the possibility of synthesizing the structural analog of **1** containing only the octahedral Mn(II) complexes (i.e., of the Mn1-type as detected in compound **1**) and the diamagnetic Zn(II)-based tetrahedral complexes (i.e., of the Mn2-type as detected in compound **1**), which could be used for the further discussion of the origin of slow magnetic relaxation effects in **1** (assigned to the Mn1 centers based on the structural and magnetic studies, as well as the comparison with the literature reports, see the Magnetic properties and their modulation with humidity changes section of the main article), we first examined the synthetic pathway for the preparation of the Zn(II) analog of **1** containing exclusively the Zn(II) complexes (both octahedral and tetrahedral, analogous to Mn1 and Mn2 sites, respectively). To achieve this, we adapted the synthetic procedure for **1** using $Zn^{II}Cl_2$ instead of $Mn^{II}Cl_2 \cdot 4H_2O$; however, instead of obtaining the structural analog of **1**, we obtained very different compound **3** consisting of neutral tetrahedral Zn(II) complexes (Table S31 as well as Fig. S60 and S62; see also the synthetic details below). It is not very surprising since Zn(II) centers can adopt the tetrahedral geometry (related to the coordination number of 4) easier than the octahedral geometry (related to the coordination number of 6),[S26–S28] while the Mn(II) centers of both coordination geometries are generally easily generated.[S29] This indicates that the formation of octahedral $[Zn^{II}(Me\text{-}dppmO_2)_3]^{2+}$ complexes, i.e., the analogs of Mn1 sites in **1**, is not favored under the applied synthetic conditions. Taking advantage of this observation, we attempted to synthesize the mixed-metal Mn(II)–Zn(II) analog of compound **1**. Thus, we modified the synthetic conditions for **1** using the 1:1 molar mixture of $Mn^{II}Cl_2 \cdot 4H_2O$, and $Zn^{II}Cl_2$ instead of only $Mn^{II}Cl_2 \cdot 4H_2O$. As a result, we obtained a new compound, named **4**, which is isostructural to **1**, for which the crystal structure could be successfully solved and refined with the Mn(II) centers occupying the octahedral sites, i.e., forming the $[Mn^{II}(Me\text{-}dppmO_2)_3]^{2+}$ complexes, while the Zn(II) centers occupying the tetrahedral sites, i.e., forming the $[Zn^{II}Cl_4]^{2-}$ complexes (Table S31, as well as Fig. S61 and S62; see also the synthetic details below). This assignment was supported by the structural data, i.e. metal-ligand bond lengths (Table S32). Comparison of the Mn-O distances in Mn1 sites in $\mathbf{1}^{\mathbf{100K}}$ (Table S2) and analogous distances in Mn1 sites in **4** (Table S32) indicates also identical related bond lengths in both compounds which suggest that the Mn(II) centers fully (or almost fully) occupy the octahedral positions in compound **4**. On the other hand, the metal-chlorido distances of the second complex (i.e., a $[M^{II}Cl_4]^{2-}$ type) are distinctly shorter, i.e., 2.2660–2.2794 Å, in **4** than in $\mathbf{1}^{\mathbf{100K}}$, where they cover the range of 2.3574–2.3689 Å (Tables S2 and S32). This suggests that the Zn(II) centers dominate the tetrahedral positions in compound **4**; thus, its formula of $[Mn^{II}(Me\text{-}dppmO_2)_3][Zn^{II}Cl_4] \cdot H_2O$ (**4**) can be postulated. The interpretation of the presence of the close-to-1:1 Mn:Zn molar ratio in **4** was also supported by the *dc* magnetic studies showing the expected value of the magnetic susceptibility–temperature product at room temperature (see Comment to Fig. S63–S65 and Table S33 below).

Synthesis of 3

The **Me-dppmO₂** precursor (50.0 mg, 0.116 mmol 3.0 eq.) and $Zn^{II}Cl_2$ (10.5 mg, 0.077 mmol, 2.0 eq.) were dissolved together in a mixture of MeCN (1 mL) and MeOH (2 mL) and layered with Et_2O. After a few days, colorless crystals of **3** were filtered and washed with Et_2O. Yield: 38%. The composition of **3**, $[Zn^{II}(Me\text{-}dppmO_2)Cl_2] \cdot 0.5\ H_2O$ (M_W = 575.72 g·mol^{-1}), was determined by the SC-XRD experiment (Fig. S60 and Table S31) and while the phase purity and its air stability were examined by the P-XRD method (Fig. S62 and the related additional comment below).

Synthesis of 4

The **Me-dppmO₂** precursor (100.0 mg, 0.232 mmol 3.0 eq.) was dissolved in 2 mL MeOH, and 2 mL MeCN, then the 1:1 mixture of $Mn^{II}Cl_2 \cdot 4H_2O$ (15.3 mg, 0.077 mmol, 1.0 eq.) and $Zn^{II}Cl_2$ (10.5 mg, 0.077 mmol, 1.0 eq.) in 0.5 mL MeOH was added, and mixed for 2 h. The resulting mixture was layered with Et_2O and left for a few days for crystallization. It results in the colorless crystals of **4** which were filtered and washed with Et_2O. Yield: 45%. The composition of **4**, $[Mn^{II}(Me\text{-}dppmO_2)_3][Zn^{II}Cl_4] \cdot H_2O$ (M_W = 1571.33 g·mol^{-1}), was determined by the SC-XRD experiment (Fig. S61 and Table S31, supported also by the *dc* magnetic studies, see Comment to Fig. S63–S65 and Table S33 below), while the phase purity and its air stability were proven by the P-XRD method (Fig. S62).

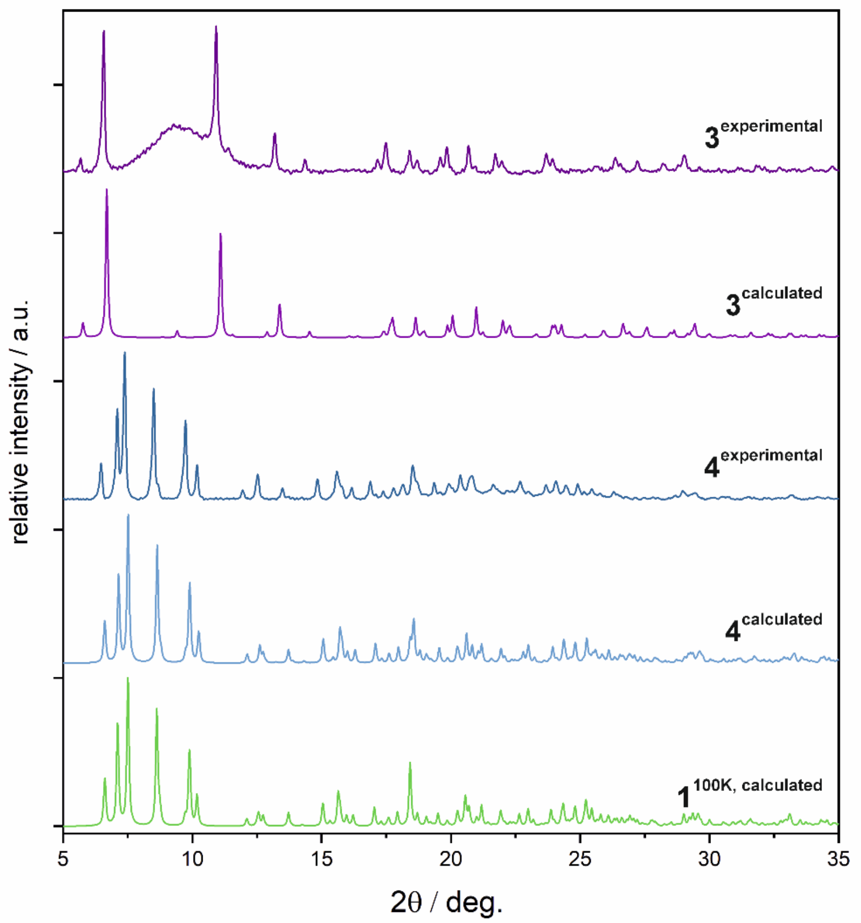

Fig. S62 Comparison of experimental (all experiments performed at room temperature) and calculated powder X-ray diffraction (P-XRD) patterns of compounds **3** and **4**. The calculated P-XRD patterns were based on the structural models measured at 100(2) K, see Table S31 as well as Fig. S60 and S61. For additional comparison, the calculated P-XRD pattern based on the structural model of $\mathbf{1}^{\mathbf{100K}}$ (the hydrated phase, measured at 100(2) K, Table S1 and Fig. 1; abbreviated here as $\mathbf{1}^{\mathbf{100K,\ calculated}}$) was added.

Additional comment to Fig. S62:

For the measured P-XRD pattern for **3**, in general, all sharp P-XRD peaks from the experimental data match the calculated ones proving the phase identity of the powder sample with the single crystal used for the SC-XRD analyses (Fig. S62). However, the additional broad peak at ca. 10° of the 2Θ angle suggests the impurity of the amorphous character. It probably comes from the excess of the organic ligand used in the synthesis. We did not attempt to further optimize the synthesis to avoid the appearance of this amorphous impurity as compound **3** was only prepared to examine the possibility of obtaining the only-Zn(II)-containing analog of **1** which was found to be impossible as the observed compound **3** reveals a very different crystal structure containing only neutral tetrahedral Zn(II) complexes. The much more important compound **4** of the mixed-metal Zn(II)-Mn(II) composition, which was used as the additional reference material for magnetic characterization, was found to be pure (i.e., without any amorphous or crystalline impurities, as depicted by the P-XRD pattern also shown in Fig. S62.

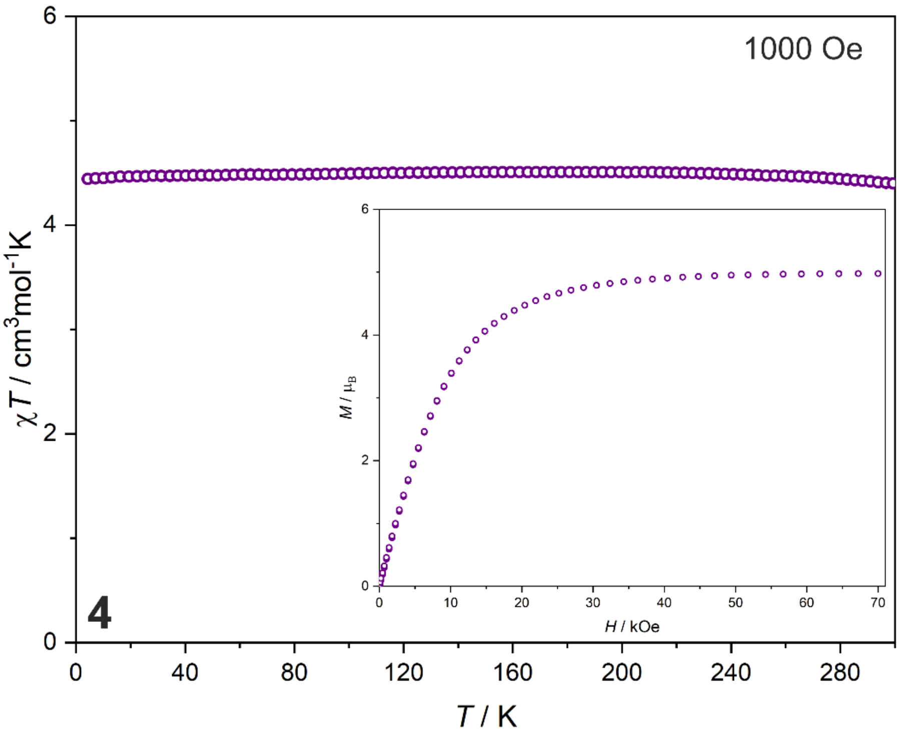

Fig. S63 Direct-current (*dc*) magnetic characteristics of Zn(II)-containing reference compound **4** (the powder sample packed at 28% of RH), including the temperature dependence of the $\chi_M T$ product under the indicated external magnetic field (the main part) and the field dependence of molar magnetization (*M*) collected at 1.8 K (the inset).

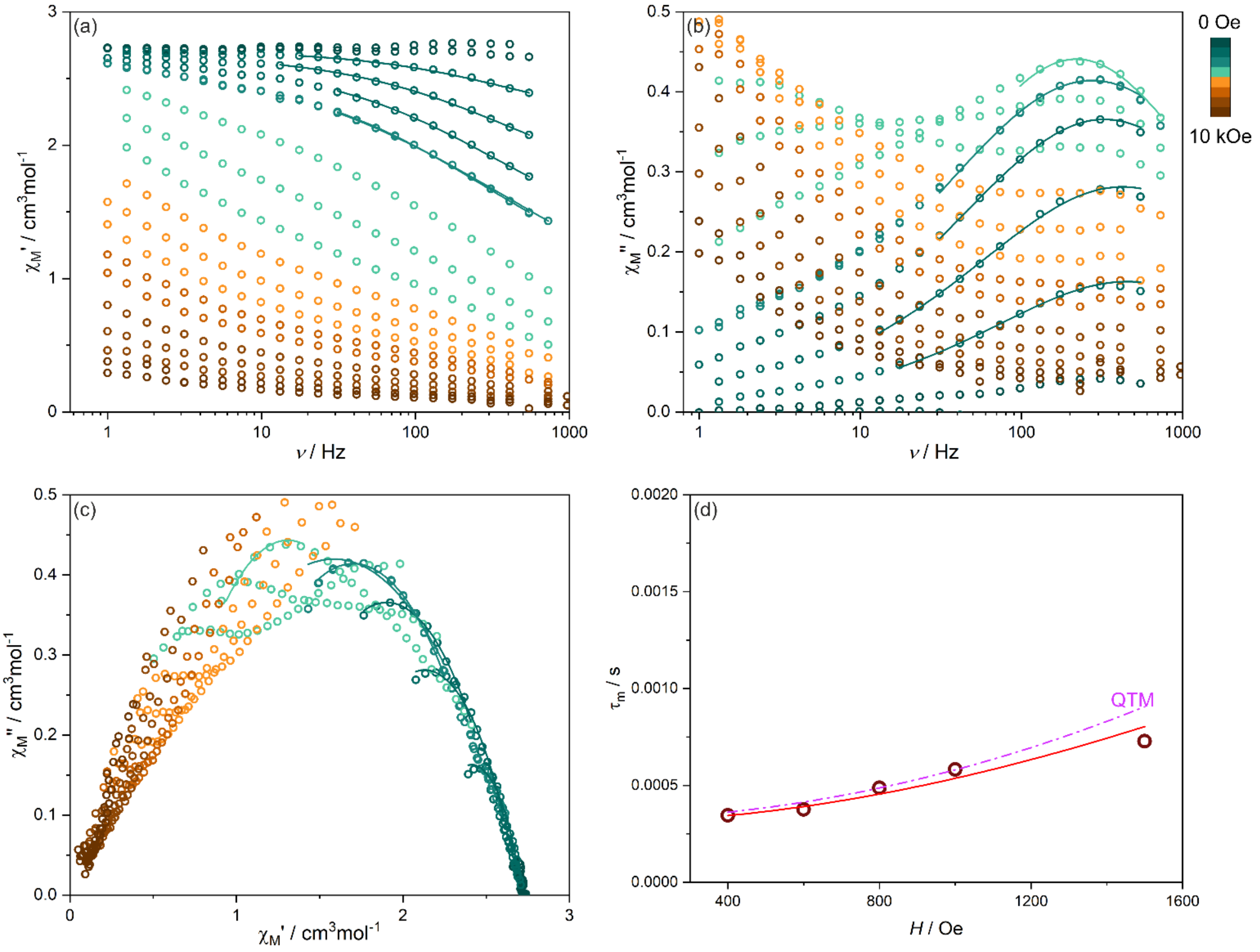

Fig. S64 Magnetic-field-variable alternate-current (*ac*) magnetic characteristics of Zn(II)-containing reference compound **4** (the powder sample packed at 28% of RH) at T = 1.8 K, including the frequency dependences of the in-phase susceptibility, χ_M', (a) and the out-of-phase susceptibility, χ_M'', (b) under variable indicated *dc* fields, the related Argand plots (c), and the field dependence of resulting magnetic relaxation times, τ_m (d). Colored solid lines in (a–c) represent the best fits using the generalized Debye model for a single relaxation process. The red solid line in (d) shows the best fit taking into account indicated relaxation processes (the result of the simultaneous fit with the temperature-dependent relaxation times shown in Fig. S65) while the dashed colored lines represent the respective course of individual magnetic relaxation processes. Best-fit parameters for the curve presented in the (d) part are gathered in Table S33.

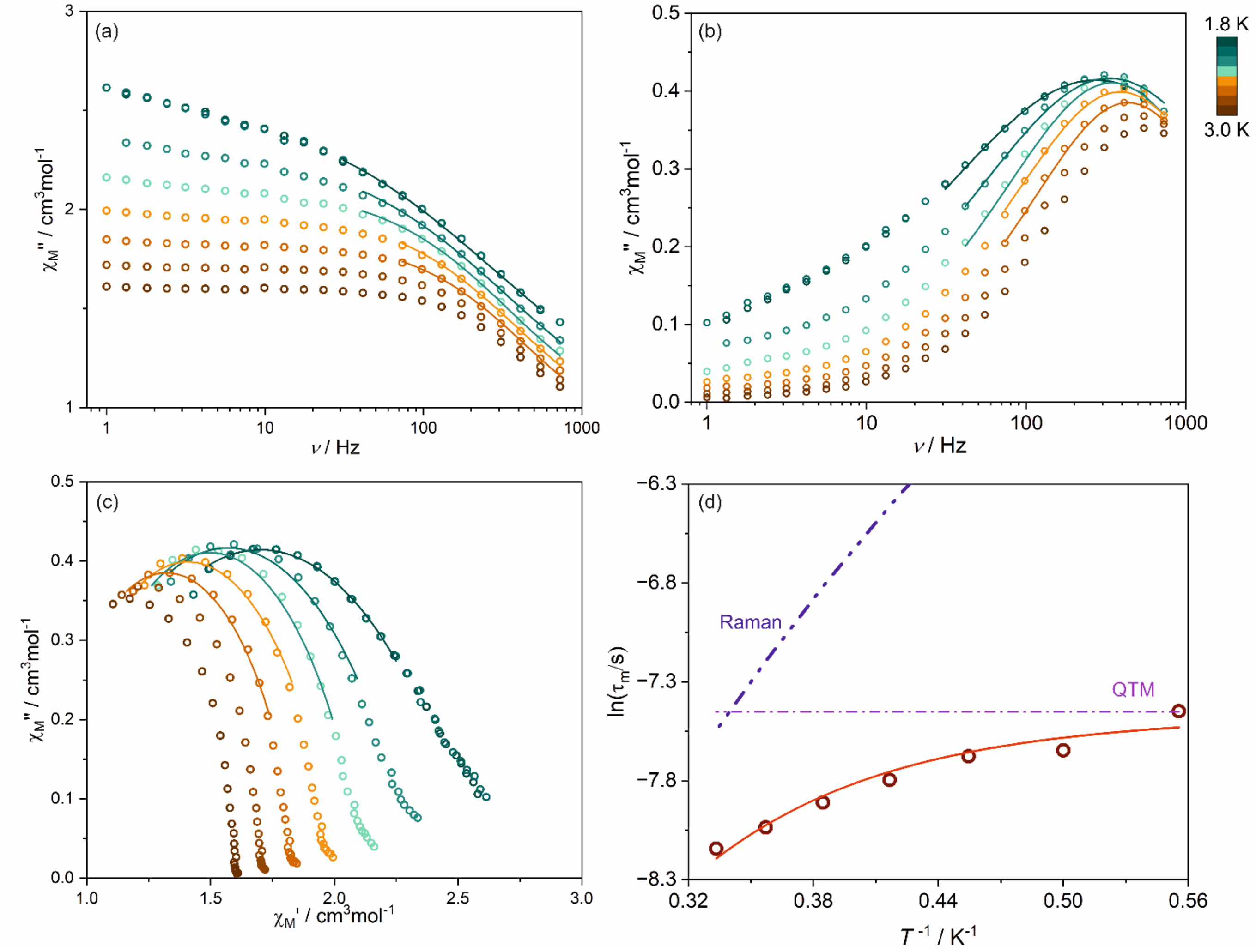

Fig. S65 Temperature-variable alternate-current (*ac*) magnetic characteristics of Zn(II)-containing reference compound **4** (the powder sample packed at 28% of RH) at H_{dc} = 1000 Oe, including the frequency dependences of the in-phase susceptibility, χ_M', (a) and the out-of-phase susceptibility, χ_M'', (b) under variable indicated *dc* fields, the related Argand plots (c), and the field dependence of resulting magnetic relaxation times, τ_m (d). Colored solid lines in (a–c) represent the best fits using the generalized Debye model for a single relaxation process. The red solid line in (d) shows the best fit taking into account indicated relaxation processes (the result of the simultaneous fit with the magnetic-field-dependent relaxation times shown in Fig. S64) while the dashed colored lines represent the respective course of individual magnetic relaxation processes. Best-fit parameters for the curve presented in the (d) part are gathered in Table S33.

Table S33 Summary of the best-fit slow magnetic relaxation parameters for Zn(II)-containing reference compound **4** obtained within the three-dimensional simultaneous fitting of the field dependence at T = 1.8 K and the temperature dependence at H_{dc} =1000 Oe of magnetic relaxation times (Fig. S64 and S65). For the related equation see the main text, i.e., equation (4).

Compound	a / s^{-1}	b / Oe^{-2}	c / Oe^{-2}	B_{Raman} / s^{-1} K^{-n}	n
**4** (28% RH)	3123.43	$8.13 \cdot 10^{-7}$	0 (fixed)	7.11	5.08

Comment to Fig. S63–S65 and Table S33:

The mixed-metal Mn(II)-Zn(II) reference compound **4** of the proposed composition of $[Mn^{II}(Me\text{-}dppmO_2)_3][Zn^{II}Cl_4]\cdot H_2O$ (see Comment on Page S121) was investigated from the viewpoint of its *dc* and *ac* magnetic properties (Fig. S63–S65). For this compound, the room-temperature $\chi_M T$ value reaches 4.45 cm^3 mol^{-1} K which is close to the theoretical value of 4.375 cm^3 mol^{-1} K calculated for a single Mn(II) center (S = 5/2, g = 2.0; Fig. S63). Moreover, the molar magnetization, measured with the increasing magnetic field at T = 1.8 K (Fig. S63, the inset), reaches the value of 4.97 μ_B at 70 kOe which also corresponds well to the value of 5 μ_B expected for a single uncoupled Mn(II) center. These results strongly support the presence of the 1:1 Mn-to-Zn molar ratio in compound **4** agreeing with the proposed formula. We also examined the *ac* magnetic characteristics for the powder sample of **4** (stabilized at the selected representative RH of 28%) which indicates the presence of a slow magnetic relaxation effect for this compound (Fig. S64 and S65, Table S33). The magnetic relaxation curves were found to be similar to those presented for **1** at similar humidity conditions (23% of RH, Fig. 10). Similarly to **1**, the combination of Raman and QTM processes was found to govern this magnetic relaxation (Table S33). Despite that the detailed comparison between these two compounds can be difficult due to the presence of diamagnetic tetrahedral Zn(II) complexes in **4** instead of paramagnetic tetrahedral Mn(II) complexes in **1**, one can now strongly conclude that the magnetic relaxation effect originates from the octahedral $[Mn^{II}(Me\text{-}dppmO_2)_3]^{2+}$ complexes as they are present in both compounds, **1** and **4** (see the whole discussion on the magnetic properties of **1** in the main article).

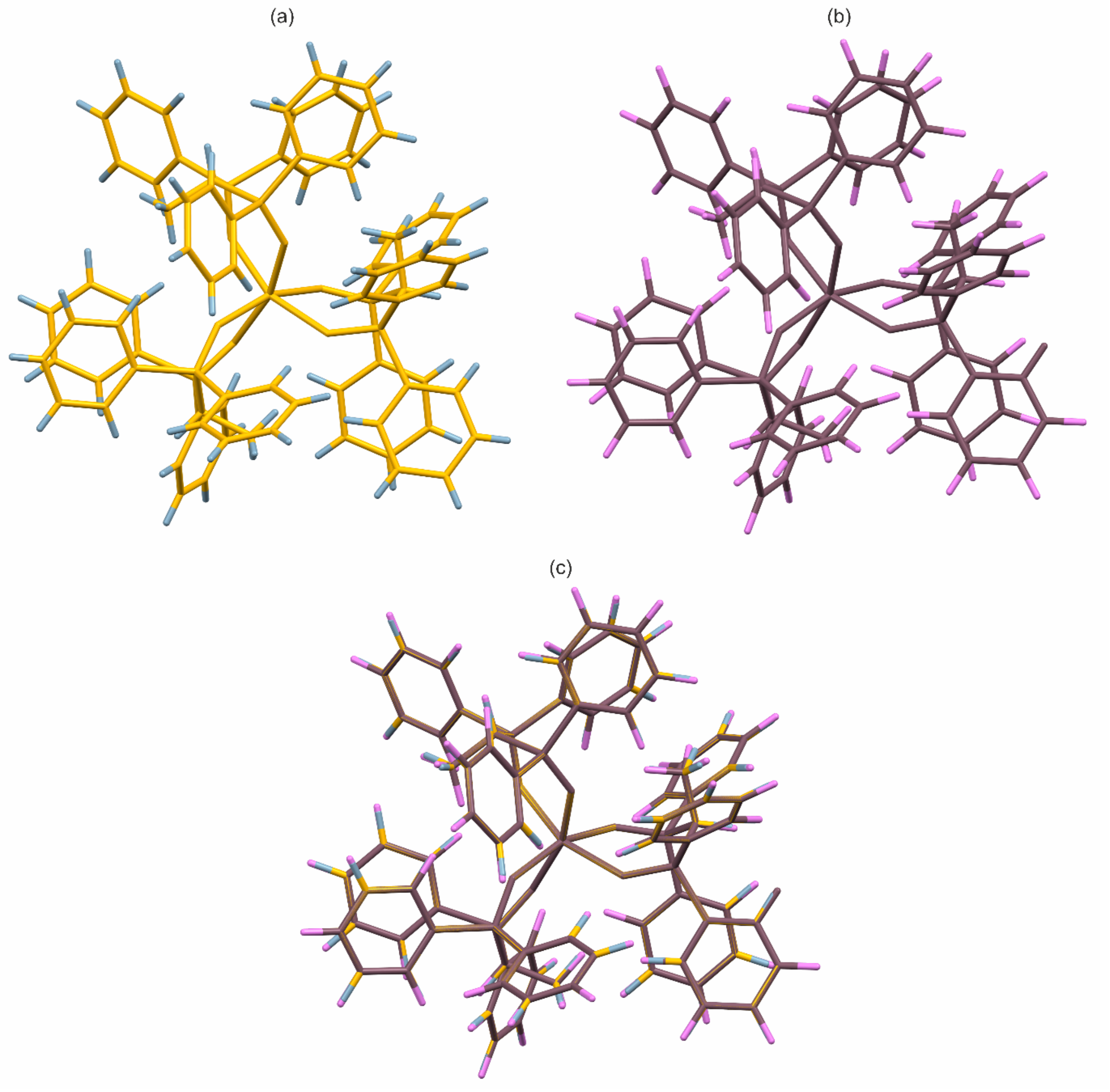

Fig. S66 Comparison of the structural model of Mn1 complexes (i.e., the cationic part of **1**) obtained from the experimental SC–XRD analysis (**1**$^{\mathbf{100K}}$, non-H-atoms colored in orange, while hydrogen atoms colored with contrasting blue color for clarity) (a), and the structural model of the same complexes, named Mn1^{opt}, with the optimized hydrogen atoms at ZORA-B3LYP level of theory using "Tight" convergence criteria for wavefunction and gradients (non-H-atoms colored in brown, while hydrogen atoms colored with contrasting violet color for clarity) (b), as well as the overlay of both structural models (c). Numerical comparison of C–H bonds is gathered in Table S34 (see below).

Table S34 Comparison between carbon-hydrogen bond lengths of the Mn1 complexes (cationic part of 1), obtained from the SC-XRD experiment (columns named SC-XRD), and the Mn1^{opt} model of the same complex with the hydrogen atoms optimized with the ZORA-B3LYP level of theory using "Tight" convergence criteria for wavefunction and gradients (Fig. S66 and Comment on Page S54).

Carbon-hydrogen bond distances of Mn1 complexes / Å					
	SC-XRD	Optimized		SC-XRD	Optimized
C1–H1	0.981	1.0984	C39–H39	0.932	1.0895
C2–H2A	0.959	1.0923	C40–H40	0.931	1.0880
C2–H2B	0.959	1.0926	C42–H42	0.929	1.0878
C2–H2C	0.961	1.0922	C43–H43	0.928	1.0890
C4–H4	0.930	1.0840	C44–H44	0.929	1.0895
C5–H5	0.928	1.0892	C45–H45	0.929	1.0889
C6–H6	0.930	1.0892	C46–H46	0.929	1.0892
C7–H7	0.932	1.0895	C48–H48	0.929	1.0890
C8–H8	0.929	1.0901	C49–H49	0.931	1.0898
C10–H10	0.929	1.0900	C50–H50	0.929	1.0890
C11–H11	0.931	1.0896	C51–H51	0.931	1.0894
C12–H12	0.931	1.0890	C52–H52	0.931	1.0879
C13–H13	0.930	1.0890	C53–H53	0.981	1.0975
C14–H14	0.931	1.0839	C54–H54A	0.960	1.0934
C16–H16	0.929	1.0881	C54–H54B	0.959	1.0949
C17–H17	0.932	1.0899	C54–H54C	0.959	1.0926
C18–H18	0.928	1.0894	C56–H56	0.931	1.0886
C19–H19	0.930	1.0890	C57–H57	0.928	1.0870
C20–H20	0.930	1.0901	C58–H58	0.928	1.0898
C22–H22	0.930	1.0879	C59–H59	0.931	1.0896
C23–H23	0.930	1.0888	C60–H60	0.930	1.0859
C24–H24	0.928	1.0890	C62–H62	0.930	1.0904
C25–H25	0.930	1.0894	C63–H63	0.928	1.0891
C26–H26	0.930	1.0901	C64–H64	0.930	1.0894
C27–H27	0.980	1.0971	C65–H65	0.929	1.0893
C28–H28A	0.960	1.0946	C66–H66	0.928	1.0885
C28–H28B	0.961	1.0954	C68–H68	0.930	1.0898
C28–H28C	0.957	1.0926	C69–H69	0.930	1.0887
C30–H30	0.930	1.0897	C70–H70	0.930	1.0907
C31–H31	0.932	1.0896	C71–H71	0.930	1.0884
C32–H32	0.928	1.0891	C72–H72	0.930	1.0839
C33–H33	0.930	1.0897	C74–H74	0.931	1.0844
C34–H34	0.930	1.0857	C75–H75	0.930	1.0888
C36–H36	0.930	1.0869	C76–H76	0.929	1.0895
C37–H37	0.930	1.0892	C77–H77	0.930	1.0895
C38–H38	0.931	1.0902	C78–H78	0.932	1.0886

Table S35 (part 1/3) Comparison of the energies of excited states (corresponding to optical transition energies from the ground sextet state, *ΔE*, Fig. S35) obtained from CASSCF-type *ab initio* calculations for $Mn1^{opt}$ and Mn1 models of octahedral Mn(II) complexes of **1** (Figure 1 and S66) when taking into account the quartet and doublet excited states, shown together with the resulting wavelengths of optical transitions and the values of energies and wavelengths after the scaling procedure (energy shift for scaling set at –8000 cm^{-1}, see the Comment on theoretical calculations of optical transitions in Mn(II) complexes in **1** above).

**$Mn1^{opt}$** complexes					**Mn1** complexes				
State	*ΔE* / cm^{-1}	Wavelength / nm	Scaled *ΔE* / cm^{-1}	Scaled wavelength / nm	State	*ΔE* / cm^{-1}	Wavelength / nm	Scaled *ΔE* / cm^{-1}	Scaled wavelength / nm
1	27731.6	360.59946	19731.6	506.80127	1	27743.7	360.44219	19743.7	506.49068
2	27751.2	360.34478	19751.2	506.29835	2	27758.7	360.24742	19758.7	506.10617
3	28120.9	355.6074	20120.9	496.99566	3	28129.8	355.49488	20129.8	496.77592
4	30189.8	331.2377	22189.8	450.65751	4	30193.3	331.19931	22193.3	450.58644
5	30434.9	328.57016	22434.9	445.7341	5	30441.2	328.50216	22441.2	445.60897
6	30499.4	327.8753	22499.4	444.4563	6	30501.2	327.85595	22501.2	444.42074
7	31103.2	321.51033	23103.2	432.84047	7	31106.5	321.47622	23106.5	432.77866
8	31109.4	321.44625	23109.4	432.72435	8	31110.7	321.43282	23110.7	432.7
9	31183	320.68755	23183	431.35056	9	31184.7	320.67007	23184.7	431.31893
10	37299.1	268.10298	29299.1	341.30741	10	37304.7	268.06274	29304.7	341.24219
11	37375	267.55853	29375	340.42553	11	37378	267.53705	29378	340.39077
12	37772.3	264.74427	29772.3	335.88268	12	37775.8	264.71974	29775.8	335.8432
13	38307.3	261.04685	30307.3	329.95351	13	38297.1	261.11638	30297.1	330.06459
14	38522.8	259.58653	30522.8	327.62394	14	38519.8	259.60675	30519.8	327.65614
15	38827.3	257.55074	30827.3	324.3878	15	38828	257.5461	30828	324.38043
16	38966.3	256.63201	30966.3	322.9317	16	38985	256.50891	30985	322.73681
17	39066.6	255.97313	31066.6	321.8891	17	39081.6	255.87489	31081.6	321.73376
18	39477.1	253.31141	31477.1	317.69127	18	39476.1	253.31783	31476.1	317.70137
19	39605.2	252.4921	31605.2	316.40363	19	39612.2	252.44748	31612.2	316.33357
20	39939.3	250.37995	31939.3	313.0939	20	39955.8	250.27656	31955.8	312.93224
21	41546.4	240.69474	33546.4	298.09458	21	41555.5	240.64203	33555.5	298.01374
22	41921.1	238.54336	33921.1	294.80176	22	41932.3	238.47964	33932.3	294.70446
23	41949	238.38471	33949	294.55949	23	41956.4	238.34266	33956.4	294.49529
24	42293.5	236.44295	34293.5	291.60045	24	42300.9	236.40159	34300.9	291.53754
25	42311.5	236.34237	34311.5	291.44747	25	42320.6	236.29155	34320.6	291.3702
26	42694.4	234.22276	34694.4	288.23095	26	42703.2	234.17449	34703.2	288.15786
27	42828.1	233.49156	34828.1	287.12448	27	42837	233.44305	34837	287.05112
28	43499.8	229.88611	35499.8	281.69173	28	43506.5	229.85071	35506.5	281.63857
29	43542.8	229.65909	35542.8	281.35093	29	43547.8	229.63273	35547.8	281.31136
30	44339	225.53508	36339	275.18644	30	44341.1	225.5244	36341.1	275.17054
31	47164.2	212.02522	39164.2	255.33523	31	47171.6	211.99196	39171.6	255.28699
32	47205.1	211.84152	39205.1	255.06886	32	47208.6	211.82581	39208.6	255.04609
33	47485.5	210.5906	39485.5	253.25752	33	47490.5	210.56843	39490.5	253.22546
34	48185.4	207.53174	40185.4	248.8466	34	48192.6	207.50074	40192.6	248.80202
35	48331.5	206.9044	40331.5	247.94515	35	48335.8	206.88599	40335.8	247.91872
36	50176	199.29847	42176	237.10167	36	50186.9	199.25518	42186.9	237.04041

Table S35 (part 2/3) Comparison of the energies of excited states (corresponding to optical transition energies from the ground sextet state, *ΔE*, Fig. S35) obtained from CASSCF-type *ab initio* calculations for $Mn1^{opt}$ and Mn1 models of octahedral Mn(II) complexes of **1** (Figure 1 and S66) when taking into account the quartet and doublet excited states, shown together with the resulting wavelengths of optical transitions and the values of energies and wavelengths after the scaling procedure (energy shift for scaling set at –8000 cm^{-1}, see the Comment on theoretical calculations of optical transitions in Mn(II) complexes in **1** above).

$Mn1^{opt}$ complexes					Mn1 complexes				
State	*ΔE* / cm^{-1}	Wavelength / nm	Scaled *ΔE* / cm^{-1}	Scaled wavelength / nm	State	*ΔE* / cm^{-1}	Wavelength / nm	Scaled *ΔE* / cm^{-1}	Scaled wavelength / nm
37	50376	198.50723	42376	235.98263	37	50386.1	198.46743	42386.1	235.9264
38	50609.6	197.59097	42609.6	234.6889	38	50614.1	197.5734	42614.1	234.66411
39	52095.7	191.95442	44095.7	226.77948	39	52098.3	191.94484	44098.3	226.76611
40	52148.8	191.75897	44148.8	226.50672	40	52156.6	191.73029	44156.6	226.46671
41	52232.9	191.45022	44232.9	226.07607	41	52235.9	191.43922	44235.9	226.06073
42	52241.9	191.41723	44241.9	226.03008	42	52244	191.40954	44244	226.01935
43	52549.6	190.29641	44549.6	224.46891	43	52551.6	190.28916	44551.6	224.45883
44	52629.2	190.00859	44629.2	224.06855	44	52633.6	189.9927	44633.6	224.04646
45	52908.7	189.00483	44908.7	222.674	45	52902.5	189.02698	44902.5	222.70475
46	53028.1	188.57926	45028.1	222.08354	46	53031.3	188.56788	45031.3	222.06776
47	53883.1	185.58695	45883.1	217.94517	47	53884.8	185.58109	45884.8	217.93709
48	53894.6	185.54735	45894.6	217.89056	48	53897.5	185.53736	45897.5	217.87679
49	54012.2	185.14336	46012.2	217.33366	49	54015.3	185.13273	46015.3	217.31902
50	54058	184.9865	46058	217.11755	50	54071.4	184.94065	46071.4	217.0544
51	54305.4	184.14375	46305.4	215.95753	51	54295.7	184.17665	46295.7	216.00278
52	54366.1	183.93815	46366.1	215.67481	52	54361	183.95541	46361	215.69854
53	54815	182.43182	46815	213.60675	53	54826.1	182.39488	46826.1	213.55612
54	55009.4	181.78711	47009.4	212.72341	54	55013.7	181.7729	47013.7	212.70396
55	55371	180.59995	47371	211.09962	55	55368.9	180.6068	47368.9	211.10898
56	55654.1	179.68128	47654.1	209.84553	56	55655.3	179.67741	47655.3	209.84025
57	55960.3	178.69811	47960.3	208.50578	57	55961.2	178.69524	47961.2	208.50187
58	56270.9	177.71175	48270.9	207.16415	58	56273.7	177.70291	48273.7	207.15213
59	56863	175.86128	48863	204.65383	59	56860	175.87056	48860	204.66639
60	57236.1	174.71491	49236.1	203.10301	60	57236.7	174.71308	49236.7	203.10053
61	57441.1	174.09137	49441.1	202.26087	61	57444.1	174.08228	49444.1	202.2486
62	57683.6	173.3595	49683.6	201.27366	62	57681.5	173.36581	49681.5	201.28217
63	57773.4	173.09004	49773.4	200.91053	63	57768	173.10622	49768	200.93233
64	58023.5	172.34396	50023.5	199.90604	64	58025.2	172.33891	50025.2	199.89925
65	58317.9	171.47394	50317.9	198.73643	65	58319	171.4707	50319	198.73209
66	58396.6	171.24285	50396.6	198.42608	66	58393.6	171.25164	50393.6	198.4379
67	59134.1	169.10716	51134.1	195.56421	67	59128.3	169.12375	51128.3	195.5864
68	59634	167.68957	51634	193.67084	68	59642.3	167.66624	51642.3	193.63971
69	59682.8	167.55246	51682.8	193.48797	69	59687	167.54067	51687	193.47225
70	61109.9	163.63961	53109.9	188.28881	70	61111.3	163.63586	53111.3	188.28385
71	61221.9	163.34024	53221.9	187.89258	71	61218.3	163.34985	53218.3	187.90529
72	61280.5	163.18405	53280.5	187.68593	72	61277.3	163.19257	53277.3	187.6972

Table S35 (part 3/3) Comparison of the energies of excited states (corresponding to optical transition energies from the ground sextet state, *ΔE*, Fig. S35) obtained from CASSCF-type *ab initio* calculations for Mn1^{opt} and Mn1 models of octahedral Mn(II) complexes of **1** (Figure 1 and S66) when taking into account the quartet and doublet excited states, shown together with the resulting wavelengths of optical transitions and the values of energies and wavelengths after the scaling procedure (energy shift for scaling set at –8000 cm^{-1}, see the Comment on theoretical calculations of optical transitions in Mn(II) complexes in **1** above).

**Mn1^{opt}** complexes					**Mn1** complexes				
State	ΔE / cm^{-1}	Wavelength / nm	Scaled ΔE / cm^{-1}	Scaled wavelength / nm	State	ΔE / cm^{-1}	Wavelength / nm	Scaled ΔE / cm^{-1}	Scaled wavelength / nm
73	61687.7	162.10687	53687.7	186.2624	73	61693.8	162.09084	53693.8	186.24124
74	61770.3	161.8901	53770.3	185.97627	74	61771.1	161.888	53771.1	185.97351
75	61993.3	161.30775	53993.3	185.20816	75	61991.6	161.31218	53991.6	185.214
76	62060.9	161.13205	54060.9	184.97657	76	62054.8	161.14789	54054.8	184.99745
77	66290	150.85232	58290	171.55601	77	66294.3	150.84253	58294.3	171.54336
78	74586.1	134.07324	66586.1	150.18149	78	74589.2	134.06767	66589.2	150.1745
79	74611.8	134.02706	66611.8	150.12355	79	74615.6	134.02023	66615.6	150.11499
80	75130	133.10262	67130	148.9647	80	75135.8	133.09235	67135.8	148.95183
81	75272	132.85153	67272	148.65026	81	75279.2	132.83882	67279.2	148.63435
82	75387.5	132.64799	67387.5	148.39547	82	75390.1	132.64341	67390.1	148.38975
83	81503	122.69487	73503	136.04887	83	81513.8	122.67861	73513.8	136.02888
84	81601.3	122.54707	73601.3	135.86717	84	81604.7	122.54196	73604.7	135.86089
85	82136.3	121.74885	74136.3	134.88669	85	82142.8	121.73922	74142.8	134.87486
86	82194.8	121.6622	74194.8	134.78034	86	82197.5	121.6582	74197.5	134.77543
87	82497.3	121.21609	74497.3	134.23305	87	82503.8	121.20654	74503.8	134.22134
88	82807.9	120.76142	74807.9	133.67572	88	82812.1	120.7553	74812.1	133.66822
89	82827.3	120.73314	74827.3	133.64106	89	82828.5	120.73139	74828.5	133.63892
90	82843.3	120.70982	74843.3	133.61249	90	82843	120.71026	74843	133.61303
91	83381.1	119.93126	75381.1	132.65925	91	83382.4	119.92939	75382.4	132.65696
92	100304.7	99.69623	92304.7	108.33685	92	100305.4	99.69553	92305.4	108.33602
93	100433.1	99.56877	92433.1	108.18635	93	100438.3	99.56361	92438.3	108.18027
94	100622.6	99.38125	92622.6	107.96501	94	100627.9	99.37602	92627.9	107.95883
95	108846.1	91.87284	100846.1	99.161	95	108850.4	91.86921	100850.4	99.15677
96	108936.6	91.79651	100936.6	99.07209	96	108953.1	91.78261	100953.1	99.0559
97	108998.2	91.74463	100998.2	99.01167	97	109005.9	91.73815	101005.9	99.00412
98	109058.3	91.69408	101058.3	98.95278	98	109063.7	91.68954	101063.7	98.9475
99	109394.8	91.41202	101394.8	98.62439	99	109401.9	91.40609	101401.9	98.61748

Table S36 (part 1/7) Comparison of the energies (*E*) of ground and excited states (the latter corresponding to optical transition energies from the ground sextet state, Fig. S35) obtained from CASSCF-SOC-type *ab initio* calculations for Mn1^{opt} and Mn1 models of octahedral Mn(II) complexes of **1** (Figure 1 and S66) when taking into account the quartet and doublet excited states, shown together with the resulting wavelengths of optical transitions and the values of energies and wavelengths after the scaling procedure (energy shift for scaling set at –8000 cm^{-1}, see the Comment on theoretical calculations of optical transitions in Mn(II) complexes in **1** above).

**Mn1^{opt}** complexes					**Mn1** complexes				
State	*E* / cm^{-1}	Wavelength / nm	Scaled *E* / cm^{-1}	Scaled wavelength / nm	State	*E* / cm^{-1}	Wavelength / nm	Scaled *E* / cm^{-1}	Scaled wavelength / nm
1	0	-	-8000	-	1	0	-	-8000	-
2	0	-	-8000	-	2	0	-	-8000	-
3	0	-	-8000	-	3	0	-	-8000	-
4	0	-	-8000	-	4	0	-	-8000	-
5	0	-	-8000	-	5	0	-	-8000	-
6	27702.3	360.98086	19702.3	507.55496	6	27712.6	360.84669	19712.6	27712.6
7	27702.3	360.98086	19702.3	507.55496	7	27712.6	360.84669	19712.6	27712.6
8	27721.4	360.73214	19721.4	507.06339	8	27732.6	360.58646	19732.6	27732.6
9	27721.4	360.73214	19721.4	507.06339	9	27732.6	360.58646	19732.6	27732.6
10	27747.9	360.38763	19747.9	506.38296	10	27756.2	360.27987	19756.2	27756.2
11	27747.9	360.38763	19747.9	506.38296	11	27756.2	360.27987	19756.2	27756.2
12	27765.8	360.1553	19765.8	505.92437	12	27775.1	360.03471	19775.1	27775.1
13	27765.8	360.1553	19765.8	505.92437	13	27775.1	360.03471	19775.1	27775.1
14	28116.1	355.6681	20116.1	497.11425	14	28124.9	355.55682	20124.9	28124.9
15	28116.1	355.6681	20116.1	497.11425	15	28124.9	355.55682	20124.9	28124.9
16	28117.6	355.64913	20117.6	497.07719	16	28126.4	355.53786	20126.4	28126.4
17	28117.6	355.64913	20117.6	497.07719	17	28126.4	355.53786	20126.4	28126.4
18	30169.4	331.46168	22169.4	451.0722	18	30173.1	331.42103	22173.1	30173.1
19	30169.4	331.46168	22169.4	451.0722	19	30173.1	331.42103	22173.1	30173.1
20	30182.3	331.32001	22182.3	450.80988	20	30185.8	331.2816	22185.8	30185.8
21	30182.3	331.32001	22182.3	450.80988	21	30185.8	331.2816	22185.8	30185.8
22	30416	328.77433	22416	446.10992	22	30421.6	328.71381	22421.6	30421.6
23	30416	328.77433	22416	446.10992	23	30421.6	328.71381	22421.6	30421.6
24	30442.1	328.49245	22442.1	445.5911	24	30448	328.4288	22448	30448
25	30442.1	328.49245	22442.1	445.5911	25	30448	328.4288	22448	30448
26	30507.2	327.79147	22507.2	444.30227	26	30509.4	327.76784	22509.4	30509.4
27	30507.2	327.79147	22507.2	444.30227	27	30509.4	327.76784	22509.4	30509.4
28	30518.2	327.67332	22518.2	444.08523	28	30520.7	327.64648	22520.7	30520.7
29	30518.2	327.67332	22518.2	444.08523	29	30520.7	327.64648	22520.7	30520.7
30	31099.6	321.54754	23099.6	432.90793	30	31102.5	321.51756	23102.5	31102.5
31	31099.6	321.54754	23099.6	432.90793	31	31102.5	321.51756	23102.5	31102.5
32	31102.9	321.51343	23102.9	432.84609	32	31105.7	321.48449	23105.7	31105.7
33	31102.9	321.51343	23102.9	432.84609	33	31105.7	321.48449	23105.7	31105.7
34	31108.5	321.45555	23108.5	432.7412	34	31110.3	321.43695	23110.3	31110.3
35	31108.5	321.45555	23108.5	432.7412	35	31110.3	321.43695	23110.3	31110.3
36	31112.4	321.41526	23112.4	432.66818	36	31114.1	321.39769	23114.1	31114.1

Table S36 (part 2/7) Comparison of the energies (*E*) of ground and excited states (the latter corresponding to optical transition energies from the ground sextet state, Fig. S35) obtained from CASSCF-SOC-type *ab initio* calculations for Mn1^{opt} and Mn1 models of octahedral Mn(II) complexes of **1** (Figure 1 and S66) when taking into account the quartet and doublet excited states, shown together with the resulting wavelengths of optical transitions and the values of energies and wavelengths after the scaling procedure (energy shift for scaling set at –8000 cm^{-1}, see the Comment on theoretical calculations of optical transitions in Mn(II) complexes in **1** above).

**Mn1^{opt}** complexes					**Mn1** complexes				
State	*E* / cm^{-1}	Wavelength / nm	Scaled *E* / cm^{-1}	Scaled wavelength / nm	State	*E* / cm^{-1}	Wavelength / nm	Scaled *E* / cm^{-1}	Scaled wavelength / nm
37	31112.4	321.41526	23112.4	432.66818	37	31114.1	321.39769	23114.1	432.63636
38	31180.8	320.71018	23180.8	431.3915	38	31182.5	320.6927	23182.5	431.35986
39	31180.8	320.71018	23180.8	431.3915	39	31182.5	320.6927	23182.5	431.35986
40	31182.8	320.68961	23182.8	431.35428	40	31184.6	320.6711	23184.6	431.32079
41	31182.8	320.68961	23182.8	431.35428	41	31184.6	320.6711	23184.6	431.32079
42	37086.4	269.64062	29086.4	343.80329	42	37091.4	269.60427	29091.4	343.7442
43	37086.4	269.64062	29086.4	343.80329	43	37091.4	269.60427	29091.4	343.7442
44	37196.1	268.84539	29196.1	342.5115	44	37199.7	268.81937	29199.7	342.46927
45	37196.1	268.84539	29196.1	342.5115	45	37199.7	268.81937	29199.7	342.46927
46	37340.2	267.80789	29340.2	340.82931	46	37342.8	267.78924	29342.8	340.79911
47	37340.2	267.80789	29340.2	340.82931	47	37342.8	267.78924	29342.8	340.79911
48	37489.8	266.73922	29489.8	339.1003	48	37493.8	266.71076	29493.8	339.05431
49	37489.8	266.73922	29489.8	339.1003	49	37493.8	266.71076	29493.8	339.05431
50	37648.1	265.61765	29648.1	337.28974	50	37649.5	265.60778	29649.5	337.27382
51	37648.1	265.61765	29648.1	337.28974	51	37649.5	265.60778	29649.5	337.27382
52	37751.9	264.88733	29751.9	336.11299	52	37755	264.86558	29755	336.07797
53	37751.9	264.88733	29751.9	336.11299	53	37755	264.86558	29755	336.07797
54	38260.5	261.36616	30260.5	330.46381	54	38254.8	261.4051	30254.8	330.52607
55	38260.5	261.36616	30260.5	330.46381	55	38254.8	261.4051	30254.8	330.52607
56	38369.8	260.62163	30369.8	329.27448	56	38363.9	260.66172	30363.9	329.33846
57	38369.8	260.62163	30369.8	329.27448	57	38363.9	260.66172	30363.9	329.33846
58	38496.7	259.76253	30496.7	327.90433	58	38494.9	259.77467	30494.9	327.92369
59	38496.7	259.76253	30496.7	327.90433	59	38494.9	259.77467	30494.9	327.92369
60	38604	259.04051	30604	326.75467	60	38599.4	259.07138	30599.4	326.80379
61	38604	259.04051	30604	326.75467	61	38599.4	259.07138	30599.4	326.80379
62	38786.4	257.82233	30786.4	324.81875	62	38786.7	257.82034	30786.7	324.81559
63	38786.4	257.82233	30786.4	324.81875	63	38786.7	257.82034	30786.7	324.81559
64	38829.2	257.53814	30829.2	324.36781	64	38840.9	257.46056	30840.9	324.24475
65	38829.2	257.53814	30829.2	324.36781	65	38840.9	257.46056	30840.9	324.24475
66	38871	257.2612	30871	323.92861	66	38875	257.23473	30875	323.88664
67	38871	257.2612	30871	323.92861	67	38875	257.23473	30875	323.88664
68	39185.6	255.19579	31185.6	320.66082	68	39201.8	255.09033	31201.8	320.49433
69	39185.6	255.19579	31185.6	320.66082	69	39201.8	255.09033	31201.8	320.49433
70	39386.6	253.89346	31386.6	318.6073	70	39387.4	253.8883	31387.4	318.59918
71	39386.6	253.89346	31386.6	318.6073	71	39387.4	253.8883	31387.4	318.59918
72	39545.8	252.87135	31545.8	316.99941	72	39546	252.87008	31546	316.9974

Table S36 (part 3/7) Comparison of the energies (*E*) of ground and excited states (the latter corresponding to optical transition energies from the ground sextet state, Fig. S35) obtained from CASSCF-SOC-type *ab initio* calculations for Mn1^{opt} and Mn1 models of octahedral Mn(II) complexes of **1** (Figure 1 and S66) when taking into account the quartet and doublet excited states, shown together with the resulting wavelengths of optical transitions and the values of energies and wavelengths after the scaling procedure (energy shift for scaling set at –8000 cm^{-1}, see the Comment on theoretical calculations of optical transitions in Mn(II) complexes in **1** above).

**Mn1^{opt}** complexes					**Mn1** complexes				
State	*E* / cm^{-1}	Wavelength / nm	Scaled *E* / cm^{-1}	Scaled wavelength / nm	State	*E* / cm^{-1}	Wavelength / nm	Scaled *E* / cm^{-1}	Scaled wavelength / nm
73	39545.8	252.87135	31545.8	316.99941	73	39546	252.87008	31546	316.9974
74	39711.6	251.81559	31711.6	315.34202	74	39717.5	251.77818	31717.5	315.28336
75	39711.6	251.81559	31711.6	315.34202	75	39717.5	251.77818	31717.5	315.28336
76	39780.1	251.38197	31780.1	314.66232	76	39784.4	251.3548	31784.4	314.61975
77	39780.1	251.38197	31780.1	314.66232	77	39784.4	251.3548	31784.4	314.61975
78	39962.8	250.23272	31962.8	312.8637	78	39978.9	250.13194	31978.9	312.70619
79	39962.8	250.23272	31962.8	312.8637	79	39978.9	250.13194	31978.9	312.70619
80	41569.5	240.56099	33569.5	297.88945	80	41578.5	240.50892	33578.5	297.80961
81	41569.5	240.56099	33569.5	297.88945	81	41578.5	240.50892	33578.5	297.80961
82	41849.6	238.95091	33849.6	295.42447	82	41859.1	238.89668	33859.1	295.34158
83	41849.6	238.95091	33849.6	295.42447	83	41859.1	238.89668	33859.1	295.34158
84	41993.8	238.13039	33993.8	294.17129	84	42003.1	238.07767	34003.1	294.09083
85	41993.8	238.13039	33993.8	294.17129	85	42003.1	238.07767	34003.1	294.09083
86	42270.9	236.56937	34270.9	291.79275	86	42279	236.52404	34279	291.7238
87	42270.9	236.56937	34270.9	291.79275	87	42279	236.52404	34279	291.7238
88	42380.7	235.95646	34380.7	290.86086	88	42389	235.91026	34389	290.79066
89	42380.7	235.95646	34380.7	290.86086	89	42389	235.91026	34389	290.79066
90	42705	234.16462	34705	288.14292	90	42713.8	234.11637	34713.8	288.06987
91	42705	234.16462	34705	288.14292	91	42713.8	234.11637	34713.8	288.06987
92	42856.4	233.33738	34856.4	286.89136	92	42865.3	233.28893	34865.3	286.81813
93	42856.4	233.33738	34856.4	286.89136	93	42865.3	233.28893	34865.3	286.81813
94	43511	229.82694	35511	281.60288	94	43517.7	229.79156	35517.7	281.54976
95	43511	229.82694	35511	281.60288	95	43517.7	229.79156	35517.7	281.54976
96	43556.4	229.58739	35556.4	281.24332	96	43561.5	229.56051	35561.5	281.20299
97	43556.4	229.58739	35556.4	281.24332	97	43561.5	229.56051	35561.5	281.20299
98	44340.7	225.52644	36340.7	275.17357	98	44342.9	225.51525	36342.9	275.15691
99	44340.7	225.52644	36340.7	275.17357	99	44342.9	225.51525	36342.9	275.15691
100	46948.6	212.9989	38948.6	256.74864	100	46953.8	212.97531	38953.8	256.71436
101	46948.6	212.9989	38948.6	256.74864	101	46953.8	212.97531	38953.8	256.71436
102	47354.3	211.17406	39354.3	254.10184	102	47359.8	211.14954	39359.8	254.06633
103	47354.3	211.17406	39354.3	254.10184	103	47359.8	211.14954	39359.8	254.06633
104	47567.2	210.2289	39567.2	252.73459	104	47572.4	210.20592	39572.4	252.70138
105	47567.2	210.2289	39567.2	252.73459	105	47572.4	210.20592	39572.4	252.70138
106	48107.3	207.86866	40107.3	249.33117	106	48113.3	207.84274	40113.3	249.29388
107	48107.3	207.86866	40107.3	249.33117	107	48113.3	207.84274	40113.3	249.29388
108	48391.3	206.64872	40391.3	247.57807	108	48396.8	206.62523	40396.8	247.54436

Table S36 (part 4/7) Comparison of the energies (*E*) of ground and excited states (the latter corresponding to optical transition energies from the ground sextet state, Fig. S35) obtained from CASSCF-SOC-type *ab initio* calculations for Mn1^{opt} and Mn1 models of octahedral Mn(II) complexes of **1** (Figure 1 and S66) when taking into account the quartet and doublet excited states, shown together with the resulting wavelengths of optical transitions and the values of energies and wavelengths after the scaling procedure (energy shift for scaling set at –8000 cm^{-1}, see the Comment on theoretical calculations of optical transitions in Mn(II) complexes in **1** above).

**Mn1^{opt}** complexes					**Mn1** complexes				
State	*E* / cm^{-1}	Wavelength / nm	Scaled *E* / cm^{-1}	Scaled wavelength / nm	State	*E* / cm^{-1}	Wavelength / nm	Scaled *E* / cm^{-1}	Scaled wavelength / nm
109	48391.3	206.64872	40391.3	247.57807	109	48396.8	206.62523	40396.8	247.54436
110	50203.7	199.18851	42203.7	236.94605	110	50215	199.14368	42215	236.88262
111	50203.7	199.18851	42203.7	236.94605	111	50215	199.14368	42215	236.88262
112	50365	198.55058	42365	236.0439	112	50373.6	198.51668	42373.6	235.996
113	50365	198.55058	42365	236.0439	113	50373.6	198.51668	42373.6	235.996
114	50674.2	197.33908	42674.2	234.33363	114	50679.5	197.31844	42679.5	234.30453
115	50674.2	197.33908	42674.2	234.33363	115	50679.5	197.31844	42679.5	234.30453
116	51974.5	192.40204	43974.5	227.40452	116	51978.8	192.38613	43978.8	227.38228
117	51974.5	192.40204	43974.5	227.40452	117	51978.8	192.38613	43978.8	227.38228
118	52038.2	192.16652	44038.2	227.07558	118	52040.9	192.15655	44040.9	227.06166
119	52038.2	192.16652	44038.2	227.07558	119	52040.9	192.15655	44040.9	227.06166
120	52104.5	191.922	44104.5	226.73423	120	52108.2	191.90838	44108.2	226.71521
121	52104.5	191.922	44104.5	226.73423	121	52108.2	191.90838	44108.2	226.71521
122	52184.7	191.62705	44184.7	226.32269	122	52188.3	191.61383	44188.3	226.30425
123	52184.7	191.62705	44184.7	226.32269	123	52188.3	191.61383	44188.3	226.30425
124	52284.2	191.26237	44284.2	225.81417	124	52287.8	191.2492	44287.8	225.79582
125	52284.2	191.26237	44284.2	225.81417	125	52287.8	191.2492	44287.8	225.79582
126	52352.5	191.01285	44352.5	225.46643	126	52356.4	190.99862	44356.4	225.44661
127	52352.5	191.01285	44352.5	225.46643	127	52356.4	190.99862	44356.4	225.44661
128	52445	190.67595	44445	224.99719	128	52445.6	190.67376	44445.6	224.99415
129	52445	190.67595	44445	224.99719	129	52445.6	190.67376	44445.6	224.99415
130	52629.8	190.00642	44629.8	224.06553	130	52634.1	189.9909	44634.1	224.04395
131	52629.8	190.00642	44629.8	224.06553	131	52634.1	189.9909	44634.1	224.04395
132	52747.6	189.58209	44747.6	223.47567	132	52750.9	189.57023	44750.9	223.45919
133	52747.6	189.58209	44747.6	223.47567	133	52750.9	189.57023	44750.9	223.45919
134	53003.9	188.66536	45003.9	222.20296	134	53001.3	188.67462	45001.3	222.2158
135	53003.9	188.66536	45003.9	222.20296	135	53001.3	188.67462	45001.3	222.2158
136	53070.8	188.42753	45070.8	221.87314	136	53073.9	188.41653	45073.9	221.85788
137	53070.8	188.42753	45070.8	221.87314	137	53073.9	188.41653	45073.9	221.85788
138	53089.3	188.36187	45089.3	221.78211	138	53091.1	188.35549	45091.1	221.77325
139	53089.3	188.36187	45089.3	221.78211	139	53091.1	188.35549	45091.1	221.77325
140	53799.6	185.87499	45799.6	218.34252	140	53802.7	185.86428	45802.7	218.32774
141	53799.6	185.87499	45799.6	218.34252	141	53802.7	185.86428	45802.7	218.32774
142	53951.8	185.35063	45951.8	217.61933	142	53956.4	185.33483	45956.4	217.59755
143	53951.8	185.35063	45951.8	217.61933	143	53956.4	185.33483	45956.4	217.59755
144	54008	185.15775	46008	217.3535	144	54016.6	185.12828	46016.6	217.31288

Table S36 (part 5/7) Comparison of the energies (*E*) of ground and excited states (the latter corresponding to optical transition energies from the ground sextet state, Fig. S35) obtained from CASSCF-SOC-type *ab initio* calculations for $Mn1^{opt}$ and Mn1 models of octahedral Mn(II) complexes of **1** (Figure 1 and S66) when taking into account the quartet and doublet excited states, shown together with the resulting wavelengths of optical transitions and the values of energies and wavelengths after the scaling procedure (energy shift for scaling set at –8000 cm^{-1}, see the Comment on theoretical calculations of optical transitions in Mn(II) complexes in **1** above).

**$Mn1^{opt}$** complexes					**Mn1** complexes				
State	*E* / cm^{-1}	Wavelength / nm	Scaled *E* / cm^{-1}	Scaled wavelength / nm	State	*E* / cm^{-1}	Wavelength / nm	Scaled *E* / cm^{-1}	Scaled wavelength / nm
145	54008	185.15775	46008	217.3535	145	54016.6	185.12828	46016.6	217.31288
146	54045	185.03099	46045	217.17885	146	54053.8	185.00087	46053.8	217.13735
147	54045	185.03099	46045	217.17885	147	54053.8	185.00087	46053.8	217.13735
148	54093.3	184.86578	46093.3	216.95127	148	54099.2	184.84562	46099.2	216.9235
149	54093.3	184.86578	46093.3	216.95127	149	54099.2	184.84562	46099.2	216.9235
150	54232.9	184.38992	46232.9	216.29619	150	54226.7	184.411	46226.7	216.3252
151	54232.9	184.38992	46232.9	216.29619	151	54226.7	184.411	46226.7	216.3252
152	54284.2	184.21566	46284.2	216.05645	152	54276.1	184.24316	46276.1	216.09427
153	54284.2	184.21566	46284.2	216.05645	153	54276.1	184.24316	46276.1	216.09427
154	54337.6	184.03463	46337.6	215.80747	154	54333.1	184.04987	46333.1	215.82843
155	54337.6	184.03463	46337.6	215.80747	155	54333.1	184.04987	46333.1	215.82843
156	54404.6	183.80799	46404.6	215.49588	156	54399.9	183.82387	46399.9	215.51771
157	54404.6	183.80799	46404.6	215.49588	157	54399.9	183.82387	46399.9	215.51771
158	54936.5	182.02834	46936.5	213.05381	158	54946.3	181.99588	46946.3	213.00933
159	54936.5	182.02834	46936.5	213.05381	159	54946.3	181.99588	46946.3	213.00933
160	55119.3	181.42466	47119.3	212.22726	160	55122.7	181.41346	47122.7	212.21195
161	55119.3	181.42466	47119.3	212.22726	161	55122.7	181.41346	47122.7	212.21195
162	55314.4	180.78475	47314.4	211.35215	162	55313	180.78933	47313	211.3584
163	55314.4	180.78475	47314.4	211.35215	163	55313	180.78933	47313	211.3584
164	55694.2	179.55191	47694.2	209.6691	164	55694.3	179.55159	47694.3	209.66866
165	55694.2	179.55191	47694.2	209.6691	165	55694.3	179.55159	47694.3	209.66866
166	56047.6	178.41977	48047.6	208.12694	166	56048.6	178.41659	48048.6	208.12261
167	56047.6	178.41977	48047.6	208.12694	167	56048.6	178.41659	48048.6	208.12261
168	56338.4	177.49883	48338.4	206.87487	168	56340.9	177.49095	48340.9	206.86417
169	56338.4	177.49883	48338.4	206.87487	169	56340.9	177.49095	48340.9	206.86417
170	56899	175.75001	48899	204.50316	170	56896.2	175.75866	48896.2	204.51487
171	56899	175.75001	48899	204.50316	171	56896.2	175.75866	48896.2	204.51487
172	57255.9	174.65449	49255.9	203.02136	172	57256.3	174.65327	49256.3	203.01972
173	57255.9	174.65449	49255.9	203.02136	173	57256.3	174.65327	49256.3	203.01972
174	57453	174.05531	49453	202.2122	174	57454.6	174.05047	49454.6	202.20566
175	57453	174.05531	49453	202.2122	175	57454.6	174.05047	49454.6	202.20566
176	57743.4	173.17997	49743.4	201.03169	176	57742.8	173.18176	49742.8	201.03412
177	57743.4	173.17997	49743.4	201.03169	177	57742.8	173.18176	49742.8	201.03412
178	57855.1	172.84561	49855.1	200.58128	178	57850.7	172.85876	49850.7	200.59899
179	57855.1	172.84561	49855.1	200.58128	179	57850.7	172.85876	49850.7	200.59899
180	58067.7	172.21278	50067.7	199.72957	180	58068.8	172.20952	50068.8	199.72518

Table S36 (part 6/7) Comparison of the energies (*E*) of ground and excited states (the latter corresponding to optical transition energies from the ground sextet state, Fig. S35) obtained from CASSCF-SOC-type *ab initio* calculations for Mn1^{opt} and Mn1 models of octahedral Mn(II) complexes of **1** (Figure 1 and S66) when taking into account the quartet and doublet excited states, shown together with the resulting wavelengths of optical transitions and the values of energies and wavelengths after the scaling procedure (energy shift for scaling set at –8000 cm^{-1}, see the Comment on theoretical calculations of optical transitions in Mn(II) complexes in **1** above).

**Mn1^{opt}** complexes					**Mn1** complexes				
State	*E* / cm^{-1}	Wavelength / nm	Scaled *E* / cm^{-1}	Scaled wavelength / nm	State	*E* / cm^{-1}	Wavelength / nm	Scaled *E* / cm^{-1}	Scaled wavelength / nm
181	58067.7	172.21278	50067.7	199.72957	181		172.20952	50068.8	199.72518
182	58343.1	171.39987	50343.1	198.63695	182	58342.6	171.40134	50342.6	198.63893
183	58343.1	171.39987	50343.1	198.63695	183	58342.6	171.40134	50342.6	198.63893
184	58471.6	171.0232	50471.6	198.13123	184	58470.1	171.02759	50470.1	198.13711
185	58471.6	171.0232	50471.6	198.13123	185	58470.1	171.02759	50470.1	198.13711
186	59177.2	168.984	51177.2	195.39951	186	59172.2	168.99828	51172.2	195.41861
187	59177.2	168.984	51177.2	195.39951	187	59172.2	168.99828	51172.2	195.41861
188	59608.9	167.76018	51608.9	193.76503	188	59615.9	167.74049	51615.9	193.73875
189	59608.9	167.76018	51608.9	193.76503	189	59615.9	167.74049	51615.9	193.73875
190	59801.5	167.21989	51801.5	193.0446	190	59806.6	167.20563	51806.6	193.0256
191	59801.5	167.21989	51801.5	193.0446	191	59806.6	167.20563	51806.6	193.0256
192	61128.2	163.59062	53128.2	188.22396	192	61129	163.58848	53129	188.22112
193	61128.2	163.59062	53128.2	188.22396	193	61129	163.58848	53129	188.22112
194	61237.7	163.2981	53237.7	187.83681	194	61234.6	163.30637	53234.6	187.84775
195	61237.7	163.2981	53237.7	187.83681	195	61234.6	163.30637	53234.6	187.84775
196	61298.6	163.13586	53298.6	187.62219	196	61295.3	163.14465	53295.3	187.63381
197	61298.6	163.13586	53298.6	187.62219	197	61295.3	163.14465	53295.3	187.63381
198	61701.9	162.06956	53701.9	186.21315	198	61707.7	162.05433	53707.7	186.19304
199	61701.9	162.06956	53701.9	186.21315	199	61707.7	162.05433	53707.7	186.19304
200	61811.1	161.78324	53811.1	185.83526	200	61813.1	161.778	53813.1	185.82836
201	61811.1	161.78324	53811.1	185.83526	201	61813.1	161.778	53813.1	185.82836
202	61924.4	161.48723	53924.4	185.44481	202	61921.5	161.4948	53921.5	185.45478
203	61924.4	161.48723	53924.4	185.44481	203	61921.5	161.4948	53921.5	185.45478
204	62316.7	160.47063	54316.7	184.10544	204	62311.1	160.48505	54311.1	184.12442
205	62316.7	160.47063	54316.7	184.10544	205	62311.1	160.48505	54311.1	184.12442
206	66306	150.81591	58306	171.50894	206	66310.2	150.80636	58310.2	171.49658
207	66306	150.81591	58306	171.50894	207	66310.2	150.80636	58310.2	171.49658
208	74607.9	134.03406	66607.9	150.13234	208	74611.1	134.02831	66611.1	150.12513
209	74607.9	134.03406	66607.9	150.13234	209	74611.1	134.02831	66611.1	150.12513
210	74635.5	133.9845	66635.5	150.07016	210	74639.3	133.97768	66639.3	150.0616
211	74635.5	133.9845	66635.5	150.07016	211	74639.3	133.97768	66639.3	150.0616
212	75140.4	133.0842	67140.4	148.94162	212	75146.1	133.0741	67146.1	148.92898
213	75140.4	133.0842	67140.4	148.94162	213	75146.1	133.0741	67146.1	148.92898
214	75281.7	132.83441	67281.7	148.62882	214	75288	132.82329	67288	148.61491
215	75281.7	132.83441	67281.7	148.62882	215	75288	132.82329	67288	148.61491
216	75443.2	132.55005	67443.2	148.27292	216	75446.5	132.54425	67446.5	148.26566

Table S36 (part 7/7) Comparison of the energies (*E*) of ground and excited states (the latter corresponding to optical transition energies from the ground sextet state, Fig. S35) obtained from CASSCF-SOC-type *ab initio* calculations for Mn1^{opt} and Mn1 models of octahedral Mn(II) complexes of **1** (Figure 1 and S66) when taking into account the quartet and doublet excited states, shown together with the resulting wavelengths of optical transitions and the values of energies and wavelengths after the scaling procedure (energy shift for scaling set at –8000 cm^{-1}, see the Comment on theoretical calculations of optical transitions in Mn(II) complexes in **1** above).

**Mn1**^{opt} complexes					**Mn1** complexes				
State	*E* / cm^{-1}	Wavelength / nm	Scaled *E* / cm^{-1}	Scaled wavelength / nm	State	*E* / cm^{-1}	Wavelength / nm	Scaled *E* / cm^{-1}	Scaled wavelength / nm
217	75443.2	132.55005	67443.2	148.27292	217	75446.5	132.54425	67446.5	148.26566
218	81519.5	122.67004	73519.5	136.01834	218	81530.2	122.65394	73530.2	135.99854
219	81519.5	122.67004	73519.5	136.01834	219	81530.2	122.65394	73530.2	135.99854
220	81619.2	122.52019	73619.2	135.83413	220	81622.6	122.51509	73622.6	135.82786
221	81619.2	122.52019	73619.2	135.83413	221	81622.6	122.51509	73622.6	135.82786
222	82145.6	121.73507	74145.6	134.86977	222	82151.6	121.72617	74151.6	134.85886
223	82145.6	121.73507	74145.6	134.86977	223	82151.6	121.72617	74151.6	134.85886
224	82223.4	121.61988	74223.4	134.7284	224	82226.6	121.61515	74226.6	134.72259
225	82223.4	121.61988	74223.4	134.7284	225	82226.6	121.61515	74226.6	134.72259
226	82522.4	121.17922	74522.4	134.18784	226	82528.9	121.16968	74528.9	134.17614
227	82522.4	121.17922	74522.4	134.18784	227	82528.9	121.16968	74528.9	134.17614
228	82827	120.73358	74827	133.6416	228	82830.4	120.72862	74830.4	133.63553
229	82827	120.73358	74827	133.6416	229	82830.4	120.72862	74830.4	133.63553
230	82850.5	120.69933	74850.5	133.59964	230	82852	120.69715	74852	133.59696
231	82850.5	120.69933	74850.5	133.59964	231	82852	120.69715	74852	133.59696
232	82873	120.66656	74873	133.55949	232	82873.2	120.66627	74873.2	133.55914
233	82873	120.66656	74873	133.55949	233	82873.2	120.66627	74873.2	133.55914
234	83403.3	119.89933	75403.3	132.62019	234	83404.6	119.89746	75404.6	132.6179
235	83403.3	119.89933	75403.3	132.62019	235	83404.6	119.89746	75404.6	132.6179
236	100293.4	99.70746	92293.4	108.35011	236	100294.3	99.70656	92294.3	108.34905
237	100293.4	99.70746	92293.4	108.35011	237	100294.3	99.70656	92294.3	108.34905
238	100455.9	99.54617	92455.9	108.15967	238	100461	99.54112	92461	108.15371
239	100455.9	99.54617	92455.9	108.15967	239	100461	99.54112	92461	108.15371
240	100639	99.36506	92639	107.9459	240	100644.3	99.35982	92644.3	107.93972
241	100639	99.36506	92639	107.9459	241	100644.3	99.35982	92644.3	107.93972
242	108858.4	91.86246	100858.4	99.14891	242	108863.5	91.85815	100863.5	99.14389
243	108858.4	91.86246	100858.4	99.14891	243	108863.5	91.85815	100863.5	99.14389
244	108951.9	91.78362	100951.9	99.05708	244	108965.5	91.77217	100965.5	99.04373
245	108951.9	91.78362	100951.9	99.05708	245	108965.5	91.77217	100965.5	99.04373
246	109025.9	91.72132	101025.9	98.98452	246	109034.8	91.71384	101034.8	98.9758
247	109025.9	91.72132	101025.9	98.98452	247	109034.8	91.71384	101034.8	98.9758
248	109095.1	91.66315	101095.1	98.91676	248	109101.2	91.65802	101101.2	98.91079
249	109095.1	91.66315	101095.1	98.91676	249	109101.2	91.65802	101101.2	98.91079
250	109431.3	91.38153	101431.3	98.5889	250	109438.5	91.37552	101438.5	98.5819
251	109431.3	91.38153	101431.3	98.5889	251	109438.5	91.37552	101438.5	98.5819

Table S37 Comparison of the energies of excited states (corresponding to optical transition energies from the ground sextet state, *ΔE*, Fig. S35) obtained from CASSCF-NEVPT2-type *ab initio* calculations for $Mn1^{opt}$ and Mn1 models of octahedral Mn(II) complexes of **1** (Figure 1 and S66) when taking into account the quartet and doublet excited states, shown together with the resulting wavelengths of optical transitions and the values of energies and wavelengths after the scaling procedure (energy shift for scaling set at –5000 cm^{-1}, see the Comment on theoretical calculations of optical transitions in Mn(II) complexes in **1** above).

**Mn1**^{opt} complexes					**Mn1** complexes				
State	ΔE / cm^{-1}	Wavelength / nm	Scaled ΔE / cm^{-1}	Scaled wavelength / nm	State	ΔE / cm^{-1}	Wavelength / nm	Scaled ΔE / cm^{-1}	Scaled wavelength / nm
1	23318.2	428.84957	18318.2	545.90517	1	23341.6	428.41965	18341.6	545.20871
2	23332.5	428.58674	18332.5	545.47934	2	23352	428.22885	18352	544.89974
3	23850.7	419.27491	18850.7	530.48428	3	23867.6	418.97803	18867.6	530.00912
4	26690.3	374.66795	21690.3	461.03558	4	26700.2	374.52903	21700.2	460.82525
5	27029.7	369.96341	22029.7	453.93265	5	27043.2	369.77872	22043.2	453.65464
6	27122.2	368.70165	22122.2	452.03461	6	27128.6	368.61467	22128.6	451.90387
7	28034.4	356.70462	23034.4	434.1333	7	28041.7	356.61176	23041.7	433.99576
8	28041.6	356.61303	23041.6	433.99764	8	28046.3	356.55327	23046.3	433.90913
9	28131.8	355.46961	23131.8	432.30531	9	28137.3	355.40013	23137.3	432.20255
Due to the computational limitations further states were not calculated for $Mn1^{opt}$ complexes in this approach.					Due to the computational limitations further states were not calculated for Mn1 complexes in this approach.				

Table S38 (part 1/2) Comparison of the energies (*E*) of ground and excited states (the latter corresponding to optical transition energies from the ground sextet state, Fig. S35) obtained from CASSCF-NEVPT2-SOC-type *ab initio* calculations for Mn1^{opt} and Mn1 models of octahedral Mn(II) complexes of **1** (Figure 1 and S66) when taking into account the quartet and doublet excited states, shown together with the resulting wavelengths of optical transitions and the values of energies and wavelengths after the scaling procedure (energy shift for scaling set at –5000 cm^{-1}, see the Comment on theoretical calculations of optical transitions in Mn(II) complexes in **1** above).

**Mn1^{opt}** complexes					**Mn1** complexes				
State	*E* / cm^{-1}	Wavelength / nm	Scaled *E* / cm^{-1}	Scaled wavelength / nm	State	*E* / cm^{-1}	Wavelength / nm	Scaled *E* / cm^{-1}	Scaled wavelength / nm
1	0	-	-5000	-	1	0	-	-5000	-
2	0	-	-5000	-	2	0	-	-5000	-
3	0	-	-5000	-	3	0	-	-5000	-
4	0	-	-5000	-	4	0	-	-5000	-
5	0	-	-5000	-	5	0	-	-5000	-
6	23309.3	429.01331	18309.3	546.17053	6	23331	428.61429	18331	545.52398
7	23309.3	429.01331	18309.3	546.17053	7	23331	428.61429	18331	545.52398
8	23322.7	428.76682	18322.7	545.77109	8	23345.2	428.35358	18345.2	545.10172
9	23322.7	428.76682	18322.7	545.77109	9	23345.2	428.35358	18345.2	545.10172
10	23343.9	428.37743	18343.9	545.14035	10	23364.2	428.00524	18364.2	544.53774
11	23343.9	428.37743	18343.9	545.14035	11	23364.2	428.00524	18364.2	544.53774
12	23358.5	428.10968	18358.5	544.70681	12	23379.7	427.72148	18379.7	544.07852
13	23358.5	428.10968	18358.5	544.70681	13	23379.7	427.72148	18379.7	544.07852
14	23858.3	419.14135	18858.3	530.27049	14	23875	418.84817	18875	529.80132
15	23858.3	419.14135	18858.3	530.27049	15	23875	418.84817	18875	529.80132
16	23860.5	419.1027	18860.5	530.20864	16	23877.2	418.80958	18877.2	529.73958
17	23860.5	419.1027	18860.5	530.20864	17	23877.2	418.80958	18877.2	529.73958
18	26684.9	374.74377	21684.9	461.15039	18	26694.9	374.60339	21694.9	460.93782
19	26684.9	374.74377	21684.9	461.15039	19	26694.9	374.60339	21694.9	460.93782
20	26692.3	374.63988	21692.3	460.99307	20	26702.2	374.50098	21702.2	460.78278
21	26692.3	374.63988	21692.3	460.99307	21	26702.2	374.50098	21702.2	460.78278
22	27024.4	370.03597	22024.4	454.04188	22	27037.1	369.86215	22037.1	453.78022
23	27024.4	370.03597	22024.4	454.04188	23	27037.1	369.86215	22037.1	453.78022
24	27040.8	369.81154	22040.8	453.70404	24	27054	369.63111	22054	453.43248
25	27040.8	369.81154	22040.8	453.70404	25	27054	369.63111	22054	453.43248
26	27133.3	368.55082	22133.3	451.80791	26	27139.9	368.4612	22139.9	451.67322
27	27133.3	368.55082	22133.3	451.80791	27	27139.9	368.4612	22139.9	451.67322
28	27142.3	368.42862	22142.3	451.62427	28	27149.4	368.33227	22149.4	451.4795
29	27142.3	368.42862	22142.3	451.62427	29	27149.4	368.33227	22149.4	451.4795
30	28039.4	356.64101	23039.4	434.03908	30	28046	356.55708	23046	433.91478
31	28039.4	356.64101	23039.4	434.03908	31	28046	356.55708	23046	433.91478
32	28041.1	356.61939	23041.1	434.00706	32	28048.2	356.52912	23048.2	433.87336
33	28041.1	356.61939	23041.1	434.00706	33	28048.2	356.52912	23048.2	433.87336
34	28048.9	356.52022	23048.9	433.86018	34	28053.8	356.45795	23053.8	433.76797
35	28048.9	356.52022	23048.9	433.86018	35	28053.8	356.45795	23053.8	433.76797
36	28050.5	356.49988	23050.5	433.83007	36	28055.9	356.43127	23055.9	433.72846

Table S38 (part 2/2) Comparison of the energies (*E*) of ground and excited states (the latter corresponding to optical transition energies from the ground sextet state, Fig. S35) obtained from CASSCF-NEVPT2-SOC-type *ab initio* calculations for Mn1^{opt} and Mn1 models of octahedral Mn(II) complexes of **1** (Figure 1 and S66) when taking into account the quartet and doublet excited states, shown together with the resulting wavelengths of optical transitions and the values of energies and wavelengths after the scaling procedure (energy shift for scaling set at –5000 cm^{-1}, see the Comment on theoretical calculations of optical transitions in Mn(II) complexes in **1** above).

**Mn1^{opt}** complexes					**Mn1** complexes				
State	*E* / cm^{-1}	Wavelength / nm	Scaled *E* / cm^{-1}	Scaled wavelength / nm	State	*E* / cm^{-1}	Wavelength / nm	Scaled *E* / cm^{-1}	Scaled wavelength / nm
37	28050.5	356.49988	23050.5	433.83007	37	28055.9	356.43127	23055.9	433.72846
38	28138	355.39129	23138	432.18947	38	28143.3	355.32436	23143.3	432.0905
39	28138	355.39129	23138	432.18947	39	28143.3	355.32436	23143.3	432.0905
40	28138.1	355.39002	23138.1	432.1876	40	28143.5	355.32183	23143.5	432.08676
41	28138.1	355.39002	23138.1	432.1876	41	28143.5	355.32183	23143.5	432.08676
Due to the computational limitations further states were not calculated for Mn1^{opt} complexes in this approach.					Due to the computational limitations further states were not calculated for Mn1 complexes in this approach.				

References to the Supporting Information

S1 M. J. Petersson, W. A. Loughlin and I. D. Jenkins, *Chem. Commun.*, 2008, **37**, 4493–4494.

S2 S. O. Grim, L. C. Satek, C. A. Tolman, and J. P. Jesson, *Inorg. Chem.* 1975, **14**, 656–660.

S3 G. M. Sheldrick, TWINABS; University of Göttingen: Göttingen, Germany, 2009.

S4 G. M. Sheldrick, *Acta Crystallogr., Sect. A: Found. Adv.,* 2015, **71**, 3–8.

S5 L. J. Farrugia, *J. Appl. Crystallogr.,* 2012, **45**, 849–854.

S6 R. Jankowski, J. J. Zakrzewski, M. Zychowicz, J. Wang, Y. Oki, S. Ohkoshi, S. Chorazy and B. Sieklucka, *J. Mater. Chem. C*, 2021, **9**, 10705–10717.

S7 B. J. Rodriguez, C. Callahan, S. Kalinin and R. Proksch, *Nanotechnology*, 2007, **18**, 475504.

S8 S. Chorazy, J. J. Zakrzewski, J. Wang, S. Ohkoshi and B. Sieklucka, *CrystEngComm*, 2018, **20**, 5695–5706.

S9 M. Llunell, D. Casanova, J. Cirera, J. Bofill, P. Alemany, S. Alvarez, M. Pinsky and D. Avnir, *SHAPE v. 2.1. Program for the Calculation of Continuous Shape Measures of Polygonal and Polyhedral Molecular Fragments*, University of Barcelona: Barcelona, Spain, 2013.

S10 M. Liberka, M. Zychowicz, W. Zychowicz and S. Chorazy, *Chem. Commun*, 2022, **58**, 6381–6384.

S11 D. Casanova, J. Cirera, M. Llunell, P. Alemany, D. Avnir and S. Alvarez, *J. Am. Chem. Soc.*, 2004, **126**, 1755–1763.

S12 F. Neese, *WIREs Comput Mol Sci.,* 2012, **2**, 73–78.

S13 F. Neese, *WIREs Comput Mol Sci.,* 2022, **12**, e1606.

S14 J. Zheng, X. Xu and D. G Truhlar, *Theor. Chem. Acc.*, 2010, **128**, 295–305.

S15 C. Kollmar, K. Sivalingam, B. Helmich-Paris, C. Angeli and F. Neese, *J. Comput. Chem.*, 2019, **40**, 1463–1470.

S16 C. Angeli, R. Cimiraglia and J. P. Malrieu, *J. Chem. Phys.*, 2002, **117**, 9138–9153.

S17 G. L. Stoychev, A. A. Auer and F. Neese, *J. Chem. Theory Comput.*, 2017, **13**, 554–562.

S18 N. Bjerrum, *Science*, 1952, **115**, 385–390.

S19 Y. Sugimoto, *Science*, 2022, **377**, 264–265.

S20 A. R. von Hippel, *IEEE Trans. Electr. Insul.*, 1988, **23**, 801–816.

S21 U. Kaatze, in *Dielectric Relaxation in Biological Systems*, Oxford University Press, 2015, pp. 189–227.

S22 I. Popov, A. Puzenko, A. Khamzin and Y. Feldman, *Phys. Chem. Chem. Phys.*, 2015, **17**, 1489–1497.

S23 I. Popov, P. Ben Ishai, A. Khamzin and Y. Feldman, *Phys. Chem. Chem. Phys.*, 2016, **18**, 13941–13953.

S24 M. A. Asghar, S. Zhang, T. Khan, Z. Sun, A. Zeb, C. Ji, L. Li, S. Zhao and J. Luo, *J. Mater. Chem. C*, 2016, **4**, 7537–7540.

S25 W.-L. Yang, X. Yan, M. Wang, H. Yuan, Y.-Y. Tang, Y. Qin and X.-J. Song, *Inorg. Chem. Front.*, 2024, **11**, 6874–6879.

S26 W. L. Steffen and G. J. Palenik, *Inorg. Chem.*, 1977, **16**, 1119–1127.

S27 Y. Liu, L. Liu, X. Zhang, G. Liang and X. Gong, *Acta Cryst.*, 2018, **C74**, 13–20.

S28 C. Näther, I. Jass and S. Mangelsen, *Z. Naturforsch. B*, 2023, **78**, 113–120.

S29 Y. Qin, P. She, X. Huang, W. Huang and Q. Zhao, *Coord. Chem. Rev.*, 2020, **416**, 213331.
